# The Burden of Parkinson’s Disease Based on the GBD 2021

**DOI:** 10.3389/ijph.2026.1608863

**Published:** 2026-02-24

**Authors:** Huiqun Yang, Liyuan Pu, Tian Zhao, Lifang Pan, Yuanbo Jiang, Liyuan Han, Qiongfeng Guan

**Affiliations:** 1 Department of Clinical Epidemiology, Ningbo 2 Hospital, Ningbo, Zhejiang, China; 2 Center for Cardiovascular and Cerebrovascular Epidemiology and Translational Medicine, Guoke Ningbo Life Science and Health Industry Research Institute, University of Chinese Academy of Sciences, Ningbo, Zhejiang, China; 3 Ningbo No. 2 Hospital, Ningbo, Zhejiang, China

**Keywords:** age-period-cohort, decomposition analysis, Parkinson’s disease, prevalance, temporal trends

## Abstract

**Objective:**

To examine global temporal trends in Parkinson’s disease (PD) prevalence from 1992 to 2021, providing a foundation for targeted prevention and control strategies for neurodegenerative disorder.

**Methods:**

Data from the Global Burden of Disease Study 2021 were analyzed using an age-period-cohort model to assess temporal trends in PD at global levels. Decomposition analysis evaluated the influence of population aging, growth and epidemiological transitions on disease burden.

**Results:**

From 1992 to 2021, the global number of PD cases increased from 3,471,682.09 to 11,756,618.58. The age-standardized prevalence rose from 191.38 per 100,000 to 292.93 per 100,000 population, with a global net drift value of 1.42%. Globally, PD prevalence increased and then declined with age, though some regions showed continuous rise. Period and cohort effects suggested increasing relative risk worldwide and in several regions. Decomposition analysis identified population growth as the primary driver of the increasing global PD burden.

**Conclusion:**

PD prevalence and age-standardized rates increased globally and regionally, with a marked surge among individuals aged 60+. Underscoring the need for region-tailored strategies aligned with World Health Organization’s objectives for 2030.

## Introduction

Parkinson’s disease (PD) is a progressive neurodegenerative disorder predominantly affecting middle-aged and older adults, with onset most commonly occurring after age 50. The risk of developing PD increases with age and peaks between 85 and 94 years, while large-scale epidemiological studies estimate the median age of onset to be approximately 75.4 years [1]. PD is characterized by degeneration of dopaminergic neurons in the substantia nigra, resulting in considerable health challenges and economic burdens for patients, families, and society [2]. In early stages, patients typically present with motor symptoms such as tremor, dyskinesia, and rigidity. As the disease advances, non-motor symptoms including constipation, anosmia, and cognitive dysfunction, such as memory impairment and difficulty in decision-making, become increasingly prominent [3]. According to the Global Burden of Disease Study 2021 (GBD 2021), an estimated 11.77 million individuals worldwide were living with PD, representing a dramatic 274% increase from 3.15 million cases in 1990. Over this period, both disability-adjusted life years and mortality rates associated with PD have risen steadily [4, 5]. Projections suggest that the global number of PD cases will reach 25.2 million by 2050 [6]. Against the backdrop of global population is aging, PD remains one of the fastest-growing neurological disorders and is poised to become a major global health challenge in the future.

A comprehensive analysis of global, regional, and national trends in PD prevalence is essential to understand its epidemiological profile, monitor disease management, and prioritize future interventions. Temporal trends reflect the interplay of multiple dimensions, as documented in several studies, and help elucidate the dynamics of disease epidemics [7]. Notably, the correlation between PD prevalence and age is especially pronounced among older adults, with disease burden rising substantially advancing age [8]. Period effects capture the influence of societal, technological, medical, and public health policy changes on disease prevalence [9, 10]. Meanwhile, birth-cohort effects highlight the long-term impact of early-life socioeconomic conditions, health exposures, and behaviors on disease risk in later life [11]. By carefully examining the interactions among these factors, we can advance our understanding of disease pathogenesis, thereby providing a robust theoretical foundation for prevention, management, and evidence-informed-making.

Although several studies have reported on the global prevalence of PD [4, 5], few have adequately addressed the relative contributions of age, period, and birth cohort to incidence trends. While the age-period-cohort (APC) model has been applied to analyze the independent effects of these factors on PD incidence in China [12], systematic analyses of PD prevalence trends and their associations with age, period, or birth cohort remain lacking in many high-, middle-, and low-income countries. Utilizing data from the GBD 2021, this study employs the APC model to evaluate the effects of age, period, and birth cohort on PD burden at global, regional, and national levels. The aim is to investigate long-term PD trends under the influence of these distinct factors and to provide a scientific basis for developing effective disease prevention and treatment strategies.

## Methods

### Data Sources and Definitions

This study conducted a secondary analysis using the latest publicly available data from the GBD 2021. It provides comprehensive global data spanning 204 countries and regions from 1990 to 2021, systematically categorized by 25 age groups (from birth to ≥95 years), sex, and geographic region. Given the extremely low and unstable prevalence estimates for PD in individuals under 30 years of age, the present analysis focuses on the population aged 30 years and older, thereby capturing the majority of the disease burden. At the same time, in recognition of shifts in modern lifestyles and environmental exposures that may contribute to earlier symptom onset in some groups, this study adopts 30 years as the lower age limit to balance epidemiological plausibility with consideration of potential early-onset risk [13]. Age-standardized indicators were calculated using global standard population weights [14].

The GBD applies a standardized case definition for PD, which includes only idiopathic PD presenting with at least two of the following clinical features: resting tremor, bradykinesia, rigidity, or postural instability. This definition explicitly excludes secondary and drug-induced Parkinsonian syndromes, and aligns with the International Classification of Diseases, 10th Revision (ICD-10) codes G20, G21, and G22. Data sources include multiple types of information, such as death registries, hospital and insurance claims records, and epidemiological surveys. These heterogeneous data are integrated using the DisMod MR 2.1 Bayesian meta-regression model to ensure consistency across incidence, prevalence, remission, and cause-specific mortality rates. For nonspecific or unclear ICD cause-of-death codes (“garbage codes”), GBD employs evidence-based garbage-code redistribution combined with bias correction techniques to further refine estimates, thereby enhancing accuracy and improving the comparability of cause-specific mortality data across regions and populations. This study directly adopts the final modeling results from GBD 2021 without modifying the original input data. Additionally, national development levels are incorporated using the Socio-demographic Index (SDI), a composite measure reflecting years of education among individuals under 25, *per capita* income, and total fertility rate. The SDI classifies countries or regions into five tiers: Low, Lower-middle, Middle, High-middle, and High socioeconomic development.

GBD 2021 strictly adheres to the Guidelines for Accurate and Transparent Health Estimates Reporting [15]. This study, as a secondary analysis of publicly available data, complies with the GBD data usage protocol.

### Age-Period-Cohort Model (APC) Model

This study extracted PD prevalence and corresponding population data from the GBD 2021 database to serve as input variables for the APC model. The data were stratified by age group (30–34 to ≥95 years), period (1992–1996 to 2017–2021), and birth cohort (1897–1901 to 1987–1991). An APC model was fitted within a log-linear Poisson regression framework to analyze relative trends in disease prevalence across the three temporal dimensions—age, period, and birth cohort. In this model, the age effect reflects age-related differences in prevalence after adjusting for period and cohort effects. The period effect represents the common influence of external environmental factors on all age groups over time, expressed as relative risk (RR) compared with a reference period. The cohort effect captures risk differences across birth cohorts arising from shared exposure histories. Based on model estimates, net drift and local drift were further calculated. Net drift represents the average annual percentage change across the entire study population, whereas local drift indicates the age-group-specific average annual trend. Due to the intrinsic linear dependency among age, period, and cohort, the Holford decomposition method was applied. This approach decomposes temporal trends into net drift, local drift, and deviations attributable to age, period, and cohort by imposing appropriate constraints, yielding unique, stable, and epidemiologically interpretable effect estimates. All analyses were conducted using R software (version 4.3), APC parameter estimation was performed based on scripts published by the National Cancer Institute’s APC Web Tool.

The basic equation of the model is as follows [16, 17]:

$$\ln\left(\frac{y_{ij}}{n_{ij}}\right)=\mu +\alpha_{i}+\beta_{j}+\gamma_{k}+\varepsilon_{ij},k=j-i$$

In this equation, *y*_*ij*_ denotes the number of cases in the i-th age group during the j-th period, while *n*_*ij*_ represents the corresponding exposed population size. The intercept is denoted by u , and $\alpha_{i}$ , $\beta_{j}$ , and $\gamma_{k}$ represent the age, period, and cohort effects, respectively. The random error term is denoted by $\varepsilon_{ij}$ .

### Decomposition Analysis

To assess the drivers of changes in PD burden from 1992 to 2021, we employed the Das Gupta decomposition method [18, 19]. This technique decomposes the overall change in disease burden into three distinct components: population aging, population growth, and epidemiological change. Unlike other decomposition approaches, the Das Gupta method avoids sequence-related bias by using an additive and internally consistent framework, which clearly distinguishes the independent contributions of demographic and epidemiological factors. Here epidemiological changes refers to shifts in disease prevalence over time while holding both population size and age structure constant, thereby capturing genuine temporal trends in disease risk, advances in medical diagnosis, and other non-demographic influences.

The calculation follows the standard Das Gupta formulation:

$$X_{a,y}=\sum_{i=1}^{n}\left(a_{i,y}\cdot p_{y}\cdot e_{i,y}\right)$$

Where *X*_*a,y*_ denotes the disease burden measure for year *y*, determined by the age structure, total population, and age-specific prevalence. *a*_*i,y*_ represents the proportion of the population in age group *i*, *p*_*y*_ is the total population size in year *y*, and *e*_*i,y*_ denotes the age-specific prevalence for age group *i*. By systematically varying one factor while keeping the others fixed, we quantified the independent contribution of each component to the total change in PD burden. The sum of these individual contributions equals equal the total observed change, ensuring mathematical consistency throughout the analysis.

This study was conducted using publicly available, de-identified data that contain no confidential or personally identifiable patient information. As a result, it was exempt from requirements for informed consent and formal ethical review.

## Results

### Trend in Global and Regional PD Prevalence, 1992–2021

Between 1992 and 2021, both the global crude prevalence and age-standardized prevalence of PD increased significantly. The estimated number of global PD cases rose by 238.64%, from 3,471,682.09 (95% UI: 31,94922.19–3,748,441.99) in 1992 to 11,756,618.58 (95% UI: 9,506,412.42–14,458,145.40) in 2021. An upward trend in crude PD prevalence was observed across all five SDI regions and across seven GBD super-regions.

**FIGURE 1:**
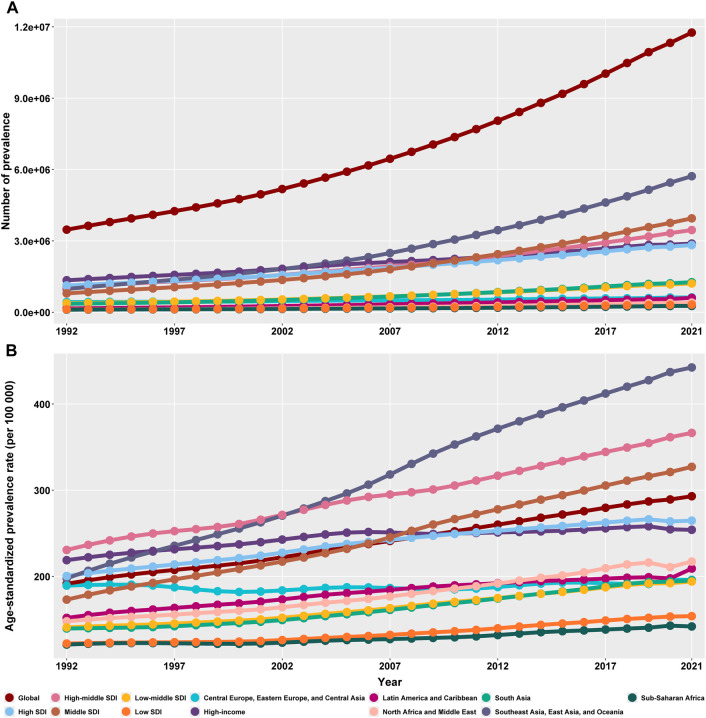
The prevalence numbers **(A)** and age-standardized prevalence rate **(B)** of Parkinson’s disease in global and the twelve regions from 1992 to 2021. SDI = sociodemographic Index. Data are from the Global Burden of Disease Study 2021, covering the period 1992–2021.

In 2021, the global age-standardized prevalence of PD was 292.93 (95% CI: 237.07–359.82) cases per 100,000 population, representing a 53.06% increase compared to 1992. Age-standardized prevalence generally increase across all five SDI regions. Among the seven super-regions, all except High-income and Sub-Saharan Africa showed an overall increasing trend. Notably, the Southeast Asia, East Asia, and Oceania super-region and the High-middle SDI region consistently exhibited age-standardized prevalence rates above the global average. Since 2008, the Middle SDI region has also remained above the global level. The most pronounced increase occurred in the Southeast Asia, East Asia and Oceania super-region, where prevalence rose by 123.84%, followed by the Middle SDI region with an increase of 89.07%. Further details are provided in Table 1 and Figure 1.

**TABLE 1:** Trends in Parkinson’s disease prevalence in global, five Socio-demographic Index regions, and seven super-regions, 1992–2021. Data are from the Global Burden of Disease Study 2021, covering the period 1992–2021.

Location	Population number (N, 95% UI)		Percentage of global level (%)		Prevalence number (N, 95% UI)		Percentage of global level (%)		Percentage change of prevalence, 1992–2021(%)	Age-standardised prevalence rate (per 100 000, 95% CI)		Percentage change of prevalence, 1992–2021(%)	APC model estimates Net drift of prevalence (% per year)
	1992	2021	1992	2021	1992	2021	1992	2021		1992	2021		
Global	2239783339.37 (2221211591.83, 2258355086.92)	4070010856.06 (4029309446.57, 4110712265.56)	100	100	3471682.09 (3194922.19, 3748441.99)	11756618.58 (9506412.42, 14458145.40)	100	100	328.64	191.38 (153.28, 236.46)	292.93 (237.07, 359.82)	53.06	1.42 (1.38, 1.46)
SDI regions													
High SDI	502435971.86 (497890887.51, 506981056.21)	724537509.13 (718454397.88, 730620620.37)	22.43	17.80	1122981.99 (1047050.87, 1198913.11)	2821780.91 (2456408.25, 3234332.48)	32.35	24.00	151.28	200.38 (165.07, 241.18)	264.60 (228.89, 305.11)	32.05	0.87 (0.83, 0.90)
High-middle SDI	524168069.18 (517175187.48, 531160950.88)	841007010.23 (829157449.81, 852856570.66)	23.40	20.66	1058626.81 (973508.01, 1143745.61)	3454145.68 (2741468.11, 4313995.85)	30.49	29.38	226.29	230.62 (186.19, 284.11)	366.42 (290.53, 457.91)	58.89	1.62 (1.54, 1.70)
Middle SDI	664808925.48 (656369158.46, 673248692.49)	1338306537.33 (1320861602.42, 1355751472.24)	29.68	32.88	792622.25 (715722.62, 869521.88)	3946464.33 (3075868.88, 4989739.89)	22.83	33.57	397.90	172.97 (132.38, 223.02)	327.05 (255.04, 412.92)	89.07	1.97 (1.91, 2.03)
Low-middle SDI	396039738.92 (390934114.29, 401145363.55)	820045058.52 (803927481.06, 836162635.99)	17.68	20.15	376932.77 (341588.78, 412276.77)	1207525.26 (935245.85, 1531804.95)	10.86	10.27	220.36	140.76 (107.41, 181.30)	193.91 (150.86, 244.72)	37.76	1.11 (1.09, 1.12)
Low SDI	150011436.32 (148484768.53, 151538104.10)	342759652.10 (336732022.38, 348787281.83)	6.70	8.42	116716.64 (105760.49, 127672.78)	319232.77 (243851.48, 407224.35)	3.36	2.72	173.51	122.01 (92.50, 156.61)	153.77 (118.66, 194.20)	26.03	0.76 (0.72, 0.79)
Super regions													
Central Europe, eastern Europe, and central Asia	224642571.86 (221797822.25, 227487321.47)	264477374.07 (259770924.11, 269183824.03)	10.03	6.50	425171.57 (388925.96, 461417.17)	620399.50 (506678.72, 749126.68)	12.25	5.28	45.92	189.22 (150.59, 235.47)	195.91 (159.68, 237.02)	3.54	0.04 (0.01, 0.07)
High-income	523014644.03 (518581928.64, 527447359.41)	718450991.41 (712710344.11, 724191638.71)	23.35	17.65	1344077.15 (1261660.66, 1426493.64)	2885655.30 (2513635.57, 3309996.09)	38.72	24.54	114.69	218.74 (182.14, 259.54)	253.95 (219.77, 293.22)	16.10	0.43 (0.38, 0.48)
Latin America and Caribbean	112292687.78 (111133459.00, 113451916.56)	306547919.10 (300697252.06, 312398586.14)	6.40	7.53	159689.00 (147036.09, 172341.91)	602220.86 (477519.61, 748623.81)	4.60	5.12	277.12	151.77 (118.47, 190.50)	208.99 (166.12, 259.20)	37.70	0.94 (0.90, 0.98)
North Africa and Middle East	143273771.06 (141477641.52, 145069900.60)	573787416.10 (568451634.34, 579123197.86)	5.01	14.10	105445.92 (96366.14, 114525.70)	403722.86 (310246.81, 509270.04)	3.04	3.43	282.87	147.81 (112.28, 187.21)	217.15 (168.30, 271.10)	46.91	1.29 (1.26, 1.32)
South Asia	385760794.06 (377055099.90, 394466488.22)	1663475782.47 (1620308716.25, 1706642848.69)	17.22	40.87	331590.33 (244427.70, 440342.02)	1261924.33 (951053.06, 1634138.89)	10.17	10.73	280.57	139.45 (103.25, 184.32)	195.12 (147.26, 252.04)	39.92	1.24 (1.20, 1.27)
Southeast Asia, east Asia, and Oceania	706936804.65 (692466346.53, 721407262.78)	1322020829.45 (1299509566.56, 1344532092.34)	31.56	32.48	977462.67 (877685.46, 1077239.88)	5717852.26 (4443610.34, 7287662.77)	28.16	48.64	484.97	197.63 (150.07, 256.65)	442.38 (343.64, 563.60)	123.84	2.51 (2.43, 2.59)
Sub-Saharan Africa	143862065.93 (142578108.13, 145146023.73)	339882142.75 (334613712.10, 345150573.40)	6.42	8.35	106719.15 (96922.53, 116515.78)	264843.47 (242175.36, 287511.58)	3.07	2.25	148.17	120.84 (92.00, 154.82)	141.83 (109.70, 179.39)	17.37	0.47 (0.44, 0.50)

Abbreviations: APC, Age - Period - Cohort, UI, uncertainty interval, CI, Confidence Interval.

In 2021, a total of 15 countries and regions reported 100,000 or more PD cases. The top five—India, China, the United States, Germany, and France—collectively accounted for 14.69% of global cases. Additionally, 26 countries had age-standardized prevalence rates exceeding the global average. Notably, Taiwan (Province of China), Canada, Israel, and China demonstrated rates more than 1.4 times the global average. Between 1992 and 2021, Norway experienced the largest increase in age-standardized prevalence (251.24%), followed by Taiwan (Province of China) with a rise of 189.92%. Additional data are available in Figure 1 and Supplementary Table 1.

### Temporal Trends in PD Prevalence in Different Age Groups

Table 1 and Supplementary Table 2 present the annual percentage changes in PD prevalence derived from the APC model analysis for all age groups globally and for each region. The global net drift in PD prevalence from 1992 to 2021 was 1.42% (95% CI: 1.38%–1.46%), indicating a consistent worldwide increase in disease prevalence. Local drift values were positive across all age groups (30–34 years to ≥95 years), reflecting an increase in PD prevalence in every age category. The rise in prevalence intensified with advancing age, peaking among individuals aged 60–64 years (1.61%, 95% CI: 1.55%–1.66%).

Among the five SDI regions, net drift values for PD prevalence in individuals aged 30–34 years to ≥95 years ranged from 0.76% (95% CI: 0.72%–0.79%) to 1.97% (95% CI: 1.91%–2.03%). Local drift values were positive across all SDI regions, emonstrating an age-related increase in PD prevalence in these groups. Within the seven GBD super-regions, net drift values for the same age range varied from 0.04% (95% CI: 0.01%–0.07%) to 2.51% (95% CI: 2.43%–2.59%). With the exception of Central Europe, Eastern Europe, and Central Asia, all other super-regions exhibited positive local drift values, indicating an age-related rise in PD prevalence in these areas. PD prevalence exceeded the global average across all age groups in the High-middle SDI, Middle SDI, North Africa and the Middle East, and Southeast Asia, East Asia, and Oceania super-regions.

**FIGURE 2:**
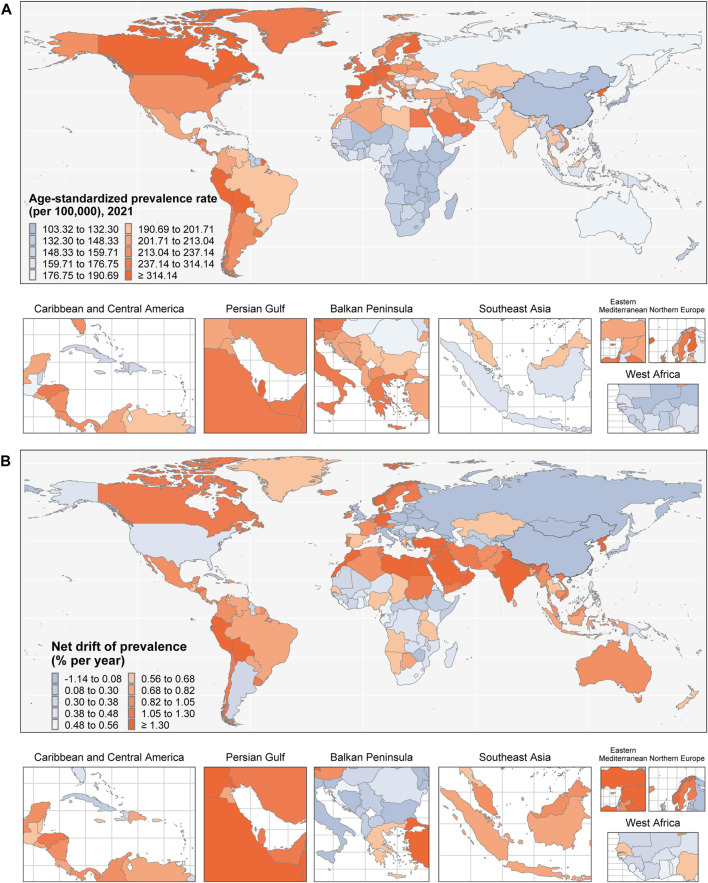
Map of **(A)** age-standardized prevalence rate in 2021 and **(B)** net drift of prevalence from 1992 to 2021 for prevalence in 204 countries and territories. Data are from the Global Burden of Disease Study 2021, covering the period 1992–2021.

**FIGURE 3:**
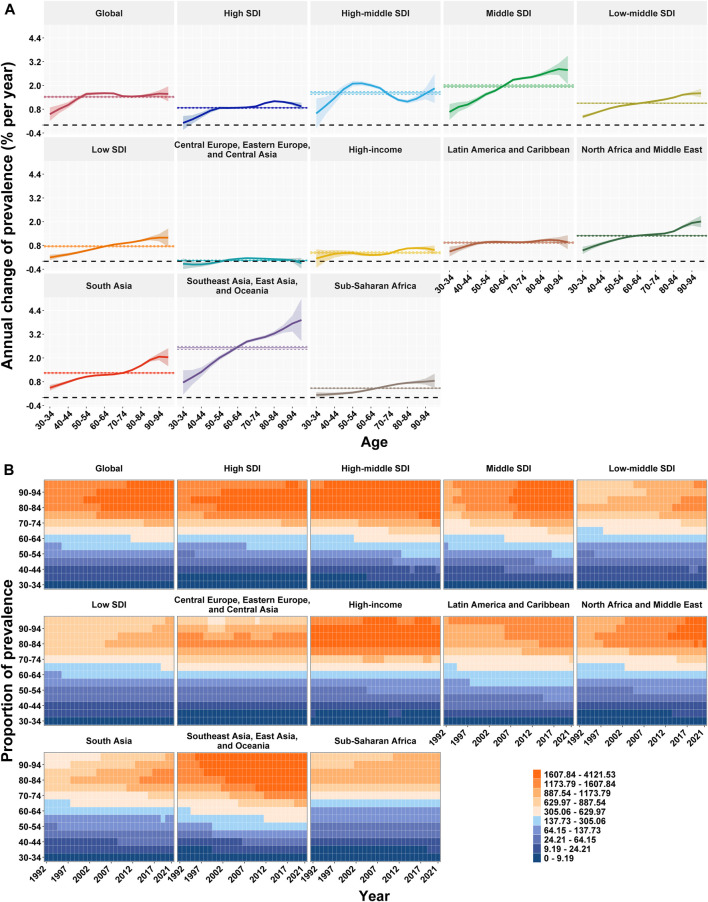
Local drift and age distribution of prevalence from 1992 to 2021 for Parkinson’s disease in global and the twelve regions. **(A)** Local drift of prevalence from 1992 to 2021 for Parkinson’s disease in fourteen age groups. **(B)** Temporal changes in age distribution of prevalence numbers in Parkinson’s disease from 1992 to 2021. The shaded areas denote the local drift (ie, annual percentage change of age-specific incidence, % per year) and their corresponding 95% CIs. SDI = sociodemographic index. Data are from the Global Burden of Disease Study 2021, covering the period 1992–2021.

Net drift and local drift values for PD prevalence across 204 countries are shown in Supplementary Tables 1, 3, respectively. Positive local drift values were observed in 170 countries, suggesting that PD prevalence generally increases with age in most settings. Temporal variations in prevalence across different age groups are illustrated in Figure 2 and Figure 3.

### Impact of Age, Period, and Birth Cohort on the Prevalence of PD

**FIGURE 4:**
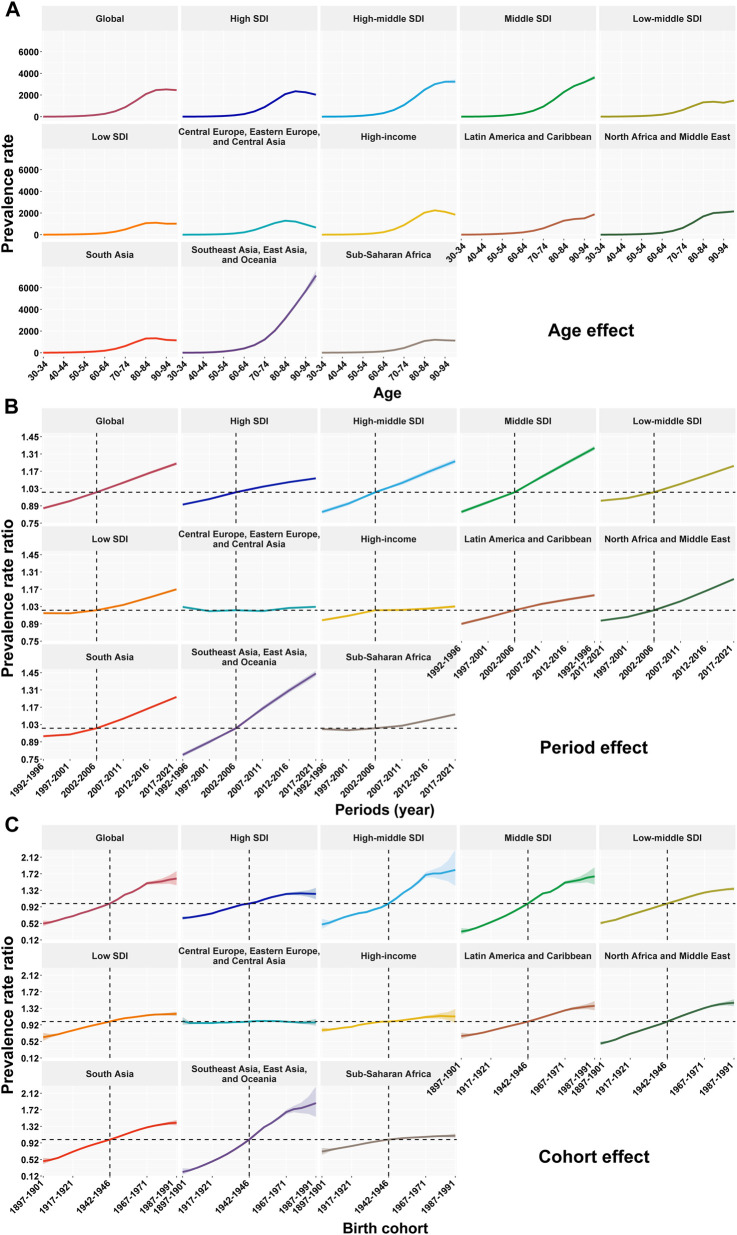
Age, period, and birth cohort effects on the prevalence of Parkinson’s disease at the global, socio-demographic index, and super-region levels (1990–2021). **(A)** The age effect is depicted through the longitudinal rates specific to age, which are adjusted for variations across different birth cohorts, taking into account the period-specific deviations. **(B)** Period effects are shown through the relative risk of Parkinson’s disease prevalence during different periods, calculated as the ratio of the age-specific rates from the period from 1992–1996 to 2017–2021, with the baseline period set as 2002–2006. **(C)** Birth cohort effects are demonstrated by the cohort relative risk of prevalence and calculated as the ratio of age-specific rates from 1897–1901 cohort to 1987–1991 cohort, with the reference cohort set at 1942–1946. The shaded areas denote the incidence rates or rate ratios and their corresponding 95% CIs. SDI = socio demographic index. Data are from the Global Burden of Disease Study 2021, covering the period 1992–2021.

The age, period, and birth cohort effects derived from the APC model analysis are presented in Figure 4 and Supplementary Tables 4–6. Regarding the age effect, prevalence was relatively low in younger age groups and rose sharply after age 60. Globally, PD prevalence increased with age until ≥95 years, after which it declined slightly. Across the five SDI regions, prevalence in the High SDI and Low SDI regions increased with age until approximately 90 years, then began to decline. The Low-middle SDI region exhibited a fluctuating upward trend, while the High-middle SDI and Middle SDI regions showed a consistent rise in prevalence with advancing age. Among the seven super-regions, Central Europe, Eastern Europe, and Central Asia displayed an upward trend until around 85 years, followed by a decline. The High-income, South Asia, and Sub-Saharan Africa regions followed patterns similar to the High SDI region. In all other super-regions, PD prevalence increased continuously with age, with the most pronounced rise observed among adults aged ≥60 years in the Southeast Asia, East Asia, and Oceania region. In terms of period effects, the RR of PD prevalence generally increased over time globally and across regions. The Middle SDI region and the Southeast Asia, East Asia, and Oceania super-region showed notably steeper increases compared with other regions. When the period 2002–2006 was set as the reference (RR = 1), the RR for PD in 2017–2021 ranged from 1.03 (95% CI: 1.02–1.03) to 1.44 (95% CI: 1.42–1.47)across regions. With regard to birth cohort effects, except for the High-middle SDI region and the High-income super-region, PD relative risk in the other four SDI region and six super-regions exhibited a generally stepwise increase starting from the 1987–1991 cohort. Compared with the reference cohort (1942–1946), individuals born in 1987–1991 had an RR ranging from 0.98 (95% CI: 0.90–1.07) to 1.88 (95% CI: 1.55–2.28).

### The Effect of Age, Period, and Birth Cohort on the Prevalence of PD in Typical Countries

To examine representative countries across different SDI levels, two nations were selected for each category (high, middle, and low) based on their net drift values: one with a value close to the median, representing the average trend, and the other with the highest net drift, indicative of a rapidly increasing trend (Supplementary Figure 3).

**FIGURE 5:**
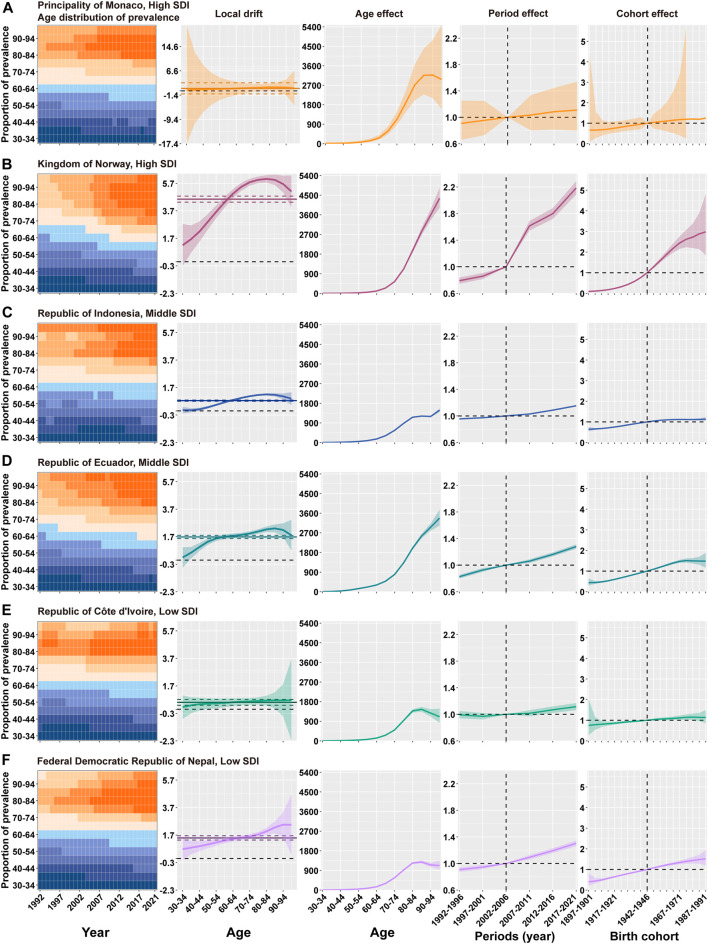
Age, period, and birth cohort effects on the prevalence of Parkinson’s disease in selected countries (Principality of Monaco, Kingdom of Norway, Republic of Indonesia, Republic of Ecuador, Republic of Côte d’Ivoire, and Federal Democratic Republic of Nepal, 1990–2021). **(A)** Principality of Monaco. From left to right: age distribution of prevalence (1990–2021, 30 -34 to 95+ years); local drift (annual percentage change, 1992–2021); age effects (LongAge); period effects (prevalence rate ratio, reference period 2002–2006); and birth cohort effects (prevalence rate ratio, reference cohort 1942–1946). **(B)** Kingdom of Norway. From left to right: age distribution of prevalence; local drift; age effects; period effects; and birth cohort effects. **(C)** Republic of Indonesia. From left to right: age distribution of prevalence; local drift; age effects; period effects; and birth cohort effects. **(D)** Republic of Ecuador. From left to right: age distribution of prevalence; local drift; age effects; period effects; and birth cohort effects. **(E)** Republic of Côte d’Ivoire. From left to right: age distribution of prevalence; local drift; age effects; period effects; and birth cohort effects. **(F)** Federal Democratic Republic of Nepal. From left to right: age distribution of prevalence; local drift; age effects; period effects; and birth cohort effects. The shaded areas denote the prevalence rates or rate ratios and their corresponding 95% CIs. SDI, socio demographic index. Data are from the Global Burden of Disease Study 2021, covering the period 1992–2021.

Figure 5 and Supplementary Tables 7–9 present the age, period, and cohort effect analyses of these representative countries. Among High-SDI countries, Monaco exhibited an unfavorable trend in disease burden. In terms of the age effect, the prevalence of PD increased with age and then declined after the age of 95. For the period effect, the risk of PD rose gradually, with the highest risk observed in the 2017–2021 period (RR = 1.11). Regarding the cohort effect, the risk of PD increased steadily, peaking in the 1987–1991 birth cohort (RR = 1.25). Norway, another High-SDI country, also showed an unfavorable trend in PD burden. For the age effect, the prevalence of PD increased continuously with age. Both the period and cohort effects in Norway indicated a sustained rise in PD risk: the period effect yielded the highest risk in the 2017–2021 interval (RR = 2.18), while the cohort effect reached its peak in the 1987–1991 birth cohort (RR = 2.98). Indonesia and Ecuador were selected as Middle-SDI countries. In terms of the age effect, the prevalence of PD increased with age in both countries, with a marked surge after the age of 65. For the period effect, the PD risk in both nations rose gradually, with the highest risk recorded in 2017–2021 (RR = 1.15 for Indonesia and RR = 1.28 for Ecuador). Regarding the cohort effect, Indonesia showed an overall upward trend in PD risk, peaking in the 1987–1991 birth cohort (RR = 1.13). In Ecuador, the PD risk increased in earlier birth cohorts, reaching its peak in the 1972–1976 cohort (RR = 1.51), and then declined in cohorts born after 1977–1981. Côte d'Ivoire and Nepal represented the Low-SDI category. For the age effect, the prevalence of PD increased with age in both countries and then decreased after the age of 90. In terms of the period effect, Côte d'Ivoire showed an initial decline followed by an increase in PD risk, whereas Nepal exhibited a continuous upward trend in risk. Both countries attained their highest risk levels in 2017–2021, with risk ratios of RR = 1.11 (Côte d'Ivoire) and RR = 1.30 (Nepal). Regarding the cohort effect, the PD risk in Côte d'Ivoire increased initially and then declined from the 1982–1986 birth cohort onward, peaking in the 1977–1981 cohort (RR = 1.15). In Nepal, the PD risk showed an overall upward trend, with the highest risk observed in the 1987–1991 birth cohort (RR = 1.52).

### Decomposition Analysis of Change in Prevalence

In this study, the relative contributions of population aging, population growth, and epidemiological changes to the global increase in PD prevalence from 1992 to 2021 were quantified as 16.54%, 48.36%, and 35.10%, respectively. These drivers varied substantially across regions and countries. Globally, population growth was the leading driver of rising PD burden. This pattern was particularly pronounced in Low-SDI regions, including North Africa and the Middle East (81.44%), and Sub-Saharan Africa (94.52%). In Central Europe, Eastern Europe, and Central Asia, population aging was the primary contributor, accounting for 47.64% of the observed increase. In contrast, epidemiological changes constituted the major driving factor in Southeast Asia, East Asia, and Oceania, responsible for 43.62% of the rise. Analysis of six representative countries across High-, Middle-, and Low-SDI levels further highlighted distinct regional patterns. Population growth significantly influenced the rising PD burden in Indonesia, Ecuador, Côte d'Ivoire and Nepal, with contributions of 65.38%, 50.92%, 81.92%, and 51.47%, respectively. Conversely, epidemiological changes were the primary driver in the Monaco and Norway, contributing 50.66% and 77.03%, respectively (Supplementary Tables 10; Supplementary Figure 4).

## Discussion

This study reveals that the global burden of PD increased steadily between 1992 and 2021. The rise in the number of affected individuals significantly outpaced population growth during this period, and age-standardized prevalence rates continued to climb indicating that the increase in cases was not solely attributable to population expansion. Most regions similarly exhibited a persistent upward trend in age-standardized prevalence. APC analysis further showed that age effects increased with advancing age across all regions, whereas period and birth-cohort effects demonstrated a continuous upward trend in nearly all areas. Decomposition analysis identified population growth as the primary demographic driver of increased case numbers, followed by population aging, with epidemiological changes also making substantial contributions in several High-middle-SDI regions. Together, these findings highlight a long-term rise in the burden of PD against the backdrop of global aging and rapidly changing population structures.

The increasing global burden of PD is driven not by a single factor but by the interplay of multiple mechanisms. Decomposition analysis indicates that population growth remains the primary contributor to case increases in Low- and Low-middle SDI regions. In contrast, the rising burden in High-middle- and High-SDI regions is influenced more by population aging and epidemiological shifts (such as heightened diagnostic sensitivity and improved disease recognition), reflecting structural disparities in risk exposure, diagnostic capacity, and healthcare across different stages of development. Beyond demographic factors, environmental exposures including organophosphate pesticides, heavy-metal contamination, and repeated head trauma, have been linked to elevated disease risk [20–22]. Concurrently, advances in imaging and biomarker technologies that enable earlier detection of milder cases may also contribute to rising standardized prevalence rates [23, 24]. It is noteworthy that East Asia may exhibit higher genetic susceptibility to PD. Previous studies indicate that mutations in genes such as LRRK2 and SNCA occur relatively frequencies in this region and are closely associated with increased PD risk [25, 26]. Combined with the more rapid growth trends observed in Southeast Asia, East Asia, and Oceania in this study, these genetic features may partly explain the higher disease burden in these regions. The persistent upward trajectory of PD across most regions underscores the need for healthcare systems worldwide to adopt more proactive and forward-looking strategies. In resource-limited settings, priorities should include reducing preventable environmental exposures and strengthening primary-level diagnostic capacity. In High-SDI regions, efforts should focus on enhancing early detection, implementing neuroprotective strategies, and establishing comprehensive long-term care systems.

Local drift results consistently demonstrate that PD prevalence rises steadily with age. This pattern is evident globally, across all SDI regions, and within all seven super-regions, underscoring the central role of aging as a key determinant of PD burden. In terms of age effects, PD prevalence increased markedly with age—particularly in those over 60 years—in the Middle-SDI, North Africa and Middle East, and Southeast Asia, East Asia, and Oceania. This aligns with previous studies identifying age as a major factor underlying the progressive rise in disease risk [27]. With aging, the accumulation of pathogenic factors can trigger neurodegenerative changes in the nervous system, including oxidative stress, mitochondrial dysfunction, and metabolic disturbances. Aging also elevates free-radical levels, exacerbating cellular damage, especially to nigrostriatal dopaminergic neurons [28]. Concurrently, impaired mitochondrial function leads to reduced cellular energy supply, compromising nervous-system integrity and accelerating neurodegenerative processes [29]. Moreover, older individuals are more likely to have chronic conditions such as hypertension and diabetes, which can worsen neurodegeneration and increase PD risk through vascular injury and metabolic abnormalities. This study also observed that PD prevalence increased initially and then declined with age in some regions, particularly among those over 85. Several factors may explain this pattern: first, PD is often diagnosed later in life, and the its clinical signs may be overlooked in older adult; second, older adults face a higher prevalence of competing fatal comorbidities such as heart disease and cance, whose associated mortality may influence PD statistics [30].

Period effects indicate that the relative risk of PD prevalence has steadily increased over the past 3 decades, with a more pronounced rise observed in High-middle and High SDI regions. Advances in socioeconomic development and medical technology have substantially enhanced imaging capabilities, neurobiological markers, and primary care diagnostic capacity [31]. This progress has enabled the identification and inclusion of more early-stage and mild-symptom cases within surveillance systems, thereby elevating reported prevalence rates. Concurrently, extended life expectancy has increased patient survival, contributing to the temporal accumulation of cases. Additionally, heightened environmental exposures resulting from rapid industrialization may significantly influence these period trends. In recent decades, broader exposure to pesticides, heavy metals, and air pollution in many countries has been associated with elevated PD risk [32–34]. Lifestyle shifts may also alter population risk structures; for example, while declining smoking rates are beneficial overall, their inverse association with PD could impact disease burden in certain regions [35]. Overall, period effects reflect the combined influence of cumulative environmental exposures, lifestyle changes, medical advances, and prolonged patient survival. As many of these factors are amenable to intervention, strengthening environmental governance, reducing harmful exposures, improving primary-level diagnostic capacity, and enhancing long-term care systems could provide substantial public health value in mitigating the sustained rise in PD burden.

Cohort effects revealed a significantly increased PD risk among later-born populations, consistent with findings from other studies [12]. Unhealthy dietary habits, physical inactivity, excessive use of electronic devices, and poor sleep quality may affect brain function through various pathways and elevate disease risk [11, 36, 37]. Furthermore, long-term exposure to high-pressure, fast-paced lifestyle increases physical and psychological burdens, raising the likelihood of immune dysfunction, chronic inflammation, and metabolic disorders. The cumulative effect of these adverse factors on the nervous system may damage dopaminergic neurons and promote the onset and progression of PD [38–40]. With the global rise in metabolic diseases such as hypertension and diabetes, these chronic conditions have become key background factors for PD. Numerous studies report that hypertension and diabetes can accelerate neurodegeneration by inducing oxidative stress, exacerbating neuroinflammation, and damaging the blood-brain barrier, thereby increasing PD risk [41, 42]. The widespread prevalence of metabolic disorders, combined with complex modern environmental influences, represents another major driver of increasing PD prevalence. Therefore, individuals born in more recent years often face multiple health challenges, including social pressures, environmental pollution, and lifestyle shifts. The convergence of these factors may significantly contribute to the rising PD incidence in this population.

Several limitations should be noted in this study. First, it is based on secondary analysis of modeled estimates from GBD 2021, which depend on the completeness of raw data and regional diagnostic coverage. In Low- and Low-middle SDI countries with weaker surveillance systems, data scarcity often leads to greater reliance on model inference. This may result in underestimation (e.g., missed or misdiagnosed cases) or overestimation due to extrapolation uncertainty. The direction and magnitude of such biases depend on regional data quality, diagnostic capacity, and reporting consistency. Second, as the study relies on cross-sectional data, it cannot establish causal relationships or evaluate the relative contributions of environmental, genetic, and behavioral risk factors. Finally, the inherent time lag in GBD data may lead to underestimation of the current disease burden, particularly in regions undergoing rapid population aging.

### Conclusion

In conclusion, both the global number of PD patients and age-standardized prevalence rates increased steadily between 1992 and 2021, with the most significant growth observed among individuals aged 60 and above. As many regions enter phases of deep aging, the burden of PD is projected to rise further. To align with the Global Agenda for Neurohealth 2030, countries should develop actionable, integrated strategies tailored to their epidemiological profiles and health resource contexts. Recommended measures include expanding population surveillance and early detection capacity, strengthening the role of primary care in managing chronic neurological diseases, improving access to long-term care services, and advancing systematic efforts in environmental risk control, health promotion, and multidisciplinary care models. Implementing such strategies will enhance healthcare systems’ capacity to address PD and more effectively mitigate its projected future burden.

## Data Availability

The data used in this study can be derived from the GBD 2021 (Available at: https://ghdx.healthdata.org/gbd-2021).
