# Supplementary material for: The Burden of Parkinson’s Disease Based on the GBD 2021
Source: Int J Public Health. 2026 Feb 24;71:1608863. doi: 10.3389/ijph.2026.1608863 (PMC12971533; doi:10.3389/ijph.2026.1608863)
Supplement: Supplementary file 1 [file Supplementaryfile2.doc]

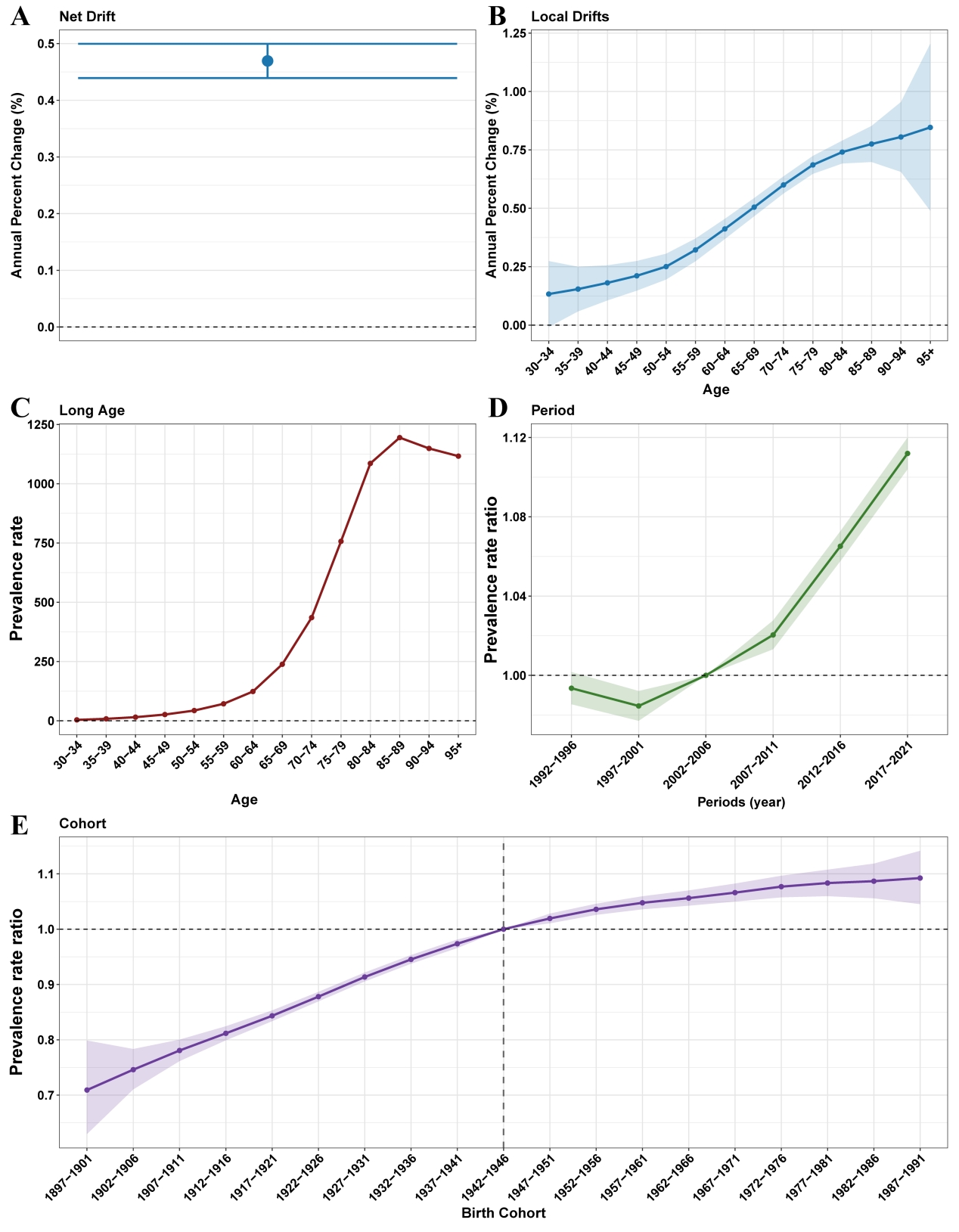


Supplementary Figure S1: Diagnostic plots of the Age–Period–Cohort model for Parkinson’s disease, 1992–2021. (A) Net drift showing the overall annual percentage change in age-adjusted incidence rates. (B) Local drifts illustrating age-specific annual percentage changes. (C) Period rate ratios relative to the reference period (2002 to 2006). (D) Cohort rate ratios relative to the reference birth cohort (1942 to 1946). (E) Age effect (LongAge), showing the adjusted prevalence differences across age groups after controlling for cohort effects. Shaded ribbons indicate 95% confidence intervals. Data are from the Global Burden of Disease Study 2021, covering the period 1992–2021.


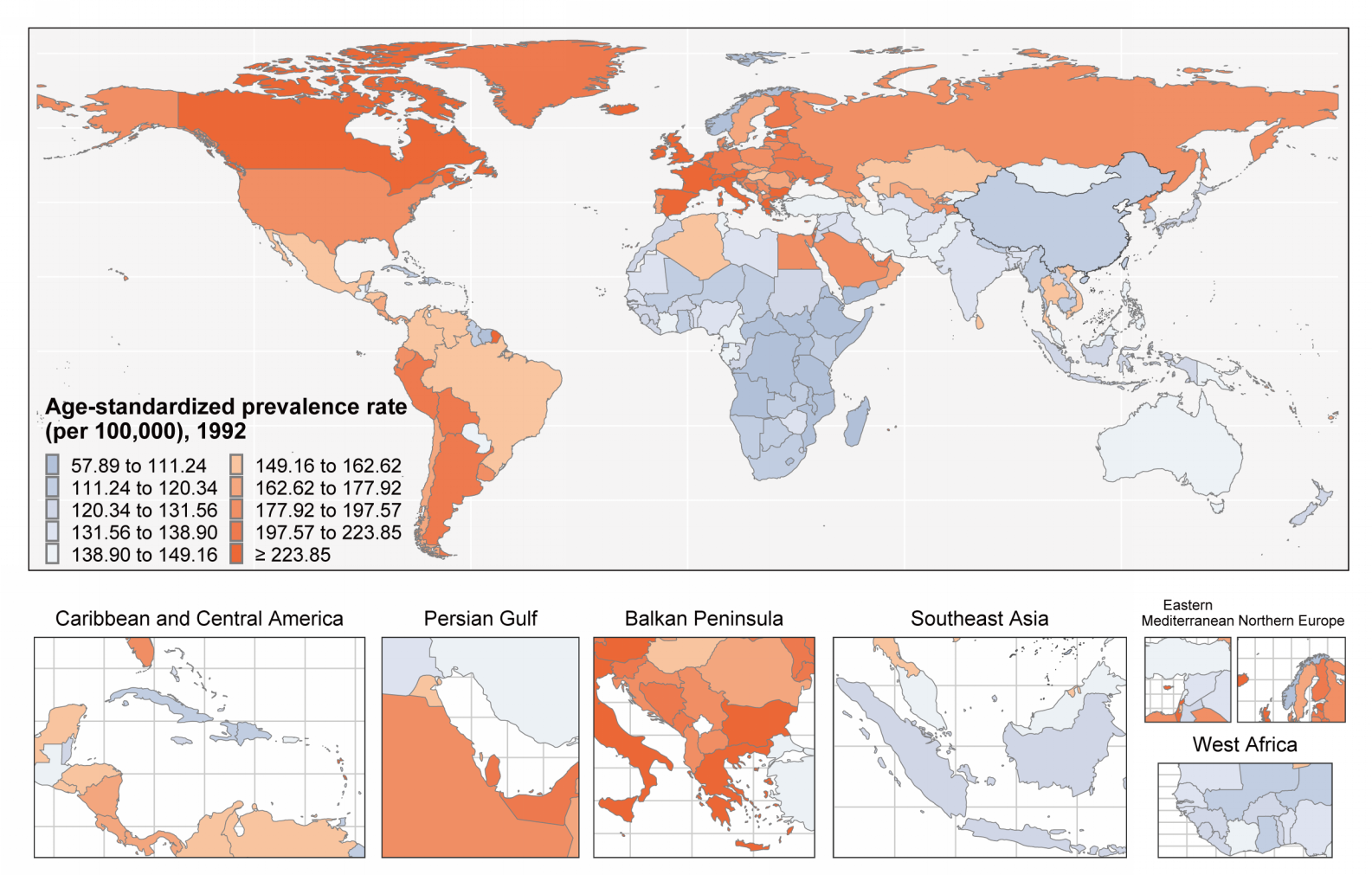


Supplementary Figure S2: Map of age-standardized prevalence rate in 1992 for prevalence in 204 countries and territories. Data are from the Global Burden of Disease Study 2021 (1992 data).
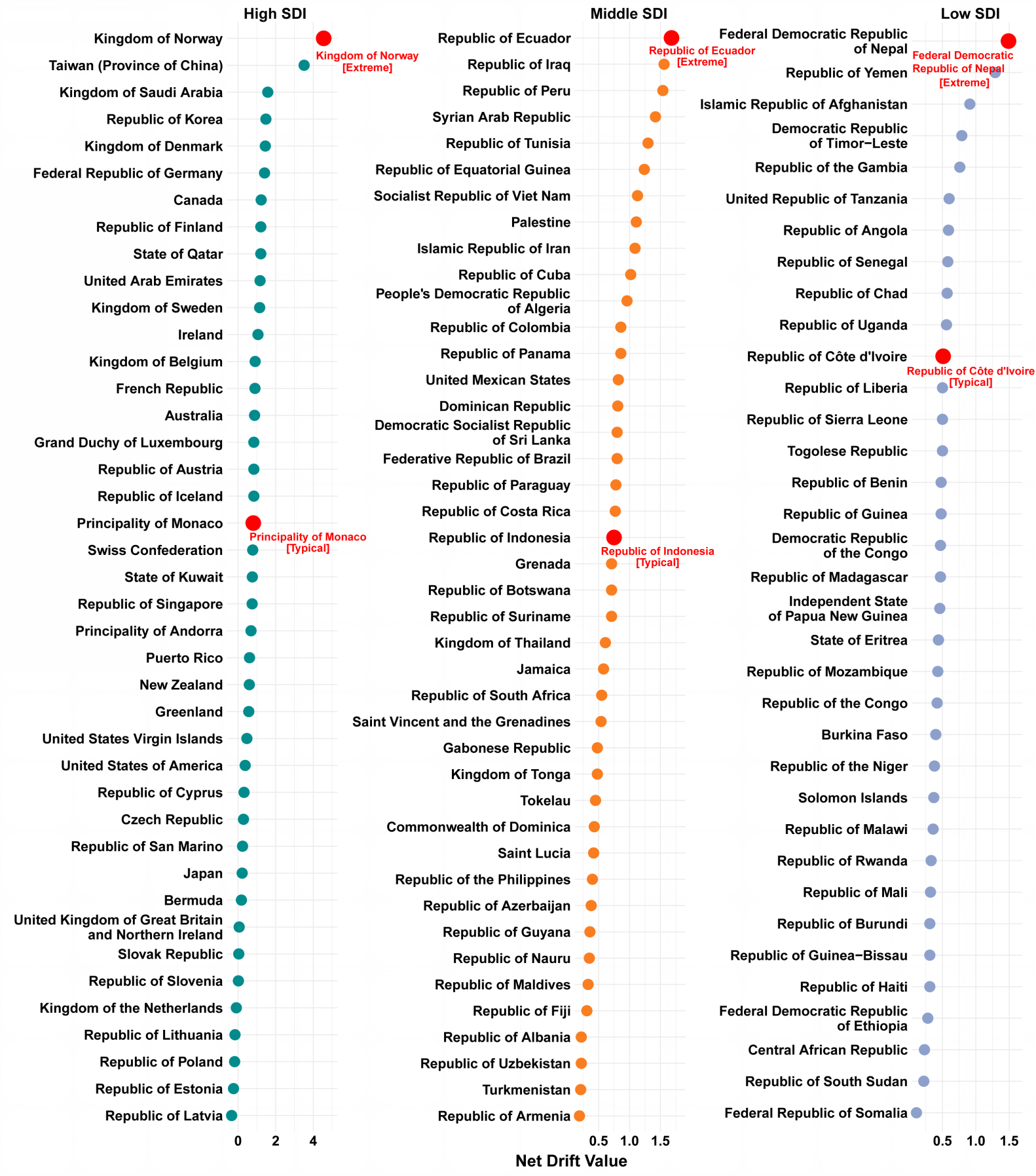


 Supplementary Figure S3: Two countries were selected from each Socio-Demographic Index category (high, middle, and low) based on their net drift values. Data are from the Global Burden of Disease S”. Please update accordingly.


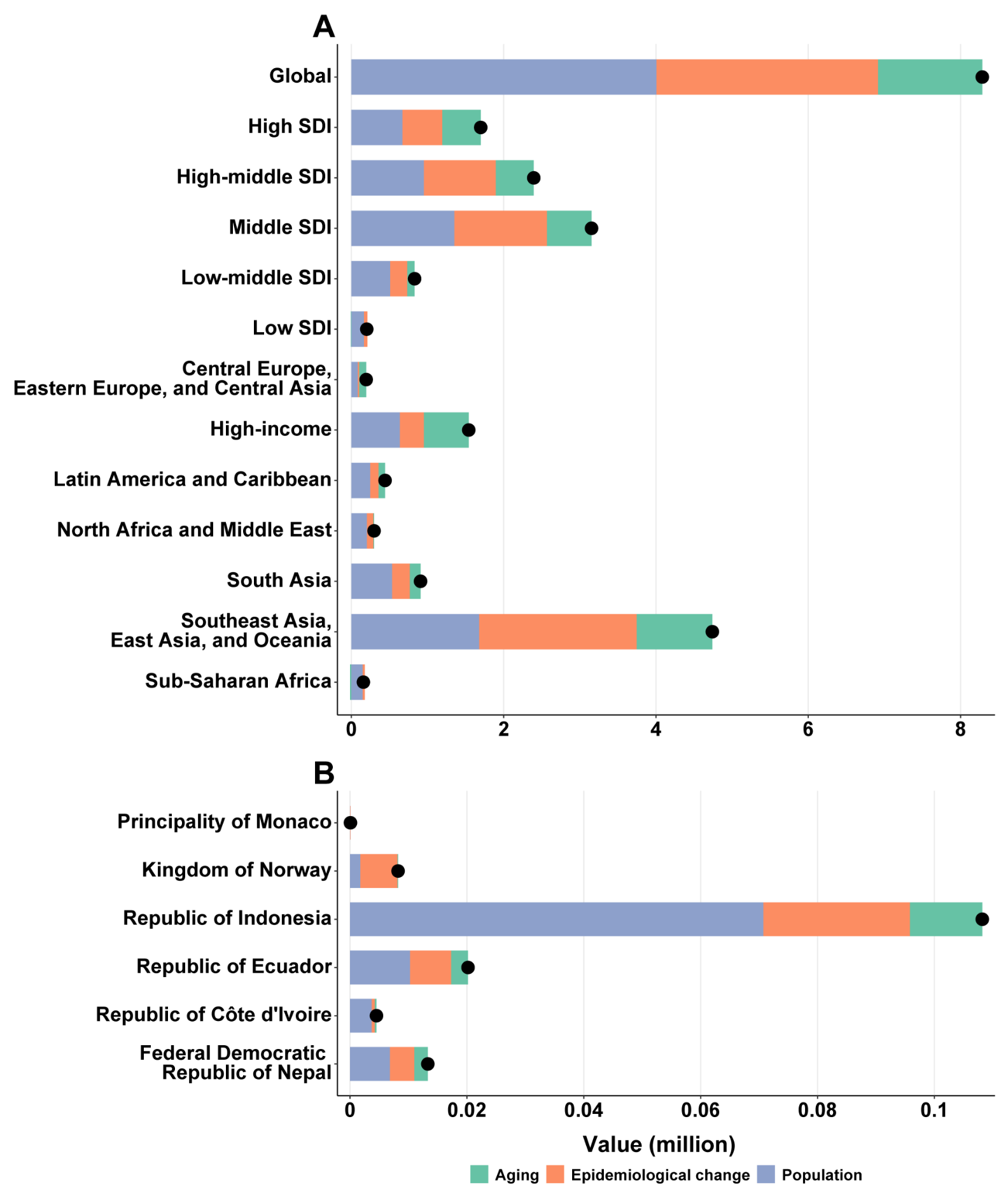


Supplementary Figure S4 Decomposition Analysis of Parkinson’s Disease Prevalence from 1992 to 2021. (A) Prevalence Change Decomposition: Global, SDI Regions, and Seven Super-Regions, (B) Decomposition analysis of prevalence changes in six representative countries. SDI, sociodemographic index.
