# Supplementary material for: The Burden of Parkinson’s Disease Based on the GBD 2021
Source: Int J Public Health. 2026 Feb 24;71:1608863. doi: 10.3389/ijph.2026.1608863 (PMC12971533; doi:10.3389/ijph.2026.1608863)
Supplement: Supplementary file 2 [file Supplementaryfile1.docx]

# ****Supplementary Methods – Age–Period–Cohort (APC) Analysis****

## ****Model Specification****

We employed the canonical **Poisson log-linear age–period–cohort (APC) model**, consistent with the methodological framework implemented in the **National Cancer Institute (NCI) APC Web Tool**. For each age group a, period p, and cohort c, the observed counts (Y_{ap}) were assumed to follow a Poisson distribution:

$$\text{Y}_{\text{ap}}\text{∼}\text{Poisson}\text{(}\text{μ}_{\text{ap}}\text{)}$$

$$\log\text{(}\text{μ}_{\text{ap}}\text{)=}\log\text{(}\text{P}_{\text{ap}}\text{)+}\text{α}_{\text{a}}\text{+}\text{π}_{\text{p}}\text{+}\text{γ}_{\text{c}}$$

In this equation, $\text{P}_{\text{ap}}$ is the population at risk (used as an offset), $\text{α}_{\text{a}}$ represents age effects, $\text{π}_{\text{p}}$ represents period effects, $\text{γ}_{\text{c}}$ represents cohort effects.

Age, period, and cohort categories were constructed in **5-year intervals**, with ages 30 to 95+years and calendar years 1992 to 2021, following GBD classifications.

## ****Identifiability and Holford Constraint****

Because there exists a linear dependence between age, period, and cohort:

$$\text{Co}\text{h}\text{ort}\text{=}\text{Period}\text{-}\text{Age}$$

the three effects cannot be uniquely estimated without additional constraints (“identifiability problem”). We addressed this using the **Holford constraint**, which is the default identifiability solution implemented in the NCI APC Web Tool and embedded in the apc2fit() function. The Holford method decomposes each effect into:

$$\text{Effect}\text{=}\text{Drift}\text{ }\text{trend}\text{+}\text{Deviation}$$

Ensuring the three sets of deviations sum to zero, linear trends are shared across effects, the resulting components are interpretable and comparable.

## ****Net Drift and Local Drift****

### ****Net Drift****

Net Drift quantifies the **overall annual percentage change** in age-adjusted rates across the entire study period, it reflects the period-cohort combined trend:

$$\text{Net}\text{ }\text{Drift}\text{=}\frac{\text{d}}{\text{dt}}\log\text{(}\text{R}\text{(}\text{t}\text{))×100\%}$$

### ****Local Drift****

Local Drift represents the **age-specific annual percentage change**:

$${\text{Local}\text{ }\text{Drift}}_{\text{a}}\text{=}\frac{\text{d}}{\text{dt}}\log\text{(}\text{R}_{\text{a}}\text{(}\text{t}\text{))×100\%}$$

These quantities are automatically derived by the NCI APC algorithm used in our code.

## ****Age, Period, and Cohort Deviations****

Deviations quantify **nonlinear departures** from the overall drift.

For effect $\text{X}\text{∈\{}\text{α}\text{,}\text{π}\text{,}\text{γ}$}:

$$\text{X}\text{=}\text{Drift}\text{×}\text{t}\text{+}\text{Deviation}$$

With the deviation components constrained to sum to zero.The deviations describe: Age-specific curvature (age deviation), Period-specific shocks (period deviation), Cohort-specific generational patterns (cohort deviation).

## ****Software and Implementation Details****

All analyses were performed in:

1. **R 4.3.1,**
2. Using the R scripts released by the **NCI APC Web Tool,**
3. Key functions: prepare_rates(), apc2fit().

My code implementation: Age interval = **5 years,** Period interval = **5 years,** StartAge = **30,** StartYear = **1992,** Populations included as offsets: population counts for each age–sex–year group.

These settings correspond exactly to the default settings in the official NCI APC framework.

## ****Model Diagnostics****

We conducted standard APC diagnostics following NCI recommendations:

1. **Residual analysis:** Checking systematic deviation across age, period, and cohort groups.
2. **Goodness of fit:** Deviance residuals, Assessment of overdispersion.
3. **Internal consistency:** Comparison of drift and deviations for expected curvature.

These diagnostics verified that the model adequately fitted the data.
