# Supplementary material for: The Burden of Parkinson’s Disease Based on the GBD 2021
Source: Int J Public Health. 2026 Feb 24;71:1608863. doi: 10.3389/ijph.2026.1608863 (PMC12971533; doi:10.3389/ijph.2026.1608863)
Supplement: Supplementary file 3 [file Supplementaryfile3.doc]

**Supplementary Table S1: Parkinson’s disease prevalence numbers and age-standardized prevalence rate from 1992 to 2021 in countries and territories**

| **Location** | **1992** | **1992** | **2021** | **2021** | **1992 - 2021 APC** |
| --- | --- | --- | --- | --- | --- |
|  | **Prevalence Number** | **Prevalence ASR** | **Prevalence Number** | **Prevalence ASR** | **Net Drift (%/year)** |
| American Samoa | 13.27 (11.98, 14.56) | 144.14 (107.25, 185.82) | 30.94 (28.05, 33.82) | 519.62 (398.51, 669.35) | 0.36 (-2.47, 3.27) |
| Antigua and Barbuda | 36.53 (33.31, 39.74) | 138.35 (105.24, 179.46) | 90.24 (80.39, 100.09) | 341.07 (251.42, 444.46) | 0.65 (-0.87, 2.20) |
| Arab Republic of Egypt | 19520.81 (17481.87, 21559.75) | 187.84 (141.28, 246.00) | 66165.45 (59936.22, 72394.67) | 411.66 (375.96, 450.60) | 1.38 (1.31, 1.46) |
| Argentine Republic | 31376.96 (29573.98, 33179.93) | 202.17 (168.94, 237.71) | 60022.13 (53967.35, 66076.91) | 155.94 (117.22, 202.14) | 0.32 (0.23, 0.41) |
| Australia | 14872.54 (13591.90, 16153.19) | 146.50 (112.54, 181.30) | 43847.67 (39284.70, 48410.63) | 167.62 (124.05, 220.73) | 0.88 (0.75, 1.01) |
| Barbados | 197.79 (181.13, 214.46) | 134.46 (101.85, 171.20) | 417.51 (380.09, 454.94) | 156.62 (115.31, 204.26) | 0.54 (-0.21, 1.29) |
| Belize | 58.47 (53.34, 63.60) | 127.56 (94.17, 165.34) | 214.81 (195.82, 233.80) | 194.75 (144.43, 262.21) | 0.64 (-0.39, 1.68) |
| Bermuda | 50.76 (45.91, 55.61) | 171.34 (128.70, 224.33) | 135.64 (124.97, 146.30) | 198.24 (149.07, 258.94) | 0.18 (-1.25, 1.63) |
| Bolivarian Republic of Venezuela | 6979.11 (6416.89, 7541.33) | 149.21 (112.84, 188.47) | 26903.43 (24675.80, 29131.06) | 159.33 (120.14, 207.25) | 0.81 (0.71, 0.90) |
| Bosnia and Herzegovina | 3729.86 (3399.87, 4059.85) | 206.86 (156.52, 259.60) | 6336.84 (5505.65, 7168.03) | 157.57 (116.95, 206.21) | -0.14 (-0.41, 0.13) |
| Brunei Darussalam | 72.01 (65.05, 78.98) | 160.45 (120.54, 207.27) | 289.37 (258.26, 320.49) | 196.62 (141.83, 264.27) | 0.69 (-1.18, 2.59) |
| Burkina Faso | 2334.73 (2057.69, 2611.77) | 131.11 (94.55, 176.00) | 5361.59 (4813.55, 5909.63) | 191.50 (147.81, 244.49) | 0.40 (0.18, 0.63) |
| Canada | 46688.75 (45006.95, 48370.54) | 281.79 (250.41, 314.31) | 151300.01 (146007.63, 156592.40) | 156.57 (114.10, 207.99) | 1.23 (1.17, 1.29) |
| Central African Republic | 515.66 (459.60, 571.72) | 110.92 (79.81, 146.19) | 1013.20 (893.31, 1133.09) | 217.91 (164.05, 287.98) | 0.23 (-0.38, 0.84) |
| Commonwealth of Dominica | 36.91 (33.47, 40.34) | 130.09 (97.54, 169.28) | 61.41 (55.68, 67.13) | 188.62 (134.84, 254.11) | 0.43 (-1.27, 2.15) |
| Commonwealth of the Bahamas | 102.98 (94.19, 111.77) | 137.13 (103.26, 175.62) | 309.15 (282.43, 335.86) | 180.36 (133.72, 243.14) | 0.37 (-0.45, 1.20) |
| Cook Islands | 10.61 (9.48, 11.74) | 196.59 (146.89, 257.93) | 27.15 (23.93, 30.37) | 208.08 (153.76, 280.42) | 0.29 (-3.31, 4.03) |
| Czech Republic | 11890.22 (11034.65, 12745.79) | 175.57 (140.83, 213.04) | 22320.07 (20325.54, 24314.60) | 190.86 (138.85, 251.63) | 0.29 (0.14, 0.45) |
| Democratic People's Republic of Korea | 12851.20 (11524.28, 14178.12) | 182.41 (135.80, 239.66) | 50708.70 (45575.17, 55842.22) | 163.79 (117.45, 216.51) | 2.14 (2.05, 2.23) |
| Democratic Republic of Sao Tome and Principe | 39.64 (34.97, 44.30) | 142.06 (104.44, 191.34) | 78.90 (70.30, 87.51) | 201.86 (149.97, 260.81) | 0.89 (-0.75, 2.54) |
| Democratic Republic of the Congo | 7015.79 (6053.28, 7978.31) | 108.63 (78.24, 150.45) | 17940.12 (15762.36, 20117.88) | 182.33 (133.64, 244.33) | 0.47 (0.33, 0.61) |
| Democratic Republic of Timor-Leste | 137.02 (121.88, 152.17) | 121.94 (89.99, 161.34) | 589.97 (521.25, 658.69) | 183.41 (137.82, 239.89) | 0.79 (0.01, 1.57) |
| Democratic Socialist Republic of Sri Lanka | 7232.76 (6571.05, 7894.47) | 152.40 (112.19, 196.47) | 24366.16 (21401.96, 27330.36) | 192.19 (142.30, 255.42) | 0.80 (0.68, 0.92) |
| Dominican Republic | 2053.19 (1846.48, 2259.90) | 113.15 (83.30, 150.61) | 7224.32 (6548.31, 7900.32) | 164.75 (125.05, 209.44) | 0.81 (0.62, 0.99) |
| Eastern Republic of Uruguay | 3582.43 (3336.37, 3828.49) | 181.12 (147.49, 218.82) | 6978.15 (6327.38, 7628.91) | 188.63 (138.44, 244.49) | 1.10 (0.83, 1.37) |
| Federal Democratic Republic of Ethiopia | 9161.56 (8119.19, 10203.94) | 108.66 (78.44, 145.82) | 23461.94 (21098.18, 25825.71) | 156.35 (124.18, 197.27) | 0.28 (0.13, 0.44) |
| Federal Democratic Republic of Nepal | 4573.00 (4032.58, 5113.42) | 113.16 (82.16, 153.38) | 17891.68 (15925.28, 19858.09) | 197.16 (151.90, 248.95) | 1.49 (1.34, 1.65) |
| Federal Republic of Germany | 142754.82 (137734.09, 147775.55) | 223.46 (197.04, 251.92) | 421741.44 (410254.00, 433228.89) | 146.72 (111.74, 190.68) | 1.41 (1.32, 1.50) |
| Federal Republic of Nigeria | 25070.16 (22392.22, 27748.10) | 134.73 (98.24, 177.59) | 57522.65 (51790.92, 63254.38) | 142.49 (105.70, 189.27) | 0.66 (0.59, 0.72) |
| Federal Republic of Somalia | 940.79 (831.71, 1049.86) | 97.97 (69.59, 132.33) | 2464.45 (2154.91, 2773.99) | 217.35 (158.67, 289.69) | 0.11 (-0.28, 0.50) |
| Federated States of Micronesia | 34.30 (30.64, 37.95) | 166.82 (123.96, 220.08) | 54.16 (48.05, 60.26) | 140.73 (106.96, 175.83) | 0.60 (-1.45, 2.69) |
| Federative Republic of Brazil | 62410.78 (56126.12, 68695.45) | 149.77 (110.28, 197.37) | 223604.71 (202669.63, 244539.78) | 191.73 (147.96, 238.95) | 0.80 (0.73, 0.86) |
| French Republic | 101766.94 (95680.40, 107853.48) | 234.80 (192.36, 280.65) | 238751.82 (215617.01, 261886.64) | 208.97 (151.05, 268.95) | 0.90 (0.82, 0.98) |
| Gabonese Republic | 360.55 (320.54, 400.55) | 141.45 (103.15, 188.58) | 709.43 (629.91, 788.94) | 204.81 (148.68, 274.08) | 0.48 (-0.09, 1.06) |
| Georgia | 5090.63 (4616.41, 5564.86) | 174.17 (135.79, 221.11) | 4581.65 (4179.10, 4984.19) | 200.95 (156.42, 253.81) | -0.37 (-0.61, -0.14) |
| Grand Duchy of Luxembourg | 692.78 (628.12, 757.44) | 251.76 (189.17, 317.11) | 1745.11 (1579.47, 1910.75) | 210.13 (156.86, 266.49) | 0.84 (0.34, 1.33) |
| Greenland | 25.88 (22.98, 28.78) | 199.15 (148.48, 261.20) | 69.49 (62.41, 76.56) | 199.95 (157.04, 252.36) | 0.57 (-2.25, 3.47) |
| Grenada | 41.31 (37.10, 45.51) | 117.64 (86.21, 156.96) | 81.35 (73.03, 89.67) | 179.34 (142.85, 222.02) | 0.71 (-0.75, 2.19) |
| Guam | 64.21 (56.37, 72.05) | 208.15 (150.94, 282.70) | 210.43 (186.60, 234.25) | 218.77 (169.79, 280.53) | 0.10 (-1.21, 1.43) |
| Hashemite Kingdom of Jordan | 676.27 (619.71, 732.82) | 125.49 (96.35, 158.13) | 4589.01 (4203.53, 4974.49) | 213.05 (159.47, 272.84) | 0.95 (0.64, 1.27) |
| Hellenic Republic | 18565.96 (16814.30, 20317.62) | 243.16 (191.15, 308.52) | 38004.92 (34144.87, 41864.96) | 218.28 (183.95, 257.65) | 0.57 (0.46, 0.68) |
| Hungary | 11686.91 (10857.82, 12515.99) | 160.89 (127.75, 195.14) | 18010.63 (16626.80, 19394.46) | 185.56 (145.73, 233.58) | 0.36 (0.19, 0.53) |
| Independent State of Papua New Guinea | 1021.92 (904.30, 1139.55) | 146.86 (106.93, 196.27) | 3072.30 (2752.67, 3391.93) | 199.07 (142.97, 249.52) | 0.46 (0.11, 0.82) |
| Independent State of Samoa | 63.54 (56.82, 70.26) | 175.90 (132.02, 229.82) | 122.06 (109.83, 134.30) | 180.16 (137.96, 233.21) | 0.53 (-0.87, 1.95) |
| Ireland | 4814.04 (4349.75, 5278.34) | 234.47 (185.05, 299.35) | 12807.87 (11721.15, 13894.58) | 213.71 (162.03, 262.85) | 1.06 (0.88, 1.24) |
| Islamic Republic of Afghanistan | 3879.25 (3422.06, 4336.44) | 131.90 (96.35, 176.68) | 6651.03 (6013.51, 7288.55) | 220.29 (172.92, 276.56) | 0.91 (0.71, 1.11) |
| Islamic Republic of Iran | 15771.27 (14010.60, 17531.94) | 149.11 (109.86, 196.71) | 72153.18 (65226.11, 79080.25) | 216.83 (168.87, 270.40) | 1.09 (1.02, 1.16) |
| Islamic Republic of Mauritania | 572.82 (512.44, 633.20) | 132.82 (97.81, 173.41) | 1363.34 (1197.66, 1529.02) | 197.10 (154.15, 252.40) | 0.35 (-0.09, 0.80) |
| Islamic Republic of Pakistan | 37821.06 (33586.98, 42055.15) | 146.02 (107.02, 194.88) | 93572.31 (83946.90, 103197.71) | 194.11 (146.62, 242.14) | 0.88 (0.82, 0.93) |
| Jamaica | 1112.33 (1018.28, 1206.39) | 124.91 (94.74, 160.55) | 2285.65 (2050.22, 2521.08) | 166.56 (125.44, 216.90) | 0.58 (0.30, 0.85) |
| Japan | 111547.90 (99692.15, 123403.64) | 129.32 (97.03, 169.24) | 203724.36 (181008.40, 226440.33) | 190.52 (143.22, 245.92) | 0.22 (0.12, 0.32) |
| Kingdom of Bahrain | 114.37 (102.87, 125.87) | 169.01 (125.18, 218.91) | 754.39 (666.90, 841.87) | 202.05 (150.98, 260.50) | 1.12 (-0.32, 2.59) |
| Kingdom of Belgium | 17442.39 (16159.86, 18724.92) | 222.90 (178.22, 271.94) | 36399.16 (33600.53, 39197.80) | 204.59 (154.63, 270.60) | 0.91 (0.80, 1.02) |
| Kingdom of Bhutan | 138.50 (123.77, 153.23) | 132.56 (95.18, 173.82) | 540.46 (481.44, 599.48) | 111.65 (83.17, 146.68) | 1.31 (0.42, 2.21) |
| Kingdom of Cambodia | 2311.87 (2054.69, 2569.05) | 118.85 (89.01, 157.56) | 8064.20 (7257.26, 8871.14) | 187.37 (142.10, 239.02) | 0.85 (0.63, 1.07) |
| Kingdom of Denmark | 7532.83 (6975.76, 8089.91) | 181.17 (137.11, 224.11) | 17635.50 (15669.11, 19601.90) | 171.91 (126.89, 224.18) | 1.45 (1.30, 1.61) |
| Kingdom of Eswatini | 148.00 (130.56, 165.45) | 122.78 (89.58, 165.55) | 312.04 (276.40, 347.68) | 190.09 (147.16, 244.43) | 0.49 (-0.38, 1.37) |
| Kingdom of Lesotho | 388.69 (349.27, 428.11) | 102.82 (74.13, 135.18) | 543.39 (483.28, 603.51) | 145.44 (106.76, 191.05) | 0.53 (-0.01, 1.07) |
| Kingdom of Morocco | 8531.82 (7689.68, 9373.96) | 130.62 (94.86, 171.07) | 30311.66 (27145.54, 33477.77) | 296.67 (228.52, 381.45) | 1.67 (1.57, 1.77) |
| Kingdom of Norway | 1957.49 (1726.57, 2188.41) | 57.89 (41.72, 77.73) | 10182.26 (9135.59, 11228.93) | 309.86 (219.41, 400.31) | 4.55 (4.33, 4.77) |
| Kingdom of Saudi Arabia | 4223.25 (3794.73, 4651.78) | 177.96 (129.84, 233.43) | 17568.20 (15496.46, 19639.95) | 300.46 (239.79, 372.79) | 1.58 (1.45, 1.72) |
| Kingdom of Spain | 91513.20 (85606.43, 97419.97) | 323.01 (269.31, 383.49) | 196154.42 (179817.37, 212491.48) | 322.52 (237.53, 436.79) | 0.66 (0.52, 0.81) |
| Kingdom of Sweden | 12885.27 (11460.91, 14309.63) | 165.77 (123.77, 216.75) | 29628.30 (26419.26, 32837.34) | 286.91 (211.45, 374.84) | 1.15 (1.03, 1.28) |
| Kingdom of Thailand | 25023.69 (22503.36, 27544.03) | 159.64 (120.37, 208.27) | 98405.10 (89520.14, 107290.05) | 322.04 (240.47, 422.73) | 0.61 (0.55, 0.67) |
| Kingdom of the Netherlands | 36381.45 (33919.48, 38843.43) | 353.44 (290.97, 425.31) | 64514.03 (60404.06, 68624.00) | 321.58 (228.42, 411.79) | -0.09 (-0.22, 0.04) |
| Kingdom of Tonga | 38.88 (34.63, 43.12) | 161.21 (120.63, 212.16) | 66.76 (60.08, 73.45) | 394.84 (360.37, 431.61) | 0.48 (-1.33, 2.32) |
| Kyrgyz Republic | 1993.37 (1786.84, 2199.90) | 149.06 (114.77, 193.43) | 2839.43 (2533.32, 3145.55) | 289.94 (223.84, 372.04) | -0.12 (-0.40, 0.16) |
| Lao People's Democratic Republic | 1153.54 (1038.14, 1268.94) | 127.13 (95.50, 164.17) | 2965.44 (2662.58, 3268.31) | 365.00 (274.68, 467.33) | 0.52 (0.16, 0.89) |
| Lebanese Republic | 1377.72 (1242.54, 1512.90) | 144.72 (107.07, 187.98) | 5855.25 (5182.43, 6528.07) | 325.46 (247.46, 408.73) | 1.06 (0.80, 1.33) |
| Malaysia | 6196.66 (5574.16, 6819.16) | 149.01 (112.09, 195.46) | 23922.27 (21100.55, 26743.98) | 422.71 (320.57, 545.34) | 0.89 (0.78, 1.00) |
| Mongolia | 643.56 (569.17, 717.95) | 140.40 (105.18, 185.30) | 1274.18 (1132.53, 1415.83) | 239.08 (178.17, 307.98) | 0.08 (-0.41, 0.57) |
| Montenegro | 597.60 (528.82, 666.38) | 205.24 (153.25, 272.31) | 1004.11 (900.00, 1108.23) | 332.77 (246.54, 425.63) | 0.21 (-0.42, 0.85) |
| New Zealand | 2499.94 (2205.70, 2794.19) | 123.17 (90.38, 161.61) | 6169.77 (5453.03, 6886.50) | 314.61 (246.70, 402.60) | 0.60 (0.31, 0.89) |
| North Macedonia | 1582.56 (1428.17, 1736.96) | 195.41 (150.26, 249.15) | 3324.99 (2980.33, 3669.65) | 357.08 (290.12, 428.27) | 0.30 (-0.08, 0.69) |
| Northern Mariana Islands | 12.97 (11.70, 14.24) | 215.37 (166.02, 273.96) | 45.52 (41.56, 49.49) | 203.33 (151.85, 263.79) | 0.01 (-2.82, 2.93) |
| Palestine | 583.99 (527.18, 640.80) | 155.92 (115.33, 201.10) | 2111.77 (1895.37, 2328.18) | 237.28 (175.46, 299.22) | 1.11 (0.70, 1.52) |
| People's Democratic Republic of Algeria | 8417.07 (7575.60, 9258.54) | 157.01 (117.14, 202.67) | 30294.00 (26804.75, 33783.26) | 376.23 (294.94, 467.92) | 0.96 (0.79, 1.14) |
| People's Republic of Bangladesh | 28545.38 (25561.80, 31528.96) | 138.19 (100.47, 183.76) | 111369.44 (96782.72, 125956.16) | 260.23 (194.12, 338.36) | 0.78 (0.72, 0.84) |
| People's Republic of China | 797843.40 (711339.44, 884347.36) | 218.92 (163.39, 288.51) | 5075679.65 (4578513.26, 5572846.03) | 314.73 (239.99, 404.41) | 2.69 (2.60, 2.79) |
| Plurinational State of Bolivia | 3231.10 (3028.11, 3434.10) | 206.97 (166.32, 249.96) | 13987.82 (12418.18, 15557.45) | 298.21 (230.97, 374.19) | 1.35 (1.18, 1.51) |
| Portuguese Republic | 11873.73 (10912.23, 12835.24) | 171.67 (131.60, 213.95) | 30897.48 (28120.69, 33674.28) | 217.96 (167.57, 279.81) | 1.06 (0.94, 1.18) |
| Principality of Andorra | 69.89 (62.38, 77.41) | 236.94 (175.08, 311.70) | 221.24 (200.31, 242.17) | 253.39 (195.92, 309.86) | 0.69 (-0.64, 2.03) |
| Principality of Monaco | 96.90 (85.81, 107.99) | 251.31 (185.84, 331.16) | 171.37 (149.93, 192.81) | 243.77 (191.80, 310.25) | 0.81 (-0.99, 2.65) |
| Puerto Rico | 2585.57 (2385.35, 2785.79) | 141.80 (108.86, 178.62) | 6224.17 (5688.71, 6759.63) | 417.82 (369.16, 464.89) | 0.61 (0.40, 0.82) |
| Republic of Albania | 1746.93 (1575.73, 1918.13) | 190.11 (143.60, 242.93) | 4520.85 (4048.26, 4993.44) | 236.56 (204.34, 270.63) | 0.22 (-0.10, 0.53) |
| Republic of Angola | 1744.78 (1546.64, 1942.93) | 110.37 (79.90, 147.89) | 6066.02 (5343.43, 6788.61) | 186.65 (138.78, 249.20) | 0.59 (0.31, 0.86) |
| Republic of Armenia | 1827.72 (1666.98, 1988.46) | 154.63 (121.00, 192.15) | 3406.00 (3078.88, 3733.12) | 168.41 (128.07, 213.70) | 0.19 (-0.12, 0.51) |
| Republic of Austria | 13976.82 (12965.39, 14988.25) | 229.56 (180.11, 281.02) | 29552.47 (26365.23, 32739.70) | 170.14 (126.84, 219.55) | 0.84 (0.71, 0.96) |
| Republic of Azerbaijan | 3449.79 (3061.95, 3837.62) | 160.49 (119.41, 214.12) | 7329.08 (6547.46, 8110.70) | 159.54 (120.18, 205.29) | 0.38 (0.18, 0.58) |
| Republic of Belarus | 13431.89 (12198.55, 14665.23) | 217.03 (172.05, 273.93) | 17129.06 (15597.09, 18661.02) | 155.70 (119.33, 197.17) | 0.02 (-0.12, 0.16) |
| Republic of Benin | 1184.17 (1057.81, 1310.53) | 133.61 (99.65, 176.85) | 3102.92 (2801.73, 3404.11) | 159.13 (117.00, 207.25) | 0.48 (0.20, 0.76) |
| Republic of Botswana | 274.24 (244.05, 304.43) | 117.31 (85.26, 156.46) | 851.66 (756.70, 946.62) | 155.12 (116.44, 202.25) | 0.71 (0.06, 1.37) |
| Republic of Bulgaria | 16047.70 (15036.04, 17059.37) | 274.20 (230.43, 320.59) | 15188.54 (13776.58, 16600.50) | 157.58 (116.89, 208.83) | -0.48 (-0.72, -0.23) |
| Republic of Burundi | 1058.13 (943.63, 1172.63) | 104.74 (75.01, 139.07) | 2314.53 (2045.86, 2583.20) | 145.30 (111.98, 187.92) | 0.31 (-0.04, 0.65) |
| Republic of Cabo Verde | 155.84 (140.16, 171.51) | 130.39 (96.67, 169.63) | 342.75 (310.03, 375.47) | 135.33 (100.45, 178.85) | 1.17 (0.31, 2.03) |
| Republic of Cameroon | 2542.61 (2303.23, 2781.99) | 144.05 (107.74, 185.85) | 7794.04 (6934.66, 8653.42) | 154.28 (116.52, 206.19) | 0.49 (0.30, 0.69) |
| Republic of Chad | 1501.21 (1327.03, 1675.39) | 119.66 (86.42, 160.14) | 3044.43 (2678.43, 3410.43) | 168.48 (122.32, 218.72) | 0.57 (0.30, 0.84) |
| Republic of Chile | 8176.37 (7607.48, 8745.26) | 175.46 (142.21, 212.54) | 31399.74 (29065.00, 33734.48) | 148.57 (111.86, 191.44) | 1.26 (1.13, 1.39) |
| Republic of Colombia | 12610.37 (11642.35, 13578.39) | 150.05 (116.86, 186.82) | 53835.40 (49269.27, 58401.52) | 137.55 (101.63, 182.37) | 0.86 (0.79, 0.94) |
| Republic of Costa Rica | 1520.64 (1401.19, 1640.09) | 174.66 (134.49, 220.55) | 5911.72 (5341.06, 6482.38) | 145.57 (107.60, 195.55) | 0.77 (0.56, 0.98) |
| Republic of Côte d'Ivoire | 2150.79 (1937.71, 2363.87) | 191.67 (150.95, 236.54) | 6681.75 (6048.91, 7314.58) | 338.14 (252.50, 451.63) | 0.51 (0.29, 0.72) |
| Republic of Croatia | 5316.20 (4867.92, 5764.49) | 112.19 (85.86, 141.40) | 9770.21 (8847.53, 10692.88) | 326.17 (245.62, 420.47) | 0.14 (-0.11, 0.39) |
| Republic of Cuba | 5672.68 (5236.81, 6108.55) | 275.60 (199.73, 378.97) | 14683.35 (13517.42, 15849.28) | 339.21 (257.82, 443.50) | 1.02 (0.89, 1.15) |
| Republic of Cyprus | 1081.34 (929.57, 1233.11) | 142.82 (106.51, 184.65) | 3318.10 (2870.40, 3765.79) | 205.40 (155.39, 262.88) | 0.32 (-0.29, 0.93) |
| Republic of Djibouti | 60.32 (53.12, 67.52) | 104.56 (74.42, 141.73) | 312.05 (277.66, 346.45) | 228.11 (169.73, 299.22) | 0.57 (-0.73, 1.88) |
| Republic of Ecuador | 4759.78 (4258.00, 5261.56) | 182.91 (135.67, 245.82) | 24956.38 (22782.68, 27130.08) | 210.79 (157.58, 272.67) | 1.68 (1.57, 1.80) |
| Republic of El Salvador | 2200.89 (2018.25, 2383.52) | 151.96 (114.96, 194.21) | 6335.07 (5784.22, 6885.92) | 178.34 (128.54, 235.35) | 0.81 (0.62, 1.00) |
| Republic of Equatorial Guinea | 88.12 (77.77, 98.46) | 110.24 (80.04, 148.16) | 324.17 (287.43, 360.91) | 242.29 (178.46, 330.44) | 1.24 (0.03, 2.46) |
| Republic of Estonia | 2356.84 (2153.32, 2560.35) | 237.99 (185.04, 298.16) | 2988.22 (2748.26, 3228.17) | 213.03 (158.69, 277.24) | -0.24 (-0.60, 0.13) |
| Republic of Fiji | 245.43 (218.83, 272.04) | 175.46 (129.78, 232.00) | 588.09 (514.37, 661.81) | 235.95 (173.33, 302.71) | 0.31 (-0.46, 1.09) |
| Republic of Finland | 7855.90 (7234.77, 8477.04) | 216.70 (174.97, 267.90) | 21570.20 (19074.00, 24066.40) | 229.46 (169.52, 300.84) | 1.21 (1.06, 1.36) |
| Republic of Ghana | 2996.47 (2681.48, 3311.47) | 120.12 (88.95, 157.15) | 9138.81 (8178.88, 10098.74) | 196.26 (150.04, 249.58) | 0.53 (0.36, 0.71) |
| Republic of Guatemala | 2163.80 (1919.64, 2407.96) | 139.74 (101.16, 189.81) | 8897.56 (8007.20, 9787.93) | 191.01 (142.93, 247.67) | 0.64 (0.41, 0.87) |
| Republic of Guinea | 1929.45 (1736.67, 2122.23) | 130.04 (93.54, 169.98) | 3400.67 (3048.51, 3752.82) | 186.47 (138.51, 240.42) | 0.31 (-0.53, 1.16) |
| Republic of Guinea-Bissau | 209.99 (185.88, 234.10) | 135.21 (97.49, 180.44) | 389.91 (347.48, 432.35) | 202.60 (148.35, 271.24) | 0.48 (0.23, 0.73) |
| Republic of Guyana | 203.22 (184.40, 222.04) | 118.41 (88.88, 154.55) | 416.41 (376.82, 455.99) | 245.89 (183.67, 326.01) | 0.36 (-0.30, 1.03) |
| Republic of Haiti | 1686.36 (1520.33, 1852.40) | 113.84 (84.01, 149.21) | 4282.16 (3843.83, 4720.49) | 287.15 (220.72, 363.40) | 0.31 (0.04, 0.59) |
| Republic of Honduras | 1558.12 (1396.05, 1720.20) | 161.57 (119.68, 214.99) | 6644.56 (5830.13, 7458.99) | 219.33 (164.18, 284.48) | 1.11 (0.89, 1.33) |
| Republic of Iceland | 408.57 (376.47, 440.67) | 487.36 (345.74, 684.93) | 1059.59 (957.50, 1161.68) | 202.90 (147.84, 268.86) | 0.84 (0.21, 1.48) |
| Republic of India | 282038.69 (252011.91, 312065.48) | 138.87 (101.95, 184.29) | 1038550.43 (933141.46, 1143959.41) | 160.12 (124.51, 202.32) | 1.33 (1.29, 1.37) |
| Republic of Indonesia | 53297.16 (47632.26, 58962.06) | 128.14 (93.94, 170.06) | 161491.99 (144190.94, 178793.03) | 203.88 (146.77, 267.49) | 0.75 (0.70, 0.79) |
| Republic of Iraq | 4717.23 (4257.04, 5177.41) | 132.77 (96.74, 173.99) | 18839.40 (16879.48, 20799.33) | 195.72 (144.67, 262.70) | 1.56 (1.43, 1.68) |
| Republic of Italy | 167080.51 (152175.44, 181985.58) | 358.95 (282.27, 446.03) | 186761.14 (167449.08, 206073.19) | 193.50 (143.42, 247.52) | -1.14 (-1.40, -0.88) |
| Republic of Kazakhstan | 8735.69 (7765.76, 9705.61) | 159.88 (117.60, 212.13) | 14827.83 (13429.65, 16226.00) | 202.66 (149.94, 265.17) | 0.67 (0.54, 0.81) |
| Republic of Kenya | 4116.11 (3685.84, 4546.38) | 112.66 (82.08, 149.36) | 12236.72 (11017.59, 13455.85) | 214.63 (157.86, 280.34) | 0.48 (0.34, 0.63) |
| Republic of Kiribati | 24.53 (21.95, 27.12) | 165.06 (120.48, 214.93) | 49.51 (43.44, 55.57) | 287.24 (203.98, 380.71) | 0.26 (-2.22, 2.81) |
| Republic of Korea | 15311.61 (13629.13, 16994.10) | 114.79 (85.97, 151.44) | 84759.81 (76966.93, 92552.70) | 341.26 (249.68, 450.95) | 1.48 (1.38, 1.58) |
| Republic of Latvia | 3638.19 (3358.88, 3917.50) | 209.65 (169.00, 257.37) | 4042.92 (3669.32, 4416.53) | 282.14 (207.11, 384.51) | -0.34 (-0.64, -0.03) |
| Republic of Liberia | 635.24 (550.23, 720.26) | 128.61 (95.89, 174.26) | 1181.78 (1063.97, 1299.60) | 201.88 (150.98, 271.58) | 0.50 (0.06, 0.93) |
| Republic of Lithuania | 4269.38 (3828.47, 4710.28) | 195.05 (154.94, 253.89) | 5700.93 (5248.20, 6153.67) | 203.49 (148.48, 265.47) | -0.16 (-0.41, 0.10) |
| Republic of Madagascar | 2007.93 (1775.35, 2240.52) | 94.89 (68.10, 128.65) | 4786.74 (4252.30, 5321.19) | 209.30 (148.68, 280.49) | 0.47 (0.25, 0.69) |
| Republic of Malawi | 1707.48 (1524.63, 1890.32) | 105.53 (76.62, 139.99) | 3653.07 (3248.36, 4057.77) | 297.44 (212.03, 403.98) | 0.36 (0.09, 0.62) |
| Republic of Maldives | 57.72 (52.00, 63.43) | 162.49 (121.44, 209.09) | 270.47 (243.41, 297.53) | 157.04 (117.25, 208.56) | 0.33 (-0.97, 1.64) |
| Republic of Mali | 1831.53 (1652.91, 2010.16) | 118.65 (89.01, 152.68) | 4526.45 (4044.34, 5008.56) | 168.53 (121.78, 219.76) | 0.32 (0.07, 0.57) |
| Republic of Malta | 487.57 (445.44, 529.70) | 233.13 (178.77, 293.41) | 1608.12 (1454.98, 1761.26) | 179.79 (131.80, 246.51) | 0.92 (0.36, 1.49) |
| Republic of Mauritius | 507.02 (463.36, 550.69) | 154.35 (117.47, 194.95) | 1579.26 (1441.40, 1717.12) | 197.96 (140.37, 263.37) | 0.43 (0.00, 0.86) |
| Republic of Moldova | 3939.56 (3509.04, 4370.08) | 197.97 (150.22, 258.22) | 4766.34 (4273.28, 5259.41) | 198.07 (147.70, 258.28) | -0.62 (-0.87, -0.37) |
| Republic of Mozambique | 2490.09 (2195.17, 2785.01) | 100.76 (72.31, 136.62) | 5212.37 (4629.58, 5795.16) | 175.92 (129.94, 233.83) | 0.43 (0.22, 0.64) |
| Republic of Namibia | 319.96 (286.75, 353.17) | 117.45 (86.00, 153.24) | 795.77 (715.94, 875.59) | 187.93 (138.96, 246.72) | 0.63 (0.03, 1.24) |
| Republic of Nauru | 3.34 (2.93, 3.75) | 197.82 (147.39, 265.92) | 4.63 (4.14, 5.12) | 133.60 (94.62, 181.87) | 0.35 (-7.13, 8.43) |
| Republic of Nicaragua | 1273.35 (1165.40, 1381.30) | 171.93 (130.96, 219.04) | 5146.76 (4688.27, 5605.25) | 118.54 (84.87, 160.96) | 0.85 (0.62, 1.08) |
| Republic of Niue | 2.12 (1.92, 2.33) | 196.13 (145.64, 254.69) | 2.17 (1.93, 2.41) | 148.27 (107.41, 199.39) | 0.39 (-9.48, 11.33) |
| Republic of Palau | 9.66 (8.69, 10.62) | 223.90 (167.35, 292.51) | 21.14 (18.44, 23.85) | 124.17 (88.43, 169.43) | 0.26 (-3.41, 4.06) |
| Republic of Panama | 1233.15 (1123.23, 1343.07) | 167.91 (128.14, 217.57) | 4822.65 (4354.77, 5290.53) | 164.17 (116.40, 221.27) | 0.86 (0.63, 1.10) |
| Republic of Paraguay | 1591.33 (1432.24, 1750.42) | 147.12 (109.40, 194.20) | 4975.10 (4539.32, 5410.88) | 168.47 (124.88, 226.64) | 0.78 (0.57, 0.99) |
| Republic of Peru | 12107.76 (11042.35, 13173.16) | 206.89 (155.98, 267.20) | 53560.65 (48502.97, 58618.33) | 118.55 (84.72, 160.31) | 1.54 (1.47, 1.62) |
| Republic of Poland | 40202.08 (35682.06, 44722.10) | 192.32 (141.99, 252.64) | 78503.48 (73035.38, 83971.58) | 127.79 (90.10, 175.52) | -0.18 (-0.37, -0.00) |
| Republic of Rwanda | 1223.34 (1086.26, 1360.41) | 105.00 (75.25, 140.03) | 3025.91 (2676.36, 3375.46) | 128.13 (93.30, 170.93) | 0.33 (-0.01, 0.67) |
| Republic of San Marino | 49.54 (45.23, 53.85) | 260.09 (200.71, 328.23) | 115.60 (100.40, 130.79) | 122.77 (88.62, 162.55) | 0.24 (-1.79, 2.31) |
| Republic of Senegal | 1871.30 (1671.88, 2070.71) | 138.25 (103.04, 181.29) | 5119.19 (4585.40, 5652.97) | 128.20 (93.57, 168.66) | 0.58 (0.35, 0.81) |
| Republic of Serbia | 8680.08 (7818.95, 9541.21) | 179.33 (143.86, 224.28) | 16649.17 (15177.75, 18120.59) | 131.95 (97.20, 172.42) | 0.29 (0.10, 0.48) |
| Republic of Seychelles | 49.58 (44.86, 54.30) | 179.85 (137.09, 232.61) | 111.83 (100.09, 123.57) | 111.86 (80.09, 150.09) | 0.56 (-0.89, 2.03) |
| Republic of Sierra Leone | 1094.33 (968.39, 1220.27) | 121.24 (88.42, 161.33) | 2108.23 (1868.29, 2348.18) | 121.42 (86.45, 161.90) | 0.50 (0.18, 0.83) |
| Republic of Singapore | 1379.13 (1268.11, 1490.15) | 135.68 (103.74, 168.64) | 6794.51 (6099.12, 7489.90) | 187.87 (136.75, 238.38) | 0.75 (0.50, 0.99) |
| Republic of Slovenia | 2480.43 (2279.89, 2680.97) | 205.72 (164.54, 254.48) | 4847.59 (4482.25, 5212.93) | 118.54 (84.55, 158.66) | 0.02 (-0.32, 0.35) |
| Republic of South Africa | 11054.43 (9895.71, 12213.16) | 117.62 (85.56, 156.17) | 27711.78 (24861.51, 30562.06) | 119.74 (86.14, 161.39) | 0.55 (0.46, 0.64) |
| Republic of South Sudan | 1176.08 (1030.20, 1321.95) | 105.10 (74.37, 142.00) | 1717.35 (1531.56, 1903.14) | 222.84 (166.63, 293.80) | 0.22 (-0.09, 0.54) |
| Republic of Sudan | 5544.73 (4982.10, 6107.37) | 129.87 (96.21, 168.31) | 15075.23 (13512.09, 16638.37) | 103.32 (72.78, 142.11) | 1.18 (1.04, 1.31) |
| Republic of Suriname | 125.89 (113.51, 138.27) | 105.48 (78.10, 138.99) | 403.24 (362.03, 444.44) | 125.94 (100.38, 156.50) | 0.71 (-0.03, 1.45) |
| Republic of Tajikistan | 2118.24 (1868.14, 2368.34) | 181.64 (129.21, 243.77) | 4402.77 (3896.09, 4909.46) | 128.55 (92.37, 170.64) | 0.35 (0.10, 0.61) |
| Republic of the Congo | 570.38 (514.78, 625.98) | 125.51 (92.98, 162.28) | 1565.43 (1383.06, 1747.80) | 128.04 (91.55, 172.07) | 0.42 (-0.27, 1.11) |
| Republic of the Gambia | 192.95 (174.14, 211.76) | 131.41 (95.96, 170.41) | 633.16 (563.06, 703.26) | 148.76 (109.36, 201.14) | 0.76 (0.04, 1.50) |
| Republic of the Marshall Islands | 11.98 (10.64, 13.32) | 181.73 (133.99, 239.49) | 26.19 (23.06, 29.31) | 126.99 (92.39, 169.24) | 0.51 (-2.99, 4.15) |
| Republic of the Niger | 1297.57 (1158.94, 1436.20) | 119.17 (86.02, 157.42) | 4050.16 (3571.05, 4529.27) | 145.31 (107.20, 188.67) | 0.38 (0.09, 0.67) |
| Republic of the Philippines | 17917.60 (16008.64, 19826.55) | 139.87 (102.64, 185.96) | 53883.56 (48496.27, 59270.86) | 144.24 (106.07, 189.57) | 0.40 (0.32, 0.49) |
| Republic of the Union of Myanmar | 11919.05 (10635.78, 13202.33) | 118.48 (88.57, 155.65) | 33218.07 (29842.23, 36593.91) | 145.15 (105.68, 193.55) | 0.89 (0.79, 0.99) |
| Republic of Trinidad and Tobago | 482.52 (441.56, 523.48) | 117.95 (89.41, 151.16) | 1320.06 (1179.77, 1460.35) | 147.90 (106.74, 195.83) | 0.47 (0.02, 0.92) |
| Republic of Tunisia | 3225.62 (2858.01, 3593.23) | 143.56 (103.34, 191.17) | 11966.63 (10710.96, 13222.30) | 152.05 (111.57, 196.94) | 1.30 (1.11, 1.49) |
| Republic of Turkey | 21013.43 (18842.68, 23184.19) | 138.93 (101.67, 183.40) | 87324.63 (76965.22, 97684.04) | 145.31 (109.56, 190.42) | 1.30 (1.24, 1.36) |
| Republic of Uganda | 3018.97 (2678.11, 3359.83) | 108.77 (78.65, 145.99) | 7708.17 (6899.30, 8517.04) | 164.45 (119.48, 220.24) | 0.56 (0.38, 0.75) |
| Republic of Uzbekistan | 8698.91 (7776.61, 9621.22) | 167.52 (123.61, 220.14) | 19258.07 (17550.45, 20965.70) | 177.99 (130.66, 231.21) | 0.22 (0.10, 0.34) |
| Republic of Vanuatu | 44.94 (39.74, 50.14) | 180.91 (133.73, 240.62) | 129.57 (115.40, 143.74) | 134.82 (98.35, 184.45) | 0.34 (-1.39, 2.10) |
| Republic of Yemen | 2300.66 (2085.03, 2516.29) | 109.87 (80.66, 141.55) | 9010.68 (8006.91, 10014.45) | 163.57 (121.08, 211.56) | 1.29 (1.08, 1.50) |
| Republic of Zambia | 1349.64 (1206.57, 1492.71) | 112.40 (81.31, 148.80) | 3562.15 (3158.98, 3965.32) | 160.78 (116.00, 213.98) | 0.46 (0.17, 0.76) |
| Republic of Zimbabwe | 2365.90 (2113.61, 2618.19) | 138.60 (100.61, 182.70) | 3722.56 (3331.71, 4113.41) | 144.79 (107.14, 190.20) | -0.05 (-0.28, 0.17) |
| Romania | 21368.75 (19528.77, 23208.74) | 162.71 (129.03, 201.78) | 35502.80 (32346.55, 38659.05) | 149.43 (110.16, 195.83) | 0.39 (0.27, 0.51) |
| Russian Federation | 159689.80 (142633.21, 176746.38) | 186.64 (140.33, 242.52) | 220356.31 (197521.09, 243191.53) | 149.61 (109.67, 200.70) | 0.07 (0.01, 0.14) |
| Saint Kitts and Nevis | 23.87 (21.56, 26.18) | 133.95 (99.60, 174.90) | 50.44 (45.31, 55.58) | 147.08 (106.92, 191.78) | 0.55 (-1.58, 2.72) |
| Saint Lucia | 58.44 (53.52, 63.35) | 138.39 (105.52, 176.38) | 190.04 (172.63, 207.46) | 132.16 (99.93, 173.17) | 0.42 (-0.86, 1.72) |
| Saint Vincent and the Grenadines | 40.87 (36.76, 44.98) | 119.86 (87.71, 157.28) | 99.06 (90.36, 107.76) | 154.98 (115.48, 210.37) | 0.54 (-0.98, 2.08) |
| Slovak Republic | 5067.92 (4599.70, 5536.13) | 174.32 (136.06, 219.75) | 8455.99 (7557.32, 9354.66) | 130.15 (94.22, 175.72) | 0.04 (-0.18, 0.26) |
| Socialist Republic of Viet Nam | 28806.08 (25440.71, 32171.46) | 154.64 (116.14, 205.90) | 92323.31 (82351.18, 102295.44) | 165.88 (121.88, 216.88) | 1.13 (1.07, 1.19) |
| Solomon Islands | 91.31 (80.84, 101.79) | 165.92 (121.54, 219.83) | 245.01 (217.03, 273.00) | 179.60 (132.66, 240.31) | 0.37 (-0.96, 1.71) |
| State of Eritrea | 422.45 (375.43, 469.48) | 104.38 (75.56, 138.64) | 1327.95 (1185.78, 1470.13) | 165.26 (123.80, 216.41) | 0.44 (-0.26, 1.13) |
| State of Israel | 8966.48 (8204.55, 9728.41) | 358.24 (285.89, 442.91) | 26255.15 (23571.53, 28938.78) | 138.34 (102.34, 184.68) | 0.05 (-0.08, 0.18) |
| State of Kuwait | 396.64 (359.61, 433.66) | 156.95 (122.48, 200.18) | 2319.94 (2090.40, 2549.49) | 144.01 (100.66, 195.22) | 0.76 (0.38, 1.14) |
| State of Libya | 1174.64 (1063.01, 1286.27) | 138.74 (103.71, 180.51) | 4210.77 (3839.73, 4581.81) | 156.00 (117.00, 200.07) | 1.35 (1.10, 1.59) |
| State of Qatar | 87.57 (77.41, 97.74) | 220.83 (162.41, 295.30) | 951.24 (841.01, 1061.47) | 201.48 (153.43, 254.34) | 1.21 (0.02, 2.42) |
| Sultanate of Oman | 490.25 (435.04, 545.45) | 177.82 (129.20, 237.59) | 2016.18 (1793.20, 2239.16) | 220.51 (164.29, 295.83) | 1.37 (0.98, 1.76) |
| Swiss Confederation | 13288.74 (12238.64, 14338.83) | 242.38 (188.28, 300.07) | 29786.32 (26826.18, 32746.46) | 245.25 (183.22, 313.22) | 0.78 (0.66, 0.90) |
| Syrian Arab Republic | 2946.69 (2653.33, 3240.06) | 137.32 (100.45, 179.91) | 10600.40 (9359.36, 11841.44) | 207.86 (157.55, 282.05) | 1.42 (1.25, 1.60) |
| Taiwan (Province of China) | 10120.12 (9557.71, 10682.53) | 141.99 (119.81, 166.26) | 84645.26 (82117.71, 87172.80) | 332.12 (243.98, 443.24) | 3.51 (3.24, 3.78) |
| Togolese Republic | 626.04 (566.73, 685.35) | 127.10 (94.89, 164.06) | 1999.76 (1768.54, 2230.99) | 210.37 (155.14, 275.11) | 0.50 (0.12, 0.88) |
| Tokelau | 1.08 (0.94, 1.23) | 174.50 (127.81, 235.86) | 1.41 (1.27, 1.54) | 217.35 (162.98, 285.87) | 0.45 (-12.37, 15.15) |
| Turkmenistan | 1101.13 (998.22, 1204.04) | 133.06 (104.17, 168.82) | 2293.54 (2091.00, 2496.07) | 230.53 (176.96, 288.08) | 0.21 (-0.13, 0.54) |
| Tuvalu | 4.91 (4.24, 5.58) | 173.45 (130.16, 233.67) | 8.75 (7.82, 9.68) | 242.51 (177.31, 328.85) | 0.47 (-5.54, 6.87) |
| Ukraine | 71586.11 (63974.96, 79197.27) | 208.37 (156.64, 270.68) | 77452.90 (69473.23, 85432.57) | 174.47 (126.84, 223.98) | -0.06 (-0.12, 0.00) |
| Union of the Comoros | 93.77 (83.97, 103.57) | 111.14 (80.04, 147.20) | 262.46 (229.63, 295.29) | 168.01 (124.44, 219.45) | 0.38 (-0.71, 1.49) |
| United Arab Emirates | 394.30 (352.78, 435.81) | 215.47 (155.86, 280.88) | 4577.27 (3966.48, 5188.06) | 288.35 (210.08, 390.76) | 1.17 (0.63, 1.72) |
| United Kingdom of Great Britain and Northern Ireland | 126009.01 (114454.46, 137563.55) | 273.68 (213.58, 344.99) | 198254.68 (180801.98, 215707.38) | 197.92 (147.68, 256.00) | 0.06 (-0.05, 0.18) |
| United Mexican States | 30891.64 (27805.74, 33977.53) | 152.77 (112.51, 202.32) | 122599.46 (111019.27, 134179.65) | 196.88 (146.35, 259.38) | 0.82 (0.77, 0.87) |
| United Republic of Tanzania | 4782.23 (4392.20, 5172.27) | 101.22 (79.28, 126.85) | 13325.76 (12284.50, 14367.02) | 217.07 (166.46, 278.53) | 0.60 (0.45, 0.75) |
| United States of America | 320335.17 (288178.44, 352491.90) | 193.13 (146.09, 247.00) | 691737.87 (654679.82, 728795.93) | 114.16 (78.40, 152.92) | 0.38 (0.27, 0.49) |
| United States Virgin Islands | 70.15 (63.25, 77.05) | 175.42 (129.83, 230.51) | 195.61 (177.02, 214.21) | 187.55 (135.27, 247.10) | 0.47 (-0.86, 1.82) |

Abbreviations: APC, Age - Period - Cohort, ASPR, age-standardized prevalence rate, ASR, age-standardized rate.

**Supplementary Table S2: The local drift of prevalence from 1992 to 2021 Parkinson’s disease for fourteen age groups across regions**

| **Location** | **Age** | **Local drift (%/year)** |
| --- | --- | --- |
| Global | 30–34 years | 0.55 (0.21, 0.89) |
| Global | 35–39 years | 0.80 (0.60, 1.01) |
| Global | 40–44 years | 1.01 (0.87, 1.15) |
| Global | 45–49 years | 1.32 (1.22, 1.42) |
| Global | 50–54 years | 1.57 (1.49, 1.64) |
| Global | 55–59 years | 1.59 (1.53, 1.66) |
| Global | 60–64 years | 1.61 (1.55, 1.66) |
| Global | 65–69 years | 1.60 (1.55, 1.64) |
| Global | 70–74 years | 1.46 (1.41, 1.50) |
| Global | 75–79 years | 1.44 (1.39, 1.49) |
| Global | 80–84 years | 1.46 (1.41, 1.52) |
| Global | 85–89 years | 1.52 (1.44, 1.61) |
| Global | 90–94 years | 1.59 (1.43, 1.74) |
| Global | 95+ years | 1.57 (1.19, 1.95) |
| High SDI | 30–34 years | 0.11 (–0.24, 0.46) |
| High SDI | 35–39 years | 0.28 (0.07, 0.49) |
| High SDI | 40–44 years | 0.50 (0.36, 0.64) |
| High SDI | 45–49 years | 0.73 (0.63, 0.83) |
| High SDI | 50–54 years | 0.86 (0.79, 0.94) |
| High SDI | 55–59 years | 0.86 (0.81, 0.92) |
| High SDI | 60–64 years | 0.87 (0.83, 0.92) |
| High SDI | 65–69 years | 0.89 (0.86, 0.93) |
| High SDI | 70–74 years | 0.94 (0.91, 0.97) |
| High SDI | 75–79 years | 1.11 (1.08, 1.14) |
| High SDI | 80–84 years | 1.20 (1.17, 1.24) |
| High SDI | 85–89 years | 1.17 (1.12, 1.21) |
| High SDI | 90–94 years | 1.09 (1.01, 1.17) |
| High SDI | 95+ years | 0.94 (0.76, 1.13) |
| High-middle SDI | 30–34 years | 0.59 ( - 0.17, 1.34) |
| High-middle SDI | 35–39 years | 0.96 (0.51, 1.41) |
| High-middle SDI | 40–44 years | 1.36 (1.07, 1.65) |
| High-middle SDI | 45–49 years | 1.81 (1.62, 1.99) |
| High-middle SDI | 50–54 years | 2.09 (1.96, 2.23) |
| High-middle SDI | 55–59 years | 2.11 (1.99, 2.22) |
| High-middle SDI | 60–64 years | 2.01 (1.91, 2.10) |
| High-middle SDI | 65–69 years | 1.87 (1.79, 1.95) |
| High-middle SDI | 70–74 years | 1.52 (1.45, 1.60) |
| High-middle SDI | 75–79 years | 1.27 (1.19, 1.35) |
| High-middle SDI | 80–84 years | 1.18 (1.09, 1.28) |
| High-middle SDI | 85–89 years | 1.33 (1.18, 1.47) |
| High-middle SDI | 90–94 years | 1.59 (1.31, 1.87) |
| High-middle SDI | 95+ years | 1.85 (1.12, 2.59) |
| Middle SDI | 30–34 years | 0.66 (0.27, 1.06) |
| Middle SDI | 35–39 years | 0.92 (0.68, 1.16) |
| Middle SDI | 40–44 years | 1.04 (0.87, 1.20) |
| Middle SDI | 45–49 years | 1.28 (1.16, 1.39) |
| Middle SDI | 50–54 years | 1.55 (1.46, 1.64) |
| Middle SDI | 55–59 years | 1.73 (1.66, 1.81) |
| Middle SDI | 60–64 years | 2.00 (1.93, 2.06) |
| Middle SDI | 65–69 years | 2.29 (2.22, 2.35) |
| Middle SDI | 70–74 years | 2.36 (2.30, 2.42) |
| Middle SDI | 75–79 years | 2.42 (2.35, 2.49) |
| Middle SDI | 80–84 years | 2.54 (2.45, 2.63) |
| Middle SDI | 85–89 years | 2.69 (2.55, 2.84) |
| Middle SDI | 90–94 years | 2.82 (2.53, 3.12) |
| Middle SDI | 95+ years | 2.78 (2.06, 3.50) |
| Low-middle SDI | 30–34 years | 0.42 (0.32, 0.52) |
| Low-middle SDI | 35–39 years | 0.56 (0.50, 0.62) |
| Low-middle SDI | 40–44 years | 0.71 (0.66, 0.75) |
| Low-middle SDI | 45–49 years | 0.85 (0.81, 0.88) |
| Low-middle SDI | 50–54 years | 0.95 (0.92, 0.98) |
| Low-middle SDI | 55–59 years | 1.02 (1.00, 1.05) |
| Low-middle SDI | 60–64 years | 1.08 (1.06, 1.11) |
| Low-middle SDI | 65–69 years | 1.15 (1.12, 1.17) |
| Low-middle SDI | 70–74 years | 1.22 (1.20, 1.24) |
| Low-middle SDI | 75–79 years | 1.29 (1.27, 1.31) |
| Low-middle SDI | 80–84 years | 1.39 (1.36, 1.42) |
| Low-middle SDI | 85–89 years | 1.53 (1.48, 1.58) |
| Low-middle SDI | 90–94 years | 1.59 (1.49, 1.69) |
| Low-middle SDI | 95+ years | 1.60 (1.38, 1.83) |
| Low SDI | 30–34 years | 0.19 (0.06, 0.33) |
| Low SDI | 35–39 years | 0.28 (0.18, 0.37) |
| Low SDI | 40–44 years | 0.35 (0.28, 0.42) |
| Low SDI | 45–49 years | 0.44 (0.38, 0.49) |
| Low SDI | 50–54 years | 0.54 (0.49, 0.59) |
| Low SDI | 55–59 years | 0.64 (0.60, 0.69) |
| Low SDI | 60–64 years | 0.75 (0.71, 0.79) |
| Low SDI | 65–69 years | 0.84 (0.81, 0.88) |
| Low SDI | 70–74 years | 0.90 (0.87, 0.94) |
| Low SDI | 75–79 years | 0.96 (0.92, 1.00) |
| Low SDI | 80–84 years | 1.04 (0.99, 1.10) |
| Low SDI | 85–89 years | 1.15 (1.06, 1.24) |
| Low SDI | 90–94 years | 1.19 (1.01, 1.38) |
| Low SDI | 95+ years | 1.19 (0.74, 1.65) |
| Central Europe, Eastern Europe, and Central Asia | 30–34 years | - 0.12 ( - 0.39, 0.15) |
| Central Europe, Eastern Europe, and Central Asia | 35–39 years | -0.16 (-0.32, -0.00) |
| Central Europe, Eastern Europe, and Central Asia | 40–44 years | -0.16 (-0.27, -0.05) |
| Central Europe, Eastern Europe, and Central Asia | 45–49 years | -0.11 (-0.18, -0.03) |
| Central Europe, Eastern Europe, and Central Asia | 50–54 years | -0.02 (-0.08, 0.04) |
| Central Europe, Eastern Europe, and Central Asia | 55–59 years | 0.08 (0.04, 0.12) |
| Central Europe, Eastern Europe, and Central Asia | 60–64 years | 0.13 (0.09, 0.16) |
| Central Europe, Eastern Europe, and Central Asia | 65–69 years | 0.16 (0.13, 0.19) |
| Central Europe, Eastern Europe, and Central Asia | 70–74 years | 0.15 (0.12, 0.17) |
| Central Europe, Eastern Europe, and Central Asia | 75–79 years | 0.13 (0.11, 0.16) |
| Central Europe, Eastern Europe, and Central Asia | 80–84 years | 0.11 (0.08, 0.14) |
| Central Europe, Eastern Europe, and Central Asia | 85–89 years | 0.09 (0.05, 0.14) |
| Central Europe, Eastern Europe, and Central Asia | 90–94 years | 0.06 (-0.04, 0.15) |
| Central Europe, Eastern Europe, and Central Asia | 95+ years | -0.10 (-0.37, 0.18) |
| High-income | 30–34 years | 0.14 (-0.32, 0.61) |
| High-income | 35–39 years | 0.27 (-0.01, 0.55) |
| High-income | 40–44 years | 0.38 (0.19, 0.57) |
| High-income | 45–49 years | 0.42 (0.29, 0.55) |
| High-income | 50–54 years | 0.42 (0.32, 0.52) |
| High-income | 55–59 years | 0.35 (0.27, 0.42) |
| High-income | 60–64 years | 0.31 (0.26, 0.37) |
| High-income | 65–69 years | 0.33 (0.28, 0.37) |
| High-income | 70–74 years | 0.39 (0.35, 0.43) |
| High-income | 75–79 years | 0.55 (0.52, 0.59) |
| High-income | 80–84 years | 0.65 (0.61, 0.69) |
| High-income | 85–89 years | 0.67 (0.61, 0.72) |
| High-income | 90–94 years | 0.66 (0.56, 0.75) |
| High-income | 95+ years | 0.57 (0.35, 0.79) |
| Latin America and Caribbean | 30–34 years | 0.49 (0.24, 0.74) |
| Latin America and Caribbean | 35–39 years | 0.64 (0.50, 0.79) |
| Latin America and Caribbean | 40–44 years | 0.78 (0.68, 0.89) |
| Latin America and Caribbean | 45–49 years | 0.91 (0.82, 0.99) |
| Latin America and Caribbean | 50–54 years | 0.97 (0.90, 1.04) |
| Latin America and Caribbean | 55–59 years | 0.98 (0.92, 1.04) |
| Latin America and Caribbean | 60–64 years | 0.98 (0.92, 1.03) |
| Latin America and Caribbean | 65–69 years | 0.96 (0.91, 1.01) |
| Latin America and Caribbean | 70–74 years | 0.96 (0.91, 1.01) |
| Latin America and Caribbean | 75–79 years | 0.99 (0.94, 1.05) |
| Latin America and Caribbean | 80–84 years | 1.05 (0.98, 1.11) |
| Latin America and Caribbean | 85–89 years | 1.08 (0.99, 1.17) |
| Latin America and Caribbean | 90–94 years | 1.05 (0.89, 1.22) |
| Latin America and Caribbean | 95+ years | 0.97 (0.62, 1.32) |
| North Africa and Middle East | 30–34 years | 0.55 (0.37, 0.73) |
| North Africa and Middle East | 35–39 years | 0.72 (0.61, 0.83) |
| North Africa and Middle East | 40–44 years | 0.88 (0.80, 0.96) |
| North Africa and Middle East | 45–49 years | 1.01 (0.95, 1.08) |
| North Africa and Middle East | 50–54 years | 1.13 (1.08, 1.19) |
| North Africa and Middle East | 55–59 years | 1.23 (1.19, 1.28) |
| North Africa and Middle East | 60–64 years | 1.30 (1.26, 1.34) |
| North Africa and Middle East | 65–69 years | 1.33 (1.30, 1.37) |
| North Africa and Middle East | 70–74 years | 1.37 (1.33, 1.40) |
| North Africa and Middle East | 75–79 years | 1.40 (1.37, 1.44) |
| North Africa and Middle East | 80–84 years | 1.51 (1.47, 1.56) |
| North Africa and Middle East | 85–89 years | 1.75 (1.67, 1.82) |
| North Africa and Middle East | 90–94 years | 1.94 (1.80, 2.07) |
| North Africa and Middle East | 95+ years | 2.01 (1.72, 2.30) |
| South Asia | 30–34 years | 0.49 (0.35, 0.63) |
| South Asia | 35–39 years | 0.64 (0.55, 0.73) |
| South Asia | 40–44 years | 0.79 (0.73, 0.86) |
| South Asia | 45–49 years | 0.94 (0.89, 0.99) |
| South Asia | 50–54 years | 1.05 (1.01, 1.10) |
| South Asia | 55–59 years | 1.11 (1.08, 1.15) |
| South Asia | 60–64 years | 1.14 (1.11, 1.18) |
| South Asia | 65–69 years | 1.17 (1.14, 1.20) |
| South Asia | 70–74 years | 1.24 (1.21, 1.27) |
| South Asia | 75–79 years | 1.38 (1.35, 1.42) |
| South Asia | 80–84 years | 1.61 (1.56, 1.66) |
| South Asia | 85–89 years | 1.90 (1.82, 1.98) |
| South Asia | 90–94 years | 2.06 (1.89, 2.24) |
| South Asia | 95+ years | 2.03 (1.58, 2.49) |
| Southeast Asia, East Asia, and Oceania | 30–34 years | 0.76 (0.14, 1.38) |
| Southeast Asia, East Asia, and Oceania | 35–39 years | 1.02 (0.65, 1.39) |
| Southeast Asia, East Asia, and Oceania | 40–44 years | 1.29 (1.06, 1.52) |
| Southeast Asia, East Asia, and Oceania | 45–49 years | 1.66 (1.51, 1.81) |
| Southeast Asia, East Asia, and Oceania | 50–54 years | 2.00 (1.89, 2.11) |
| Southeast Asia, East Asia, and Oceania | 55–59 years | 2.27 (2.18, 2.36) |
| Southeast Asia, East Asia, and Oceania | 60–64 years | 2.55 (2.47, 2.64) |
| Southeast Asia, East Asia, and Oceania | 65–69 years | 2.82 (2.75, 2.90) |
| Southeast Asia, East Asia, and Oceania | 70–74 years | 2.94 (2.87, 3.02) |
| Southeast Asia, East Asia, and Oceania | 75–79 years | 3.06 (2.97, 3.14) |
| Southeast Asia, East Asia, and Oceania | 80–84 years | 3.23 (3.11, 3.35) |
| Southeast Asia, East Asia, and Oceania | 85–89 years | 3.46 (3.27, 3.66) |
| Southeast Asia, East Asia, and Oceania | 90–94 years | 3.73 (3.33, 4.14) |
| Southeast Asia, East Asia, and Oceania | 95+ years | 3.91 (2.85, 4.98) |
| Sub-Saharan Africa | 30–34 years | 0.13 (-0.01, 0.27) |
| Sub-Saharan Africa | 35–39 years | 0.15 (0.06, 0.25) |
| Sub-Saharan Africa | 40–44 years | 0.18 (0.11, 0.26) |
| Sub-Saharan Africa | 45–49 years | 0.21 (0.15, 0.27) |
| Sub-Saharan Africa | 50–54 years | 0.25 (0.20, 0.31) |
| Sub-Saharan Africa | 55–59 years | 0.32 (0.27, 0.37) |
| Sub-Saharan Africa | 60–64 years | 0.41 (0.37, 0.45) |
| Sub-Saharan Africa | 65–69 years | 0.50 (0.47, 0.54) |
| Sub-Saharan Africa | 70–74 years | 0.60 (0.56, 0.64) |
| Sub-Saharan Africa | 75–79 years | 0.69 (0.65, 0.72) |
| Sub-Saharan Africa | 80–84 years | 0.74 (0.69, 0.79) |
| Sub-Saharan Africa | 85–89 years | 0.78 (0.70, 0.85) |
| Sub-Saharan Africa | 90–94 years | 0.81 (0.66, 0.95) |
| Sub-Saharan Africa | 95+ years | 0.85 (0.49, 1.20) |

**Supplementary Table S3: The local drift of prevalence from 1992 to 2021 Parkinson’s disease for fourteen age groups across 204 countries**

| **Location** | **Age** | **Local drift (%/year)** |
| --- | --- | --- |
| American Samoa | 30–34 years | 0.28 (-18.58, 23.51) |
| American Samoa | 35–39 years | 0.30 (-11.48, 13.64) |
| American Samoa | 40–44 years | 0.30 (-7.94, 9.28) |
| American Samoa | 45–49 years | 0.32 (-5.82, 6.87) |
| American Samoa | 50–54 years | 0.34 (-4.48, 5.40) |
| American Samoa | 55–59 years | 0.34 (-3.69, 4.53) |
| American Samoa | 60–64 years | 0.31 (-3.21, 3.96) |
| American Samoa | 65–69 years | 0.28 (-2.91, 3.58) |
| American Samoa | 70–74 years | 0.28 (-2.80, 3.45) |
| American Samoa | 75–79 years | 0.29 (-3.03, 3.72) |
| American Samoa | 80–84 years | 0.33 (-3.90, 4.74) |
| American Samoa | 85–89 years | 0.43 (-6.23, 7.55) |
| American Samoa | 90–94 years | 0.68 (-11.39, 14.38) |
| American Samoa | 95+ years | 0.99 ( - 26.62, 39.00) |
| Antigua and Barbuda | 30–34 years | -0.02 (-10.51, 11.70) |
| Antigua and Barbuda | 35–39 years | 0.10 (-6.20, 6.83) |
| Antigua and Barbuda | 40–44 years | 0.20 (-4.24, 4.84) |
| Antigua and Barbuda | 45–49 years | 0.32 (-3.10, 3.87) |
| Antigua and Barbuda | 50–54 years | 0.45 (-2.42, 3.41) |
| Antigua and Barbuda | 55–59 years | 0.59 (-1.99, 3.23) |
| Antigua and Barbuda | 60–64 years | 0.74 (-1.64, 3.18) |
| Antigua and Barbuda | 65–69 years | 0.86 (-1.36, 3.12) |
| Antigua and Barbuda | 70–74 years | 0.93 (-1.17, 3.08) |
| Antigua and Barbuda | 75–79 years | 0.97 (-1.15, 3.13) |
| Antigua and Barbuda | 80–84 years | 0.96 (-1.44, 3.42) |
| Antigua and Barbuda | 85–89 years | 0.89 (-2.33, 4.22) |
| Antigua and Barbuda | 90–94 years | 0.77 (-4.42, 6.24) |
| Antigua and Barbuda | 95+ years | 0.60 ( - 9.43, 11.74) |
| Arab Republic of Egypt | 30–34 years | 0.56 (0.11, 1.01) |
| Arab Republic of Egypt | 35–39 years | 0.82 (0.55, 1.09) |
| Arab Republic of Egypt | 40–44 years | 1.07 (0.88, 1.26) |
| Arab Republic of Egypt | 45–49 years | 1.27 (1.12, 1.41) |
| Arab Republic of Egypt | 50–54 years | 1.37 (1.25, 1.48) |
| Arab Republic of Egypt | 55–59 years | 1.40 (1.30, 1.50) |
| Arab Republic of Egypt | 60–64 years | 1.40 (1.31, 1.48) |
| Arab Republic of Egypt | 65–69 years | 1.42 (1.34, 1.49) |
| Arab Republic of Egypt | 70–74 years | 1.47 (1.38, 1.55) |
| Arab Republic of Egypt | 75–79 years | 1.46 (1.37, 1.55) |
| Arab Republic of Egypt | 80–84 years | 1.50 (1.38, 1.62) |
| Arab Republic of Egypt | 85–89 years | 1.68 (1.49, 1.86) |
| Arab Republic of Egypt | 90–94 years | 1.81 (1.42, 2.21) |
| Arab Republic of Egypt | 95+ years | 1.87 (0.81, 2.94) |
| Argentine Republic | 30–34 years | 0.59 (-0.25, 1.44) |
| Argentine Republic | 35–39 years | 0.74 (0.22, 1.26) |
| Argentine Republic | 40–44 years | 0.73 (0.36, 1.10) |
| Argentine Republic | 45–49 years | 0.58 (0.31, 0.85) |
| Argentine Republic | 50–54 years | 0.36 (0.16, 0.57) |
| Argentine Republic | 55–59 years | 0.18 (0.03, 0.33) |
| Argentine Republic | 60–64 years | 0.12 (0.01, 0.23) |
| Argentine Republic | 65–69 years | 0.19 (0.10, 0.28) |
| Argentine Republic | 70–74 years | 0.30 (0.23, 0.38) |
| Argentine Republic | 75–79 years | 0.36 (0.29, 0.44) |
| Argentine Republic | 80–84 years | 0.33 (0.24, 0.41) |
| Argentine Republic | 85–89 years | 0.18 (0.06, 0.30) |
| Argentine Republic | 90–94 years | 0.04 (-0.17, 0.25) |
| Argentine Republic | 95+ years | -0.00 (-0.48, 0.48) |
| Australia | 30–34 years | 0.75 (-0.38, 1.89) |
| Australia | 35–39 years | 0.84 (0.12, 1.55) |
| Australia | 40–44 years | 0.83 (0.32, 1.34) |
| Australia | 45–49 years | 0.70 (0.33, 1.07) |
| Australia | 50–54 years | 0.52 (0.25, 0.79) |
| Australia | 55–59 years | 0.40 (0.20, 0.60) |
| Australia | 60–64 years | 0.46 (0.31, 0.61) |
| Australia | 65–69 years | 0.66 (0.55, 0.77) |
| Australia | 70–74 years | 0.88 (0.79, 0.97) |
| Australia | 75–79 years | 1.08 (0.98, 1.17) |
| Australia | 80–84 years | 1.27 (1.16, 1.37) |
| Australia | 85–89 years | 1.48 (1.32, 1.64) |
| Australia | 90–94 years | 1.73 (1.42, 2.05) |
| Australia | 95+ years | 2.06 (1.25, 2.88) |
| Barbados | 30–34 years | 0.19 (-6.05, 6.83) |
| Barbados | 35–39 years | 0.26 (-3.32, 3.97) |
| Barbados | 40–44 years | 0.31 (-2.15, 2.83) |
| Barbados | 45–49 years | 0.38 (-1.43, 2.23) |
| Barbados | 50–54 years | 0.45 (-1.01, 1.94) |
| Barbados | 55–59 years | 0.51 (-0.74, 1.78) |
| Barbados | 60–64 years | 0.57 (-0.53, 1.68) |
| Barbados | 65–69 years | 0.62 (-0.37, 1.61) |
| Barbados | 70–74 years | 0.66 (-0.26, 1.58) |
| Barbados | 75–79 years | 0.70 (-0.20, 1.61) |
| Barbados | 80–84 years | 0.71 (-0.25, 1.69) |
| Barbados | 85–89 years | 0.68 (-0.59, 1.96) |
| Barbados | 90–94 years | 0.61 (-1.63, 2.90) |
| Barbados | 95+ years | 0.52 ( - 4.65, 5.97) |
| Belize | 30-34 years | 0.24 ( - 5.30, 6.11) |
| Belize | 35–39 years | 0.38 ( - 3.15, 4.05) |
| Belize | 40–44 years | 0.50 (-2.16, 3.23) |
| Belize | 45–49 years | 0.61 (-1.57, 2.84) |
| Belize | 50–54 years | 0.68 (-1.23, 2.63) |
| Belize | 55–59 years | 0.70 (-1.04, 2.47) |
| Belize | 60–64 years | 0.70 (-0.93, 2.36) |
| Belize | 65–69 years | 0.71 (-0.85, 2.28) |
| Belize | 70–74 years | 0.73 (-0.82, 2.30) |
| Belize | 75–79 years | 0.76 (-0.89, 2.43) |
| Belize | 80–84 years | 0.75 (-1.19, 2.72) |
| Belize | 85–89 years | 0.64 (-1.94, 3.29) |
| Belize | 90–94 years | 0.57 (-3.31, 4.60) |
| Belize | 95+ years | 0.46 ( - 6.02, 7.38) |
| Bermuda | 30–34 years | -0.08 (-13.09, 14.89) |
| Bermuda | 35–39 years | 0.04 (-7.16, 7.81) |
| Bermuda | 40–44 years | 0.10 (-4.67, 5.10) |
| Bermuda | 45–49 years | 0.11 (-3.31, 3.66) |
| Bermuda | 50–54 years | 0.11 (-2.55, 2.84) |
| Bermuda | 55–59 years | 0.10 (-2.11, 2.37) |
| Bermuda | 60–64 years | 0.15 (-1.81, 2.15) |
| Bermuda | 65–69 years | 0.23 (-1.55, 2.04) |
| Bermuda | 70–74 years | 0.27 (-1.40, 1.96) |
| Bermuda | 75–79 years | 0.26 (-1.38, 1.93) |
| Bermuda | 80–84 years | 0.27 (-1.57, 2.14) |
| Bermuda | 85–89 years | 0.28 (-2.23, 2.84) |
| Bermuda | 90–94 years | 0.32 (-4.11, 4.95) |
| Bermuda | 95+ years | 0.30 (-9.44, 11.08) |
| Bolivarian Republic of Venezuela | 30–34 years | 0.51 (-0.12, 1.13) |
| Bolivarian Republic of Venezuela | 35–39 years | 0.62 (0.26, 0.99) |
| Bolivarian Republic of Venezuela | 40–44 years | 0.74 (0.49, 1.00) |
| Bolivarian Republic of Venezuela | 45–49 years | 0.84 (0.63, 1.05) |
| Bolivarian Republic of Venezuela | 50–54 years | 0.87 (0.69, 1.04) |
| Bolivarian Republic of Venezuela | 55–59 years | 0.82 (0.66, 0.98) |
| Bolivarian Republic of Venezuela | 60–64 years | 0.73 (0.59, 0.87) |
| Bolivarian Republic of Venezuela | 65–69 years | 0.66 (0.53, 0.79) |
| Bolivarian Republic of Venezuela | 70–74 years | 0.68 (0.55, 0.82) |
| Bolivarian Republic of Venezuela | 75–79 years | 0.82 (0.68, 0.96) |
| Bolivarian Republic of Venezuela | 80–84 years | 1.00 (0.84, 1.16) |
| Bolivarian Republic of Venezuela | 85–89 years | 1.07 (0.86, 1.28) |
| Bolivarian Republic of Venezuela | 90–94 years | 1.03 (0.69, 1.37) |
| Bolivarian Republic of Venezuela | 95+ years | 0.98 (0.25, 1.72) |
| Bosnia and Herzegovina | 30–34 years | -0.28 (-3.15, 2.67) |
| Bosnia and Herzegovina | 35–39 years | -0.27 (-1.89, 1.38) |
| Bosnia and Herzegovina | 40–44 years | -0.29 (-1.35, 0.77) |
| Bosnia and Herzegovina | 45–49 years | -0.37 (-1.09, 0.37) |
| Bosnia and Herzegovina | 50–54 years | -0.51 (-1.00, -0.01) |
| Bosnia and Herzegovina | 55–59 years | -0.60 (-0.95, -0.26) |
| Bosnia and Herzegovina | 60–64 years | -0.56 (-0.81, -0.30) |
| Bosnia and Herzegovina | 65–69 years | -0.35 (-0.55, -0.14) |
| Bosnia and Herzegovina | 70–74 years | -0.05 (-0.24, 0.14) |
| Bosnia and Herzegovina | 75–79 years | 0.24 (0.02, 0.46) |
| Bosnia and Herzegovina | 80–84 years | 0.42 (0.17, 0.68) |
| Bosnia and Herzegovina | 85–89 years | 0.46 (0.07, 0.84) |
| Bosnia and Herzegovina | 90–94 years | 0.44 (-0.33, 1.22) |
| Bosnia and Herzegovina | 95+ years | 0.64 (-1.84, 3.18) |
| Brunei Darussalam | 30–34 years | 0.55 (-5.05, 6.48) |
| Brunei Darussalam | 35–39 years | 0.64 (-2.91, 4.33) |
| Brunei Darussalam | 40–44 years | 0.66 (-1.99, 3.39) |
| Brunei Darussalam | 45–49 years | 0.66 (-1.48, 2.85) |
| Brunei Darussalam | 50–54 years | 0.68 (-1.13, 2.53) |
| Brunei Darussalam | 55–59 years | 0.72 (-0.85, 2.33) |
| Brunei Darussalam | 60–64 years | 0.77 (-0.63, 2.19) |
| Brunei Darussalam | 65–69 years | 0.67 (-0.63, 2.00) |
| Brunei Darussalam | 70–74 years | 0.45 (-0.82, 1.74) |
| Brunei Darussalam | 75–79 years | 0.36 (-0.99, 1.72) |
| Brunei Darussalam | 80–84 years | 0.56 (-1.27, 2.42) |
| Brunei Darussalam | 85–89 years | 0.85 (-2.17, 3.96) |
| Brunei Darussalam | 90–94 years | 1.07 (-7.35, 10.27) |
| Brunei Darussalam | 95+ years | 1.69 (-31.43, 50.80) |
| Burkina Faso | 30–34 years | 0.49 (-0.58, 1.58) |
| Burkina Faso | 35–39 years | 0.50 (-0.24, 1.24) |
| Burkina Faso | 40–44 years | 0.49 (-0.10, 1.08) |
| Burkina Faso | 45–49 years | 0.46 (-0.04, 0.96) |
| Burkina Faso | 50–54 years | 0.41 (-0.01, 0.83) |
| Burkina Faso | 55–59 years | 0.36 (0.00, 0.73) |
| Burkina Faso | 60–64 years | 0.34 (0.03, 0.65) |
| Burkina Faso | 65–69 years | 0.34 (0.07, 0.61) |
| Burkina Faso | 70–74 years | 0.35 (0.11, 0.60) |
| Burkina Faso | 75–79 years | 0.38 (0.14, 0.63) |
| Burkina Faso | 80–84 years | 0.41 (0.11, 0.72) |
| Burkina Faso | 85–89 years | 0.44 (-0.08, 0.95) |
| Burkina Faso | 90–94 years | 0.44 (-0.65, 1.54) |
| Burkina Faso | 95+ years | 0.43 (-2.38, 3.32) |
| Canada | 30–34 years | 0.67 (0.01, 1.32) |
| Canada | 35–39 years | 0.75 (0.39, 1.12) |
| Canada | 40–44 years | 0.85 (0.61, 1.09) |
| Canada | 45–49 years | 0.97 (0.81, 1.13) |
| Canada | 50–54 years | 1.09 (0.97, 1.20) |
| Canada | 55–59 years | 1.19 (1.10, 1.27) |
| Canada | 60–64 years | 1.26 (1.20, 1.33) |
| Canada | 65–69 years | 1.34 (1.28, 1.40) |
| Canada | 70–74 years | 1.41 (1.36, 1.46) |
| Canada | 75–79 years | 1.46 (1.41, 1.51) |
| Canada | 80–84 years | 1.49 (1.43, 1.54) |
| Canada | 85–89 years | 1.48 (1.40, 1.55) |
| Canada | 90–94 years | 1.41 (1.28, 1.55) |
| Canada | 95+ years | 1.38 (1.06, 1.69) |
| Central African Republic | 30–34 years | 0.14 (-1.84, 2.16) |
| Central African Republic | 35–39 years | 0.11 (-1.18, 1.42) |
| Central African Republic | 40–44 years | 0.12 (-0.86, 1.11) |
| Central African Republic | 45–49 years | 0.11 (-0.70, 0.92) |
| Central African Republic | 50–54 years | 0.10 (-0.59, 0.80) |
| Central African Republic | 55–59 years | 0.13 (-0.48, 0.75) |
| Central African Republic | 60–64 years | 0.16 (-0.40, 0.73) |
| Central African Republic | 65–69 years | 0.17 (-0.37, 0.72) |
| Central African Republic | 70–74 years | 0.18 (-0.40, 0.76) |
| Central African Republic | 75–79 years | 0.23 (-0.48, 0.95) |
| Central African Republic | 80–84 years | 0.33 (-0.76, 1.43) |
| Central African Republic | 85–89 years | 0.45 (-1.68, 2.63) |
| Central African Republic | 90–94 years | 0.68 (-3.51, 5.06) |
| Central African Republic | 95+ years | 0.84 (-8.10, 10.65) |
| Commonwealth of Dominica | 30–34 years | 0.03 (-12.09, 13.81) |
| Commonwealth of Dominica | 35–39 years | 0.07 (-7.37, 8.11) |
| Commonwealth of Dominica | 40–44 years | 0.11 (-5.23, 5.75) |
| Commonwealth of Dominica | 45–49 years | 0.19 (-3.73, 4.27) |
| Commonwealth of Dominica | 50–54 years | 0.34 (-2.84, 3.62) |
| Commonwealth of Dominica | 55–59 years | 0.53 (-2.26, 3.41) |
| Commonwealth of Dominica | 60–64 years | 0.64 (-1.90, 3.24) |
| Commonwealth of Dominica | 65–69 years | 0.61 (-1.72, 3.00) |
| Commonwealth of Dominica | 70–74 years | 0.57 (-1.62, 2.81) |
| Commonwealth of Dominica | 75–79 years | 0.53 (-1.64, 2.76) |
| Commonwealth of Dominica | 80–84 years | 0.54 (-1.98, 3.12) |
| Commonwealth of Dominica | 85–89 years | 0.55 (-2.97, 4.21) |
| Commonwealth of Dominica | 90–94 years | 0.48 (-5.64, 6.99) |
| Commonwealth of Dominica | 95+ years | 0.15 ( - 12.76, 14.97) |
| Commonwealth of the Bahamas | 30–34 years | 0.17 (-4.96, 5.58) |
| Commonwealth of the Bahamas | 35–39 years | 0.20 (-2.84, 3.34) |
| Commonwealth of the Bahamas | 40–44 years | 0.21 (-1.93, 2.40) |
| Commonwealth of the Bahamas | 45–49 years | 0.26 (-1.41, 1.95) |
| Commonwealth of the Bahamas | 50–54 years | 0.32 (-1.09, 1.75) |
| Commonwealth of the Bahamas | 55–59 years | 0.38 (-0.90, 1.67) |
| Commonwealth of the Bahamas | 60–64 years | 0.41 (-0.81, 1.64) |
| Commonwealth of the Bahamas | 65–69 years | 0.42 (-0.78, 1.64) |
| Commonwealth of the Bahamas | 70–74 years | 0.43 (-0.77, 1.65) |
| Commonwealth of the Bahamas | 75–79 years | 0.47 (-0.78, 1.74) |
| Commonwealth of the Bahamas | 80–84 years | 0.53 (-0.95, 2.03) |
| Commonwealth of the Bahamas | 85–89 years | 0.51 (-1.57, 2.63) |
| Commonwealth of the Bahamas | 90–94 years | 0.41 (-3.07, 4.01) |
| Commonwealth of the Bahamas | 95+ years | 0.23 (-6.44, 7.36) |
| Cook Islands | 30–34 years | 0.22 (-28.76, 40.99) |
| Cook Islands | 35–39 years | 0.21 (-18.43, 23.10) |
| Cook Islands | 40–44 years | 0.17 (-12.57, 14.78) |
| Cook Islands | 45–49 years | 0.14 (-8.81, 9.97) |
| Cook Islands | 50–54 years | 0.09 (-6.43, 7.07) |
| Cook Islands | 55–59 years | 0.08 (-4.95, 5.38) |
| Cook Islands | 60–64 years | 0.03 (-4.19, 4.44) |
| Cook Islands | 65–69 years | 0.03 (-3.60, 3.81) |
| Cook Islands | 70–74 years | 0.10 (-3.35, 3.67) |
| Cook Islands | 75–79 years | 0.30 (-3.17, 3.90) |
| Cook Islands | 80–84 years | 0.55 (-3.70, 5.00) |
| Cook Islands | 85–89 years | 0.91 (-5.41, 7.65) |
| Cook Islands | 90–94 years | 1.19 (-10.38, 14.26) |
| Cook Islands | 95+ years | 1.26 ( - 25.02, 36.75) |
| Czech Republic | 30–34 years | 0.04 (-1.63, 1.73) |
| Czech Republic | 35–39 years | 0.10 (-0.86, 1.07) |
| Czech Republic | 40–44 years | 0.10 (-0.50, 0.72) |
| Czech Republic | 45–49 years | 0.07 (-0.36, 0.50) |
| Czech Republic | 50–54 years | 0.04 (-0.27, 0.36) |
| Czech Republic | 55–59 years | 0.08 ( - 0.15, 0.30) |
| Czech Republic | 60–64 years | 0.17 (0.01, 0.34) |
| Czech Republic | 65–69 years | 0.31 (0.19, 0.43) |
| Czech Republic | 70–74 years | 0.43 (0.32, 0.53) |
| Czech Republic | 75–79 years | 0.52 (0.40, 0.63) |
| Czech Republic | 80–84 years | 0.59 (0.45, 0.72) |
| Czech Republic | 85–89 years | 0.61 (0.42, 0.81) |
| Czech Republic | 90–94 years | 0.61 (0.21, 1.01) |
| Czech Republic | 95+ years | 0.64 (-0.56, 1.86) |
| Democratic People's Republic of Korea | 30–34 years | 0.67 (-0.06, 1.40) |
| Democratic People's Republic of Korea | 35–39 years | 1.04 (0.60, 1.47) |
| Democratic People's Republic of Korea | 40–44 years | 1.42 (1.13, 1.70) |
| Democratic People's Republic of Korea | 45–49 years | 1.73 (1.54, 1.92) |
| Democratic People's Republic of Korea | 50–54 years | 1.95 (1.81, 2.09) |
| Democratic People's Republic of Korea | 55–59 years | 2.10 (1.98, 2.21) |
| Democratic People's Republic of Korea | 60–64 years | 2.24 (2.14, 2.34) |
| Democratic People's Republic of Korea | 65–69 years | 2.38 (2.29, 2.48) |
| Democratic People's Republic of Korea | 70–74 years | 2.48 (2.39, 2.58) |
| Democratic People's Republic of Korea | 75–79 years | 2.53 (2.41, 2.64) |
| Democratic People's Republic of Korea | 80–84 years | 2.53 (2.37, 2.68) |
| Democratic People's Republic of Korea | 85–89 years | 2.56 (2.31, 2.81) |
| Democratic People's Republic of Korea | 90–94 years | 2.64 (2.19, 3.10) |
| Democratic People's Republic of Korea | 95+ years | 2.69 (1.76, 3.63) |
| Democratic Republic of Sao Tome and Principe | 30–34 years | 0.49 (-8.22, 10.04) |
| Democratic Republic of Sao Tome and Principe | 35–39 years | 0.63 (-5.26, 6.90) |
| Democratic Republic of Sao Tome and Principe | 40–44 years | 0.72 (-3.98, 5.65) |
| Democratic Republic of Sao Tome and Principe | 45–49 years | 0.76 (-3.27, 4.96) |
| Democratic Republic of Sao Tome and Principe | 50–54 years | 0.79 (-2.73, 4.43) |
| Democratic Republic of Sao Tome and Principe | 55–59 years | 0.81 (-2.19, 3.90) |
| Democratic Republic of Sao Tome and Principe | 60–64 years | 0.87 (-1.69, 3.50) |
| Democratic Republic of Sao Tome and Principe | 65–69 years | 0.97 (-1.25, 3.24) |
| Democratic Republic of Sao Tome and Principe | 70–74 years | 1.05 (-0.97, 3.10) |
| Democratic Republic of Sao Tome and Principe | 75–79 years | 1.07 (-0.90, 3.07) |
| Democratic Republic of Sao Tome and Principe | 80–84 years | 1.04 (-1.24, 3.36) |
| Democratic Republic of Sao Tome and Principe | 85–89 years | 0.96 (-2.28, 4.32) |
| Democratic Republic of Sao Tome and Principe | 90–94 years | 0.92 (-5.04, 7.25) |
| Democratic Republic of Sao Tome and Principe | 95+ years | 1.02 (-11.64, 15.49) |
| Democratic Republic of the Congo | 30–34 years | 0.14 (-0.39, 0.66) |
| Democratic Republic of the Congo | 35–39 years | 0.21 (-0.15, 0.56) |
| Democratic Republic of the Congo | 40–44 years | 0.29 (0.02, 0.56) |
| Democratic Republic of the Congo | 45–49 years | 0.38 (0.15, 0.60) |
| Democratic Republic of the Congo | 50–54 years | 0.43 (0.24, 0.63) |
| Democratic Republic of the Congo | 55–59 years | 0.49 (0.32, 0.66) |
| Democratic Republic of the Congo | 60–64 years | 0.53 (0.38, 0.68) |
| Democratic Republic of the Congo | 65–69 years | 0.56 (0.42, 0.70) |
| Democratic Republic of the Congo | 70–74 years | 0.57 (0.42, 0.71) |
| Democratic Republic of the Congo | 75–79 years | 0.55 (0.39, 0.71) |
| Democratic Republic of the Congo | 80–84 years | 0.50 (0.26, 0.74) |
| Democratic Republic of the Congo | 85–89 years | 0.46 (0.02, 0.91) |
| Democratic Republic of the Congo | 90–94 years | 0.50 (-0.42, 1.43) |
| Democratic Republic of the Congo | 95+ years | 0.64 (-1.45, 2.77) |
| Democratic Republic of Timor-Leste | 30–34 years | 0.18 (-3.92, 4.45) |
| Democratic Republic of Timor-Leste | 35–39 years | 0.21 (-2.52, 3.01) |
| Democratic Republic of Timor-Leste | 40–44 years | 0.29 (-1.72, 2.33) |
| Democratic Republic of Timor-Leste | 45–49 years | 0.41 (-1.16, 2.01) |
| Democratic Republic of Timor-Leste | 50–54 years | 0.55 (-0.76, 1.88) |
| Democratic Republic of Timor-Leste | 55–59 years | 0.70 (-0.41, 1.82) |
| Democratic Republic of Timor-Leste | 60–64 years | 0.84 (-0.11, 1.79) |
| Democratic Republic of Timor-Leste | 65–69 years | 0.91 (0.05, 1.78) |
| Democratic Republic of Timor-Leste | 70–74 years | 0.95 (0.07, 1.84) |
| Democratic Republic of Timor-Leste | 75–79 years | 1.00 (-0.10, 2.11) |
| Democratic Republic of Timor-Leste | 80–84 years | 1.05 (-0.58, 2.71) |
| Democratic Republic of Timor-Leste | 85–89 years | 1.15 (-1.27, 3.63) |
| Democratic Republic of Timor-Leste | 90–94 years | 1.32 (-2.76, 5.58) |
| Democratic Republic of Timor-Leste | 95+ years | 1.36 (-6.61, 10.02) |
| Democratic Socialist Republic of Sri Lanka | 30–34 years | 0.28 (-0.55, 1.13) |
| Democratic Socialist Republic of Sri Lanka | 35–39 years | 0.33 (-0.17, 0.84) |
| Democratic Socialist Republic of Sri Lanka | 40–44 years | 0.42 (0.06, 0.79) |
| Democratic Socialist Republic of Sri Lanka | 45–49 years | 0.51 (0.23, 0.79) |
| Democratic Socialist Republic of Sri Lanka | 50–54 years | 0.59 (0.38, 0.81) |
| Democratic Socialist Republic of Sri Lanka | 55–59 years | 0.70 (0.52, 0.88) |
| Democratic Socialist Republic of Sri Lanka | 60–64 years | 0.81 (0.66, 0.96) |
| Democratic Socialist Republic of Sri Lanka | 65–69 years | 0.91 (0.77, 1.04) |
| Democratic Socialist Republic of Sri Lanka | 70–74 years | 0.98 (0.85, 1.11) |
| Democratic Socialist Republic of Sri Lanka | 75–79 years | 1.03 (0.89, 1.16) |
| Democratic Socialist Republic of Sri Lanka | 80–84 years | 1.06 (0.89, 1.23) |
| Democratic Socialist Republic of Sri Lanka | 85–89 years | 1.14 (0.87, 1.41) |
| Democratic Socialist Republic of Sri Lanka | 90–94 years | 1.20 (0.67, 1.74) |
| Democratic Socialist Republic of Sri Lanka | 95+ years | 0.98 ( - 0.36, 2.33) |
| Dominican Republic | 30–34 years | 0.38 (-0.67, 1.44) |
| Dominican Republic | 35-39 years | 0.49 (-0.16, 1.14) |
| Dominican Republic | 40–44 years | 0.58 (0.11, 1.06) |
| Dominican Republic | 45–49 years | 0.70 (0.32, 1.08) |
| Dominican Republic | 50–54 years | 0.81 (0.49, 1.14) |
| Dominican Republic | 55–59 years | 0.93 (0.64, 1.22) |
| Dominican Republic | 60–64 years | 1.05 (0.78, 1.32) |
| Dominican Republic | 65–69 years | 1.12 (0.87, 1.38) |
| Dominican Republic | 70–74 years | 1.11 (0.86, 1.36) |
| Dominican Republic | 75–79 years | 1.00 (0.75, 1.26) |
| Dominican Republic | 80–84 years | 0.80 (0.50, 1.10) |
| Dominican Republic | 85–89 years | 0.52 (0.09, 0.95) |
| Dominican Republic | 90–94 years | 0.33 (-0.48, 1.15) |
| Dominican Republic | 95+ years | 0.23 (-1.58, 2.07) |
| Eastern Republic of Uruguay | 30–34 years | 0.56 (-2.11, 3.29) |
| Eastern Republic of Uruguay | 35–39 years | 0.73 (-0.87, 2.36) |
| Eastern Republic of Uruguay | 40–44 years | 0.89 (-0.21, 1.99) |
| Eastern Republic of Uruguay | 45–49 years | 0.98 (0.19, 1.77) |
| Eastern Republic of Uruguay | 50–54 years | 1.00 (0.44, 1.57) |
| Eastern Republic of Uruguay | 55–59 years | 1.02 (0.62, 1.43) |
| Eastern Republic of Uruguay | 60–64 years | 1.09 (0.78, 1.39) |
| Eastern Republic of Uruguay | 65–69 years | 1.19 (0.94, 1.43) |
| Eastern Republic of Uruguay | 70–74 years | 1.28 (1.07, 1.49) |
| Eastern Republic of Uruguay | 75–79 years | 1.32 (1.13, 1.52) |
| Eastern Republic of Uruguay | 80–84 years | 1.31 (1.09, 1.52) |
| Eastern Republic of Uruguay | 85–89 years | 1.25 (0.95, 1.54) |
| Eastern Republic of Uruguay | 90–94 years | 1.19 (0.67, 1.71) |
| Eastern Republic of Uruguay | 95+ years | 1.18 (0.04, 2.33) |
| Federal Democratic Republic of Ethiopia | 30–34 years | -0.34 (-0.78, 0.10) |
| Federal Democratic Republic of Ethiopia | 35–39 years | -0.39 (-0.69, -0.09) |
| Federal Democratic Republic of Ethiopia | 40–44 years | -0.41 (-0.64, -0.17) |
| Federal Democratic Republic of Ethiopia | 45–49 years | -0.40 (-0.60, -0.20) |
| Federal Democratic Republic of Ethiopia | 50–54 years | -0.32 (-0.49, -0.14) |
| Federal Democratic Republic of Ethiopia | 55–59 years | -0.17 (-0.32, -0.01) |
| Federal Democratic Republic of Ethiopia | 60–64 years | 0.02 (-0.12, 0.16) |
| Federal Democratic Republic of Ethiopia | 65–69 years | 0.23 (0.10, 0.36) |
| Federal Democratic Republic of Ethiopia | 70–74 years | 0.46 (0.33, 0.58) |
| Federal Democratic Republic of Ethiopia | 75–79 years | 0.67 (0.53, 0.81) |
| Federal Democratic Republic of Ethiopia | 80–84 years | 0.96 (0.75, 1.17) |
| Federal Democratic Republic of Ethiopia | 85–89 years | 1.30 (0.87, 1.73) |
| Federal Democratic Republic of Ethiopia | 90–94 years | 1.58 (0.53, 2.65) |
| Federal Democratic Republic of Ethiopia | 95+ years | 1.76 (-1.14, 4.74) |
| Federal Democratic Republic of Nepal | 30–34 years | 0.68 (-0.09, 1.45) |
| Federal Democratic Republic of Nepal | 35–39 years | 0.78 (0.30, 1.27) |
| Federal Democratic Republic of Nepal | 40–44 years | 0.89 (0.53, 1.25) |
| Federal Democratic Republic of Nepal | 45–49 years | 1.02 (0.73, 1.30) |
| Federal Democratic Republic of Nepal | 50–54 years | 1.16 (0.93, 1.40) |
| Federal Democratic Republic of Nepal | 55–59 years | 1.30 (1.10, 1.51) |
| Federal Democratic Republic of Nepal | 60–64 years | 1.42 (1.24, 1.60) |
| Federal Democratic Republic of Nepal | 65–69 years | 1.50 (1.34, 1.66) |
| Federal Democratic Republic of Nepal | 70–74 years | 1.60 (1.44, 1.75) |
| Federal Democratic Republic of Nepal | 75–79 years | 1.74 (1.56, 1.92) |
| Federal Democratic Republic of Nepal | 80–84 years | 1.96 (1.71, 2.22) |
| Federal Democratic Republic of Nepal | 85–89 years | 2.25 (1.82, 2.68) |
| Federal Democratic Republic of Nepal | 90–94 years | 2.46 (1.58, 3.35) |
| Federal Democratic Republic of Nepal | 95+ years | 2.44 (0.29, 4.65) |
| Federal Republic of Germany | 30–34 years | 0.36 (-0.52, 1.25) |
| Federal Republic of Germany | 35–39 years | 0.41 (-0.14, 0.97) |
| Federal Republic of Germany | 40–44 years | 0.54 (0.15, 0.94) |
| Federal Republic of Germany | 45–49 years | 0.66 (0.39, 0.94) |
| Federal Republic of Germany | 50–54 years | 0.72 (0.53, 0.91) |
| Federal Republic of Germany | 55–59 years | 0.77 (0.62, 0.91) |
| Federal Republic of Germany | 60–64 years | 0.93 (0.82, 1.04) |
| Federal Republic of Germany | 65–69 years | 1.28 (1.19, 1.37) |
| Federal Republic of Germany | 70–74 years | 1.80 (1.72, 1.88) |
| Federal Republic of Germany | 75–79 years | 2.28 (2.21, 2.35) |
| Federal Republic of Germany | 80–84 years | 2.61 (2.54, 2.68) |
| Federal Republic of Germany | 85–89 years | 2.71 (2.62, 2.81) |
| Federal Republic of Germany | 90–94 years | 2.64 (2.46, 2.81) |
| Federal Republic of Germany | 95+ years | 2.50 (2.09, 2.90) |
| Federal Republic of Nigeria | 30–34 years | 0.24 (-0.08, 0.57) |
| Federal Republic of Nigeria | 35–39 years | 0.24 (0.02, 0.46) |
| Federal Republic of Nigeria | 40–44 years | 0.23 (0.06, 0.40) |
| Federal Republic of Nigeria | 45–49 years | 0.22 (0.08, 0.36) |
| Federal Republic of Nigeria | 50–54 years | 0.26 (0.14, 0.39) |
| Federal Republic of Nigeria | 55–59 years | 0.36 (0.25, 0.47) |
| Federal Republic of Nigeria | 60–64 years | 0.51 (0.42, 0.61) |
| Federal Republic of Nigeria | 65–69 years | 0.71 (0.63, 0.80) |
| Federal Republic of Nigeria | 70–74 years | 0.92 (0.84, 1.00) |
| Federal Republic of Nigeria | 75–79 years | 1.04 (0.97, 1.12) |
| Federal Republic of Nigeria | 80–84 years | 1.09 (1.00, 1.18) |
| Federal Republic of Nigeria | 85–89 years | 1.11 (0.97, 1.25) |
| Federal Republic of Nigeria | 90–94 years | 1.14 (0.87, 1.42) |
| Federal Republic of Nigeria | 95+ years | 1.25 (0.62, 1.88) |
| Federal Republic of Somalia | 30–34 years | 0.07 (-1.06, 1.22) |
| Federal Republic of Somalia | 35–39 years | 0.11 (-0.64, 0.86) |
| Federal Republic of Somalia | 40–44 years | 0.06 (-0.52, 0.66) |
| Federal Republic of Somalia | 45–49 years | 0.00 (-0.52, 0.52) |
| Federal Republic of Somalia | 50–54 years | -0.02 (-0.50, 0.46) |
| Federal Republic of Somalia | 55–59 years | -0.01 (-0.43, 0.42) |
| Federal Republic of Somalia | 60–64 years | 0.03 (-0.37, 0.43) |
| Federal Republic of Somalia | 65–69 years | 0.10 (-0.30, 0.50) |
| Federal Republic of Somalia | 70–74 years | 0.16 (-0.29, 0.60) |
| Federal Republic of Somalia | 75–79 years | 0.17 (-0.38, 0.73) |
| Federal Republic of Somalia | 80–84 years | 0.18 (-0.57, 0.94) |
| Federal Republic of Somalia | 85–89 years | 0.23 (-1.02, 1.50) |
| Federal Republic of Somalia | 90–94 years | 0.33 (-2.22, 2.94) |
| Federal Republic of Somalia | 95+ years | 0.43 (-5.33, 6.55) |
| Federated States of Micronesia | 30–34 years | 0.11 (-12.94, 15.12) |
| Federated States of Micronesia | 35–39 years | 0.22 (-8.20, 9.40) |
| Federated States of Micronesia | 40–44 years | 0.37 (-5.57, 6.68) |
| Federated States of Micronesia | 45–49 years | 0.52 (-3.83, 5.07) |
| Federated States of Micronesia | 50–54 years | 0.62 (-2.82, 4.18) |
| Federated States of Micronesia | 55–59 years | 0.65 (-2.19, 3.57) |
| Federated States of Micronesia | 60–64 years | 0.62 (-1.84, 3.15) |
| Federated States of Micronesia | 65–69 years | 0.58 (-1.70, 2.92) |
| Federated States of Micronesia | 70–74 years | 0.54 (-1.72, 2.86) |
| Federated States of Micronesia | 75–79 years | 0.54 (-1.91, 3.06) |
| Federated States of Micronesia | 80–84 years | 0.62 (-2.41, 3.74) |
| Federated States of Micronesia | 85–89 years | 0.79 (-3.92, 5.73) |
| Federated States of Micronesia | 90–94 years | 1.05 (-8.41, 11.49) |
| Federated States of Micronesia | 95+ years | 1.30 (-21.05, 29.97) |
| Federative Republic of Brazil | 30–34 years | 0.71 (0.29, 1.13) |
| Federative Republic of Brazil | 35–39 years | 0.81 (0.57, 1.06) |
| Federative Republic of Brazil | 40–44 years | 0.90 (0.72, 1.08) |
| Federative Republic of Brazil | 45–49 years | 0.96 (0.82, 1.10) |
| Federative Republic of Brazil | 50–54 years | 0.95 (0.83, 1.06) |
| Federative Republic of Brazil | 55–59 years | 0.90 (0.79, 1.00) |
| Federative Republic of Brazil | 60–64 years | 0.84 (0.75, 0.93) |
| Federative Republic of Brazil | 65–69 years | 0.80 (0.72, 0.89) |
| Federative Republic of Brazil | 70–74 years | 0.77 (0.69, 0.85) |
| Federative Republic of Brazil | 75–79 years | 0.75 (0.66, 0.83) |
| Federative Republic of Brazil | 80–84 years | 0.70 (0.59, 0.81) |
| Federative Republic of Brazil | 85–89 years | 0.63 (0.47, 0.79) |
| Federative Republic of Brazil | 90–94 years | 0.58 (0.28, 0.88) |
| Federative Republic of Brazil | 95+ years | 0.57 (-0.12, 1.27) |
| French Republic | 30–34 years | 0.26 (-0.49, 1.03) |
| French Republic | 35–39 years | 0.36 (-0.10, 0.83) |
| French Republic | 40–44 years | 0.49 (0.17, 0.82) |
| French Republic | 45–49 years | 0.60 (0.37, 0.83) |
| French Republic | 50–54 years | 0.68 (0.51, 0.86) |
| French Republic | 55–59 years | 0.78 (0.64, 0.91) |
| French Republic | 60–64 years | 0.92 (0.82, 1.03) |
| French Republic | 65–69 years | 1.12 (1.04, 1.20) |
| French Republic | 70–74 years | 1.28 (1.21, 1.34) |
| French Republic | 75–79 years | 1.35 (1.29, 1.42) |
| French Republic | 80–84 years | 1.31 (1.24, 1.37) |
| French Republic | 85–89 years | 1.15 (1.07, 1.23) |
| French Republic | 90–94 years | 0.86 (0.72, 0.99) |
| French Republic | 95+ years | 0.48 (0.17, 0.80) |
| Gabonese Republic | 30–34 years | -0.07 (-3.27, 3.23) |
| Gabonese Republic | 35–39 years | -0.05 (-2.12, 2.06) |
| Gabonese Republic | 40–44 years | -0.03 (-1.57, 1.54) |
| Gabonese Republic | 45–49 years | 0.05 (-1.18, 1.29) |
| Gabonese Republic | 50–54 years | 0.20 (-0.81, 1.23) |
| Gabonese Republic | 55–59 years | 0.38 (-0.48, 1.25) |
| Gabonese Republic | 60–64 years | 0.53 (-0.21, 1.28) |
| Gabonese Republic | 65–69 years | 0.63 (-0.04, 1.30) |
| Gabonese Republic | 70–74 years | 0.64 (-0.01, 1.30) |
| Gabonese Republic | 75–79 years | 0.64 (-0.07, 1.35) |
| Gabonese Republic | 80–84 years | 0.68 (-0.23, 1.61) |
| Gabonese Republic | 85–89 years | 0.83 (-0.58, 2.25) |
| Gabonese Republic | 90–94 years | 1.10 (-1.70, 3.98) |
| Gabonese Republic | 95+ years | 1.34 ( - 5.40, 8.56) |
| Georgia | 30–34 years | -0.18 (-2.52, 2.22) |
| Georgia | 35–39 years | -0.13 (-1.52, 1.27) |
| Georgia | 40–44 years | -0.12 (-1.07, 0.83) |
| Georgia | 45–49 years | -0.18 (-0.86, 0.50) |
| Georgia | 50–54 years | -0.29 (-0.78, 0.20) |
| Georgia | 55–59 years | -0.41 (-0.76, -0.05) |
| Georgia | 60–64 years | -0.51 (-0.78, -0.24) |
| Georgia | 65–69 years | -0.57 (-0.78, -0.35) |
| Georgia | 70–74 years | -0.51 (-0.71, -0.31) |
| Georgia | 75–79 years | -0.40 (-0.60, -0.20) |
| Georgia | 80–84 years | -0.31 (-0.55, -0.08) |
| Georgia | 85–89 years | -0.33 (-0.66, -0.00) |
| Georgia | 90–94 years | -0.44 (-1.02, 0.14) |
| Georgia | 95+ years | -0.52 (-1.94, 0.93) |
| Grand Duchy of Luxembourg | 30–34 years | 0.27 (-3.69, 4.40) |
| Grand Duchy of Luxembourg | 35–39 years | 0.34 (-2.21, 2.95) |
| Grand Duchy of Luxembourg | 40–44 years | 0.38 (-1.47, 2.27) |
| Grand Duchy of Luxembourg | 45–49 years | 0.43 (-0.95, 1.82) |
| Grand Duchy of Luxembourg | 50–54 years | 0.50 (-0.55, 1.56) |
| Grand Duchy of Luxembourg | 55–59 years | 0.60 (-0.24, 1.44) |
| Grand Duchy of Luxembourg | 60–64 years | 0.75 (0.08, 1.42) |
| Grand Duchy of Luxembourg | 65–69 years | 0.96 (0.42, 1.50) |
| Grand Duchy of Luxembourg | 70–74 years | 1.15 (0.69, 1.61) |
| Grand Duchy of Luxembourg | 75–79 years | 1.23 (0.80, 1.67) |
| Grand Duchy of Luxembourg | 80–84 years | 1.25 (0.79, 1.71) |
| Grand Duchy of Luxembourg | 85–89 years | 1.24 (0.61, 1.87) |
| Grand Duchy of Luxembourg | 90–94 years | 1.20 (0.00, 2.42) |
| Grand Duchy of Luxembourg | 95+ years | 1.22 (-2.06, 4.62) |
| Greenland | 30–34 years | 0.08 (-19.37, 24.23) |
| Greenland | 35–39 years | 0.10 (-11.96, 13.81) |
| Greenland | 40–44 years | 0.15 (-8.16, 9.21) |
| Greenland | 45–49 years | 0.19 (-5.18, 5.86) |
| Greenland | 50–54 years | 0.30 (-3.30, 4.04) |
| Greenland | 55–59 years | 0.46 (-2.43, 3.43) |
| Greenland | 60–64 years | 0.64 (-1.85, 3.19) |
| Greenland | 65–69 years | 0.73 (-1.52, 3.04) |
| Greenland | 70–74 years | 0.76 (-1.50, 3.06) |
| Greenland | 75–79 years | 0.74 (-1.76, 3.31) |
| Greenland | 80–84 years | 0.78 (-2.59, 4.27) |
| Greenland | 85–89 years | 0.88 (-4.61, 6.68) |
| Greenland | 90–94 years | 1.02 (-10.62, 14.18) |
| Greenland | 95+ years | 1.06 (-35.59, 58.56) |
| Grenada | 30–34 years | 0.06 (-10.35, 11.68) |
| Grenada | 35–39 years | 0.18 (-6.29, 7.09) |
| Grenada | 40–44 years | 0.29 (-4.20, 4.99) |
| Grenada | 45–49 years | 0.42 (-2.93, 3.89) |
| Grenada | 50–54 years | 0.53 (-2.21, 3.36) |
| Grenada | 55–59 years | 0.68 (-1.77, 3.19) |
| Grenada | 60–64 years | 0.86 (-1.43, 3.20) |
| Grenada | 65–69 years | 0.98 (-1.16, 3.17) |
| Grenada | 70–74 years | 1.04 (-1.02, 3.14) |
| Grenada | 75–79 years | 1.01 (-1.12, 3.19) |
| Grenada | 80–84 years | 0.89 (-1.60, 3.45) |
| Grenada | 85–89 years | 0.75 (-2.45, 4.06) |
| Grenada | 90–94 years | 0.71 (-4.01, 5.67) |
| Grenada | 95+ years | 0.61 (-7.38, 9.28) |
| Guam | 30–34 years | 0.51 (-9.68, 11.84) |
| Guam | 35–39 years | 0.50 (-5.81, 7.24) |
| Guam | 40–44 years | 0.54 (-3.78, 5.05) |
| Guam | 45–49 years | 0.57 (-2.44, 3.67) |
| Guam | 50–54 years | 0.50 (-1.77, 2.83) |
| Guam | 55–59 years | 0.30 (-1.52, 2.15) |
| Guam | 60–64 years | 0.01 (-1.51, 1.57) |
| Guam | 65–69 years | -0.30 (-1.65, 1.07) |
| Guam | 70–74 years | -0.39 (-1.68, 0.92) |
| Guam | 75–79 years | -0.24 (-1.63, 1.18) |
| Guam | 80–84 years | -0.00 (-1.63, 1.65) |
| Guam | 85–89 years | 0.20 (-2.12, 2.58) |
| Guam | 90–94 years | 0.14 (-4.64, 5.15) |
| Guam | 95+ years | -0.45 (-14.12, 15.40) |
| Hashemite Kingdom of Jordan | 30–34 years | 0.31 (-1.34, 1.99) |
| Hashemite Kingdom of Jordan | 35–39 years | 0.40 (-0.65, 1.46) |
| Hashemite Kingdom of Jordan | 40–44 years | 0.52 (-0.27, 1.31) |
| Hashemite Kingdom of Jordan | 45–49 years | 0.64 (0.01, 1.28) |
| Hashemite Kingdom of Jordan | 50–54 years | 0.73 (0.20, 1.27) |
| Hashemite Kingdom of Jordan | 55–59 years | 0.80 (0.33, 1.27) |
| Hashemite Kingdom of Jordan | 60–64 years | 0.87 (0.43, 1.30) |
| Hashemite Kingdom of Jordan | 65–69 years | 0.92 (0.51, 1.33) |
| Hashemite Kingdom of Jordan | 70–74 years | 1.01 (0.61, 1.42) |
| Hashemite Kingdom of Jordan | 75–79 years | 1.18 (0.74, 1.63) |
| Hashemite Kingdom of Jordan | 80–84 years | 1.33 (0.77, 1.90) |
| Hashemite Kingdom of Jordan | 85–89 years | 1.49 (0.67, 2.32) |
| Hashemite Kingdom of Jordan | 90–94 years | 1.61 (0.22, 3.01) |
| Hashemite Kingdom of Jordan | 95+ years | 1.72 (-1.22, 4.75) |
| Hellenic Republic | 30–34 years | 0.21 (-0.87, 1.31) |
| Hellenic Republic | 35–39 years | 0.30 (-0.33, 0.93) |
| Hellenic Republic | 40–44 years | 0.40 (-0.04, 0.84) |
| Hellenic Republic | 45–49 years | 0.48 (0.16, 0.80) |
| Hellenic Republic | 50–54 years | 0.52 (0.28, 0.76) |
| Hellenic Republic | 55–59 years | 0.54 (0.35, 0.72) |
| Hellenic Republic | 60–64 years | 0.54 (0.40, 0.69) |
| Hellenic Republic | 65–69 years | 0.55 (0.44, 0.66) |
| Hellenic Republic | 70–74 years | 0.57 (0.48, 0.66) |
| Hellenic Republic | 75–79 years | 0.64 (0.55, 0.72) |
| Hellenic Republic | 80–84 years | 0.73 (0.64, 0.82) |
| Hellenic Republic | 85–89 years | 0.82 (0.69, 0.94) |
| Hellenic Republic | 90–94 years | 0.88 (0.64, 1.11) |
| Hellenic Republic | 95+ years | 0.86 (0.24, 1.49) |
| Hungary | 30–34 years | 0.01 (-1.77, 1.83) |
| Hungary | 35–39 years | 0.04 (-0.97, 1.06) |
| Hungary | 40–44 years | 0.08 (-0.55, 0.72) |
| Hungary | 45–49 years | 0.11 (-0.34, 0.56) |
| Hungary | 50–54 years | 0.14 (-0.19, 0.47) |
| Hungary | 55–59 years | 0.21 (-0.03, 0.45) |
| Hungary | 60–64 years | 0.34 (0.17, 0.51) |
| Hungary | 65–69 years | 0.51 (0.38, 0.64) |
| Hungary | 70–74 years | 0.63 (0.52, 0.74) |
| Hungary | 75–79 years | 0.64 (0.52, 0.76) |
| Hungary | 80–84 years | 0.58 (0.44, 0.72) |
| Hungary | 85–89 years | 0.51 (0.29, 0.72) |
| Hungary | 90–94 years | 0.42 (-0.04, 0.89) |
| Hungary | 95+ years | 0.43 (-1.02, 1.89) |
| Independent State of Papua New Guinea | 30–34 years | 0.43 (-1.09, 1.97) |
| Independent State of Papua New Guinea | 35–39 years | 0.45 (-0.57, 1.48) |
| Independent State of Papua New Guinea | 40–44 years | 0.46 (-0.30, 1.24) |
| Independent State of Papua New Guinea | 45–49 years | 0.47 (-0.13, 1.08) |
| Independent State of Papua New Guinea | 50–54 years | 0.48 (-0.02, 0.98) |
| Independent State of Papua New Guinea | 55–59 years | 0.45 (0.02, 0.88) |
| Independent State of Papua New Guinea | 60-64 years | 0.38 (0.00, 0.77) |
| Independent State of Papua New Guinea | 65–69 years | 0.33 (-0.02, 0.69) |
| Independent State of Papua New Guinea | 70–74 years | 0.33 (-0.02, 0.68) |
| Independent State of Papua New Guinea | 75–79 years | 0.39 (-0.02, 0.79) |
| Independent State of Papua New Guinea | 80–84 years | 0.50 (-0.07, 1.09) |
| Independent State of Papua New Guinea | 85–89 years | 0.62 (-0.39, 1.64) |
| Independent State of Papua New Guinea | 90–94 years | 0.74 (-1.42, 2.94) |
| Independent State of Papua New Guinea | 95+ years | 0.91 (-4.19, 6.28) |
| Independent State of Samoa | 30–34 years | 0.34 (-9.29, 10.99) |
| Independent State of Samoa | 35–39 years | 0.42 (-5.91, 7.17) |
| Independent State of Samoa | 40–44 years | 0.46 (-4.10, 5.24) |
| Independent State of Samoa | 45–49 years | 0.48 (-2.90, 3.99) |
| Independent State of Samoa | 50–54 years | 0.48 (-2.15, 3.18) |
| Independent State of Samoa | 55–59 years | 0.44 (-1.68, 2.61) |
| Independent State of Samoa | 60–64 years | 0.38 (-1.42, 2.20) |
| Independent State of Samoa | 65–69 years | 0.32 (-1.25, 1.92) |
| Independent State of Samoa | 70–74 years | 0.32 (-1.16, 1.81) |
| Independent State of Samoa | 75–79 years | 0.41 (-1.15, 1.99) |
| Independent State of Samoa | 80–84 years | 0.63 (-1.40, 2.69) |
| Independent State of Samoa | 85–89 years | 0.94 (-2.21, 4.19) |
| Independent State of Samoa | 90–94 years | 1.27 (-4.40, 7.28) |
| Independent State of Samoa | 95+ years | 1.52 (-11.00, 15.80) |
| Ireland | 30–34 years | 0.42 (-1.09, 1.94) |
| Ireland | 35–39 years | 0.52 (-0.39, 1.43) |
| Ireland | 40–44 years | 0.61 (-0.04, 1.27) |
| Ireland | 45–49 years | 0.68 (0.19, 1.17) |
| Ireland | 50-54 years | 0.74 (0.36, 1.12) |
| Ireland | 55–59 years | 0.84 (0.54, 1.15) |
| Ireland | 60–64 years | 1.00 (0.76, 1.25) |
| Ireland | 65–69 years | 1.20 (1.00, 1.40) |
| Ireland | 70–74 years | 1.33 (1.16, 1.50) |
| Ireland | 75–79 years | 1.38 (1.22, 1.54) |
| Ireland | 80–84 years | 1.40 (1.23, 1.58) |
| Ireland | 85–89 years | 1.46 (1.21, 1.72) |
| Ireland | 90–94 years | 1.55 (1.06, 2.04) |
| Ireland | 95+ years | 1.53 (0.26, 2.81) |
| Islamic Republic of Afghanistan | 30–34 years | 0.31 (-0.66, 1.29) |
| Islamic Republic of Afghanistan | 35–39 years | 0.41 (-0.23, 1.06) |
| Islamic Republic of Afghanistan | 40–44 years | 0.58 (0.12, 1.04) |
| Islamic Republic of Afghanistan | 45–49 years | 0.73 (0.37, 1.08) |
| Islamic Republic of Afghanistan | 50–54 years | 0.84 (0.52, 1.15) |
| Islamic Republic of Afghanistan | 55–59 years | 0.89 (0.59, 1.19) |
| Islamic Republic of Afghanistan | 60–64 years | 0.90 (0.63, 1.16) |
| Islamic Republic of Afghanistan | 65–69 years | 0.92 (0.70, 1.14) |
| Islamic Republic of Afghanistan | 70–74 years | 0.96 (0.76, 1.17) |
| Islamic Republic of Afghanistan | 75–79 years | 1.02 (0.80, 1.25) |
| Islamic Republic of Afghanistan | 80–84 years | 1.11 (0.81, 1.41) |
| Islamic Republic of Afghanistan | 85–89 years | 1.22 (0.72, 1.71) |
| Islamic Republic of Afghanistan | 90–94 years | 1.32 (0.32, 2.34) |
| Islamic Republic of Afghanistan | 95+ years | 1.42 (-1.29, 4.20) |
| Islamic Republic of Iran | 30–34 years | 0.38 (-0.02, 0.79) |
| Islamic Republic of Iran | 35–39 years | 0.58 (0.33, 0.83) |
| Islamic Republic of Iran | 40–44 years | 0.74 (0.55, 0.93) |
| Islamic Republic of Iran | 45–49 years | 0.88 (0.72, 1.03) |
| Islamic Republic of Iran | 50–54 years | 0.97 (0.85, 1.10) |
| Islamic Republic of Iran | 55–59 years | 1.01 (0.90, 1.12) |
| Islamic Republic of Iran | 60–64 years | 1.00 (0.91, 1.10) |
| Islamic Republic of Iran | 65–69 years | 0.99 (0.90, 1.07) |
| Islamic Republic of Iran | 70–74 years | 0.99 (0.91, 1.07) |
| Islamic Republic of Iran | 75–79 years | 1.09 (1.01, 1.18) |
| Islamic Republic of Iran | 80–84 years | 1.37 (1.24, 1.49) |
| Islamic Republic of Iran | 85–89 years | 1.72 (1.50, 1.94) |
| Islamic Republic of Iran | 90–94 years | 1.93 (1.61, 2.26) |
| Islamic Republic of Iran | 95+ years | 2.07 (1.41, 2.73) |
| Islamic Republic of Mauritania | 30–34 years | 0.05 (-2.36, 2.51) |
| Islamic Republic of Mauritania | 35–39 years | -0.01 (-1.63, 1.64) |
| Islamic Republic of Mauritania | 40–44 years | -0.02 (-1.28, 1.25) |
| Islamic Republic of Mauritania | 45–49 years | 0.00 (-1.04, 1.05) |
| Islamic Republic of Mauritania | 50–54 years | 0.04 (-0.85, 0.93) |
| Islamic Republic of Mauritania | 55–59 years | 0.08 (-0.67, 0.84) |
| Islamic Republic of Mauritania | 60–64 years | 0.15 (-0.48, 0.80) |
| Islamic Republic of Mauritania | 65–69 years | 0.30 (-0.25, 0.85) |
| Islamic Republic of Mauritania | 70–74 years | 0.48 (-0.01, 0.98) |
| Islamic Republic of Mauritania | 75–79 years | 0.66 (0.19, 1.13) |
| Islamic Republic of Mauritania | 80–84 years | 0.78 (0.19, 1.37) |
| Islamic Republic of Mauritania | 85–89 years | 0.86 (-0.09, 1.81) |
| Islamic Republic of Mauritania | 90–94 years | 0.93 (-0.93, 2.82) |
| Islamic Republic of Mauritania | 95+ years | 1.11 (-3.87, 6.35) |
| Islamic Republic of Pakistan | 30–34 years | 0.33 (0.06, 0.60) |
| Islamic Republic of Pakistan | 35–39 years | 0.44 (0.27, 0.62) |
| Islamic Republic of Pakistan | 40–44 years | 0.54 (0.41, 0.68) |
| Islamic Republic of Pakistan | 45–49 years | 0.63 (0.52, 0.74) |
| Islamic Republic of Pakistan | 50–54 years | 0.70 (0.61, 0.79) |
| Islamic Republic of Pakistan | 55–59 years | 0.75 (0.67, 0.83) |
| Islamic Republic of Pakistan | 60–64 years | 0.78 (0.70, 0.85) |
| Islamic Republic of Pakistan | 65–69 years | 0.80 (0.74, 0.87) |
| Islamic Republic of Pakistan | 70–74 years | 0.87 (0.81, 0.93) |
| Islamic Republic of Pakistan | 75–79 years | 1.00 (0.93, 1.06) |
| Islamic Republic of Pakistan | 80–84 years | 1.20 (1.11, 1.28) |
| Islamic Republic of Pakistan | 85–89 years | 1.45 (1.31, 1.58) |
| Islamic Republic of Pakistan | 90–94 years | 1.60 (1.31, 1.88) |
| Islamic Republic of Pakistan | 95+ years | 1.52 (0.81, 2.22) |
| Jamaica | 30–34 years | 0.42 (-1.59, 2.47) |
| Jamaica | 35–39 years | 0.46 (-0.77, 1.71) |
| Jamaica | 40–44 years | 0.47 (-0.41, 1.36) |
| Jamaica | 45–49 years | 0.50 (-0.17, 1.19) |
| Jamaica | 50–54 years | 0.55 (-0.01, 1.12) |
| Jamaica | 55–59 years | 0.60 (0.11, 1.09) |
| Jamaica | 60–64 years | 0.65 (0.21, 1.10) |
| Jamaica | 65–69 years | 0.70 (0.29, 1.11) |
| Jamaica | 70–74 years | 0.71 (0.32, 1.09) |
| Jamaica | 75–79 years | 0.65 (0.27, 1.04) |
| Jamaica | 80–84 years | 0.59 (0.17, 1.01) |
| Jamaica | 85–89 years | 0.47 (-0.05, 0.99) |
| Jamaica | 90–94 years | 0.44 (-0.34, 1.24) |
| Jamaica | 95+ years | 0.42 (-0.97, 1.83) |
| Japan | 30–34 years | 0.45 (-0.59, 1.50) |
| Japan | 35–39 years | 0.47 (-0.12, 1.05) |
| Japan | 40–44 years | 0.43 (0.05, 0.81) |
| Japan | 45–49 years | 0.33 (0.07, 0.60) |
| Japan | 50–54 years | 0.17 (-0.03, 0.38) |
| Japan | 55–59 years | -0.00 (-0.16, 0.15) |
| Japan | 60–64 years | -0.12 (-0.24, 0.00) |
| Japan | 65–69 years | -0.13 (-0.23, -0.04) |
| Japan | 70–74 years | -0.03 (-0.11, 0.06) |
| Japan | 75–79 years | 0.18 (0.10, 0.27) |
| Japan | 80–84 years | 0.40 (0.31, 0.50) |
| Japan | 85–89 years | 0.61 (0.47, 0.76) |
| Japan | 90–94 years | 0.76 (0.48, 1.04) |
| Japan | 95+ years | 0.80 (0.10, 1.50) |
| Kingdom of Bahrain | 30–34 years | 0.47 (-2.69, 3.73) |
| Kingdom of Bahrain | 35–39 years | 0.60 (-1.35, 2.58) |
| Kingdom of Bahrain | 40–44 years | 0.59 (-0.89, 2.10) |
| Kingdom of Bahrain | 45–49 years | 0.57 (-0.71, 1.86) |
| Kingdom of Bahrain | 50–54 years | 0.66 (-0.51, 1.84) |
| Kingdom of Bahrain | 55–59 years | 0.80 (-0.26, 1.88) |
| Kingdom of Bahrain | 60–64 years | 0.98 (-0.01, 1.99) |
| Kingdom of Bahrain | 65–69 years | 1.17 (0.20, 2.15) |
| Kingdom of Bahrain | 70–74 years | 1.27 (0.26, 2.29) |
| Kingdom of Bahrain | 75–79 years | 1.34 (0.17, 2.53) |
| Kingdom of Bahrain | 80–84 years | 1.58 (-0.10, 3.28) |
| Kingdom of Bahrain | 85–89 years | 1.87 (-1.33, 5.17) |
| Kingdom of Bahrain | 90–94 years | 2.12 (-6.64, 11.70) |
| Kingdom of Bahrain | 95+ years | 2.34 (-23.39, 36.71) |
| Kingdom of Belgium | 30–34 years | 0.38 (-0.62, 1.38) |
| Kingdom of Belgium | 35–39 years | 0.47 (-0.15, 1.09) |
| Kingdom of Belgium | 40–44 years | 0.55 (0.12, 0.99) |
| Kingdom of Belgium | 45–49 years | 0.62 (0.31, 0.94) |
| Kingdom of Belgium | 50–54 years | 0.67 (0.44, 0.91) |
| Kingdom of Belgium | 55–59 years | 0.75 (0.57, 0.93) |
| Kingdom of Belgium | 60–64 years | 0.87 (0.73, 1.01) |
| Kingdom of Belgium | 65–69 years | 1.02 (0.91, 1.13) |
| Kingdom of Belgium | 70–74 years | 1.13 (1.04, 1.22) |
| Kingdom of Belgium | 75–79 years | 1.19 (1.10, 1.28) |
| Kingdom of Belgium | 80–84 years | 1.24 (1.15, 1.34) |
| Kingdom of Belgium | 85–89 years | 1.27 (1.15, 1.40) |
| Kingdom of Belgium | 90–94 years | 1.25 (1.03, 1.46) |
| Kingdom of Belgium | 95+ years | 1.10 (0.58, 1.62) |
| Kingdom of Bhutan | 30–34 years | 0.21 (-3.82, 4.40) |
| Kingdom of Bhutan | 35–39 years | 0.36 (-2.26, 3.05) |
| Kingdom of Bhutan | 40–44 years | 0.54 (-1.47, 2.58) |
| Kingdom of Bhutan | 45–49 years | 0.72 (-0.92, 2.38) |
| Kingdom of Bhutan | 50–54 years | 0.88 (-0.49, 2.28) |
| Kingdom of Bhutan | 55–59 years | 1.04 (-0.14, 2.23) |
| Kingdom of Bhutan | 60–64 years | 1.18 (0.15, 2.21) |
| Kingdom of Bhutan | 65–69 years | 1.33 (0.41, 2.25) |
| Kingdom of Bhutan | 70–74 years | 1.51 (0.62, 2.40) |
| Kingdom of Bhutan | 75–79 years | 1.74 (0.74, 2.75) |
| Kingdom of Bhutan | 80–84 years | 2.05 (0.60, 3.53) |
| Kingdom of Bhutan | 85–89 years | 2.31 (-0.15, 4.82) |
| Kingdom of Bhutan | 90–94 years | 2.37 (-2.63, 7.63) |
| Kingdom of Bhutan | 95+ years | 2.24 (-9.67, 15.73) |
| Kingdom of Cambodia | 30–34 years | 0.24 (-0.74, 1.22) |
| Kingdom of Cambodia | 35–39 years | 0.27 (-0.39, 0.93) |
| Kingdom of Cambodia | 40–44 years | 0.33 (-0.19, 0.84) |
| Kingdom of Cambodia | 45–49 years | 0.39 (-0.02, 0.80) |
| Kingdom of Cambodia | 50–54 years | 0.50 (0.17, 0.84) |
| Kingdom of Cambodia | 55–59 years | 0.65 (0.37, 0.93) |
| Kingdom of Cambodia | 60–64 years | 0.80 (0.54, 1.05) |
| Kingdom of Cambodia | 65–69 years | 0.96 (0.73, 1.19) |
| Kingdom of Cambodia | 70–74 years | 1.10 (0.87, 1.33) |
| Kingdom of Cambodia | 75–79 years | 1.20 (0.94, 1.47) |
| Kingdom of Cambodia | 80–84 years | 1.29 (0.92, 1.66) |
| Kingdom of Cambodia | 85–89 years | 1.36 (0.70, 2.02) |
| Kingdom of Cambodia | 90–94 years | 1.39 (0.04, 2.75) |
| Kingdom of Cambodia | 95+ years | 1.26 (-1.58, 4.19) |
| Kingdom of Denmark | 30–34 years | 0.57 (-0.85, 2.02) |
| Kingdom of Denmark | 35–39 years | 0.62 (-0.29, 1.53) |
| Kingdom of Denmark | 40–44 years | 0.68 (0.07, 1.31) |
| Kingdom of Denmark | 45–49 years | 0.77 (0.33, 1.22) |
| Kingdom of Denmark | 50–54 years | 0.92 (0.59, 1.25) |
| Kingdom of Denmark | 55–59 years | 1.12 (0.86, 1.38) |
| Kingdom of Denmark | 60–64 years | 1.39 (1.18, 1.60) |
| Kingdom of Denmark | 65–69 years | 1.68 (1.52, 1.85) |
| Kingdom of Denmark | 70–74 years | 1.92 (1.78, 2.05) |
| Kingdom of Denmark | 75–79 years | 2.05 (1.92, 2.19) |
| Kingdom of Denmark | 80–84 years | 2.13 (1.99, 2.28) |
| Kingdom of Denmark | 85–89 years | 2.16 (1.96, 2.35) |
| Kingdom of Denmark | 90–94 years | 2.08 (1.76, 2.40) |
| Kingdom of Denmark | 95+ years | 1.87 (1.19, 2.56) |
| Kingdom of Eswatini | 30–34 years | 0.23 (-3.40, 3.99) |
| Kingdom of Eswatini | 35–39 years | 0.37 (-2.07, 2.88) |
| Kingdom of Eswatini | 40–44 years | 0.42 (-1.49, 2.37) |
| Kingdom of Eswatini | 45–49 years | 0.39 (-1.21, 2.01) |
| Kingdom of Eswatini | 50–54 years | 0.32 (-1.07, 1.73) |
| Kingdom of Eswatini | 55–59 years | 0.30 (-0.94, 1.55) |
| Kingdom of Eswatini | 60–64 years | 0.32 (-0.81, 1.45) |
| Kingdom of Eswatini | 65–69 years | 0.35 (-0.68, 1.40) |
| Kingdom of Eswatini | 70–74 years | 0.44 (-0.57, 1.47) |
| Kingdom of Eswatini | 75–79 years | 0.60 (-0.53, 1.75) |
| Kingdom of Eswatini | 80–84 years | 0.77 (-0.74, 2.31) |
| Kingdom of Eswatini | 85–89 years | 0.93 (-1.54, 3.46) |
| Kingdom of Eswatini | 90–94 years | 1.02 (-3.75, 6.03) |
| Kingdom of Eswatini | 95+ years | 0.95 (-9.94, 13.16) |
| Kingdom of Lesotho | 30–34 years | 0.20 (-2.82, 3.32) |
| Kingdom of Lesotho | 35–39 years | 0.31 (-1.69, 2.34) |
| Kingdom of Lesotho | 40–44 years | 0.33 (-1.21, 1.90) |
| Kingdom of Lesotho | 45–49 years | 0.32 (-0.94, 1.60) |
| Kingdom of Lesotho | 50–54 years | 0.34 (-0.71, 1.40) |
| Kingdom of Lesotho | 55–59 years | 0.40 (-0.49, 1.31) |
| Kingdom of Lesotho | 60–64 years | 0.49 (-0.29, 1.29) |
| Kingdom of Lesotho | 65–69 years | 0.58 (-0.12, 1.29) |
| Kingdom of Lesotho | 70–74 years | 0.63 (-0.03, 1.30) |
| Kingdom of Lesotho | 75–79 years | 0.68 (-0.02, 1.39) |
| Kingdom of Lesotho | 80–84 years | 0.77 (-0.11, 1.66) |
| Kingdom of Lesotho | 85–89 years | 0.86 (-0.50, 2.23) |
| Kingdom of Lesotho | 90–94 years | 0.85 (-1.53, 3.28) |
| Kingdom of Lesotho | 95+ years | 0.69 (-4.35, 5.98) |
| Kingdom of Morocco | 30–34 years | 0.86 (0.16, 1.56) |
| Kingdom of Morocco | 35–39 years | 1.08 (0.66, 1.49) |
| Kingdom of Morocco | 40–44 years | 1.28 (0.99, 1.58) |
| Kingdom of Morocco | 45–49 years | 1.45 (1.23, 1.68) |
| Kingdom of Morocco | 50–54 years | 1.59 (1.40, 1.77) |
| Kingdom of Morocco | 55–59 years | 1.67 (1.52, 1.83) |
| Kingdom of Morocco | 60–64 years | 1.74 (1.60, 1.88) |
| Kingdom of Morocco | 65–69 years | 1.79 (1.67, 1.92) |
| Kingdom of Morocco | 70–74 years | 1.82 (1.70, 1.94) |
| Kingdom of Morocco | 75–79 years | 1.81 (1.68, 1.93) |
| Kingdom of Morocco | 80–84 years | 1.87 (1.71, 2.02) |
| Kingdom of Morocco | 85–89 years | 1.97 (1.74, 2.20) |
| Kingdom of Morocco | 90–94 years | 1.99 (1.57, 2.42) |
| Kingdom of Morocco | 95+ years | 1.90 (0.97, 2.84) |
| Kingdom of Norway | 30–34 years | 1.20 (-0.32, 2.75) |
| Kingdom of Norway | 35–39 years | 1.64 (0.65, 2.64) |
| Kingdom of Norway | 40–44 years | 2.19 (1.46, 2.92) |
| Kingdom of Norway | 45–49 years | 2.90 (2.34, 3.46) |
| Kingdom of Norway | 50–54 years | 3.62 (3.17, 4.09) |
| Kingdom of Norway | 55–59 years | 4.32 (3.93, 4.70) |
| Kingdom of Norway | 60–64 years | 4.94 (4.61, 5.27) |
| Kingdom of Norway | 65–69 years | 5.44 (5.16, 5.72) |
| Kingdom of Norway | 70–74 years | 5.78 (5.53, 6.02) |
| Kingdom of Norway | 75–79 years | 5.95 (5.71, 6.19) |
| Kingdom of Norway | 80–84 years | 6.02 (5.75, 6.29) |
| Kingdom of Norway | 85–89 years | 5.95 (5.58, 6.32) |
| Kingdom of Norway | 90–94 years | 5.65 (5.05, 6.26) |
| Kingdom of Norway | 95+ years | 5.11 (3.96, 6.27) |
| Kingdom of Saudi Arabia | 30–34 years | 0.81 (0.19, 1.43) |
| Kingdom of Saudi Arabia | 35–39 years | 1.04 (0.65, 1.43) |
| Kingdom of Saudi Arabia | 40–44 years | 1.27 (0.98, 1.57) |
| Kingdom of Saudi Arabia | 45–49 years | 1.48 (1.23, 1.74) |
| Kingdom of Saudi Arabia | 50–54 years | 1.65 (1.42, 1.88) |
| Kingdom of Saudi Arabia | 55–59 years | 1.75 (1.54, 1.96) |
| Kingdom of Saudi Arabia | 60–64 years | 1.79 (1.60, 1.99) |
| Kingdom of Saudi Arabia | 65–69 years | 1.72 (1.54, 1.91) |
| Kingdom of Saudi Arabia | 70–74 years | 1.57 (1.39, 1.75) |
| Kingdom of Saudi Arabia | 75–79 years | 1.47 (1.29, 1.66) |
| Kingdom of Saudi Arabia | 80–84 years | 1.50 (1.27, 1.72) |
| Kingdom of Saudi Arabia | 85–89 years | 1.65 (1.31, 2.00) |
| Kingdom of Saudi Arabia | 90–94 years | 1.93 (1.26, 2.59) |
| Kingdom of Saudi Arabia | 95+ years | 2.17 (0.63, 3.73) |
| Kingdom of Spain | 30–34 years | 1.05 ( - 0.39, 2.51) |
| Kingdom of Spain | 35–39 years | 1.12 (0.28, 1.96) |
| Kingdom of Spain | 40–44 years | 1.17 (0.60, 1.75) |
| Kingdom of Spain | 45–49 years | 1.20 (0.77, 1.63) |
| Kingdom of Spain | 50–54 years | 1.13 (0.80, 1.45) |
| Kingdom of Spain | 55–59 years | 0.95 (0.70, 1.19) |
| Kingdom of Spain | 60–64 years | 0.70 (0.52, 0.89) |
| Kingdom of Spain | 65–69 years | 0.47 (0.33, 0.62) |
| Kingdom of Spain | 70–74 years | 0.31 (0.20, 0.42) |
| Kingdom of Spain | 75–79 years | 0.23 (0.13, 0.34) |
| Kingdom of Spain | 80–84 years | 0.22 (0.11, 0.32) |
| Kingdom of Spain | 85–89 years | 0.25 (0.10, 0.39) |
| Kingdom of Spain | 90–94 years | 0.31 (0.05, 0.56) |
| Kingdom of Spain | 95+ years | 0.35 (-0.26, 0.96) |
| Kingdom of Sweden | 30–34 years | -0.13 (-1.18, 0.93) |
| Kingdom of Sweden | 35–39 years | 0.00 (-0.67, 0.69) |
| Kingdom of Sweden | 40–44 years | 0.12 (-0.36, 0.61) |
| Kingdom of Sweden | 45–49 years | 0.22 (-0.13, 0.57) |
| Kingdom of Sweden | 50–54 years | 0.34 (0.08, 0.61) |
| Kingdom of Sweden | 55–59 years | 0.52 (0.31, 0.73) |
| Kingdom of Sweden | 60–64 years | 0.82 (0.66, 0.99) |
| Kingdom of Sweden | 65–69 years | 1.23 (1.10, 1.36) |
| Kingdom of Sweden | 70–74 years | 1.64 (1.54, 1.75) |
| Kingdom of Sweden | 75–79 years | 2.03 (1.93, 2.13) |
| Kingdom of Sweden | 80–84 years | 2.35 (2.24, 2.46) |
| Kingdom of Sweden | 85–89 years | 2.53 (2.38, 2.68) |
| Kingdom of Sweden | 90–94 years | 2.50 (2.24, 2.75) |
| Kingdom of Sweden | 95+ years | 2.19 (1.64, 2.74) |
| Kingdom of Thailand | 30–34 years | 0.28 (-0.19, 0.75) |
| Kingdom of Thailand | 35–39 years | 0.33 (0.06, 0.60) |
| Kingdom of Thailand | 40–44 years | 0.34 (0.15, 0.53) |
| Kingdom of Thailand | 45–49 years | 0.36 (0.22, 0.50) |
| Kingdom of Thailand | 50–54 years | 0.43 (0.32, 0.54) |
| Kingdom of Thailand | 55–59 years | 0.53 (0.44, 0.62) |
| Kingdom of Thailand | 60–64 years | 0.61 (0.53, 0.69) |
| Kingdom of Thailand | 65–69 years | 0.65 (0.58, 0.72) |
| Kingdom of Thailand | 70–74 years | 0.65 (0.58, 0.72) |
| Kingdom of Thailand | 75–79 years | 0.64 (0.57, 0.71) |
| Kingdom of Thailand | 80–84 years | 0.69 (0.60, 0.77) |
| Kingdom of Thailand | 85–89 years | 0.83 (0.71, 0.96) |
| Kingdom of Thailand | 90–94 years | 1.13 (0.90, 1.36) |
| Kingdom of Thailand | 95+ years | 1.50 (1.02, 1.97) |
| Kingdom of the Netherlands | 30–34 years | 0.46 (-0.81, 1.75) |
| Kingdom of the Netherlands | 35–39 years | 0.56 (-0.24, 1.37) |
| Kingdom of the Netherlands | 40–44 years | 0.56 (0.01, 1.12) |
| Kingdom of the Netherlands | 45–49 years | 0.49 (0.11, 0.87) |
| Kingdom of the Netherlands | 50–54 years | 0.40 (0.12, 0.68) |
| Kingdom of the Netherlands | 55–59 years | 0.26 (0.05, 0.48) |
| Kingdom of the Netherlands | 60–64 years | 0.10 (-0.07, 0.26) |
| Kingdom of the Netherlands | 65–69 years | -0.10 (-0.23, 0.02) |
| Kingdom of the Netherlands | 70–74 years | -0.34 (-0.45, -0.24) |
| Kingdom of the Netherlands | 75–79 years | -0.58 (-0.67, -0.48) |
| Kingdom of the Netherlands | 80–84 years | -0.75 (-0.85, -0.65) |
| Kingdom of the Netherlands | 85–89 years | -0.85 (-0.98, -0.72) |
| Kingdom of the Netherlands | 90–94 years | -0.88 (-1.09, -0.66) |
| Kingdom of the Netherlands | 95+ years | -0.90 (-1.37, -0.42) |
| Kingdom of Tonga | 30–34 years | 0.24 (-13.60, 16.30) |
| Kingdom of Tonga | 35–39 years | 0.34 (-8.55, 10.09) |
| Kingdom of Tonga | 40–44 years | 0.42 (-5.99, 7.26) |
| Kingdom of Tonga | 45–49 years | 0.46 (-4.27, 5.43) |
| Kingdom of Tonga | 50–54 years | 0.48 (-3.16, 4.25) |
| Kingdom of Tonga | 55–59 years | 0.46 (-2.46, 3.47) |
| Kingdom of Tonga | 60–64 years | 0.42 (-2.02, 2.91) |
| Kingdom of Tonga | 65–69 years | 0.39 (-1.72, 2.54) |
| Kingdom of Tonga | 70–74 years | 0.39 (-1.55, 2.37) |
| Kingdom of Tonga | 75–79 years | 0.43 (-1.52, 2.42) |
| Kingdom of Tonga | 80–84 years | 0.52 (-1.81, 2.91) |
| Kingdom of Tonga | 85–89 years | 0.68 (-2.72, 4.20) |
| Kingdom of Tonga | 90–94 years | 0.87 (-4.88, 6.97) |
| Kingdom of Tonga | 95+ years | 0.88 (-11.07, 14.42) |
| Kyrgyz Republic | 30–34 years | -0.43 (-2.30, 1.47) |
| Kyrgyz Republic | 35–39 years | -0.42 (-1.64, 0.82) |
| Kyrgyz Republic | 40–44 years | -0.44 (-1.34, 0.46) |
| Kyrgyz Republic | 45–49 years | -0.44 (-1.13, 0.25) |
| Kyrgyz Republic | 50–54 years | -0.39 (-0.94, 0.16) |
| Kyrgyz Republic | 55–59 years | -0.26 (-0.70, 0.17) |
| Kyrgyz Republic | 60–64 years | -0.13 (-0.49, 0.23) |
| Kyrgyz Republic | 65–69 years | -0.03 (-0.35, 0.28) |
| Kyrgyz Republic | 70–74 years | 0.04 (-0.27, 0.35) |
| Kyrgyz Republic | 75–79 years | 0.07 (-0.24, 0.39) |
| Kyrgyz Republic | 80–84 years | 0.09 (-0.28, 0.47) |
| Kyrgyz Republic | 85–89 years | 0.18 (-0.38, 0.74) |
| Kyrgyz Republic | 90–94 years | 0.29 (-0.79, 1.39) |
| Kyrgyz Republic | 95+ years | 0.31 (-2.78, 3.49) |
| Lao People's Democratic Republic | 30–34 years | -0.20 (-1.69, 1.31) |
| Lao People's Democratic Republic | 35–39 years | -0.21 (-1.21, 0.79) |
| Lao People's Democratic Republic | 40–44 years | -0.17 (-0.93, 0.60) |
| Lao People's Democratic Republic | 45–49 years | -0.05 (-0.67, 0.57) |
| Lao People's Democratic Republic | 50–54 years | 0.13 (-0.39, 0.64) |
| Lao People's Democratic Republic | 55–59 years | 0.31 (-0.12, 0.75) |
| Lao People's Democratic Republic | 60–64 years | 0.49 (0.10, 0.87) |
| Lao People's Democratic Republic | 65–69 years | 0.63 (0.28, 0.98) |
| Lao People's Democratic Republic | 70–74 years | 0.76 (0.42, 1.10) |
| Lao People's Democratic Republic | 75–79 years | 0.86 (0.48, 1.24) |
| Lao People's Democratic Republic | 80–84 years | 0.97 (0.42, 1.51) |
| Lao People's Democratic Republic | 85–89 years | 1.15 (0.11, 2.19) |
| Lao People's Democratic Republic | 90–94 years | 1.36 (-0.98, 3.76) |
| Lao People's Democratic Republic | 95+ years | 1.38 (-4.05, 7.12) |
| Lebanese Republic | 30–34 years | 0.48 (-1.41, 2.40) |
| Lebanese Republic | 35–39 years | 0.56 (-0.63, 1.77) |
| Lebanese Republic | 40–44 years | 0.71 (-0.16, 1.57) |
| Lebanese Republic | 45–49 years | 0.85 (0.20, 1.51) |
| Lebanese Republic | 50–54 years | 0.99 (0.47, 1.50) |
| Lebanese Republic | 55–59 years | 1.06 (0.64, 1.48) |
| Lebanese Republic | 60–64 years | 1.05 (0.69, 1.41) |
| Lebanese Republic | 65–69 years | 0.99 (0.69, 1.30) |
| Lebanese Republic | 70–74 years | 1.00 (0.73, 1.28) |
| Lebanese Republic | 75–79 years | 1.10 (0.82, 1.38) |
| Lebanese Republic | 80–84 years | 1.29 (0.94, 1.65) |
| Lebanese Republic | 85–89 years | 1.52 (0.99, 2.05) |
| Lebanese Republic | 90–94 years | 1.67 (0.72, 2.63) |
| Lebanese Republic | 95+ years | 1.80 (-0.38, 4.03) |
| Malaysia | 30–34 years | 0.59 (-0.08, 1.26) |
| Malaysia | 35–39 years | 0.56 (0.12, 1.00) |
| Malaysia | 40–44 years | 0.54 (0.21, 0.87) |
| Malaysia | 45–49 years | 0.54 (0.28, 0.80) |
| Malaysia | 50–54 years | 0.60 (0.39, 0.82) |
| Malaysia | 55–59 years | 0.72 (0.54, 0.90) |
| Malaysia | 60–64 years | 0.85 (0.69, 1.01) |
| Malaysia | 65–69 years | 0.96 (0.82, 1.10) |
| Malaysia | 70–74 years | 1.07 (0.93, 1.21) |
| Malaysia | 75–79 years | 1.11 (0.96, 1.27) |
| Malaysia | 80–84 years | 1.15 (0.96, 1.34) |
| Malaysia | 85–89 years | 1.23 (0.98, 1.49) |
| Malaysia | 90–94 years | 1.32 (0.90, 1.74) |
| Malaysia | 95+ years | 1.42 (0.70, 2.14) |
| Mongolia | 30–34 years | -0.26 (-2.85, 2.40) |
| Mongolia | 35–39 years | -0.22 (-1.91, 1.49) |
| Mongolia | 40–44 years | -0.26 (-1.51, 1.01) |
| Mongolia | 45–49 years | -0.29 (-1.27, 0.71) |
| Mongolia | 50–54 years | -0.29 (-1.08, 0.52) |
| Mongolia | 55–59 years | -0.22 (-0.87, 0.44) |
| Mongolia | 60–64 years | -0.07 (-0.64, 0.50) |
| Mongolia | 65–69 years | 0.11 (-0.41, 0.64) |
| Mongolia | 70–74 years | 0.24 (-0.26, 0.74) |
| Mongolia | 75–79 years | 0.31 (-0.24, 0.86) |
| Mongolia | 80–84 years | 0.35 (-0.35, 1.06) |
| Mongolia | 85–89 years | 0.51 (-0.64, 1.68) |
| Mongolia | 90–94 years | 0.78 (-1.70, 3.33) |
| Mongolia | 95+ years | 0.91 (-5.66, 7.94) |
| Montenegro | 30–34 years | -0.02 (-6.42, 6.82) |
| Montenegro | 35–39 years | -0.03 (-3.80, 3.89) |
| Montenegro | 40–44 years | 0.01 (-2.54, 2.63) |
| Montenegro | 45–49 years | 0.07 (-1.72, 1.90) |
| Montenegro | 50–54 years | 0.13 (-1.14, 1.41) |
| Montenegro | 55–59 years | 0.14 (-0.77, 1.05) |
| Montenegro | 60–64 years | 0.11 (-0.57, 0.79) |
| Montenegro | 65–69 years | 0.09 (-0.45, 0.63) |
| Montenegro | 70–74 years | 0.15 (-0.34, 0.65) |
| Montenegro | 75–79 years | 0.32 (-0.20, 0.84) |
| Montenegro | 80–84 years | 0.48 (-0.11, 1.08) |
| Montenegro | 85–89 years | 0.57 (-0.34, 1.48) |
| Montenegro | 90–94 years | 0.62 (-1.09, 2.36) |
| Montenegro | 95+ years | 0.63 (-3.32, 4.74) |
| New Zealand | 30–34 years | 0.85 (-1.59, 3.35) |
| New Zealand | 35–39 years | 0.79 (-0.79, 2.40) |
| New Zealand | 40–44 years | 0.76 (-0.39, 1.91) |
| New Zealand | 45–49 years | 0.70 (-0.13, 1.54) |
| New Zealand | 50–54 years | 0.62 (-0.01, 1.25) |
| New Zealand | 55–59 years | 0.53 (0.06, 1.00) |
| New Zealand | 60–64 years | 0.47 (0.11, 0.83) |
| New Zealand | 65–69 years | 0.45 (0.17, 0.72) |
| New Zealand | 70–74 years | 0.46 (0.23, 0.69) |
| New Zealand | 75–79 years | 0.51 (0.29, 0.73) |
| New Zealand | 80–84 years | 0.59 (0.34, 0.83) |
| New Zealand | 85–89 years | 0.69 (0.33, 1.05) |
| New Zealand | 90–94 years | 0.77 (0.10, 1.45) |
| New Zealand | 95+ years | 0.80 (-0.79, 2.41) |
| North Macedonia | 30–34 years | 0.03 (-3.38, 3.58) |
| North Macedonia | 35–39 years | 0.07 (-1.95, 2.13) |
| North Macedonia | 40–44 years | 0.13 (-1.22, 1.51) |
| North Macedonia | 45–49 years | 0.18 (-0.76, 1.14) |
| North Macedonia | 50–54 years | 0.16 (-0.52, 0.84) |
| North Macedonia | 55–59 years | 0.12 (-0.39, 0.62) |
| North Macedonia | 60–64 years | 0.10 (-0.28, 0.48) |
| North Macedonia | 65–69 years | 0.13 (-0.17, 0.44) |
| North Macedonia | 70–74 years | 0.25 (-0.03, 0.53) |
| North Macedonia | 75–79 years | 0.42 (0.11, 0.73) |
| North Macedonia | 80–84 years | 0.56 (0.14, 0.98) |
| North Macedonia | 85–89 years | 0.70 (-0.01, 1.42) |
| North Macedonia | 90–94 years | 0.90 (-0.60, 2.43) |
| North Macedonia | 95+ years | 1.21 (-2.66, 5.24) |
| Northern Mariana Islands | 30–34 years | 0.32 (-17.12, 21.44) |
| Northern Mariana Islands | 35–39 years | 0.24 (-10.27, 11.98) |
| Northern Mariana Islands | 40–44 years | 0.02 (-6.75, 7.29) |
| Northern Mariana Islands | 45–49 years | 0.11 (-4.81, 5.28) |
| Northern Mariana Islands | 50–54 years | -0.06 (-3.97, 4.01) |
| Northern Mariana Islands | 55–59 years | 0.05 (-3.42, 3.64) |
| Northern Mariana Islands | 60–64 years | -0.01 (-3.24, 3.32) |
| Northern Mariana Islands | 65–69 years | -0.10 (-3.19, 3.08) |
| Northern Mariana Islands | 70–74 years | -0.19 (-3.27, 2.99) |
| Northern Mariana Islands | 75–79 years | -0.19 (-3.54, 3.27) |
| Northern Mariana Islands | 80–84 years | -0.08 (-4.44, 4.48) |
| Northern Mariana Islands | 85–89 years | 0.13 (-6.83, 7.60) |
| Northern Mariana Islands | 90–94 years | 0.33 (-13.34, 16.15) |
| Northern Mariana Islands | 95+ years | 0.31 (-32.99, 50.16) |
| Palestine | 30–34 years | 0.45 (-1.79, 2.75) |
| Palestine | 35–39 years | 0.58 (-0.87, 2.05) |
| Palestine | 40–44 years | 0.70 (-0.38, 1.78) |
| Palestine | 45–49 years | 0.83 (-0.02, 1.69) |
| Palestine | 50–54 years | 1.01 (0.30, 1.73) |
| Palestine | 55–59 years | 1.17 (0.56, 1.79) |
| Palestine | 60–64 years | 1.23 (0.69, 1.78) |
| Palestine | 65–69 years | 1.25 (0.75, 1.75) |
| Palestine | 70–74 years | 1.20 (0.74, 1.66) |
| Palestine | 75–79 years | 1.14 (0.66, 1.62) |
| Palestine | 80–84 years | 1.20 (0.60, 1.80) |
| Palestine | 85–89 years | 1.40 (0.44, 2.38) |
| Palestine | 90–94 years | 1.56 (-0.41, 3.57) |
| Palestine | 95+ years | 1.58 (-3.03, 6.40) |
| People's Democratic Republic of Algeria | 30–34 years | 0.38 (-0.29, 1.05) |
| People's Democratic Republic of Algeria | 35–39 years | 0.56 (0.15, 0.96) |
| People's Democratic Republic of Algeria | 40–44 years | 0.68 (0.38, 0.98) |
| People's Democratic Republic of Algeria | 45–49 years | 0.81 (0.57, 1.05) |
| People's Democratic Republic of Algeria | 50–54 years | 0.99 (0.79, 1.19) |
| People's Democratic Republic of Algeria | 55–59 years | 1.13 (0.95, 1.30) |
| People's Democratic Republic of Algeria | 60–64 years | 1.12 (0.97, 1.27) |
| People's Democratic Republic of Algeria | 65–69 years | 1.04 (0.90, 1.18) |
| People's Democratic Republic of Algeria | 70–74 years | 0.88 (0.75, 1.01) |
| People's Democratic Republic of Algeria | 75–79 years | 0.67 (0.55, 0.80) |
| People's Democratic Republic of Algeria | 80–84 years | 0.63 (0.48, 0.78) |
| People's Democratic Republic of Algeria | 85–89 years | 1.06 (0.73, 1.38) |
| People's Democratic Republic of Algeria | 90–94 years | 1.82 (0.77, 2.87) |
| People's Democratic Republic of Algeria | 95+ years | 2.12 (-1.08, 5.42) |
| People's Republic of Bangladesh | 30–34 years | 0.03 (-0.28, 0.34) |
| People's Republic of Bangladesh | 35–39 years | 0.16 (-0.03, 0.36) |
| People's Republic of Bangladesh | 40–44 years | 0.28 (0.14, 0.43) |
| People's Republic of Bangladesh | 45-49 years | 0.40 (0.28, 0.51) |
| People's Republic of Bangladesh | 50–54 years | 0.49 (0.39, 0.59) |
| People's Republic of Bangladesh | 55-59 years | 0.56 (0.48, 0.65) |
| People's Republic of Bangladesh | 60–64 years | 0.65 (0.58, 0.73) |
| People's Republic of Bangladesh | 65-69 years | 0.78 (0.72, 0.85) |
| People's Republic of Bangladesh | 70–74 years | 0.95 (0.88, 1.02) |
| People's Republic of Bangladesh | 75–79 years | 1.10 (1.03, 1.18) |
| People's Republic of Bangladesh | 80–84 years | 1.25 (1.16, 1.34) |
| People's Republic of Bangladesh | 85–89 years | 1.39 (1.25, 1.54) |
| People's Republic of Bangladesh | 90–94 years | 1.46 (1.17, 1.74) |
| People's Republic of Bangladesh | 95+ years | 1.43 (0.69, 2.17) |
| People's Republic of China | 30–34 years | 0.98 (0.26, 1.70) |
| People's Republic of China | 35–39 years | 1.27 (0.85, 1.70) |
| People's Republic of China | 40–44 years | 1.62 (1.36, 1.87) |
| People's Republic of China | 45–49 years | 1.94 (1.79, 2.10) |
| People's Republic of China | 50–54 years | 2.25 (2.14, 2.37) |
| People's Republic of China | 55–59 years | 2.56 (2.46, 2.65) |
| People's Republic of China | 60–64 years | 2.81 (2.72, 2.89) |
| People's Republic of China | 65–69 years | 3.02 (2.94, 3.10) |
| People's Republic of China | 70–74 years | 3.17 (3.09, 3.25) |
| People's Republic of China | 75–79 years | 3.28 (3.19, 3.37) |
| People's Republic of China | 80–84 years | 3.38 (3.25, 3.50) |
| People's Republic of China | 85–89 years | 3.46 (3.25, 3.67) |
| People's Republic of China | 90–94 years | 3.50 (3.05, 3.95) |
| People's Republic of China | 95+ years | 3.46 (2.13, 4.81) |
| Plurinational State of Bolivia | 30–34 years | -0.86 (-1.75, 0.03) |
| Plurinational State of Bolivia | 35–39 years | -0.90 (-1.40, -0.40) |
| Plurinational State of Bolivia | 40–44 years | -0.61 (-0.95, -0.27) |
| Plurinational State of Bolivia | 45–49 years | -0.05 (-0.31, 0.21) |
| Plurinational State of Bolivia | 50-54 years | 0.62 (0.40, 0.84) |
| Plurinational State of Bolivia | 55–59 years | 1.33 (1.13, 1.53) |
| Plurinational State of Bolivia | 60–64 years | 1.93 (1.73, 2.12) |
| Plurinational State of Bolivia | 65–69 years | 2.27 (2.08, 2.46) |
| Plurinational State of Bolivia | 70–74 years | 2.35 (2.16, 2.55) |
| Plurinational State of Bolivia | 75–79 years | 2.26 (2.03, 2.48) |
| Plurinational State of Bolivia | 80–84 years | 2.15 (1.86, 2.45) |
| Plurinational State of Bolivia | 85–89 years | 2.08 (1.60, 2.55) |
| Plurinational State of Bolivia | 90–94 years | 1.96 (1.01, 2.91) |
| Plurinational State of Bolivia | 95+ years | 1.80 (-0.44, 4.08) |
| Portuguese Republic | 30–34 years | 0.17 (-0.90, 1.25) |
| Portuguese Republic | 35–39 years | 0.38 (-0.25, 1.02) |
| Portuguese Republic | 40–44 years | 0.59 (0.16, 1.03) |
| Portuguese Republic | 45–49 years | 0.77 (0.44, 1.10) |
| Portuguese Republic | 50–54 years | 0.88 (0.63, 1.14) |
| Portuguese Republic | 55–59 years | 0.99 (0.79, 1.19) |
| Portuguese Republic | 60–64 years | 1.12 (0.96, 1.27) |
| Portuguese Republic | 65–69 years | 1.24 (1.12, 1.37) |
| Portuguese Republic | 70–74 years | 1.30 (1.19, 1.40) |
| Portuguese Republic | 75–79 years | 1.32 (1.22, 1.42) |
| Portuguese Republic | 80–84 years | 1.35 (1.24, 1.46) |
| Portuguese Republic | 85–89 years | 1.42 (1.25, 1.58) |
| Portuguese Republic | 90–94 years | 1.46 (1.11, 1.81) |
| Portuguese Republic | 95+ years | 1.33 (0.42, 2.25) |
| Principality of Andorra | 30–34 years | 0.32 (-10.32, 12.21) |
| Principality of Andorra | 35–39 years | 0.33 (-6.08, 7.17) |
| Principality of Andorra | 40–44 years | 0.40 (-4.02, 5.03) |
| Principality of Andorra | 45–49 years | 0.45 (-2.85, 3.86) |
| Principality of Andorra | 50–54 years | 0.53 (-2.09, 3.21) |
| Principality of Andorra | 55–59 years | 0.63 (-1.51, 2.81) |
| Principality of Andorra | 60–64 years | 0.74 (-1.03, 2.55) |
| Principality of Andorra | 65–69 years | 0.85 (-0.62, 2.35) |
| Principality of Andorra | 70–74 years | 0.90 (-0.37, 2.19) |
| Principality of Andorra | 75–79 years | 0.87 (-0.36, 2.12) |
| Principality of Andorra | 80–84 years | 0.82 (-0.51, 2.17) |
| Principality of Andorra | 85–89 years | 0.80 (-1.00, 2.64) |
| Principality of Andorra | 90–94 years | 0.81 (-2.65, 4.40) |
| Principality of Andorra | 95+ years | 0.76 (-11.17, 14.28) |
| Principality of Monaco | 30–34 years | 0.27 (-17.34, 21.64) |
| Principality of Monaco | 35–39 years | 0.30 (-10.71, 12.66) |
| Principality of Monaco | 40–44 years | 0.46 (-7.02, 8.55) |
| Principality of Monaco | 45–49 years | 0.57 (-4.54, 5.94) |
| Principality of Monaco | 50–54 years | 0.65 (-2.97, 4.41) |
| Principality of Monaco | 55–59 years | 0.71 (-2.07, 3.56) |
| Principality of Monaco | 60–64 years | 0.79 (-1.35, 2.97) |
| Principality of Monaco | 65–69 years | 0.88 (-0.75, 2.54) |
| Principality of Monaco | 70–74 years | 0.98 (-0.30, 2.27) |
| Principality of Monaco | 75–79 years | 1.09 (-0.11, 2.30) |
| Principality of Monaco | 80–84 years | 1.19 (-0.05, 2.44) |
| Principality of Monaco | 85–89 years | 1.19 (-0.37, 2.77) |
| Principality of Monaco | 90–94 years | 1.11 (-1.46, 3.74) |
| Principality of Monaco | 95+ years | 0.85 (-4.68, 6.70) |
| Puerto Rico | 30–34 years | 0.25 (-1.69, 2.22) |
| Puerto Rico | 35–39 years | 0.38 (-0.70, 1.47) |
| Puerto Rico | 40–44 years | 0.47 (-0.24, 1.18) |
| Puerto Rico | 45–49 years | 0.53 (0.02, 1.05) |
| Puerto Rico | 50–54 years | 0.59 (0.19, 0.99) |
| Puerto Rico | 55–59 years | 0.64 (0.31, 0.97) |
| Puerto Rico | 60–64 years | 0.68 (0.40, 0.97) |
| Puerto Rico | 65–69 years | 0.71 (0.46, 0.97) |
| Puerto Rico | 70–74 years | 0.72 (0.49, 0.96) |
| Puerto Rico | 75–79 years | 0.73 (0.50, 0.96) |
| Puerto Rico | 80–84 years | 0.70 (0.45, 0.96) |
| Puerto Rico | 85–89 years | 0.61 (0.27, 0.95) |
| Puerto Rico | 90–94 years | 0.58 (-0.03, 1.20) |
| Puerto Rico | 95+ years | 0.51 (-0.97, 2.00) |
| Republic of Albania | 30–34 years | -0.06 (-2.94, 2.92) |
| Republic of Albania | 35–39 years | -0.08 (-1.88, 1.75) |
| Republic of Albania | 40–44 years | -0.12 (-1.34, 1.12) |
| Republic of Albania | 45–49 years | -0.10 (-0.94, 0.74) |
| Republic of Albania | 50–54 years | -0.06 (-0.65, 0.53) |
| Republic of Albania | 55–59 years | -0.02 (-0.46, 0.41) |
| Republic of Albania | 60–64 years | 0.04 (-0.30, 0.38) |
| Republic of Albania | 65–69 years | 0.14 (-0.14, 0.43) |
| Republic of Albania | 70–74 years | 0.29 (0.04, 0.55) |
| Republic of Albania | 75–79 years | 0.42 (0.15, 0.69) |
| Republic of Albania | 80–84 years | 0.58 (0.21, 0.94) |
| Republic of Albania | 85–89 years | 0.77 (0.16, 1.39) |
| Republic of Albania | 90–94 years | 0.87 (-0.32, 2.07) |
| Republic of Albania | 95+ years | 0.91 (-1.62, 3.49) |
| Republic of Angola | 30–34 years | 0.13 (-0.79, 1.05) |
| Republic of Angola | 35–39 years | 0.15 (-0.47, 0.77) |
| Republic of Angola | 40–44 years | 0.17 (-0.31, 0.65) |
| Republic of Angola | 45–49 years | 0.22 (-0.18, 0.62) |
| Republic of Angola | 50–54 years | 0.28 (-0.06, 0.63) |
| Republic of Angola | 55–59 years | 0.36 (0.05, 0.67) |
| Republic of Angola | 60–64 years | 0.46 (0.18, 0.74) |
| Republic of Angola | 65–69 years | 0.56 (0.30, 0.83) |
| Republic of Angola | 70–74 years | 0.69 (0.41, 0.97) |
| Republic of Angola | 75–79 years | 0.84 (0.50, 1.18) |
| Republic of Angola | 80–84 years | 1.01 (0.52, 1.50) |
| Republic of Angola | 85–89 years | 1.16 (0.30, 2.03) |
| Republic of Angola | 90–94 years | 1.26 (-0.53, 3.08) |
| Republic of Angola | 95+ years | 1.27 (-3.00, 5.73) |
| Republic of Armenia | 30–34 years | 0.05 (-2.52, 2.69) |
| Republic of Armenia | 35–39 years | 0.08 (-1.51, 1.69) |
| Republic of Armenia | 40–44 years | 0.04 (-1.10, 1.20) |
| Republic of Armenia | 45–49 years | 0.00 (-0.85, 0.86) |
| Republic of Armenia | 50–54 years | 0.02 (-0.61, 0.64) |
| Republic of Armenia | 55–59 years | 0.08 (-0.37, 0.53) |
| Republic of Armenia | 60–64 years | 0.15 (-0.19, 0.50) |
| Republic of Armenia | 65–69 years | 0.22 (-0.06, 0.51) |
| Republic of Armenia | 70–74 years | 0.28 (-0.01, 0.57) |
| Republic of Armenia | 75–79 years | 0.33 (0.01, 0.64) |
| Republic of Armenia | 80–84 years | 0.36 (-0.01, 0.74) |
| Republic of Armenia | 85–89 years | 0.41 (-0.17, 0.99) |
| Republic of Armenia | 90–94 years | 0.43 (-0.70, 1.58) |
| Republic of Armenia | 95+ years | 0.40 (-2.56, 3.44) |
| Republic of Austria | 30–34 years | 0.27 (-0.84, 1.39) |
| Republic of Austria | 35–39 years | 0.34 (-0.37, 1.05) |
| Republic of Austria | 40–44 years | 0.38 (-0.12, 0.88) |
| Republic of Austria | 45–49 years | 0.44 (0.08, 0.79) |
| Republic of Austria | 50–54 years | 0.51 (0.25, 0.78) |
| Republic of Austria | 55–59 years | 0.61 (0.40, 0.81) |
| Republic of Austria | 60–64 years | 0.76 (0.60, 0.92) |
| Republic of Austria | 65–69 years | 0.95 (0.82, 1.08) |
| Republic of Austria | 70–74 years | 1.12 (1.01, 1.23) |
| Republic of Austria | 75–79 years | 1.21 (1.11, 1.31) |
| Republic of Austria | 80–84 years | 1.26 (1.15, 1.36) |
| Republic of Austria | 85–89 years | 1.26 (1.13, 1.40) |
| Republic of Austria | 90–94 years | 1.25 (1.01, 1.49) |
| Republic of Austria | 95+ years | 1.17 (0.55, 1.80) |
| Republic of Azerbaijan | 30–34 years | -0.05 (-1.42, 1.34) |
| Republic of Azerbaijan | 35–39 years | -0.07 (-0.95, 0.83) |
| Republic of Azerbaijan | 40–44 years | -0.09 (-0.75, 0.57) |
| Republic of Azerbaijan | 45–49 years | -0.09 (-0.59, 0.41) |
| Republic of Azerbaijan | 50–54 years | -0.03 (-0.42, 0.35) |
| Republic of Azerbaijan | 55–59 years | 0.06 (-0.24, 0.36) |
| Republic of Azerbaijan | 60–64 years | 0.16 (-0.08, 0.41) |
| Republic of Azerbaijan | 65–69 years | 0.31 (0.09, 0.53) |
| Republic of Azerbaijan | 70–74 years | 0.52 (0.30, 0.74) |
| Republic of Azerbaijan | 75–79 years | 0.74 (0.52, 0.96) |
| Republic of Azerbaijan | 80–84 years | 0.94 (0.69, 1.19) |
| Republic of Azerbaijan | 85–89 years | 1.11 (0.75, 1.48) |
| Republic of Azerbaijan | 90–94 years | 1.21 (0.51, 1.92) |
| Republic of Azerbaijan | 95+ years | 1.07 (-1.18, 3.36) |
| Republic of Belarus | 30–34 years | -0.18 (-1.67, 1.33) |
| Republic of Belarus | 35–39 years | -0.17 (-1.04, 0.71) |
| Republic of Belarus | 40–44 years | -0.11 (-0.70, 0.48) |
| Republic of Belarus | 45–49 years | -0.03 (-0.43, 0.38) |
| Republic of Belarus | 50–54 years | 0.09 (-0.19, 0.38) |
| Republic of Belarus | 55–59 years | 0.21 (0.01, 0.41) |
| Republic of Belarus | 60–64 years | 0.30 (0.15, 0.45) |
| Republic of Belarus | 65–69 years | 0.29 (0.16, 0.41) |
| Republic of Belarus | 70–74 years | 0.16 (0.05, 0.28) |
| Republic of Belarus | 75–79 years | -0.01 (-0.13, 0.11) |
| Republic of Belarus | 80–84 years | -0.15 (-0.29, -0.02) |
| Republic of Belarus | 85–89 years | -0.26 (-0.46, -0.06) |
| Republic of Belarus | 90–94 years | -0.33 (-0.70, 0.05) |
| Republic of Belarus | 95+ years | -0.39 (-1.33, 0.56) |
| Republic of Benin | 30–34 years | 0.26 (-1.12, 1.66) |
| Republic of Benin | 35–39 years | 0.28 (-0.67, 1.25) |
| Republic of Benin | 40–44 years | 0.33 (-0.43, 1.10) |
| Republic of Benin | 45–49 years | 0.39 (-0.26, 1.05) |
| Republic of Benin | 50–54 years | 0.42 (-0.15, 1.00) |
| Republic of Benin | 55–59 years | 0.45 (-0.06, 0.95) |
| Republic of Benin | 60–64 years | 0.46 (0.03, 0.90) |
| Republic of Benin | 65–69 years | 0.45 (0.07, 0.84) |
| Republic of Benin | 70–74 years | 0.44 (0.09, 0.78) |
| Republic of Benin | 75–79 years | 0.48 (0.15, 0.81) |
| Republic of Benin | 80–84 years | 0.57 (0.17, 0.98) |
| Republic of Benin | 85–89 years | 0.70 (0.07, 1.34) |
| Republic of Benin | 90–94 years | 0.82 (-0.34, 1.99) |
| Republic of Benin | 95+ years | 0.92 (-1.67, 3.59) |
| Republic of Botswana | 30–34 years | 0.15 (-2.37, 2.74) |
| Republic of Botswana | 35–39 years | 0.27 (-1.41, 1.99) |
| Republic of Botswana | 40–44 years | 0.37 (-0.97, 1.73) |
| Republic of Botswana | 45–49 years | 0.39 (-0.75, 1.54) |
| Republic of Botswana | 50–54 years | 0.42 (-0.58, 1.43) |
| Republic of Botswana | 55–59 years | 0.51 (-0.37, 1.39) |
| Republic of Botswana | 60–64 years | 0.67 (-0.11, 1.46) |
| Republic of Botswana | 65–69 years | 0.86 (0.14, 1.57) |
| Republic of Botswana | 70–74 years | 0.98 (0.29, 1.67) |
| Republic of Botswana | 75–79 years | 1.01 (0.27, 1.75) |
| Republic of Botswana | 80–84 years | 0.99 (0.01, 1.98) |
| Republic of Botswana | 85–89 years | 1.01 (-0.72, 2.77) |
| Republic of Botswana | 90–94 years | 1.07 (-2.63, 4.92) |
| Republic of Botswana | 95+ years | 1.11 (-8.34, 11.55) |
| Republic of Bulgaria | 30–34 years | 1.98 (-0.25, 4.26) |
| Republic of Bulgaria | 35–39 years | 1.70 (0.39, 3.02) |
| Republic of Bulgaria | 40–44 years | 1.24 (0.40, 2.09) |
| Republic of Bulgaria | 45–49 years | 0.82 (0.25, 1.40) |
| Republic of Bulgaria | 50–54 years | 0.43 (0.02, 0.84) |
| Republic of Bulgaria | 55–59 years | 0.04 (-0.24, 0.33) |
| Republic of Bulgaria | 60–64 years | -0.37 (-0.57, -0.17) |
| Republic of Bulgaria | 65–69 years | -0.79 (-0.93, -0.64) |
| Republic of Bulgaria | 70–74 years | -1.15 (-1.26, -1.04) |
| Republic of Bulgaria | 75–79 years | -1.48 (-1.59, -1.37) |
| Republic of Bulgaria | 80–84 years | -1.79 (-1.91, -1.66) |
| Republic of Bulgaria | 85–89 years | -2.02 (-2.23, -1.81) |
| Republic of Bulgaria | 90–94 years | -2.18 (-3.31, -1.04) |
| Republic of Bulgaria | 95+ years | -2.37 (-5.42, 0.78) |
| Republic of Burundi | 30–34 years | 0.08 (-1.23, 1.41) |
| Republic of Burundi | 35–39 years | 0.04 (-0.86, 0.94) |
| Republic of Burundi | 40–44 years | 0.00 (-0.72, 0.73) |
| Republic of Burundi | 45–49 years | -0.00 (-0.62, 0.62) |
| Republic of Burundi | 50–54 years | 0.06 (-0.48, 0.60) |
| Republic of Burundi | 55–59 years | 0.17 (-0.31, 0.66) |
| Republic of Burundi | 60–64 years | 0.28 (-0.16, 0.71) |
| Republic of Burundi | 65–69 years | 0.36 (-0.05, 0.76) |
| Republic of Burundi | 70–74 years | 0.40 (0.00, 0.80) |
| Republic of Burundi | 75–79 years | 0.44 (0.01, 0.87) |
| Republic of Burundi | 80–84 years | 0.53 (-0.03, 1.09) |
| Republic of Burundi | 85–89 years | 0.66 (-0.28, 1.61) |
| Republic of Burundi | 90–94 years | 0.71 (-1.20, 2.67) |
| Republic of Burundi | 95+ years | 0.78 (-4.18, 6.01) |
| Republic of Cabo Verde | 30–34 years | 0.45 (-4.39, 5.53) |
| Republic of Cabo Verde | 35–39 years | 0.62 (-2.69, 4.04) |
| Republic of Cabo Verde | 40–44 years | 0.84 (-1.86, 3.62) |
| Republic of Cabo Verde | 45–49 years | 1.01 (-1.32, 3.40) |
| Republic of Cabo Verde | 50–54 years | 1.10 (-0.91, 3.15) |
| Republic of Cabo Verde | 55–59 years | 1.11 (-0.57, 2.81) |
| Republic of Cabo Verde | 60–64 years | 1.08 (-0.31, 2.49) |
| Republic of Cabo Verde | 65–69 years | 1.09 (-0.11, 2.30) |
| Republic of Cabo Verde | 70–74 years | 1.20 (0.13, 2.29) |
| Republic of Cabo Verde | 75–79 years | 1.41 (0.45, 2.38) |
| Republic of Cabo Verde | 80–84 years | 1.61 (0.66, 2.57) |
| Republic of Cabo Verde | 85–89 years | 1.69 (0.47, 2.92) |
| Republic of Cabo Verde | 90–94 years | 1.56 (-0.54, 3.72) |
| Republic of Cabo Verde | 95+ years | 1.34 (-2.98, 5.85) |
| Republic of Cameroon | 30–34 years | 0.34 (-0.53, 1.21) |
| Republic of Cameroon | 35–39 years | 0.40 (-0.20, 1.00) |
| Republic of Cameroon | 40–44 years | 0.45 (-0.03, 0.94) |
| Republic of Cameroon | 45–49 years | 0.47 (0.06, 0.88) |
| Republic of Cameroon | 50–54 years | 0.46 (0.11, 0.82) |
| Republic of Cameroon | 55–59 years | 0.45 (0.14, 0.76) |
| Republic of Cameroon | 60–64 years | 0.45 (0.18, 0.72) |
| Republic of Cameroon | 65–69 years | 0.50 (0.26, 0.73) |
| Republic of Cameroon | 70–74 years | 0.57 (0.34, 0.79) |
| Republic of Cameroon | 75–79 years | 0.59 (0.36, 0.83) |
| Republic of Cameroon | 80–84 years | 0.58 (0.28, 0.88) |
| Republic of Cameroon | 85–89 years | 0.54 (0.08, 1.01) |
| Republic of Cameroon | 90–94 years | 0.50 (-0.42, 1.42) |
| Republic of Cameroon | 95+ years | 0.50 (-1.76, 2.81) |
| Republic of Chad | 30–34 years | 0.39 (-0.95, 1.76) |
| Republic of Chad | 35–39 years | 0.41 (-0.52, 1.34) |
| Republic of Chad | 40–44 years | 0.47 (-0.26, 1.20) |
| Republic of Chad | 45–49 years | 0.53 (-0.09, 1.15) |
| Republic of Chad | 50–54 years | 0.57 (0.04, 1.11) |
| Republic of Chad | 55–59 years | 0.60 (0.13, 1.07) |
| Republic of Chad | 60–64 years | 0.61 (0.21, 1.02) |
| Republic of Chad | 65–69 years | 0.66 (0.30, 1.01) |
| Republic of Chad | 70–74 years | 0.65 (0.33, 0.97) |
| Republic of Chad | 75–79 years | 0.63 (0.31, 0.94) |
| Republic of Chad | 80–84 years | 0.59 (0.19, 0.98) |
| Republic of Chad | 85–89 years | 0.54 (-0.08, 1.17) |
| Republic of Chad | 90–94 years | 0.49 (-0.73, 1.73) |
| Republic of Chad | 95+ years | 0.54 (-2.34, 3.50) |
| Republic of Chile | 30–34 years | 0.67 (-0.48, 1.83) |
| Republic of Chile | 35–39 years | 0.82 (0.11, 1.54) |
| Republic of Chile | 40–44 years | 0.97 (0.47, 1.47) |
| Republic of Chile | 45–49 years | 1.07 (0.71, 1.43) |
| Republic of Chile | 50–54 years | 1.10 (0.83, 1.36) |
| Republic of Chile | 55–59 years | 1.12 (0.92, 1.32) |
| Republic of Chile | 60–64 years | 1.20 (1.04, 1.36) |
| Republic of Chile | 65–69 years | 1.32 (1.19, 1.46) |
| Republic of Chile | 70–74 years | 1.47 (1.36, 1.59) |
| Republic of Chile | 75–79 years | 1.59 (1.48, 1.71) |
| Republic of Chile | 80–84 years | 1.64 (1.51, 1.77) |
| Republic of Chile | 85–89 years | 1.61 (1.43, 1.79) |
| Republic of Chile | 90–94 years | 1.46 (1.14, 1.78) |
| Republic of Chile | 95+ years | 1.01 (0.19, 1.85) |
| Republic of Colombia | 30–34 years | 0.41 (-0.08, 0.90) |
| Republic of Colombia | 35–39 years | 0.52 (0.23, 0.82) |
| Republic of Colombia | 40–44 years | 0.61 (0.40, 0.82) |
| Republic of Colombia | 45–49 years | 0.72 (0.56, 0.88) |
| Republic of Colombia | 50–54 years | 0.86 (0.73, 0.99) |
| Republic of Colombia | 55–59 years | 0.96 (0.85, 1.08) |
| Republic of Colombia | 60–64 years | 1.00 (0.89, 1.10) |
| Republic of Colombia | 65–69 years | 0.95 (0.85, 1.05) |
| Republic of Colombia | 70–74 years | 0.86 (0.77, 0.96) |
| Republic of Colombia | 75–79 years | 0.83 (0.73, 0.93) |
| Republic of Colombia | 80–84 years | 0.88 (0.76, 1.00) |
| Republic of Colombia | 85–89 years | 1.00 (0.83, 1.16) |
| Republic of Colombia | 90–94 years | 1.08 (0.82, 1.35) |
| Republic of Colombia | 95+ years | 1.20 (0.69, 1.71) |
| Republic of Costa Rica | 30–34 years | 0.48 (-0.99, 1.97) |
| Republic of Costa Rica | 35–39 years | 0.58 (-0.30, 1.47) |
| Republic of Costa Rica | 40–44 years | 0.70 (0.07, 1.34) |
| Republic of Costa Rica | 45–49 years | 0.81 (0.33, 1.30) |
| Republic of Costa Rica | 50–54 years | 0.85 (0.45, 1.25) |
| Republic of Costa Rica | 55–59 years | 0.81 (0.46, 1.16) |
| Republic of Costa Rica | 60–64 years | 0.73 (0.41, 1.05) |
| Republic of Costa Rica | 65–69 years | 0.65 (0.36, 0.95) |
| Republic of Costa Rica | 70–74 years | 0.65 (0.37, 0.93) |
| Republic of Costa Rica | 75–79 years | 0.74 (0.45, 1.03) |
| Republic of Costa Rica | 80–84 years | 0.88 (0.55, 1.22) |
| Republic of Costa Rica | 85–89 years | 1.00 (0.55, 1.44) |
| Republic of Costa Rica | 90–94 years | 1.02 (0.33, 1.71) |
| Republic of Costa Rica | 95+ years | 0.90 (-0.40, 2.21) |
| Republic of Côte d'Ivoire | 30–34 years | 0.15 (-0.70, 1.01) |
| Republic of Côte d'Ivoire | 35–39 years | 0.29 (-0.29, 0.87) |
| Republic of Côte d'Ivoire | 40–44 years | 0.37 (-0.09, 0.84) |
| Republic of Côte d'Ivoire | 45–49 years | 0.40 (0.00, 0.80) |
| Republic of Côte d'Ivoire | 50–54 years | 0.42 (0.07, 0.78) |
| Republic of Côte d'Ivoire | 55–59 years | 0.46 (0.15, 0.78) |
| Republic of Côte d'Ivoire | 60–64 years | 0.51 (0.23, 0.79) |
| Republic of Côte d'Ivoire | 65–69 years | 0.55 (0.30, 0.80) |
| Republic of Côte d'Ivoire | 70–74 years | 0.57 (0.33, 0.82) |
| Republic of Côte d'Ivoire | 75–79 years | 0.61 (0.35, 0.87) |
| Republic of Côte d'Ivoire | 80–84 years | 0.64 (0.29, 0.99) |
| Republic of Côte d'Ivoire | 85–89 years | 0.65 (0.08, 1.22) |
| Republic of Côte d'Ivoire | 90–94 years | 0.64 (-0.51, 1.81) |
| Republic of Côte d'Ivoire | 95+ years | 0.65 (-2.25, 3.64) |
| Republic of Croatia | 30–34 years | -0.11 (-2.77, 2.62) |
| Republic of Croatia | 35–39 years | -0.10 (-1.62, 1.44) |
| Republic of Croatia | 40–44 years | -0.10 (-1.10, 0.92) |
| Republic of Croatia | 45–49 years | -0.06 (-0.74, 0.63) |
| Republic of Croatia | 50–54 years | -0.01 (-0.47, 0.46) |
| Republic of Croatia | 55–59 years | 0.04 (-0.28, 0.37) |
| Republic of Croatia | 60–64 years | 0.07 (-0.16, 0.31) |
| Republic of Croatia | 65–69 years | 0.08 (-0.11, 0.26) |
| Republic of Croatia | 70–74 years | 0.11 (-0.05, 0.26) |
| Republic of Croatia | 75–79 years | 0.21 (0.05, 0.38) |
| Republic of Croatia | 80–84 years | 0.41 (0.19, 0.62) |
| Republic of Croatia | 85–89 years | 0.56 (0.26, 0.87) |
| Republic of Croatia | 90–94 years | 0.66 (0.03, 1.29) |
| Republic of Croatia | 95+ years | 0.57 (-1.51, 2.68) |
| Republic of Cuba | 30–34 years | 0.48 (-0.57, 1.54) |
| Republic of Cuba | 35–39 years | 0.55 (-0.09, 1.19) |
| Republic of Cuba | 40–44 years | 0.63 (0.22, 1.05) |
| Republic of Cuba | 45–49 years | 0.76 (0.48, 1.05) |
| Republic of Cuba | 50–54 years | 0.94 (0.71, 1.17) |
| Republic of Cuba | 55–59 years | 1.12 (0.91, 1.32) |
| Republic of Cuba | 60–64 years | 1.22 (1.04, 1.41) |
| Republic of Cuba | 65–69 years | 1.25 (1.08, 1.42) |
| Republic of Cuba | 70–74 years | 1.23 (1.07, 1.39) |
| Republic of Cuba | 75–79 years | 1.17 (1.01, 1.33) |
| Republic of Cuba | 80–84 years | 1.13 (0.94, 1.31) |
| Republic of Cuba | 85–89 years | 1.08 (0.84, 1.32) |
| Republic of Cuba | 90–94 years | 1.07 (0.67, 1.48) |
| Republic of Cuba | 95+ years | 0.84 (-0.13, 1.82) |
| Republic of Cyprus | 30–34 years | 0.13 (-2.56, 2.91) |
| Republic of Cyprus | 35–39 years | 0.03 (-1.74, 1.83) |
| Republic of Cyprus | 40–44 years | -0.04 (-1.37, 1.31) |
| Republic of Cyprus | 45–49 years | -0.04 (-1.05, 0.98) |
| Republic of Cyprus | 50–54 years | 0.01 (-0.76, 0.79) |
| Republic of Cyprus | 55–59 years | 0.08 (-0.52, 0.68) |
| Republic of Cyprus | 60–64 years | 0.19 (-0.28, 0.67) |
| Republic of Cyprus | 65–69 years | 0.36 (-0.03, 0.75) |
| Republic of Cyprus | 70–74 years | 0.49 (0.15, 0.82) |
| Republic of Cyprus | 75–79 years | 0.53 (0.22, 0.84) |
| Republic of Cyprus | 80–84 years | 0.59 (0.20, 0.98) |
| Republic of Cyprus | 85–89 years | 0.67 (-0.29, 1.64) |
| Republic of Cyprus | 90–94 years | 0.88 (-1.56, 3.37) |
| Republic of Cyprus | 95+ years | 0.98 (-10.51, 13.94) |
| Republic of Djibouti | 30–34 years | -0.05 (-3.92, 3.98) |
| Republic of Djibouti | 35–39 years | 0.03 (-2.60, 2.73) |
| Republic of Djibouti | 40–44 years | 0.14 (-1.95, 2.27) |
| Republic of Djibouti | 45–49 years | 0.27 (-1.52, 2.09) |
| Republic of Djibouti | 50–54 years | 0.39 (-1.21, 2.02) |
| Republic of Djibouti | 55–59 years | 0.48 (-1.00, 1.98) |
| Republic of Djibouti | 60–64 years | 0.55 (-0.85, 1.97) |
| Republic of Djibouti | 65–69 years | 0.63 (-0.73, 2.01) |
| Republic of Djibouti | 70–74 years | 0.72 (-0.70, 2.16) |
| Republic of Djibouti | 75–79 years | 0.83 (-0.86, 2.55) |
| Republic of Djibouti | 80–84 years | 0.92 (-1.45, 3.36) |
| Republic of Djibouti | 85–89 years | 1.00 (-3.21, 5.40) |
| Republic of Djibouti | 90–94 years | 0.95 (-7.30, 9.94) |
| Republic of Djibouti | 95+ years | 0.79 (-16.96, 22.34) |
| Republic of Ecuador | 30–34 years | 0.19 (-0.55, 0.94) |
| Republic of Ecuador | 35–39 years | 0.54 (0.12, 0.96) |
| Republic of Ecuador | 40–44 years | 0.95 (0.66, 1.24) |
| Republic of Ecuador | 45–49 years | 1.34 (1.12, 1.56) |
| Republic of Ecuador | 50–54 years | 1.58 (1.39, 1.77) |
| Republic of Ecuador | 55–59 years | 1.70 (1.53, 1.87) |
| Republic of Ecuador | 60–64 years | 1.77 (1.61, 1.93) |
| Republic of Ecuador | 65–69 years | 1.83 (1.68, 1.99) |
| Republic of Ecuador | 70–74 years | 1.93 (1.78, 2.09) |
| Republic of Ecuador | 75–79 years | 2.07 (1.91, 2.24) |
| Republic of Ecuador | 80–84 years | 2.23 (2.03, 2.43) |
| Republic of Ecuador | 85–89 years | 2.30 (2.02, 2.59) |
| Republic of Ecuador | 90–94 years | 2.19 (1.71, 2.68) |
| Republic of Ecuador | 95+ years | 1.80 (0.68, 2.93) |
| Republic of El Salvador | 30–34 years | 0.22 (-1.15, 1.61) |
| Republic of El Salvador | 35–39 years | 0.36 (-0.47, 1.19) |
| Republic of El Salvador | 40–44 years | 0.47 (-0.11, 1.06) |
| Republic of El Salvador | 45–49 years | 0.59 (0.14, 1.05) |
| Republic of El Salvador | 50–54 years | 0.70 (0.32, 1.08) |
| Republic of El Salvador | 55–59 years | 0.79 (0.46, 1.11) |
| Republic of El Salvador | 60–64 years | 0.85 (0.56, 1.14) |
| Republic of El Salvador | 65–69 years | 0.89 (0.63, 1.15) |
| Republic of El Salvador | 70–74 years | 0.90 (0.65, 1.14) |
| Republic of El Salvador | 75–79 years | 0.91 (0.66, 1.15) |
| Republic of El Salvador | 80–84 years | 0.96 (0.68, 1.24) |
| Republic of El Salvador | 85–89 years | 1.04 (0.68, 1.41) |
| Republic of El Salvador | 90–94 years | 1.17 (0.62, 1.73) |
| Republic of El Salvador | 95+ years | 1.35 (0.41, 2.31) |
| Republic of Equatorial Guinea | 30–34 years | 0.35 (-3.69, 4.56) |
| Republic of Equatorial Guinea | 35–39 years | 0.31 (-2.55, 3.25) |
| Republic of Equatorial Guinea | 40–44 years | 0.35 (-1.93, 2.68) |
| Republic of Equatorial Guinea | 45–49 years | 0.46 (-1.44, 2.40) |
| Republic of Equatorial Guinea | 50–54 years | 0.60 (-1.03, 2.26) |
| Republic of Equatorial Guinea | 55–59 years | 0.77 (-0.66, 2.22) |
| Republic of Equatorial Guinea | 60–64 years | 0.95 (-0.33, 2.24) |
| Republic of Equatorial Guinea | 65–69 years | 1.15 (-0.03, 2.34) |
| Republic of Equatorial Guinea | 70–74 years | 1.42 (0.23, 2.61) |
| Republic of Equatorial Guinea | 75–79 years | 1.80 (0.43, 3.18) |
| Republic of Equatorial Guinea | 80–84 years | 2.19 (0.25, 4.17) |
| Republic of Equatorial Guinea | 85–89 years | 2.51 (-0.92, 6.04) |
| Republic of Equatorial Guinea | 90–94 years | 2.63 (-4.70, 10.52) |
| Republic of Equatorial Guinea | 95+ years | 2.58 (-15.04, 23.86) |
| Republic of Estonia | 30–34 years | 0.23 (-3.66, 4.27) |
| Republic of Estonia | 35–39 years | -0.00 (-2.29, 2.33) |
| Republic of Estonia | 40–44 years | -0.18 (-1.67, 1.33) |
| Republic of Estonia | 45–49 years | -0.28 (-1.29, 0.73) |
| Republic of Estonia | 50–54 years | -0.34 (-1.05, 0.37) |
| Republic of Estonia | 55–59 years | -0.34 (-0.84, 0.15) |
| Republic of Estonia | 60–64 years | -0.29 (-0.66, 0.08) |
| Republic of Estonia | 65–69 years | -0.23 (-0.52, 0.07) |
| Republic of Estonia | 70–74 years | -0.20 (-0.47, 0.06) |
| Republic of Estonia | 75–79 years | -0.24 (-0.51, 0.04) |
| Republic of Estonia | 80–84 years | -0.28 (-0.59, 0.04) |
| Republic of Estonia | 85–89 years | -0.29 (-0.76, 0.19) |
| Republic of Estonia | 90–94 years | -0.25 (-1.21, 0.72) |
| Republic of Estonia | 95+ years | -0.23 (-2.97, 2.58) |
| Republic of Fiji | 30–34 years | 0.33 (-4.01, 4.87) |
| Republic of Fiji | 35–39 years | 0.33 (-2.39, 3.12) |
| Republic of Fiji | 40–44 years | 0.30 (-1.67, 2.31) |
| Republic of Fiji | 45–49 years | 0.28 (-1.19, 1.77) |
| Republic of Fiji | 50–54 years | 0.28 (-0.84, 1.42) |
| Republic of Fiji | 55–59 years | 0.28 (-0.64, 1.21) |
| Republic of Fiji | 60–64 years | 0.26 (-0.54, 1.07) |
| Republic of Fiji | 65–69 years | 0.24 (-0.51, 1.00) |
| Republic of Fiji | 70–74 years | 0.28 (-0.48, 1.05) |
| Republic of Fiji | 75–79 years | 0.31 (-0.55, 1.18) |
| Republic of Fiji | 80–84 years | 0.29 (-0.86, 1.45) |
| Republic of Fiji | 85–89 years | 0.31 (-1.63, 2.30) |
| Republic of Fiji | 90–94 years | 0.49 (-4.07, 5.27) |
| Republic of Fiji | 95+ years | 0.68 (-9.56, 12.07) |
| Republic of Finland | 30–34 years | 0.39 (-1.03, 1.82) |
| Republic of Finland | 35–39 years | 0.46 (-0.42, 1.35) |
| Republic of Finland | 40–44 years | 0.54 (-0.09, 1.17) |
| Republic of Finland | 45–49 years | 0.59 (0.13, 1.05) |
| Republic of Finland | 50–54 years | 0.68 (0.35, 1.01) |
| Republic of Finland | 55–59 years | 0.88 (0.63, 1.13) |
| Republic of Finland | 60–64 years | 1.17 (0.98, 1.37) |
| Republic of Finland | 65–69 years | 1.47 (1.32, 1.62) |
| Republic of Finland | 70–74 years | 1.67 (1.54, 1.80) |
| Republic of Finland | 75–79 years | 1.74 (1.62, 1.87) |
| Republic of Finland | 80–84 years | 1.75 (1.61, 1.89) |
| Republic of Finland | 85–89 years | 1.76 (1.57, 1.95) |
| Republic of Finland | 90–94 years | 1.75 (1.42, 2.07) |
| Republic of Finland | 95+ years | 1.75 (0.97, 2.53) |
| Republic of Ghana | 30–34 years | 0.18 (-0.62, 0.99) |
| Republic of Ghana | 35–39 years | 0.19 (-0.35, 0.74) |
| Republic of Ghana | 40–44 years | 0.22 (-0.21, 0.66) |
| Republic of Ghana | 45–49 years | 0.27 (-0.10, 0.64) |
| Republic of Ghana | 50–54 years | 0.33 (0.01, 0.66) |
| Republic of Ghana | 55–59 years | 0.41 (0.13, 0.69) |
| Republic of Ghana | 60–64 years | 0.50 (0.26, 0.74) |
| Republic of Ghana | 65–69 years | 0.60 (0.38, 0.81) |
| Republic of Ghana | 70–74 years | 0.67 (0.46, 0.87) |
| Republic of Ghana | 75–79 years | 0.72 (0.51, 0.93) |
| Republic of Ghana | 80–84 years | 0.78 (0.51, 1.05) |
| Republic of Ghana | 85–89 years | 0.84 (0.40, 1.28) |
| Republic of Ghana | 90–94 years | 0.86 (-0.02, 1.74) |
| Republic of Ghana | 95+ years | 0.88 (-1.27, 3.07) |
| Republic of Guatemala | 30–34 years | 0.23 (-0.73, 1.20) |
| Republic of Guatemala | 35–39 years | 0.34 (-0.26, 0.94) |
| Republic of Guatemala | 40–44 years | 0.47 (0.02, 0.91) |
| Republic of Guatemala | 45–49 years | 0.57 (0.21, 0.93) |
| Republic of Guatemala | 50–54 years | 0.62 (0.32, 0.93) |
| Republic of Guatemala | 55–59 years | 0.61 (0.34, 0.89) |
| Republic of Guatemala | 60–64 years | 0.58 (0.33, 0.83) |
| Republic of Guatemala | 65–69 years | 0.57 (0.33, 0.80) |
| Republic of Guatemala | 70–74 years | 0.59 (0.36, 0.82) |
| Republic of Guatemala | 75–79 years | 0.65 (0.39, 0.90) |
| Republic of Guatemala | 80–84 years | 0.75 (0.41, 1.09) |
| Republic of Guatemala | 85–89 years | 0.92 (0.37, 1.48) |
| Republic of Guatemala | 90–94 years | 1.08 (-0.11, 2.28) |
| Republic of Guatemala | 95+ years | 1.18 (-2.51, 5.00) |
| Republic of Guinea-Bissau | 30–34 years | 0.01 (-3.14, 3.26) |
| Republic of Guinea-Bissau | 35–39 years | 0.03 (-2.14, 2.25) |
| Republic of Guinea-Bissau | 40–44 years | 0.09 (-1.65, 1.87) |
| Republic of Guinea-Bissau | 45–49 years | 0.15 (-1.35, 1.67) |
| Republic of Guinea-Bissau | 50–54 years | 0.18 (-1.13, 1.51) |
| Republic of Guinea-Bissau | 55–59 years | 0.20 (-0.95, 1.37) |
| Republic of Guinea-Bissau | 60–64 years | 0.24 (-0.78, 1.26) |
| Republic of Guinea-Bissau | 65–69 years | 0.28 (-0.63, 1.19) |
| Republic of Guinea-Bissau | 70–74 years | 0.33 (-0.53, 1.20) |
| Republic of Guinea-Bissau | 75–79 years | 0.41 (-0.52, 1.36) |
| Republic of Guinea-Bissau | 80–84 years | 0.52 (-0.71, 1.77) |
| Republic of Guinea-Bissau | 85–89 years | 0.65 (-1.51, 2.85) |
| Republic of Guinea-Bissau | 90–94 years | 0.74 (-4.00, 5.73) |
| Republic of Guinea-Bissau | 95+ years | 0.78 (-11.24, 14.42) |
| Republic of Guinea | 30–34 years | 0.31 (-1.08, 1.71) |
| Republic of Guinea | 35–39 years | 0.28 (-0.67, 1.24) |
| Republic of Guinea | 40–44 years | 0.32 (-0.42, 1.07) |
| Republic of Guinea | 45–49 years | 0.39 (-0.23, 1.01) |
| Republic of Guinea | 50–54 years | 0.43 (-0.09, 0.96) |
| Republic of Guinea | 55–59 years | 0.45 (0.01, 0.89) |
| Republic of Guinea | 60–64 years | 0.48 (0.11, 0.85) |
| Republic of Guinea | 65–69 years | 0.51 (0.20, 0.82) |
| Republic of Guinea | 70–74 years | 0.54 (0.27, 0.81) |
| Republic of Guinea | 75–79 years | 0.57 (0.30, 0.84) |
| Republic of Guinea | 80–84 years | 0.59 (0.25, 0.93) |
| Republic of Guinea | 85–89 years | 0.60 (0.07, 1.12) |
| Republic of Guinea | 90–94 years | 0.57 (-0.42, 1.58) |
| Republic of Guinea | 95+ years | 0.56 (-1.76, 2.94) |
| Republic of Guyana | 30–34 years | 0.10 (-3.89, 4.27) |
| Republic of Guyana | 35–39 years | 0.25 (-2.12, 2.68) |
| Republic of Guyana | 40–44 years | 0.30 (-1.35, 1.97) |
| Republic of Guyana | 45–49 years | 0.31 (-0.98, 1.62) |
| Republic of Guyana | 50–54 years | 0.34 (-0.76, 1.45) |
| Republic of Guyana | 55–59 years | 0.38 (-0.61, 1.39) |
| Republic of Guyana | 60–64 years | 0.43 (-0.52, 1.38) |
| Republic of Guyana | 65–69 years | 0.46 (-0.45, 1.38) |
| Republic of Guyana | 70–74 years | 0.46 (-0.47, 1.40) |
| Republic of Guyana | 75–79 years | 0.43 (-0.63, 1.50) |
| Republic of Guyana | 80–84 years | 0.39 (-0.95, 1.75) |
| Republic of Guyana | 85–89 years | 0.36 (-1.46, 2.23) |
| Republic of Guyana | 90–94 years | 0.30 (-2.78, 3.49) |
| Republic of Guyana | 95+ years | 0.15 (-6.65, 7.45) |
| Republic of Haiti | 30–34 years | 0.06 (-0.94, 1.08) |
| Republic of Haiti | 35–39 years | 0.09 (-0.55, 0.74) |
| Republic of Haiti | 40–44 years | 0.11 (-0.38, 0.60) |
| Republic of Haiti | 45–49 years | 0.14 (-0.26, 0.55) |
| Republic of Haiti | 50–54 years | 0.20 (-0.15, 0.56) |
| Republic of Haiti | 55–59 years | 0.28 (-0.04, 0.60) |
| Republic of Haiti | 60–64 years | 0.36 (0.05, 0.66) |
| Republic of Haiti | 65–69 years | 0.41 (0.12, 0.71) |
| Republic of Haiti | 70–74 years | 0.45 (0.14, 0.76) |
| Republic of Haiti | 75–79 years | 0.44 (0.07, 0.80) |
| Republic of Haiti | 80–84 years | 0.42 (-0.09, 0.93) |
| Republic of Haiti | 85–89 years | 0.40 (-0.45, 1.26) |
| Republic of Haiti | 90–94 years | 0.40 (-1.32, 2.16) |
| Republic of Haiti | 95+ years | 0.39 (-3.75, 4.71) |
| Republic of Honduras | 30–34 years | 0.19 (-1.01, 1.40) |
| Republic of Honduras | 35–39 years | 0.34 (-0.41, 1.10) |
| Republic of Honduras | 40–44 years | 0.54 (-0.01, 1.09) |
| Republic of Honduras | 45–49 years | 0.74 (0.30, 1.18) |
| Republic of Honduras | 50–54 years | 0.91 (0.53, 1.28) |
| Republic of Honduras | 55–59 years | 1.04 (0.70, 1.37) |
| Republic of Honduras | 60–64 years | 1.16 (0.85, 1.46) |
| Republic of Honduras | 65–69 years | 1.27 (0.99, 1.55) |
| Republic of Honduras | 70–74 years | 1.38 (1.11, 1.65) |
| Republic of Honduras | 75–79 years | 1.48 (1.19, 1.77) |
| Republic of Honduras | 80–84 years | 1.55 (1.19, 1.91) |
| Republic of Honduras | 85–89 years | 1.53 (0.99, 2.07) |
| Republic of Honduras | 90–94 years | 1.44 (0.45, 2.44) |
| Republic of Honduras | 95+ years | 1.24 (-1.24, 3.80) |
| Republic of Iceland | 30–34 years | 0.62 (-4.67, 6.21) |
| Republic of Iceland | 35–39 years | 0.63 (-2.76, 4.14) |
| Republic of Iceland | 40–44 years | 0.66 (-1.80, 3.19) |
| Republic of Iceland | 45–49 years | 0.68 (-1.15, 2.55) |
| Republic of Iceland | 50–54 years | 0.71 (-0.69, 2.12) |
| Republic of Iceland | 55–59 years | 0.74 (-0.34, 1.84) |
| Republic of Iceland | 60–64 years | 0.77 (-0.08, 1.63) |
| Republic of Iceland | 65–69 years | 0.80 (0.12, 1.48) |
| Republic of Iceland | 70–74 years | 0.85 (0.27, 1.42) |
| Republic of Iceland | 75–79 years | 0.92 (0.38, 1.47) |
| Republic of Iceland | 80–84 years | 1.05 (0.45, 1.64) |
| Republic of Iceland | 85–89 years | 1.17 (0.39, 1.97) |
| Republic of Iceland | 90–94 years | 1.22 (-0.07, 2.52) |
| Republic of Iceland | 95+ years | 1.19 (-1.29, 3.72) |
| Republic of India | 30–34 years | 0.56 (0.39, 0.73) |
| Republic of India | 35–39 years | 0.72 (0.61, 0.83) |
| Republic of India | 40–44 years | 0.88 (0.80, 0.96) |
| Republic of India | 45–49 years | 1.04 (0.97, 1.10) |
| Republic of India | 50–54 years | 1.15 (1.09, 1.20) |
| Republic of India | 55–59 years | 1.21 (1.16, 1.25) |
| Republic of India | 60–64 years | 1.23 (1.18, 1.27) |
| Republic of India | 65–69 years | 1.24 (1.21, 1.28) |
| Republic of India | 70–74 years | 1.31 (1.28, 1.35) |
| Republic of India | 75–79 years | 1.47 (1.43, 1.52) |
| Republic of India | 80–84 years | 1.72 (1.66, 1.78) |
| Republic of India | 85–89 years | 2.05 (1.94, 2.15) |
| Republic of India | 90–94 years | 2.24 (2.01, 2.47) |
| Republic of India | 95+ years | 2.21 (1.61, 2.81) |
| Republic of Indonesia | 30–34 years | 0.06 (-0.18, 0.29) |
| Republic of Indonesia | 35–39 years | 0.05 (-0.10, 0.20) |
| Republic of Indonesia | 40–44 years | 0.12 (0.01, 0.23) |
| Republic of Indonesia | 45–49 years | 0.26 (0.18, 0.35) |
| Republic of Indonesia | 50–54 years | 0.44 (0.37, 0.51) |
| Republic of Indonesia | 55–59 years | 0.62 (0.56, 0.68) |
| Republic of Indonesia | 60–64 years | 0.79 (0.74, 0.85) |
| Republic of Indonesia | 65–69 years | 0.94 (0.89, 0.99) |
| Republic of Indonesia | 70–74 years | 1.07 (1.02, 1.12) |
| Republic of Indonesia | 75–79 years | 1.16 (1.10, 1.21) |
| Republic of Indonesia | 80–84 years | 1.20 (1.12, 1.27) |
| Republic of Indonesia | 85–89 years | 1.17 (1.06, 1.29) |
| Republic of Indonesia | 90–94 years | 1.06 (0.85, 1.27) |
| Republic of Indonesia | 95+ years | 0.89 (0.42, 1.36) |
| Republic of Iraq | 30–34 years | 0.52 (-0.21, 1.26) |
| Republic of Iraq | 35–39 years | 0.81 (0.35, 1.27) |
| Republic of Iraq | 40–44 years | 1.03 (0.70, 1.37) |
| Republic of Iraq | 45–49 years | 1.19 (0.92, 1.46) |
| Republic of Iraq | 50–54 years | 1.31 (1.08, 1.55) |
| Republic of Iraq | 55–59 years | 1.44 (1.23, 1.65) |
| Republic of Iraq | 60–64 years | 1.59 (1.40, 1.78) |
| Republic of Iraq | 65–69 years | 1.76 (1.58, 1.94) |
| Republic of Iraq | 70–74 years | 1.90 (1.73, 2.07) |
| Republic of Iraq | 75–79 years | 1.95 (1.78, 2.12) |
| Republic of Iraq | 80–84 years | 1.92 (1.72, 2.11) |
| Republic of Iraq | 85–89 years | 1.90 (1.62, 2.18) |
| Republic of Iraq | 90–94 years | 1.89 (1.43, 2.36) |
| Republic of Iraq | 95+ years | 1.86 (0.98, 2.76) |
| Republic of Italy | 30–34 years | 1.25 (-1.13, 3.69) |
| Republic of Italy | 35–39 years | 0.90 (-0.57, 2.40) |
| Republic of Italy | 40–44 years | 0.54 (-0.49, 1.58) |
| Republic of Italy | 45–49 years | 0.14 (-0.63, 0.91) |
| Republic of Italy | 50–54 years | -0.29 (-0.88, 0.31) |
| Republic of Italy | 55–59 years | -0.84 (-1.30, -0.37) |
| Republic of Italy | 60–64 years | -1.53 (-1.89, -1.17) |
| Republic of Italy | 65–69 years | -2.20 (-2.47, -1.93) |
| Republic of Italy | 70–74 years | -2.53 (-2.74, -2.32) |
| Republic of Italy | 75–79 years | -2.45 (-2.64, -2.27) |
| Republic of Italy | 80–84 years | -2.09 (-2.28, -1.91) |
| Republic of Italy | 85–89 years | -1.61 (-1.86, -1.35) |
| Republic of Italy | 90–94 years | -1.12 (-1.59, -0.65) |
| Republic of Italy | 95+ years | -0.77 (-1.94, 0.41) |
| Republic of Kazakhstan | 30–34 years | -0.10 (-1.08, 0.90) |
| Republic of Kazakhstan | 35–39 years | 0.09 (-0.53, 0.71) |
| Republic of Kazakhstan | 40–44 years | 0.17 (-0.27, 0.61) |
| Republic of Kazakhstan | 45–49 years | 0.23 (-0.11, 0.57) |
| Republic of Kazakhstan | 50–54 years | 0.34 (0.08, 0.61) |
| Republic of Kazakhstan | 55–59 years | 0.46 (0.26, 0.66) |
| Republic of Kazakhstan | 60–64 years | 0.59 (0.42, 0.76) |
| Republic of Kazakhstan | 65–69 years | 0.74 (0.60, 0.89) |
| Republic of Kazakhstan | 70–74 years | 0.92 (0.78, 1.06) |
| Republic of Kazakhstan | 75–79 years | 1.08 (0.93, 1.23) |
| Republic of Kazakhstan | 80–84 years | 1.26 (1.08, 1.44) |
| Republic of Kazakhstan | 85–89 years | 1.31 (1.03, 1.60) |
| Republic of Kazakhstan | 90–94 years | 1.22 (0.68, 1.76) |
| Republic of Kazakhstan | 95+ years | 0.88 (-0.47, 2.26) |
| Republic of Kenya | 30–34 years | 0.36 (-0.26, 0.99) |
| Republic of Kenya | 35–39 years | 0.40 (-0.03, 0.82) |
| Republic of Kenya | 40–44 years | 0.43 (0.10, 0.77) |
| Republic of Kenya | 45–49 years | 0.44 (0.15, 0.73) |
| Republic of Kenya | 50–54 years | 0.44 (0.18, 0.69) |
| Republic of Kenya | 55–59 years | 0.46 (0.23, 0.69) |
| Republic of Kenya | 60–64 years | 0.48 (0.28, 0.69) |
| Republic of Kenya | 65–69 years | 0.53 (0.34, 0.72) |
| Republic of Kenya | 70–74 years | 0.58 (0.40, 0.77) |
| Republic of Kenya | 75–79 years | 0.60 (0.40, 0.79) |
| Republic of Kenya | 80–84 years | 0.56 (0.31, 0.81) |
| Republic of Kenya | 85–89 years | 0.50 (0.13, 0.88) |
| Republic of Kenya | 90–94 years | 0.38 (-0.32, 1.08) |
| Republic of Kenya | 95+ years | 0.29 (-1.44, 2.04) |
| Republic of Kiribati | 30–34 years | 0.05 (-11.96, 13.70) |
| Republic of Kiribati | 35–39 years | 0.03 (-7.99, 8.75) |
| Republic of Kiribati | 40–44 years | -0.01 (-6.01, 6.37) |
| Republic of Kiribati | 45–49 years | -0.01 (-4.61, 4.81) |
| Republic of Kiribati | 50–54 years | 0.06 (-3.53, 3.79) |
| Republic of Kiribati | 55–59 years | 0.13 (-2.85, 3.20) |
| Republic of Kiribati | 60–64 years | 0.17 (-2.48, 2.90) |
| Republic of Kiribati | 65–69 years | 0.20 (-2.27, 2.74) |
| Republic of Kiribati | 70–74 years | 0.26 (-2.20, 2.78) |
| Republic of Kiribati | 75–79 years | 0.39 (-2.42, 3.28) |
| Republic of Kiribati | 80–84 years | 0.57 (-3.34, 4.64) |
| Republic of Kiribati | 85–89 years | 0.70 (-6.09, 7.98) |
| Republic of Kiribati | 90–94 years | 0.74 (-13.40, 17.19) |
| Republic of Kiribati | 95+ years | 0.76 (-29.08, 43.17) |
| Republic of Korea | 30–34 years | 0.66 (0.02, 1.30) |
| Republic of Korea | 35–39 years | 0.80 (0.44, 1.15) |
| Republic of Korea | 40–44 years | 0.94 (0.70, 1.18) |
| Republic of Korea | 45–49 years | 1.08 (0.91, 1.26) |
| Republic of Korea | 50–54 years | 1.23 (1.09, 1.36) |
| Republic of Korea | 55–59 years | 1.35 (1.24, 1.45) |
| Republic of Korea | 60–64 years | 1.45 (1.36, 1.54) |
| Republic of Korea | 65–69 years | 1.55 (1.47, 1.63) |
| Republic of Korea | 70–74 years | 1.61 (1.53, 1.69) |
| Republic of Korea | 75–79 years | 1.69 (1.60, 1.77) |
| Republic of Korea | 80–84 years | 1.84 (1.72, 1.95) |
| Republic of Korea | 85–89 years | 2.02 (1.83, 2.22) |
| Republic of Korea | 90–94 years | 2.21 (1.75, 2.66) |
| Republic of Korea | 95+ years | 2.35 (0.66, 4.07) |
| Republic of Latvia | 30–34 years | -0.34 (-3.68, 3.11) |
| Republic of Latvia | 35–39 years | -0.34 (-2.30, 1.66) |
| Republic of Latvia | 40–44 years | -0.36 (-1.64, 0.93) |
| Republic of Latvia | 45–49 years | -0.37 (-1.22, 0.49) |
| Republic of Latvia | 50–54 years | -0.35 (-0.95, 0.25) |
| Republic of Latvia | 55–59 years | -0.32 (-0.74, 0.11) |
| Republic of Latvia | 60–64 years | -0.24 (-0.56, 0.08) |
| Republic of Latvia | 65–69 years | -0.16 (-0.42, 0.09) |
| Republic of Latvia | 70–74 years | -0.18 (-0.40, 0.04) |
| Republic of Latvia | 75–79 years | -0.30 (-0.52, -0.07) |
| Republic of Latvia | 80–84 years | -0.45 (-0.71, -0.19) |
| Republic of Latvia | 85–89 years | -0.59 (-0.96, -0.23) |
| Republic of Latvia | 90–94 years | -0.62 (-1.33, 0.09) |
| Republic of Latvia | 95+ years | -0.57 (-2.43, 1.32) |
| Republic of Liberia | 30–34 years | 0.38 (-1.70, 2.50) |
| Republic of Liberia | 35–39 years | 0.39 (-0.99, 1.79) |
| Republic of Liberia | 40–44 years | 0.40 (-0.69, 1.50) |
| Republic of Liberia | 45–49 years | 0.41 (-0.54, 1.37) |
| Republic of Liberia | 50–54 years | 0.45 (-0.41, 1.31) |
| Republic of Liberia | 55–59 years | 0.45 (-0.30, 1.20) |
| Republic of Liberia | 60–64 years | 0.46 (-0.19, 1.11) |
| Republic of Liberia | 65–69 years | 0.46 (-0.10, 1.02) |
| Republic of Liberia | 70–74 years | 0.45 (-0.05, 0.96) |
| Republic of Liberia | 75–79 years | 0.49 (-0.00, 0.98) |
| Republic of Liberia | 80–84 years | 0.56 (-0.04, 1.17) |
| Republic of Liberia | 85–89 years | 0.64 (-0.32, 1.61) |
| Republic of Liberia | 90–94 years | 0.74 (-1.23, 2.76) |
| Republic of Liberia | 95+ years | 0.96 (-3.87, 6.04) |
| Republic of Lithuania | 30–34 years | -0.09 (-2.92, 2.83) |
| Republic of Lithuania | 35–39 years | -0.15 (-1.82, 1.55) |
| Republic of Lithuania | 40–44 years | -0.11 (-1.18, 0.97) |
| Republic of Lithuania | 45–49 years | -0.03 (-0.73, 0.68) |
| Republic of Lithuania | 50–54 years | 0.02 (-0.47, 0.51) |
| Republic of Lithuania | 55–59 years | 0.00 (-0.35, 0.35) |
| Republic of Lithuania | 60–64 years | -0.07 (-0.34, 0.20) |
| Republic of Lithuania | 65–69 years | -0.16 (-0.38, 0.06) |
| Republic of Lithuania | 70–74 years | -0.21 (-0.41, -0.02) |
| Republic of Lithuania | 75–79 years | -0.22 (-0.42, -0.01) |
| Republic of Lithuania | 80–84 years | -0.19 (-0.43, 0.04) |
| Republic of Lithuania | 85–89 years | -0.27 (-0.60, 0.06) |
| Republic of Lithuania | 90–94 years | -0.42 (-1.03, 0.20) |
| Republic of Lithuania | 95+ years | -0.72 (-2.21, 0.80) |
| Republic of Madagascar | 30–34 years | 0.21 (-0.70, 1.12) |
| Republic of Madagascar | 35–39 years | 0.19 (-0.43, 0.80) |
| Republic of Madagascar | 40–44 years | 0.21 (-0.27, 0.70) |
| Republic of Madagascar | 45–49 years | 0.25 (-0.16, 0.67) |
| Republic of Madagascar | 50–54 years | 0.32 (-0.04, 0.69) |
| Republic of Madagascar | 55–59 years | 0.40 (0.07, 0.74) |
| Republic of Madagascar | 60–64 years | 0.48 (0.17, 0.78) |
| Republic of Madagascar | 65–69 years | 0.54 (0.26, 0.83) |
| Republic of Madagascar | 70–74 years | 0.57 (0.29, 0.86) |
| Republic of Madagascar | 75–79 years | 0.57 (0.25, 0.89) |
| Republic of Madagascar | 80–84 years | 0.62 (0.20, 1.04) |
| Republic of Madagascar | 85–89 years | 0.66 (0.03, 1.29) |
| Republic of Madagascar | 90–94 years | 0.69 (-0.46, 1.85) |
| Republic of Madagascar | 95+ years | 0.78 (-1.81, 3.43) |
| Republic of Malawi | 30–34 years | 0.04 (-1.03, 1.13) |
| Republic of Malawi | 35–39 years | 0.12 (-0.62, 0.86) |
| Republic of Malawi | 40–44 years | 0.19 (-0.40, 0.78) |
| Republic of Malawi | 45–49 years | 0.22 (-0.28, 0.72) |
| Republic of Malawi | 50–54 years | 0.22 (-0.22, 0.67) |
| Republic of Malawi | 55–59 years | 0.25 (-0.14, 0.65) |
| Republic of Malawi | 60–64 years | 0.31 (-0.03, 0.66) |
| Republic of Malawi | 65–69 years | 0.40 (0.09, 0.71) |
| Republic of Malawi | 70–74 years | 0.48 (0.18, 0.77) |
| Republic of Malawi | 75–79 years | 0.53 (0.19, 0.86) |
| Republic of Malawi | 80–84 years | 0.54 (0.08, 1.00) |
| Republic of Malawi | 85–89 years | 0.53 (-0.23, 1.30) |
| Republic of Malawi | 90–94 years | 0.53 (-0.98, 2.06) |
| Republic of Malawi | 95+ years | 0.57 (-2.92, 4.18) |
| Republic of Maldives | 30–34 years | 0.03 (-4.98, 5.31) |
| Republic of Maldives | 35–39 years | -0.11 (-3.68, 3.58) |
| Republic of Maldives | 40–44 years | -0.29 (-3.21, 2.71) |
| Republic of Maldives | 45–49 years | -0.38 (-2.81, 2.12) |
| Republic of Maldives | 50–54 years | -0.30 (-2.33, 1.77) |
| Republic of Maldives | 55–59 years | -0.09 (-1.79, 1.64) |
| Republic of Maldives | 60–64 years | 0.19 (-1.29, 1.70) |
| Republic of Maldives | 65–69 years | 0.48 (-0.90, 1.89) |
| Republic of Maldives | 70–74 years | 0.70 (-0.66, 2.07) |
| Republic of Maldives | 75–79 years | 0.84 (-0.65, 2.34) |
| Republic of Maldives | 80–84 years | 0.93 (-1.08, 2.98) |
| Republic of Maldives | 85–89 years | 0.99 (-2.25, 4.34) |
| Republic of Maldives | 90–94 years | 0.97 (-5.76, 8.17) |
| Republic of Maldives | 95+ years | 0.91 (-16.60, 22.09) |
| Republic of Mali | 30–34 years | 0.06 (-1.08, 1.21) |
| Republic of Mali | 35–39 years | 0.10 (-0.68, 0.89) |
| Republic of Mali | 40–44 years | 0.15 (-0.46, 0.77) |
| Republic of Mali | 45–49 years | 0.18 (-0.33, 0.70) |
| Republic of Mali | 50–54 years | 0.19 (-0.25, 0.63) |
| Republic of Mali | 55–59 years | 0.21 (-0.16, 0.59) |
| Republic of Mali | 60–64 years | 0.28 (-0.05, 0.60) |
| Republic of Mali | 65–69 years | 0.37 (0.09, 0.66) |
| Republic of Mali | 70–74 years | 0.45 (0.17, 0.72) |
| Republic of Mali | 75–79 years | 0.47 (0.18, 0.77) |
| Republic of Mali | 80–84 years | 0.46 (0.07, 0.85) |
| Republic of Mali | 85–89 years | 0.44 (-0.23, 1.11) |
| Republic of Mali | 90–94 years | 0.48 (-0.89, 1.87) |
| Republic of Mali | 95+ years | 0.63 (-2.53, 3.89) |
| Republic of Malta | 30–34 years | 0.43 (-4.34, 5.44) |
| Republic of Malta | 35–39 years | 0.51 (-2.51, 3.62) |
| Republic of Malta | 40–44 years | 0.59 (-1.58, 2.79) |
| Republic of Malta | 45–49 years | 0.65 (-0.97, 2.29) |
| Republic of Malta | 50–54 years | 0.72 (-0.51, 1.96) |
| Republic of Malta | 55–59 years | 0.82 (-0.10, 1.74) |
| Republic of Malta | 60–64 years | 0.97 (0.26, 1.68) |
| Republic of Malta | 65–69 years | 1.10 (0.54, 1.67) |
| Republic of Malta | 70–74 years | 1.17 (0.70, 1.65) |
| Republic of Malta | 75–79 years | 1.17 (0.70, 1.65) |
| Republic of Malta | 80–84 years | 1.14 (0.60, 1.68) |
| Republic of Malta | 85–89 years | 1.13 (0.38, 1.89) |
| Republic of Malta | 90–94 years | 1.11 (-0.27, 2.50) |
| Republic of Malta | 95+ years | 1.01 (-2.18, 4.31) |
| Republic of Mauritius | 30–34 years | 0.00 (-3.23, 3.34) |
| Republic of Mauritius | 35–39 years | 0.08 (-1.83, 2.03) |
| Republic of Mauritius | 40–44 years | 0.13 (-1.22, 1.49) |
| Republic of Mauritius | 45–49 years | 0.19 (-0.84, 1.23) |
| Republic of Mauritius | 50–54 years | 0.29 (-0.50, 1.10) |
| Republic of Mauritius | 55–59 years | 0.41 (-0.25, 1.07) |
| Republic of Mauritius | 60–64 years | 0.47 (-0.10, 1.03) |
| Republic of Mauritius | 65–69 years | 0.47 (-0.04, 0.97) |
| Republic of Mauritius | 70–74 years | 0.47 (-0.01, 0.96) |
| Republic of Mauritius | 75–79 years | 0.52 (-0.01, 1.06) |
| Republic of Mauritius | 80–84 years | 0.63 (-0.04, 1.31) |
| Republic of Mauritius | 85–89 years | 0.78 (-0.22, 1.78) |
| Republic of Mauritius | 90–94 years | 0.83 (-0.89, 2.58) |
| Republic of Mauritius | 95+ years | 0.67 (-2.81, 4.27) |
| Republic of Moldova | 30–34 years | -0.33 (-2.51, 1.89) |
| Republic of Moldova | 35–39 years | -0.36 (-1.65, 0.94) |
| Republic of Moldova | 40–44 years | -0.34 (-1.23, 0.55) |
| Republic of Moldova | 45–49 years | -0.30 (-0.95, 0.34) |
| Republic of Moldova | 50–54 years | -0.28 (-0.75, 0.19) |
| Republic of Moldova | 55–59 years | -0.30 (-0.64, 0.04) |
| Republic of Moldova | 60–64 years | -0.39 (-0.65, -0.12) |
| Republic of Moldova | 65–69 years | -0.54 (-0.76, -0.32) |
| Republic of Moldova | 70–74 years | -0.72 (-0.93, -0.50) |
| Republic of Moldova | 75–79 years | -0.91 (-1.13, -0.69) |
| Republic of Moldova | 80–84 years | -1.09 (-1.36, -0.82) |
| Republic of Moldova | 85–89 years | -1.21 (-1.65, -0.77) |
| Republic of Moldova | 90–94 years | -1.23 (-2.17, -0.29) |
| Republic of Moldova | 95+ years | -1.12 (-3.97, 1.82) |
| Republic of Mozambique | 30–34 years | 0.29 (-0.62, 1.20) |
| Republic of Mozambique | 35–39 years | 0.31 (-0.30, 0.92) |
| Republic of Mozambique | 40–44 years | 0.34 (-0.14, 0.82) |
| Republic of Mozambique | 45–49 years | 0.37 (-0.03, 0.77) |
| Republic of Mozambique | 50–54 years | 0.41 (0.05, 0.76) |
| Republic of Mozambique | 55–59 years | 0.42 (0.11, 0.74) |
| Republic of Mozambique | 60–64 years | 0.43 (0.15, 0.71) |
| Republic of Mozambique | 65–69 years | 0.43 (0.17, 0.68) |
| Republic of Mozambique | 70–74 years | 0.44 (0.19, 0.69) |
| Republic of Mozambique | 75–79 years | 0.49 (0.21, 0.76) |
| Republic of Mozambique | 80–84 years | 0.51 (0.15, 0.87) |
| Republic of Mozambique | 85–89 years | 0.50 (-0.07, 1.07) |
| Republic of Mozambique | 90–94 years | 0.47 (-0.65, 1.61) |
| Republic of Mozambique | 95+ years | 0.50 (-2.29, 3.36) |
| Republic of Namibia | 30–34 years | 0.22 (-2.45, 2.97) |
| Republic of Namibia | 35–39 years | 0.31 (-1.49, 2.14) |
| Republic of Namibia | 40–44 years | 0.36 (-1.03, 1.78) |
| Republic of Namibia | 45–49 years | 0.38 (-0.77, 1.55) |
| Republic of Namibia | 50–54 years | 0.39 (-0.60, 1.40) |
| Republic of Namibia | 55–59 years | 0.44 (-0.43, 1.32) |
| Republic of Namibia | 60–64 years | 0.52 (-0.25, 1.29) |
| Republic of Namibia | 65–69 years | 0.60 (-0.08, 1.29) |
| Republic of Namibia | 70–74 years | 0.69 (0.05, 1.34) |
| Republic of Namibia | 75–79 years | 0.79 (0.09, 1.50) |
| Republic of Namibia | 80–84 years | 0.92 (-0.05, 1.90) |
| Republic of Namibia | 85–89 years | 1.08 (-0.62, 2.80) |
| Republic of Namibia | 90–94 years | 1.23 (-2.23, 4.81) |
| Republic of Namibia | 95+ years | 1.34 (-6.40, 9.72) |
| Republic of Nauru | 30–34 years | 0.18 (-32.17, 47.95) |
| Republic of Nauru | 35–39 years | 0.28 (-21.92, 28.79) |
| Republic of Nauru | 40–44 years | 0.35 (-16.36, 20.40) |
| Republic of Nauru | 45–49 years | 0.41 (-12.54, 15.28) |
| Republic of Nauru | 50–54 years | 0.49 (-10.06, 12.29) |
| Republic of Nauru | 55–59 years | 0.55 (-8.42, 10.41) |
| Republic of Nauru | 60–64 years | 0.55 (-7.25, 9.00) |
| Republic of Nauru | 65–69 years | 0.47 (-6.55, 8.02) |
| Republic of Nauru | 70–74 years | 0.36 (-6.55, 7.79) |
| Republic of Nauru | 75–79 years | 0.25 (-7.72, 8.91) |
| Republic of Nauru | 80–84 years | 0.15 (-10.73, 12.36) |
| Republic of Nauru | 85–89 years | 0.06 (-17.58, 21.48) |
| Republic of Nauru | 90–94 years | 0.04 (-34.42, 52.61) |
| Republic of Nauru | 95+ years | 0.17 (-70.77, 243.31) |
| Republic of Nicaragua | 30–34 years | 0.17 (-1.19, 1.55) |
| Republic of Nicaragua | 35–39 years | 0.33 (-0.51, 1.19) |
| Republic of Nicaragua | 40–44 years | 0.46 (-0.16, 1.09) |
| Republic of Nicaragua | 45–49 years | 0.60 (0.10, 1.10) |
| Republic of Nicaragua | 50–54 years | 0.71 (0.29, 1.14) |
| Republic of Nicaragua | 55–59 years | 0.78 (0.41, 1.16) |
| Republic of Nicaragua | 60–64 years | 0.82 (0.48, 1.16) |
| Republic of Nicaragua | 65–69 years | 0.84 (0.52, 1.16) |
| Republic of Nicaragua | 70–74 years | 0.88 (0.58, 1.18) |
| Republic of Nicaragua | 75–79 years | 0.98 (0.67, 1.29) |
| Republic of Nicaragua | 80–84 years | 1.16 (0.78, 1.53) |
| Republic of Nicaragua | 85–89 years | 1.34 (0.78, 1.89) |
| Republic of Nicaragua | 90–94 years | 1.44 (0.50, 2.38) |
| Republic of Nicaragua | 95+ years | 1.47 (-0.28, 3.25) |
| Republic of Niue | 30–34 years | 0.05 (-65.92, 193.71) |
| Republic of Niue | 35–39 years | 0.16 (-46.74, 88.39) |
| Republic of Niue | 40–44 years | 0.22 (-34.38, 53.05) |
| Republic of Niue | 45–49 years | 0.26 (-25.08, 34.16) |
| Republic of Niue | 50–54 years | 0.34 (-18.45, 23.47) |
| Republic of Niue | 55–59 years | 0.39 (-14.17, 17.41) |
| Republic of Niue | 60–64 years | 0.39 (-11.63, 14.03) |
| Republic of Niue | 65–69 years | 0.36 (-10.03, 11.95) |
| Republic of Niue | 70–74 years | 0.31 (-9.13, 10.72) |
| Republic of Niue | 75–79 years | 0.31 (-8.97, 10.53) |
| Republic of Niue | 80–84 years | 0.46 (-9.99, 12.13) |
| Republic of Niue | 85–89 years | 0.69 (-13.13, 16.71) |
| Republic of Niue | 90–94 years | 0.87 (-20.78, 28.44) |
| Republic of Niue | 95+ years | 0.97 (-38.43, 65.59) |
| Republic of Palau | 30–34 years | 0.37 (-23.87, 32.33) |
| Republic of Palau | 35–39 years | 0.22 (-14.53, 17.52) |
| Republic of Palau | 40–44 years | 0.12 (-10.02, 11.40) |
| Republic of Palau | 45–49 years | 0.08 (-7.30, 8.05) |
| Republic of Palau | 50–54 years | 0.09 (-5.76, 6.31) |
| Republic of Palau | 55–59 years | 0.12 (-4.81, 5.32) |
| Republic of Palau | 60–64 years | 0.11 (-4.16, 4.57) |
| Republic of Palau | 65–69 years | 0.06 (-3.80, 4.08) |
| Republic of Palau | 70–74 years | 0.05 (-3.68, 3.92) |
| Republic of Palau | 75–79 years | 0.12 (-3.90, 4.31) |
| Republic of Palau | 80–84 years | 0.33 (-4.76, 5.68) |
| Republic of Palau | 85–89 years | 0.71 (-7.47, 9.61) |
| Republic of Palau | 90–94 years | 1.06 (-15.45, 20.79) |
| Republic of Palau | 95+ years | 1.42 (-36.82, 62.80) |
| Republic of Panama | 30–34 years | 0.37 (-1.28, 2.06) |
| Republic of Panama | 35–39 years | 0.49 (-0.49, 1.49) |
| Republic of Panama | 40–44 years | 0.66 (-0.04, 1.36) |
| Republic of Panama | 45–49 years | 0.83 (0.29, 1.37) |
| Republic of Panama | 50–54 years | 0.94 (0.49, 1.38) |
| Republic of Panama | 55–59 years | 0.93 (0.54, 1.33) |
| Republic of Panama | 60–64 years | 0.87 (0.51, 1.23) |
| Republic of Panama | 65–69 years | 0.81 (0.48, 1.14) |
| Republic of Panama | 70–74 years | 0.80 (0.49, 1.11) |
| Republic of Panama | 75–79 years | 0.87 (0.55, 1.19) |
| Republic of Panama | 80–84 years | 0.98 (0.61, 1.35) |
| Republic of Panama | 85–89 years | 1.07 (0.57, 1.58) |
| Republic of Panama | 90–94 years | 1.14 (0.35, 1.94) |
| Republic of Panama | 95+ years | 1.20 (-0.19, 2.60) |
| Republic of Paraguay | 30–34 years | 0.59 (-0.74, 1.94) |
| Republic of Paraguay | 35–39 years | 0.69 (-0.13, 1.52) |
| Republic of Paraguay | 40–44 years | 0.83 (0.23, 1.44) |
| Republic of Paraguay | 45–49 years | 0.96 (0.48, 1.43) |
| Republic of Paraguay | 50–54 years | 1.01 (0.62, 1.41) |
| Republic of Paraguay | 55–59 years | 1.01 (0.66, 1.36) |
| Republic of Paraguay | 60–64 years | 0.96 (0.64, 1.29) |
| Republic of Paraguay | 65–69 years | 0.89 (0.59, 1.19) |
| Republic of Paraguay | 70–74 years | 0.79 (0.51, 1.08) |
| Republic of Paraguay | 75–79 years | 0.68 (0.39, 0.98) |
| Republic of Paraguay | 80–84 years | 0.57 (0.22, 0.92) |
| Republic of Paraguay | 85–89 years | 0.43 (-0.05, 0.92) |
| Republic of Paraguay | 90–94 years | 0.38 (-0.38, 1.15) |
| Republic of Paraguay | 95+ years | 0.38 (-0.93, 1.71) |
| Republic of Peru | 30–34 years | 0.12 (-0.38, 0.61) |
| Republic of Peru | 35–39 years | 0.40 (0.12, 0.68) |
| Republic of Peru | 40–44 years | 0.80 (0.61, 0.99) |
| Republic of Peru | 45–49 years | 1.20 (1.05, 1.35) |
| Republic of Peru | 50–54 years | 1.47 (1.35, 1.59) |
| Republic of Peru | 55–59 years | 1.61 (1.50, 1.72) |
| Republic of Peru | 60–64 years | 1.67 (1.57, 1.78) |
| Republic of Peru | 65–69 years | 1.69 (1.59, 1.80) |
| Republic of Peru | 70–74 years | 1.74 (1.64, 1.84) |
| Republic of Peru | 75–79 years | 1.81 (1.70, 1.92) |
| Republic of Peru | 80–84 years | 1.92 (1.79, 2.05) |
| Republic of Peru | 85–89 years | 2.04 (1.87, 2.22) |
| Republic of Peru | 90–94 years | 2.12 (1.84, 2.41) |
| Republic of Peru | 95+ years | 2.12 (1.59, 2.66) |
| Republic of Poland | 30–34 years | -2.15 (-4.01, -0.26) |
| Republic of Poland | 35–39 years | -2.11 (-3.20, -1.02) |
| Republic of Poland | 40–44 years | -1.72 (-2.46, -0.98) |
| Republic of Poland | 45–49 years | -1.11 (-1.63, -0.58) |
| Republic of Poland | 50–54 years | -0.53 (-0.91, -0.15) |
| Republic of Poland | 55–59 years | -0.09 (-0.36, 0.17) |
| Republic of Poland | 60–64 years | 0.21 (0.02, 0.40) |
| Republic of Poland | 65–69 years | 0.43 (0.28, 0.58) |
| Republic of Poland | 70–74 years | 0.57 (0.44, 0.71) |
| Republic of Poland | 75–79 years | 0.66 (0.51, 0.81) |
| Republic of Poland | 80–84 years | 0.68 (0.51, 0.85) |
| Republic of Poland | 85–89 years | 0.55 (0.30, 0.80) |
| Republic of Poland | 90–94 years | 0.12 (-0.40, 0.64) |
| Republic of Poland | 95+ years | -0.78 (-2.19, 0.66) |
| Republic of Rwanda | 30–34 years | -0.18 (-1.43, 1.08) |
| Republic of Rwanda | 35–39 years | -0.28 (-1.11, 0.55) |
| Republic of Rwanda | 40–44 years | -0.39 (-1.06, 0.28) |
| Republic of Rwanda | 45–49 years | -0.32 (-0.89, 0.25) |
| Republic of Rwanda | 50–54 years | -0.16 (-0.65, 0.34) |
| Republic of Rwanda | 55–59 years | 0.05 (-0.39, 0.48) |
| Republic of Rwanda | 60–64 years | 0.23 (-0.17, 0.63) |
| Republic of Rwanda | 65–69 years | 0.39 (0.01, 0.76) |
| Republic of Rwanda | 70–74 years | 0.52 (0.15, 0.89) |
| Republic of Rwanda | 75–79 years | 0.69 (0.27, 1.10) |
| Republic of Rwanda | 80–84 years | 0.86 (0.29, 1.43) |
| Republic of Rwanda | 85–89 years | 1.04 (0.04, 2.05) |
| Republic of Rwanda | 90–94 years | 1.21 (-0.92, 3.39) |
| Republic of Rwanda | 95+ years | 1.39 (-3.48, 6.50) |
| Republic of San Marino | 30–34 years | 0.22 (-18.12, 22.67) |
| Republic of San Marino | 35–39 years | 0.21 (-11.20, 13.09) |
| Republic of San Marino | 40–44 years | 0.19 (-7.64, 8.69) |
| Republic of San Marino | 45–49 years | 0.18 (-5.46, 6.16) |
| Republic of San Marino | 50–54 years | 0.15 (-4.13, 4.63) |
| Republic of San Marino | 55–59 years | 0.12 (-3.26, 3.62) |
| Republic of San Marino | 60–64 years | 0.11 (-2.57, 2.86) |
| Republic of San Marino | 65–69 years | 0.11 (-1.99, 2.26) |
| Republic of San Marino | 70–74 years | 0.14 (-1.57, 1.89) |
| Republic of San Marino | 75–79 years | 0.20 (-1.34, 1.77) |
| Republic of San Marino | 80–84 years | 0.34 (-1.21, 1.91) |
| Republic of San Marino | 85–89 years | 0.53 (-1.42, 2.52) |
| Republic of San Marino | 90–94 years | 0.71 (-2.37, 3.89) |
| Republic of San Marino | 95+ years | 0.77 (-5.02, 6.91) |
| Republic of Senegal | 30–34 years | 0.45 (-0.75, 1.66) |
| Republic of Senegal | 35–39 years | 0.48 (-0.33, 1.31) |
| Republic of Senegal | 40–44 years | 0.50 (-0.15, 1.15) |
| Republic of Senegal | 45–49 years | 0.49 (-0.05, 1.03) |
| Republic of Senegal | 50–54 years | 0.51 (0.06, 0.97) |
| Republic of Senegal | 55–59 years | 0.55 (0.16, 0.94) |
| Republic of Senegal | 60–64 years | 0.57 (0.24, 0.91) |
| Republic of Senegal | 65–69 years | 0.59 (0.30, 0.89) |
| Republic of Senegal | 70–74 years | 0.59 (0.33, 0.85) |
| Republic of Senegal | 75–79 years | 0.60 (0.34, 0.86) |
| Republic of Senegal | 80–84 years | 0.64 (0.31, 0.96) |
| Republic of Senegal | 85–89 years | 0.69 (0.18, 1.21) |
| Republic of Senegal | 90–94 years | 0.75 (-0.24, 1.74) |
| Republic of Senegal | 95+ years | 0.84 (-1.61, 3.35) |
| Republic of Serbia | 30–34 years | -0.11 (-1.90, 1.70) |
| Republic of Serbia | 35–39 years | -0.09 (-1.13, 0.97) |
| Republic of Serbia | 40–44 years | -0.06 (-0.76, 0.64) |
| Republic of Serbia | 45–49 years | -0.04 (-0.53, 0.46) |
| Republic of Serbia | 50–54 years | -0.02 (-0.37, 0.33) |
| Republic of Serbia | 55–59 years | 0.01 (-0.23, 0.25) |
| Republic of Serbia | 60–64 years | 0.08 (-0.10, 0.25) |
| Republic of Serbia | 65–69 years | 0.17 (0.03, 0.31) |
| Republic of Serbia | 70–74 years | 0.31 (0.19, 0.43) |
| Republic of Serbia | 75–79 years | 0.53 (0.40, 0.65) |
| Republic of Serbia | 80–84 years | 0.83 (0.62, 1.04) |
| Republic of Serbia | 85–89 years | 1.04 (0.75, 1.34) |
| Republic of Serbia | 90–94 years | 1.06 (0.52, 1.61) |
| Republic of Serbia | 95+ years | 0.92 (-1.27, 3.16) |
| Republic of Seychelles | 30–34 years | 0.18 (-10.34, 11.92) |
| Republic of Seychelles | 35–39 years | 0.19 (-6.35, 7.18) |
| Republic of Seychelles | 40–44 years | 0.17 (-4.58, 5.14) |
| Republic of Seychelles | 45–49 years | 0.16 (-3.52, 3.99) |
| Republic of Seychelles | 50–54 years | 0.24 (-2.74, 3.30) |
| Republic of Seychelles | 55–59 years | 0.39 (-2.06, 2.91) |
| Republic of Seychelles | 60–64 years | 0.57 (-1.52, 2.69) |
| Republic of Seychelles | 65–69 years | 0.65 (-1.19, 2.52) |
| Republic of Seychelles | 70–74 years | 0.66 (-1.03, 2.39) |
| Republic of Seychelles | 75–79 years | 0.70 (-0.99, 2.42) |
| Republic of Seychelles | 80–84 years | 0.84 (-1.17, 2.90) |
| Republic of Seychelles | 85–89 years | 1.02 (-1.84, 3.95) |
| Republic of Seychelles | 90–94 years | 1.17 (-3.59, 6.17) |
| Republic of Seychelles | 95+ years | 1.13 (-7.69, 10.80) |
| Republic of Sierra Leone | 30–34 years | 0.42 (-1.26, 2.13) |
| Republic of Sierra Leone | 35–39 years | 0.39 (-0.77, 1.56) |
| Republic of Sierra Leone | 40–44 years | 0.42 (-0.50, 1.35) |
| Republic of Sierra Leone | 45–49 years | 0.48 (-0.30, 1.27) |
| Republic of Sierra Leone | 50–54 years | 0.52 (-0.16, 1.20) |
| Republic of Sierra Leone | 55–59 years | 0.53 (-0.06, 1.12) |
| Republic of Sierra Leone | 60–64 years | 0.51 (-0.01, 1.02) |
| Republic of Sierra Leone | 65–69 years | 0.45 (0.01, 0.90) |
| Republic of Sierra Leone | 70–74 years | 0.42 (0.04, 0.81) |
| Republic of Sierra Leone | 75–79 years | 0.46 (0.09, 0.83) |
| Republic of Sierra Leone | 80–84 years | 0.54 (0.10, 0.99) |
| Republic of Sierra Leone | 85–89 years | 0.64 (-0.07, 1.35) |
| Republic of Sierra Leone | 90–94 years | 0.70 (-0.71, 2.13) |
| Republic of Sierra Leone | 95+ years | 0.71 (-2.40, 3.91) |
| Republic of Singapore | 30–34 years | 0.55 (-1.16, 2.29) |
| Republic of Singapore | 35–39 years | 0.55 (-0.45, 1.55) |
| Republic of Singapore | 40–44 years | 0.50 (-0.23, 1.23) |
| Republic of Singapore | 45–49 years | 0.47 (-0.09, 1.04) |
| Republic of Singapore | 50–54 years | 0.45 (0.00, 0.90) |
| Republic of Singapore | 55–59 years | 0.48 (0.11, 0.85) |
| Republic of Singapore | 60–64 years | 0.55 (0.23, 0.86) |
| Republic of Singapore | 65–69 years | 0.65 (0.37, 0.93) |
| Republic of Singapore | 70–74 years | 0.72 (0.45, 0.99) |
| Republic of Singapore | 75–79 years | 0.82 (0.53, 1.11) |
| Republic of Singapore | 80–84 years | 1.05 (0.70, 1.40) |
| Republic of Singapore | 85–89 years | 1.25 (0.72, 1.78) |
| Republic of Singapore | 90–94 years | 1.53 (0.53, 2.55) |
| Republic of Singapore | 95+ years | 1.88 (-0.70, 4.52) |
| Republic of Slovenia | 30–34 years | -0.12 (-3.73, 3.62) |
| Republic of Slovenia | 35–39 years | -0.17 (-2.23, 1.94) |
| Republic of Slovenia | 40–44 years | -0.20 (-1.57, 1.18) |
| Republic of Slovenia | 45–49 years | -0.21 (-1.15, 0.74) |
| Republic of Slovenia | 50–54 years | -0.16 (-0.82, 0.49) |
| Republic of Slovenia | 55–59 years | -0.06 (-0.52, 0.42) |
| Republic of Slovenia | 60–64 years | 0.07 (-0.28, 0.41) |
| Republic of Slovenia | 65–69 years | 0.15 (-0.12, 0.42) |
| Republic of Slovenia | 70–74 years | 0.17 (-0.07, 0.41) |
| Republic of Slovenia | 75–79 years | 0.15 (-0.10, 0.40) |
| Republic of Slovenia | 80–84 years | 0.14 (-0.13, 0.41) |
| Republic of Slovenia | 85–89 years | 0.14 (-0.25, 0.54) |
| Republic of Slovenia | 90–94 years | 0.13 (-0.61, 0.87) |
| Republic of Slovenia | 95+ years | 0.02 (-1.78, 1.85) |
| Republic of South Africa | 30–34 years | 0.13 (-0.35, 0.62) |
| Republic of South Africa | 35–39 years | 0.16 (-0.17, 0.48) |
| Republic of South Africa | 40–44 years | 0.15 (-0.10, 0.40) |
| Republic of South Africa | 45–49 years | 0.15 (-0.05, 0.36) |
| Republic of South Africa | 50–54 years | 0.20 (0.02, 0.38) |
| Republic of South Africa | 55–59 years | 0.32 (0.17, 0.48) |
| Republic of South Africa | 60–64 years | 0.48 (0.34, 0.61) |
| Republic of South Africa | 65–69 years | 0.60 (0.47, 0.72) |
| Republic of South Africa | 70–74 years | 0.70 (0.58, 0.81) |
| Republic of South Africa | 75–79 years | 0.81 (0.69, 0.92) |
| Republic of South Africa | 80–84 years | 0.92 (0.78, 1.06) |
| Republic of South Africa | 85–89 years | 1.04 (0.85, 1.23) |
| Republic of South Africa | 90–94 years | 1.14 (0.80, 1.48) |
| Republic of South Africa | 95+ years | 1.11 (0.29, 1.94) |
| Republic of South Sudan | 30–34 years | -0.10 (-1.63, 1.45) |
| Republic of South Sudan | 35–39 years | -0.02 (-0.99, 0.97) |
| Republic of South Sudan | 40–44 years | 0.04 (-0.72, 0.81) |
| Republic of South Sudan | 45–49 years | 0.09 (-0.56, 0.74) |
| Republic of South Sudan | 50–54 years | 0.09 (-0.48, 0.67) |
| Republic of South Sudan | 55–59 years | 0.09 (-0.42, 0.61) |
| Republic of South Sudan | 60–64 years | 0.10 (-0.37, 0.57) |
| Republic of South Sudan | 65–69 years | 0.11 (-0.31, 0.54) |
| Republic of South Sudan | 70–74 years | 0.14 (-0.25, 0.53) |
| Republic of South Sudan | 75–79 years | 0.27 (-0.14, 0.68) |
| Republic of South Sudan | 80–84 years | 0.46 (-0.07, 0.98) |
| Republic of South Sudan | 85–89 years | 0.67 (-0.16, 1.50) |
| Republic of South Sudan | 90–94 years | 0.83 (-0.77, 2.47) |
| Republic of South Sudan | 95+ years | 0.90 (-2.79, 4.74) |
| Republic of Sudan | 30–34 years | 0.33 (-0.40, 1.06) |
| Republic of Sudan | 35–39 years | 0.47 (0.00, 0.93) |
| Republic of Sudan | 40–44 years | 0.61 (0.26, 0.95) |
| Republic of Sudan | 45–49 years | 0.73 (0.45, 1.00) |
| Republic of Sudan | 50–54 years | 0.88 (0.64, 1.12) |
| Republic of Sudan | 55–59 years | 1.04 (0.83, 1.25) |
| Republic of Sudan | 60–64 years | 1.18 (0.99, 1.37) |
| Republic of Sudan | 65–69 years | 1.28 (1.11, 1.45) |
| Republic of Sudan | 70–74 years | 1.35 (1.20, 1.50) |
| Republic of Sudan | 75–79 years | 1.45 (1.29, 1.61) |
| Republic of Sudan | 80–84 years | 1.61 (1.41, 1.82) |
| Republic of Sudan | 85–89 years | 1.76 (1.41, 2.11) |
| Republic of Sudan | 90–94 years | 1.83 (1.12, 2.54) |
| Republic of Sudan | 95+ years | 1.84 (0.12, 3.58) |
| Republic of Suriname | 30–34 years | 0.23 (-4.49, 5.19) |
| Republic of Suriname | 35–39 years | 0.37 (-2.49, 3.32) |
| Republic of Suriname | 40–44 years | 0.49 (-1.57, 2.60) |
| Republic of Suriname | 45–49 years | 0.61 (-0.99, 2.22) |
| Republic of Suriname | 50–54 years | 0.71 (-0.61, 2.05) |
| Republic of Suriname | 55–59 years | 0.78 (-0.39, 1.97) |
| Republic of Suriname | 60–64 years | 0.81 (-0.28, 1.92) |
| Republic of Suriname | 65–69 years | 0.83 (-0.22, 1.90) |
| Republic of Suriname | 70–74 years | 0.85 (-0.21, 1.92) |
| Republic of Suriname | 75–79 years | 0.87 (-0.29, 2.04) |
| Republic of Suriname | 80–84 years | 0.85 (-0.61, 2.33) |
| Republic of Suriname | 85–89 years | 0.74 (-1.22, 2.73) |
| Republic of Suriname | 90–94 years | 0.63 (-2.20, 3.55) |
| Republic of Suriname | 95+ years | 0.44 (-4.20, 5.31) |
| Republic of Tajikistan | 30–34 years | 0.04 (-1.51, 1.62) |
| Republic of Tajikistan | 35–39 years | 0.04 (-1.02, 1.11) |
| Republic of Tajikistan | 40–44 years | -0.02 (-0.83, 0.80) |
| Republic of Tajikistan | 45–49 years | -0.08 (-0.73, 0.56) |
| Republic of Tajikistan | 50–54 years | -0.12 (-0.64, 0.39) |
| Republic of Tajikistan | 55–59 years | -0.06 (-0.47, 0.35) |
| Republic of Tajikistan | 60–64 years | 0.07 (-0.27, 0.41) |
| Republic of Tajikistan | 65–69 years | 0.25 (-0.04, 0.55) |
| Republic of Tajikistan | 70–74 years | 0.54 (0.26, 0.81) |
| Republic of Tajikistan | 75–79 years | 0.84 (0.57, 1.12) |
| Republic of Tajikistan | 80–84 years | 1.07 (0.74, 1.39) |
| Republic of Tajikistan | 85–89 years | 1.14 (0.65, 1.64) |
| Republic of Tajikistan | 90–94 years | 1.03 (0.11, 1.96) |
| Republic of Tajikistan | 95+ years | 0.67 (-1.83, 3.24) |
| Republic of the Congo | 30–34 years | -0.21 (-2.10, 1.71) |
| Republic of the Congo | 35–39 years | -0.19 (-1.42, 1.06) |
| Republic of the Congo | 40–44 years | -0.09 (-1.03, 0.86) |
| Republic of the Congo | 45–49 years | 0.02 (-0.76, 0.81) |
| Republic of the Congo | 50–54 years | 0.13 (-0.54, 0.81) |
| Republic of the Congo | 55–59 years | 0.26 (-0.33, 0.86) |
| Republic of the Congo | 60–64 years | 0.38 (-0.16, 0.92) |
| Republic of the Congo | 65–69 years | 0.47 (-0.02, 0.96) |
| Republic of the Congo | 70–74 years | 0.55 (0.06, 1.04) |
| Republic of the Congo | 75–79 years | 0.63 (0.07, 1.20) |
| Republic of the Congo | 80–84 years | 0.73 (-0.09, 1.55) |
| Republic of the Congo | 85–89 years | 0.89 (-0.64, 2.45) |
| Republic of the Congo | 90–94 years | 1.11 (-2.76, 5.12) |
| Republic of the Congo | 95+ years | 1.32 (-11.66, 16.21) |
| Republic of the Gambia | 30–34 years | 0.55 (-2.56, 3.76) |
| Republic of the Gambia | 35–39 years | 0.54 (-1.63, 2.75) |
| Republic of the Gambia | 40–44 years | 0.58 (-1.17, 2.35) |
| Republic of the Gambia | 45–49 years | 0.63 (-0.86, 2.14) |
| Republic of the Gambia | 50–54 years | 0.69 (-0.60, 2.01) |
| Republic of the Gambia | 55–59 years | 0.75 (-0.38, 1.88) |
| Republic of the Gambia | 60–64 years | 0.78 (-0.19, 1.77) |
| Republic of the Gambia | 65–69 years | 0.81 (-0.05, 1.67) |
| Republic of the Gambia | 70–74 years | 0.81 (0.04, 1.60) |
| Republic of the Gambia | 75–79 years | 0.80 (0.03, 1.58) |
| Republic of the Gambia | 80–84 years | 0.85 (-0.13, 1.84) |
| Republic of the Gambia | 85–89 years | 0.92 (-0.70, 2.58) |
| Republic of the Gambia | 90–94 years | 0.98 (-2.59, 4.68) |
| Republic of the Gambia | 95+ years | 0.98 (-8.47, 11.40) |
| Republic of the Marshall Islands | 30–34 years | 0.29 (-15.35, 18.83) |
| Republic of the Marshall Islands | 35–39 years | 0.36 (-9.94, 11.83) |
| Republic of the Marshall Islands | 40–44 years | 0.38 (-7.34, 8.73) |
| Republic of the Marshall Islands | 45–49 years | 0.39 (-5.63, 6.79) |
| Republic of the Marshall Islands | 50–54 years | 0.40 (-4.56, 5.62) |
| Republic of the Marshall Islands | 55–59 years | 0.39 (-3.90, 4.87) |
| Republic of the Marshall Islands | 60–64 years | 0.36 (-3.48, 4.37) |
| Republic of the Marshall Islands | 65–69 years | 0.35 (-3.25, 4.08) |
| Republic of the Marshall Islands | 70–74 years | 0.38 (-3.23, 4.13) |
| Republic of the Marshall Islands | 75–79 years | 0.48 (-3.64, 4.77) |
| Republic of the Marshall Islands | 80–84 years | 0.62 (-4.81, 6.36) |
| Republic of the Marshall Islands | 85–89 years | 0.93 (-8.02, 10.74) |
| Republic of the Marshall Islands | 90–94 years | 1.30 (-17.32, 24.12) |
| Republic of the Marshall Islands | 95+ years | 1.42 (-40.58, 73.11) |
| Republic of the Niger | 30–34 years | 0.15 (-1.00, 1.30) |
| Republic of the Niger | 35–39 years | 0.05 (-0.74, 0.83) |
| Republic of the Niger | 40–44 years | 0.07 (-0.56, 0.70) |
| Republic of the Niger | 45–49 years | 0.16 (-0.38, 0.70) |
| Republic of the Niger | 50–54 years | 0.24 (-0.22, 0.71) |
| Republic of the Niger | 55–59 years | 0.28 (-0.12, 0.69) |
| Republic of the Niger | 60–64 years | 0.29 (-0.07, 0.65) |
| Republic of the Niger | 65–69 years | 0.32 (-0.00, 0.65) |
| Republic of the Niger | 70–74 years | 0.42 (0.11, 0.73) |
| Republic of the Niger | 75–79 years | 0.54 (0.20, 0.88) |
| Republic of the Niger | 80–84 years | 0.63 (0.18, 1.08) |
| Republic of the Niger | 85–89 years | 0.70 (-0.05, 1.45) |
| Republic of the Niger | 90–94 years | 0.76 (-0.83, 2.37) |
| Republic of the Niger | 95+ years | 0.91 (-3.11, 5.10) |
| Republic of the Philippines | 30–34 years | 0.35 (-0.03, 0.73) |
| Republic of the Philippines | 35–39 years | 0.32 (0.08, 0.57) |
| Republic of the Philippines | 40–44 years | 0.32 (0.13, 0.50) |
| Republic of the Philippines | 45–49 years | 0.36 (0.22, 0.51) |
| Republic of the Philippines | 50–54 years | 0.45 (0.32, 0.57) |
| Republic of the Philippines | 55–59 years | 0.53 (0.42, 0.64) |
| Republic of the Philippines | 60–64 years | 0.58 (0.48, 0.67) |
| Republic of the Philippines | 65–69 years | 0.58 (0.49, 0.66) |
| Republic of the Philippines | 70–74 years | 0.53 (0.45, 0.62) |
| Republic of the Philippines | 75–79 years | 0.45 (0.35, 0.54) |
| Republic of the Philippines | 80–84 years | 0.35 (0.24, 0.47) |
| Republic of the Philippines | 85–89 years | 0.22 (0.06, 0.38) |
| Republic of the Philippines | 90–94 years | 0.01 (-0.30, 0.33) |
| Republic of the Philippines | 95+ years | -0.13 (-1.50, 1.27) |
| Republic of the Union of Myanmar | 30–34 years | 0.21 (-0.32, 0.75) |
| Republic of the Union of Myanmar | 35–39 years | 0.23 (-0.11, 0.57) |
| Republic of the Union of Myanmar | 40–44 years | 0.27 (0.02, 0.52) |
| Republic of the Union of Myanmar | 45–49 years | 0.35 (0.16, 0.55) |
| Republic of the Union of Myanmar | 50–54 years | 0.50 (0.34, 0.66) |
| Republic of the Union of Myanmar | 55–59 years | 0.68 (0.54, 0.81) |
| Republic of the Union of Myanmar | 60–64 years | 0.83 (0.71, 0.94) |
| Republic of the Union of Myanmar | 65–69 years | 0.97 (0.86, 1.07) |
| Republic of the Union of Myanmar | 70–74 years | 1.12 (1.02, 1.22) |
| Republic of the Union of Myanmar | 75–79 years | 1.25 (1.13, 1.36) |
| Republic of the Union of Myanmar | 80–84 years | 1.36 (1.20, 1.52) |
| Republic of the Union of Myanmar | 85–89 years | 1.54 (1.26, 1.82) |
| Republic of the Union of Myanmar | 90–94 years | 1.69 (1.11, 2.27) |
| Republic of the Union of Myanmar | 95+ years | 1.50 (0.24, 2.77) |
| Republic of Trinidad and Tobago | 30–34 years | 0.15 (-2.62, 3.01) |
| Republic of Trinidad and Tobago | 35–39 years | 0.23 (-1.40, 1.89) |
| Republic of Trinidad and Tobago | 40–44 years | 0.29 (-0.89, 1.48) |
| Republic of Trinidad and Tobago | 45–49 years | 0.35 (-0.56, 1.27) |
| Republic of Trinidad and Tobago | 50–54 years | 0.43 (-0.31, 1.17) |
| Republic of Trinidad and Tobago | 55–59 years | 0.51 (-0.13, 1.16) |
| Republic of Trinidad and Tobago | 60–64 years | 0.57 (-0.02, 1.17) |
| Republic of Trinidad and Tobago | 65–69 years | 0.61 (0.05, 1.18) |
| Republic of Trinidad and Tobago | 70–74 years | 0.64 (0.09, 1.19) |
| Republic of Trinidad and Tobago | 75–79 years | 0.64 (0.06, 1.22) |
| Republic of Trinidad and Tobago | 80–84 years | 0.60 (-0.07, 1.28) |
| Republic of Trinidad and Tobago | 85–89 years | 0.45 (-0.50, 1.41) |
| Republic of Trinidad and Tobago | 90–94 years | 0.25 (-1.61, 2.15) |
| Republic of Trinidad and Tobago | 95+ years | 0.19 (-5.41, 6.12) |
| Republic of Tunisia | 30–34 years | 0.72 (-0.54, 1.99) |
| Republic of Tunisia | 35–39 years | 0.90 (0.16, 1.63) |
| Republic of Tunisia | 40–44 years | 1.06 (0.54, 1.58) |
| Republic of Tunisia | 45–49 years | 1.20 (0.80, 1.60) |
| Republic of Tunisia | 50–54 years | 1.32 (0.99, 1.64) |
| Republic of Tunisia | 55–59 years | 1.40 (1.13, 1.67) |
| Republic of Tunisia | 60–64 years | 1.43 (1.20, 1.66) |
| Republic of Tunisia | 65–69 years | 1.42 (1.21, 1.63) |
| Republic of Tunisia | 70–74 years | 1.38 (1.19, 1.57) |
| Republic of Tunisia | 75–79 years | 1.31 (1.12, 1.50) |
| Republic of Tunisia | 80–84 years | 1.26 (1.03, 1.48) |
| Republic of Tunisia | 85–89 years | 1.33 (0.96, 1.70) |
| Republic of Tunisia | 90–94 years | 1.45 (0.62, 2.28) |
| Republic of Tunisia | 95+ years | 1.58 (-0.85, 4.08) |
| Republic of Turkey | 30–34 years | 0.33 (-0.14, 0.81) |
| Republic of Turkey | 35–39 years | 0.41 (0.14, 0.69) |
| Republic of Turkey | 40–44 years | 0.55 (0.36, 0.74) |
| Republic of Turkey | 45–49 years | 0.73 (0.58, 0.88) |
| Republic of Turkey | 50–54 years | 0.91 (0.79, 1.03) |
| Republic of Turkey | 55–59 years | 1.09 (0.99, 1.19) |
| Republic of Turkey | 60–64 years | 1.26 (1.18, 1.35) |
| Republic of Turkey | 65–69 years | 1.44 (1.37, 1.52) |
| Republic of Turkey | 70–74 years | 1.62 (1.54, 1.69) |
| Republic of Turkey | 75–79 years | 1.77 (1.69, 1.85) |
| Republic of Turkey | 80–84 years | 1.87 (1.78, 1.96) |
| Republic of Turkey | 85–89 years | 1.96 (1.84, 2.09) |
| Republic of Turkey | 90–94 years | 2.00 (1.79, 2.22) |
| Republic of Turkey | 95+ years | 1.95 (1.46, 2.45) |
| Republic of Uganda | 30–34 years | 0.21 (-0.55, 0.98) |
| Republic of Uganda | 35–39 years | 0.28 (-0.25, 0.82) |
| Republic of Uganda | 40–44 years | 0.36 (-0.06, 0.79) |
| Republic of Uganda | 45–49 years | 0.42 (0.06, 0.79) |
| Republic of Uganda | 50–54 years | 0.44 (0.12, 0.76) |
| Republic of Uganda | 55–59 years | 0.44 (0.16, 0.72) |
| Republic of Uganda | 60–64 years | 0.48 (0.23, 0.73) |
| Republic of Uganda | 65–69 years | 0.58 (0.35, 0.81) |
| Republic of Uganda | 70–74 years | 0.68 (0.46, 0.91) |
| Republic of Uganda | 75–79 years | 0.77 (0.53, 1.01) |
| Republic of Uganda | 80–84 years | 0.80 (0.49, 1.11) |
| Republic of Uganda | 85–89 years | 0.77 (0.28, 1.27) |
| Republic of Uganda | 90–94 years | 0.75 (-0.22, 1.73) |
| Republic of Uganda | 95+ years | 0.80 (-1.61, 3.27) |
| Republic of Uzbekistan | 30–34 years | -0.18 (-0.97, 0.62) |
| Republic of Uzbekistan | 35–39 years | -0.13 (-0.65, 0.38) |
| Republic of Uzbekistan | 40–44 years | -0.15 (-0.53, 0.23) |
| Republic of Uzbekistan | 45–49 years | -0.12 (-0.42, 0.18) |
| Republic of Uzbekistan | 50–54 years | -0.03 (-0.27, 0.21) |
| Republic of Uzbekistan | 55–59 years | 0.11 (-0.08, 0.30) |
| Republic of Uzbekistan | 60–64 years | 0.26 (0.11, 0.42) |
| Republic of Uzbekistan | 65–69 years | 0.39 (0.25, 0.53) |
| Republic of Uzbekistan | 70–74 years | 0.50 (0.36, 0.63) |
| Republic of Uzbekistan | 75–79 years | 0.59 (0.45, 0.73) |
| Republic of Uzbekistan | 80–84 years | 0.59 (0.42, 0.75) |
| Republic of Uzbekistan | 85–89 years | 0.46 (0.22, 0.70) |
| Republic of Uzbekistan | 90–94 years | 0.18 (-0.26, 0.62) |
| Republic of Uzbekistan | 95+ years | -0.08 (-1.34, 1.20) |
| Republic of Vanuatu | 30–34 years | 0.37 (-7.49, 8.90) |
| Republic of Vanuatu | 35–39 years | 0.41 (-4.85, 5.96) |
| Republic of Vanuatu | 40–44 years | 0.45 (-3.47, 4.53) |
| Republic of Vanuatu | 45–49 years | 0.49 (-2.55, 3.62) |
| Republic of Vanuatu | 50–54 years | 0.51 (-1.96, 3.03) |
| Republic of Vanuatu | 55–59 years | 0.47 (-1.61, 2.60) |
| Republic of Vanuatu | 60–64 years | 0.39 (-1.44, 2.25) |
| Republic of Vanuatu | 65–69 years | 0.29 (-1.38, 1.98) |
| Republic of Vanuatu | 70–74 years | 0.21 (-1.44, 1.89) |
| Republic of Vanuatu | 75–79 years | 0.18 (-1.73, 2.13) |
| Republic of Vanuatu | 80–84 years | 0.21 (-2.48, 2.96) |
| Republic of Vanuatu | 85–89 years | 0.25 (-4.41, 5.14) |
| Republic of Vanuatu | 90–94 years | 0.24 (-9.48, 11.00) |
| Republic of Vanuatu | 95+ years | 0.28 (-22.81, 30.26) |
| Republic of Yemen | 30–34 years | 0.74 (-0.14, 1.64) |
| Republic of Yemen | 35–39 years | 0.82 (0.25, 1.40) |
| Republic of Yemen | 40–44 years | 0.93 (0.48, 1.37) |
| Republic of Yemen | 45–49 years | 1.02 (0.65, 1.39) |
| Republic of Yemen | 50–54 years | 1.09 (0.78, 1.41) |
| Republic of Yemen | 55–59 years | 1.14 (0.86, 1.42) |
| Republic of Yemen | 60–64 years | 1.18 (0.93, 1.43) |
| Republic of Yemen | 65–69 years | 1.24 (1.01, 1.46) |
| Republic of Yemen | 70–74 years | 1.36 (1.14, 1.58) |
| Republic of Yemen | 75–79 years | 1.52 (1.27, 1.77) |
| Republic of Yemen | 80–84 years | 1.71 (1.37, 2.05) |
| Republic of Yemen | 85–89 years | 1.86 (1.26, 2.45) |
| Republic of Yemen | 90–94 years | 1.86 (0.63, 3.10) |
| Republic of Yemen | 95+ years | 1.71 (-1.10, 4.60) |
| Republic of Zambia | 30–34 years | -0.04 (-1.14, 1.06) |
| Republic of Zambia | 35–39 years | 0.09 (-0.66, 0.85) |
| Republic of Zambia | 40–44 years | 0.19 (-0.40, 0.79) |
| Republic of Zambia | 45–49 years | 0.27 (-0.24, 0.79) |
| Republic of Zambia | 50–54 years | 0.34 (-0.11, 0.79) |
| Republic of Zambia | 55–59 years | 0.40 (-0.01, 0.81) |
| Republic of Zambia | 60–64 years | 0.45 (0.08, 0.82) |
| Republic of Zambia | 65–69 years | 0.50 (0.16, 0.84) |
| Republic of Zambia | 70–74 years | 0.52 (0.19, 0.85) |
| Republic of Zambia | 75–79 years | 0.55 (0.18, 0.92) |
| Republic of Zambia | 80–84 years | 0.63 (0.13, 1.12) |
| Republic of Zambia | 85–89 years | 0.76 (-0.05, 1.57) |
| Republic of Zambia | 90–94 years | 0.88 (-0.74, 2.54) |
| Republic of Zambia | 95+ years | 0.98 (-3.37, 5.52) |
| Republic of Zimbabwe | 30–34 years | 0.01 (-1.08, 1.10) |
| Republic of Zimbabwe | 35–39 years | 0.12 (-0.60, 0.84) |
| Republic of Zimbabwe | 40–44 years | 0.16 (-0.41, 0.73) |
| Republic of Zimbabwe | 45–49 years | 0.08 (-0.40, 0.57) |
| Republic of Zimbabwe | 50–54 years | -0.10 (-0.53, 0.32) |
| Republic of Zimbabwe | 55–59 years | -0.28 (-0.65, 0.10) |
| Republic of Zimbabwe | 60–64 years | -0.34 (-0.66, -0.01) |
| Republic of Zimbabwe | 65–69 years | -0.26 (-0.54, 0.02) |
| Republic of Zimbabwe | 70–74 years | -0.12 (-0.38, 0.14) |
| Republic of Zimbabwe | 75–79 years | -0.01 (-0.28, 0.26) |
| Republic of Zimbabwe | 80–84 years | 0.08 (-0.27, 0.43) |
| Republic of Zimbabwe | 85–89 years | 0.17 (-0.38, 0.73) |
| Republic of Zimbabwe | 90–94 years | 0.21 (-0.85, 1.28) |
| Republic of Zimbabwe | 95+ years | 0.19 (-2.34, 2.79) |
| Romania | 30–34 years | 0.21 (-0.95, 1.39) |
| Romania | 35–39 years | 0.27 (-0.40, 0.95) |
| Romania | 40–44 years | 0.31 (-0.12, 0.75) |
| Romania | 45–49 years | 0.35 (0.05, 0.66) |
| Romania | 50–54 years | 0.40 (0.17, 0.62) |
| Romania | 55–59 years | 0.42 (0.25, 0.59) |
| Romania | 60–64 years | 0.43 (0.31, 0.55) |
| Romania | 65–69 years | 0.45 (0.36, 0.55) |
| Romania | 70–74 years | 0.48 (0.40, 0.56) |
| Romania | 75–79 years | 0.47 (0.39, 0.56) |
| Romania | 80–84 years | 0.42 (0.33, 0.52) |
| Romania | 85–89 years | 0.35 (0.19, 0.52) |
| Romania | 90–94 years | 0.26 (-0.15, 0.67) |
| Romania | 95+ years | 0.28 (-1.22, 1.81) |
| Russian Federation | 30–34 years | -0.02 (-0.61, 0.57) |
| Russian Federation | 35–39 years | -0.04 (-0.39, 0.30) |
| Russian Federation | 40–44 years | -0.03 (-0.26, 0.21) |
| Russian Federation | 45–49 years | 0.02 (-0.15, 0.19) |
| Russian Federation | 50–54 years | 0.12 (-0.01, 0.24) |
| Russian Federation | 55–59 years | 0.19 (0.10, 0.28) |
| Russian Federation | 60–64 years | 0.24 (0.17, 0.31) |
| Russian Federation | 65–69 years | 0.21 (0.15, 0.26) |
| Russian Federation | 70–74 years | 0.12 (0.07, 0.18) |
| Russian Federation | 75–79 years | 0.00 (-0.06, 0.06) |
| Russian Federation | 80–84 years | -0.07 (-0.14, -0.00) |
| Russian Federation | 85–89 years | -0.09 (-0.20, 0.01) |
| Russian Federation | 90–94 years | -0.04 (-0.26, 0.18) |
| Russian Federation | 95+ years | 0.04 (-0.61, 0.68) |
| Saint Kitts and Nevis | 30–34 years | 0.05 (-12.58, 14.51) |
| Saint Kitts and Nevis | 35–39 years | 0.13 (-7.54, 8.43) |
| Saint Kitts and Nevis | 40–44 years | 0.24 (-5.25, 6.06) |
| Saint Kitts and Nevis | 45–49 years | 0.38 (-3.96, 4.93) |
| Saint Kitts and Nevis | 50–54 years | 0.47 (-3.25, 4.34) |
| Saint Kitts and Nevis | 55–59 years | 0.51 (-2.81, 3.94) |
| Saint Kitts and Nevis | 60–64 years | 0.56 (-2.45, 3.66) |
| Saint Kitts and Nevis | 65–69 years | 0.63 (-2.17, 3.52) |
| Saint Kitts and Nevis | 70–74 years | 0.72 (-1.96, 3.46) |
| Saint Kitts and Nevis | 75–79 years | 0.81 (-1.87, 3.56) |
| Saint Kitts and Nevis | 80–84 years | 0.87 (-2.28, 4.13) |
| Saint Kitts and Nevis | 85–89 years | 0.83 (-3.94, 5.85) |
| Saint Kitts and Nevis | 90–94 years | 0.61 (-8.95, 11.17) |
| Saint Kitts and Nevis | 95+ years | 0.26 (-20.92, 27.11) |
| Saint Lucia | 30–34 years | 0.17 (-7.58, 8.57) |
| Saint Lucia | 35–39 years | 0.24 (-4.35, 5.05) |
| Saint Lucia | 40–44 years | 0.29 (-2.94, 3.63) |
| Saint Lucia | 45–49 years | 0.32 (-2.16, 2.87) |
| Saint Lucia | 50–54 years | 0.36 (-1.71, 2.47) |
| Saint Lucia | 55–59 years | 0.36 (-1.48, 2.23) |
| Saint Lucia | 60–64 years | 0.38 (-1.31, 2.09) |
| Saint Lucia | 65–69 years | 0.42 (-1.14, 2.01) |
| Saint Lucia | 70–74 years | 0.48 (-0.99, 1.97) |
| Saint Lucia | 75–79 years | 0.51 (-1.00, 2.04) |
| Saint Lucia | 80–84 years | 0.55 (-1.28, 2.42) |
| Saint Lucia | 85–89 years | 0.63 (-2.32, 3.67) |
| Saint Lucia | 90–94 years | 0.68 (-5.96, 7.79) |
| Saint Lucia | 95+ years | 0.50 (-13.87, 17.27) |
| Saint Vincent and the Grenadines | 30–34 years | 0.03 (-9.91, 11.08) |
| Saint Vincent and the Grenadines | 35–39 years | 0.13 (-5.72, 6.34) |
| Saint Vincent and the Grenadines | 40–44 years | 0.21 (-3.94, 4.54) |
| Saint Vincent and the Grenadines | 45–49 years | 0.32 (-2.91, 3.66) |
| Saint Vincent and the Grenadines | 50–54 years | 0.44 (-2.27, 3.22) |
| Saint Vincent and the Grenadines | 55–59 years | 0.55 (-1.83, 2.98) |
| Saint Vincent and the Grenadines | 60–64 years | 0.64 (-1.52, 2.85) |
| Saint Vincent and the Grenadines | 65–69 years | 0.71 (-1.31, 2.78) |
| Saint Vincent and the Grenadines | 70–74 years | 0.76 (-1.16, 2.73) |
| Saint Vincent and the Grenadines | 75–79 years | 0.78 (-1.15, 2.75) |
| Saint Vincent and the Grenadines | 80–84 years | 0.77 (-1.51, 3.09) |
| Saint Vincent and the Grenadines | 85–89 years | 0.69 (-2.73, 4.24) |
| Saint Vincent and the Grenadines | 90–94 years | 0.54 (-5.93, 7.45) |
| Saint Vincent and the Grenadines | 95+ years | 0.05 (-14.48, 17.05) |
| Slovak Republic | 30–34 years | -0.04 (-2.23, 2.21) |
| Slovak Republic | 35–39 years | -0.06 (-1.35, 1.24) |
| Slovak Republic | 40–44 years | -0.04 (-0.90, 0.83) |
| Slovak Republic | 45–49 years | -0.03 (-0.65, 0.60) |
| Slovak Republic | 50–54 years | -0.03 (-0.48, 0.42) |
| Slovak Republic | 55–59 years | -0.05 (-0.37, 0.28) |
| Slovak Republic | 60–64 years | -0.07 (-0.31, 0.17) |
| Slovak Republic | 65–69 years | -0.06 (-0.25, 0.13) |
| Slovak Republic | 70–74 years | 0.01 (-0.15, 0.18) |
| Slovak Republic | 75–79 years | 0.10 (-0.08, 0.28) |
| Slovak Republic | 80–84 years | 0.20 (-0.01, 0.41) |
| Slovak Republic | 85–89 years | 0.30 (-0.01, 0.62) |
| Slovak Republic | 90–94 years | 0.36 (-0.26, 0.98) |
| Slovak Republic | 95+ years | 0.33 (-1.26, 1.95) |
| Socialist Republic of Viet Nam | 30–34 years | 0.41 (0.05, 0.77) |
| Socialist Republic of Viet Nam | 35–39 years | 0.56 (0.33, 0.78) |
| Socialist Republic of Viet Nam | 40–44 years | 0.69 (0.52, 0.86) |
| Socialist Republic of Viet Nam | 45–49 years | 0.83 (0.69, 0.96) |
| Socialist Republic of Viet Nam | 50–54 years | 0.96 (0.85, 1.07) |
| Socialist Republic of Viet Nam | 55–59 years | 1.08 (0.99, 1.17) |
| Socialist Republic of Viet Nam | 60–64 years | 1.13 (1.06, 1.21) |
| Socialist Republic of Viet Nam | 65–69 years | 1.18 (1.11, 1.25) |
| Socialist Republic of Viet Nam | 70–74 years | 1.24 (1.17, 1.30) |
| Socialist Republic of Viet Nam | 75–79 years | 1.31 (1.24, 1.38) |
| Socialist Republic of Viet Nam | 80–84 years | 1.42 (1.33, 1.51) |
| Socialist Republic of Viet Nam | 85–89 years | 1.58 (1.44, 1.71) |
| Socialist Republic of Viet Nam | 90–94 years | 1.70 (1.46, 1.94) |
| Socialist Republic of Viet Nam | 95+ years | 1.60 (1.15, 2.05) |
| Solomon Islands | 30–34 years | 0.08 (-5.40, 5.87) |
| Solomon Islands | 35–39 years | 0.21 (-3.40, 3.95) |
| Solomon Islands | 40–44 years | 0.29 (-2.36, 3.02) |
| Solomon Islands | 45–49 years | 0.35 (-1.71, 2.44) |
| Solomon Islands | 50–54 years | 0.41 (-1.26, 2.11) |
| Solomon Islands | 55–59 years | 0.45 (-0.97, 1.88) |
| Solomon Islands | 60–64 years | 0.41 (-0.83, 1.68) |
| Solomon Islands | 65–69 years | 0.34 (-0.83, 1.51) |
| Solomon Islands | 70–74 years | 0.26 (-0.92, 1.46) |
| Solomon Islands | 75–79 years | 0.23 (-1.17, 1.65) |
| Solomon Islands | 80–84 years | 0.28 (-1.74, 2.35) |
| Solomon Islands | 85–89 years | 0.45 (-3.12, 4.16) |
| Solomon Islands | 90–94 years | 0.70 (-7.17, 9.24) |
| Solomon Islands | 95+ years | 0.88 (-19.00, 25.63) |
| State of Eritrea | 30–34 years | 0.10 (-1.57, 1.79) |
| State of Eritrea | 35–39 years | 0.19 (-0.93, 1.33) |
| State of Eritrea | 40–44 years | 0.21 (-0.67, 1.10) |
| State of Eritrea | 45–49 years | 0.18 (-0.58, 0.94) |
| State of Eritrea | 50–54 years | 0.13 (-0.54, 0.79) |
| State of Eritrea | 55–59 years | 0.13 (-0.48, 0.74) |
| State of Eritrea | 60–64 years | 0.21 (-0.36, 0.78) |
| State of Eritrea | 65–69 years | 0.35 (-0.21, 0.92) |
| State of Eritrea | 70–74 years | 0.53 (-0.08, 1.15) |
| State of Eritrea | 75–79 years | 0.72 (-0.03, 1.47) |
| State of Eritrea | 80–84 years | 0.86 (-0.29, 2.01) |
| State of Eritrea | 85–89 years | 0.97 (-1.19, 3.17) |
| State of Eritrea | 90–94 years | 1.04 (-3.79, 6.12) |
| State of Eritrea | 95+ years | 1.02 (-10.37, 13.86) |
| State of Israel | 30–34 years | 0.17 (-0.92, 1.28) |
| State of Israel | 35–39 years | 0.30 (-0.40, 1.00) |
| State of Israel | 40–44 years | 0.26 (-0.24, 0.76) |
| State of Israel | 45–49 years | 0.03 (-0.34, 0.41) |
| State of Israel | 50–54 years | -0.23 (-0.52, 0.06) |
| State of Israel | 55–59 years | -0.40 (-0.62, -0.17) |
| State of Israel | 60–64 years | -0.34 (-0.52, -0.17) |
| State of Israel | 65–69 years | -0.09 (-0.23, 0.05) |
| State of Israel | 70–74 years | 0.16 (0.04, 0.28) |
| State of Israel | 75–79 years | 0.30 (0.19, 0.42) |
| State of Israel | 80–84 years | 0.37 (0.25, 0.49) |
| State of Israel | 85–89 years | 0.36 (0.19, 0.52) |
| State of Israel | 90–94 years | 0.34 (0.01, 0.67) |
| State of Israel | 95+ years | 0.31 (-0.54, 1.18) |
| State of Kuwait | 30–34 years | 0.21 (-1.58, 2.03) |
| State of Kuwait | 35–39 years | 0.41 (-0.74, 1.56) |
| State of Kuwait | 40–44 years | 0.45 (-0.44, 1.35) |
| State of Kuwait | 45–49 years | 0.38 (-0.38, 1.14) |
| State of Kuwait | 50–54 years | 0.37 (-0.31, 1.06) |
| State of Kuwait | 55–59 years | 0.39 (-0.24, 1.01) |
| State of Kuwait | 60–64 years | 0.54 (-0.04, 1.12) |
| State of Kuwait | 65–69 years | 0.73 (0.18, 1.28) |
| State of Kuwait | 70–74 years | 0.96 (0.41, 1.51) |
| State of Kuwait | 75–79 years | 1.13 (0.55, 1.71) |
| State of Kuwait | 80–84 years | 1.29 (0.61, 1.99) |
| State of Kuwait | 85–89 years | 1.35 (0.36, 2.34) |
| State of Kuwait | 90–94 years | 1.38 (-0.20, 2.98) |
| State of Kuwait | 95+ years | 1.51 (-1.59, 4.69) |
| State of Libya | 30–34 years | 0.98 (-0.57, 2.54) |
| State of Libya | 35–39 years | 1.13 (0.20, 2.07) |
| State of Libya | 40–44 years | 1.26 (0.57, 1.95) |
| State of Libya | 45–49 years | 1.32 (0.77, 1.88) |
| State of Libya | 50–54 years | 1.33 (0.86, 1.81) |
| State of Libya | 55–59 years | 1.33 (0.92, 1.75) |
| State of Libya | 60–64 years | 1.37 (1.00, 1.74) |
| State of Libya | 65–69 years | 1.41 (1.07, 1.75) |
| State of Libya | 70–74 years | 1.41 (1.09, 1.74) |
| State of Libya | 75–79 years | 1.41 (1.07, 1.75) |
| State of Libya | 80–84 years | 1.38 (0.98, 1.79) |
| State of Libya | 85–89 years | 1.36 (0.82, 1.91) |
| State of Libya | 90–94 years | 1.41 (0.56, 2.26) |
| State of Libya | 95+ years | 1.53 (0.05, 3.04) |
| State of Qatar | 30–34 years | 0.34 (-1.92, 2.64) |
| State of Qatar | 35–39 years | 0.42 (-1.15, 2.01) |
| State of Qatar | 40–44 years | 0.46 (-0.81, 1.75) |
| State of Qatar | 45–49 years | 0.48 (-0.64, 1.62) |
| State of Qatar | 50–54 years | 0.56 (-0.50, 1.63) |
| State of Qatar | 55–59 years | 0.72 (-0.31, 1.77) |
| State of Qatar | 60–64 years | 0.97 (-0.08, 2.02) |
| State of Qatar | 65–69 years | 1.33 (0.24, 2.44) |
| State of Qatar | 70–74 years | 1.69 (0.53, 2.86) |
| State of Qatar | 75–79 years | 2.00 (0.67, 3.34) |
| State of Qatar | 80–84 years | 2.19 (0.39, 4.02) |
| State of Qatar | 85–89 years | 2.19 (-1.15, 5.65) |
| State of Qatar | 90–94 years | 1.83 (-5.08, 9.25) |
| State of Qatar | 95+ years | 1.59 (-18.55, 26.72) |
| Sultanate of Oman | 30–34 years | 0.54 (-1.26, 2.37) |
| Sultanate of Oman | 35–39 years | 0.67 (-0.53, 1.89) |
| Sultanate of Oman | 40–44 years | 0.82 (-0.14, 1.78) |
| Sultanate of Oman | 45–49 years | 0.93 (0.12, 1.75) |
| Sultanate of Oman | 50–54 years | 0.96 (0.24, 1.69) |
| Sultanate of Oman | 55–59 years | 0.95 (0.29, 1.61) |
| Sultanate of Oman | 60–64 years | 1.04 (0.44, 1.64) |
| Sultanate of Oman | 65–69 years | 1.18 (0.63, 1.74) |
| Sultanate of Oman | 70–74 years | 1.47 (0.95, 1.98) |
| Sultanate of Oman | 75–79 years | 1.91 (1.40, 2.42) |
| Sultanate of Oman | 80–84 years | 2.31 (1.69, 2.93) |
| Sultanate of Oman | 85–89 years | 2.44 (1.50, 3.39) |
| Sultanate of Oman | 90–94 years | 2.35 (0.61, 4.11) |
| Sultanate of Oman | 95+ years | 1.99 (-2.08, 6.24) |
| Swiss Confederation | 30–34 years | 0.44 (-0.66, 1.55) |
| Swiss Confederation | 35–39 years | 0.45 (-0.24, 1.15) |
| Swiss Confederation | 40–44 years | 0.47 (-0.03, 0.96) |
| Swiss Confederation | 45–49 years | 0.51 (0.15, 0.87) |
| Swiss Confederation | 50–54 years | 0.58 (0.32, 0.85) |
| Swiss Confederation | 55–59 years | 0.67 (0.47, 0.88) |
| Swiss Confederation | 60–64 years | 0.77 (0.60, 0.93) |
| Swiss Confederation | 65–69 years | 0.86 (0.73, 0.99) |
| Swiss Confederation | 70–74 years | 0.94 (0.83, 1.05) |
| Swiss Confederation | 75–79 years | 0.98 (0.88, 1.08) |
| Swiss Confederation | 80–84 years | 1.02 (0.92, 1.12) |
| Swiss Confederation | 85–89 years | 1.05 (0.92, 1.18) |
| Swiss Confederation | 90–94 years | 1.06 (0.84, 1.27) |
| Swiss Confederation | 95+ years | 1.01 (0.53, 1.48) |
| Syrian Arab Republic | 30–34 years | 0.78 (-0.55, 2.13) |
| Syrian Arab Republic | 35–39 years | 0.96 (0.28, 1.65) |
| Syrian Arab Republic | 40–44 years | 1.07 (0.59, 1.55) |
| Syrian Arab Republic | 45–49 years | 1.14 (0.77, 1.52) |
| Syrian Arab Republic | 50–54 years | 1.20 (0.90, 1.51) |
| Syrian Arab Republic | 55–59 years | 1.26 (1.00, 1.52) |
| Syrian Arab Republic | 60–64 years | 1.31 (1.08, 1.55) |
| Syrian Arab Republic | 65–69 years | 1.35 (1.15, 1.56) |
| Syrian Arab Republic | 70–74 years | 1.40 (1.20, 1.60) |
| Syrian Arab Republic | 75–79 years | 1.49 (1.28, 1.71) |
| Syrian Arab Republic | 80–84 years | 1.69 (1.42, 1.97) |
| Syrian Arab Republic | 85–89 years | 2.00 (1.57, 2.43) |
| Syrian Arab Republic | 90–94 years | 2.32 (1.51, 3.14) |
| Syrian Arab Republic | 95+ years | 2.60 (1.16, 4.06) |
| Taiwan (Province of China) | 30–34 years | 1.85 (-0.38, 4.14) |
| Taiwan (Province of China) | 35–39 years | 1.69 (0.38, 3.01) |
| Taiwan (Province of China) | 40–44 years | 1.76 (0.87, 2.67) |
| Taiwan (Province of China) | 45–49 years | 2.07 (1.43, 2.72) |
| Taiwan (Province of China) | 50–54 years | 2.55 (2.07, 3.03) |
| Taiwan (Province of China) | 55–59 years | 3.05 (2.68, 3.43) |
| Taiwan (Province of China) | 60–64 years | 3.54 (3.23, 3.84) |
| Taiwan (Province of China) | 65–69 years | 3.93 (3.66, 4.19) |
| Taiwan (Province of China) | 70–74 years | 4.22 (3.97, 4.47) |
| Taiwan (Province of China) | 75–79 years | 4.46 (4.20, 4.72) |
| Taiwan (Province of China) | 80–84 years | 4.75 (4.43, 5.08) |
| Taiwan (Province of China) | 85–89 years | 5.02 (4.50, 5.54) |
| Taiwan (Province of China) | 90–94 years | 5.03 (4.06, 6.01) |
| Taiwan (Province of China) | 95+ years | 4.63 (2.57, 6.74) |
| Togolese Republic | 30–34 years | 0.27 (-1.36, 1.93) |
| Togolese Republic | 35–39 years | 0.37 (-0.74, 1.49) |
| Togolese Republic | 40–44 years | 0.47 (-0.40, 1.36) |
| Togolese Republic | 45–49 years | 0.59 (-0.17, 1.34) |
| Togolese Republic | 50–54 years | 0.60 (-0.06, 1.27) |
| Togolese Republic | 55–59 years | 0.53 (-0.06, 1.13) |
| Togolese Republic | 60–64 years | 0.49 (-0.05, 1.02) |
| Togolese Republic | 65–69 years | 0.44 (-0.04, 0.93) |
| Togolese Republic | 70–74 years | 0.41 (-0.05, 0.87) |
| Togolese Republic | 75–79 years | 0.47 (-0.00, 0.95) |
| Togolese Republic | 80–84 years | 0.53 (-0.08, 1.14) |
| Togolese Republic | 85–89 years | 0.56 (-0.41, 1.55) |
| Togolese Republic | 90–94 years | 0.61 (-1.33, 2.60) |
| Togolese Republic | 95+ years | 0.59 (-4.05, 5.45) |
| Tokelau | 30–34 years | 0.23 (-72.56, 266.15) |
| Tokelau | 35–39 years | 0.21 (-52.86, 113.05) |
| Tokelau | 40–44 years | 0.23 (-42.19, 73.78) |
| Tokelau | 45–49 years | 0.21 (-31.19, 45.94) |
| Tokelau | 50–54 years | 0.21 (-24.61, 33.19) |
| Tokelau | 55–59 years | 0.26 (-19.31, 24.57) |
| Tokelau | 60–64 years | 0.31 (-15.64, 19.28) |
| Tokelau | 65–69 years | 0.32 (-12.28, 14.74) |
| Tokelau | 70–74 years | 0.36 (-11.65, 14.00) |
| Tokelau | 75–79 years | 0.49 (-11.54, 14.15) |
| Tokelau | 80–84 years | 0.65 (-13.16, 16.65) |
| Tokelau | 85–89 years | 0.97 (-18.19, 24.63) |
| Tokelau | 90–94 years | 1.35 (-32.40, 51.97) |
| Tokelau | 95+ years | 1.53 (-59.46, 154.28) |
| Turkmenistan | 30–34 years | 0.24 (-1.81, 2.32) |
| Turkmenistan | 35–39 years | 0.27 (-1.05, 1.61) |
| Turkmenistan | 40–44 years | 0.22 (-0.75, 1.20) |
| Turkmenistan | 45–49 years | 0.14 (-0.62, 0.91) |
| Turkmenistan | 50–54 years | 0.13 (-0.49, 0.74) |
| Turkmenistan | 55–59 years | 0.17 (-0.33, 0.67) |
| Turkmenistan | 60–64 years | 0.23 (-0.19, 0.65) |
| Turkmenistan | 65–69 years | 0.32 (-0.05, 0.69) |
| Turkmenistan | 70–74 years | 0.42 (0.05, 0.79) |
| Turkmenistan | 75–79 years | 0.43 (0.02, 0.85) |
| Turkmenistan | 80–84 years | 0.35 (-0.18, 0.88) |
| Turkmenistan | 85–89 years | 0.17 (-0.66, 1.01) |
| Turkmenistan | 90–94 years | -0.14 (-1.68, 1.42) |
| Turkmenistan | 95+ years | -0.71 (-4.44, 3.17) |
| Tuvalu | 30–34 years | 0.40 (-32.44, 49.19) |
| Tuvalu | 35–39 years | 0.39 (-22.12, 29.41) |
| Tuvalu | 40–44 years | 0.31 (-16.24, 20.13) |
| Tuvalu | 45–49 years | 0.22 (-12.05, 14.20) |
| Tuvalu | 50–54 years | 0.20 (-9.16, 10.54) |
| Tuvalu | 55–59 years | 0.23 (-7.25, 8.32) |
| Tuvalu | 60–64 years | 0.27 (-5.95, 6.91) |
| Tuvalu | 65–69 years | 0.29 (-5.09, 5.98) |
| Tuvalu | 70–74 years | 0.32 (-4.78, 5.70) |
| Tuvalu | 75–79 years | 0.46 (-5.38, 6.66) |
| Tuvalu | 80–84 years | 0.73 (-7.58, 9.79) |
| Tuvalu | 85–89 years | 1.06 (-13.05, 17.46) |
| Tuvalu | 90–94 years | 1.41 (-27.34, 41.54) |
| Tuvalu | 95+ years | 1.56 (-56.25, 135.79) |
| Ukraine | 30–34 years | 0.22 (-0.42, 0.86) |
| Ukraine | 35–39 years | 0.21 (-0.15, 0.58) |
| Ukraine | 40–44 years | 0.21 (-0.03, 0.46) |
| Ukraine | 45–49 years | 0.20 (0.03, 0.37) |
| Ukraine | 50–54 years | 0.18 (0.06, 0.30) |
| Ukraine | 55–59 years | 0.12 (0.04, 0.21) |
| Ukraine | 60–64 years | 0.06 (-0.01, 0.12) |
| Ukraine | 65–69 years | -0.01 (-0.07, 0.04) |
| Ukraine | 70–74 years | -0.09 (-0.14, -0.04) |
| Ukraine | 75–79 years | -0.23 (-0.29, -0.18) |
| Ukraine | 80–84 years | -0.39 (-0.46, -0.33) |
| Ukraine | 85–89 years | -0.55 (-0.65, -0.45) |
| Ukraine | 90–94 years | -0.60 (-0.81, -0.39) |
| Ukraine | 95+ years | -0.55 (-1.15, 0.04) |
| Union of the Comoros | 30–34 years | 0.10 (-4.74, 5.18) |
| Union of the Comoros | 35–39 years | 0.12 (-3.09, 3.44) |
| Union of the Comoros | 40–44 years | 0.12 (-2.34, 2.65) |
| Union of the Comoros | 45–49 years | 0.11 (-1.91, 2.18) |
| Union of the Comoros | 50–54 years | 0.11 (-1.63, 1.88) |
| Union of the Comoros | 55–59 years | 0.13 (-1.39, 1.67) |
| Union of the Comoros | 60–64 years | 0.18 (-1.17, 1.55) |
| Union of the Comoros | 65–69 years | 0.29 (-0.92, 1.52) |
| Union of the Comoros | 70–74 years | 0.44 (-0.73, 1.61) |
| Union of the Comoros | 75–79 years | 0.60 (-0.69, 1.91) |
| Union of the Comoros | 80–84 years | 0.76 (-0.97, 2.52) |
| Union of the Comoros | 85–89 years | 0.92 (-2.01, 3.94) |
| Union of the Comoros | 90–94 years | 1.01 (-4.85, 7.24) |
| Union of the Comoros | 95+ years | 1.02 (-13.44, 17.90) |
| United Arab Emirates | 30–34 years | 0.76 (-0.29, 1.81) |
| United Arab Emirates | 35–39 years | 0.87 (0.18, 1.56) |
| United Arab Emirates | 40–44 years | 0.99 (0.42, 1.55) |
| United Arab Emirates | 45–49 years | 1.05 (0.54, 1.57) |
| United Arab Emirates | 50–54 years | 1.12 (0.61, 1.64) |
| United Arab Emirates | 55–59 years | 1.22 (0.68, 1.76) |
| United Arab Emirates | 60–64 years | 1.32 (0.74, 1.90) |
| United Arab Emirates | 65–69 years | 1.39 (0.77, 2.01) |
| United Arab Emirates | 70–74 years | 1.33 (0.65, 2.01) |
| United Arab Emirates | 75–79 years | 1.13 (0.35, 1.92) |
| United Arab Emirates | 80–84 years | 1.00 (0.10, 1.90) |
| United Arab Emirates | 85–89 years | 1.05 (-0.43, 2.56) |
| United Arab Emirates | 90–94 years | 1.28 (-2.48, 5.19) |
| United Arab Emirates | 95+ years | 1.58 (-6.94, 10.90) |
| United Kingdom of Great Britain and Northern Ireland | 30–34 years | -0.36 (-1.36, 0.65) |
| United Kingdom of Great Britain and Northern Ireland | 35–39 years | 0.03 (-0.60, 0.67) |
| United Kingdom of Great Britain and Northern Ireland | 40–44 years | 0.07 (-0.38, 0.53) |
| United Kingdom of Great Britain and Northern Ireland | 45–49 years | -0.20 (-0.52, 0.12) |
| United Kingdom of Great Britain and Northern Ireland | 50–54 years | -0.49 (-0.72, -0.25) |
| United Kingdom of Great Britain and Northern Ireland | 55–59 years | -0.61 (-0.80, -0.43) |
| United Kingdom of Great Britain and Northern Ireland | 60–64 years | -0.50 (-0.64, -0.35) |
| United Kingdom of Great Britain and Northern Ireland | 65–69 years | -0.20 (-0.31, -0.08) |
| United Kingdom of Great Britain and Northern Ireland | 70–74 years | 0.14 (0.05, 0.24) |
| United Kingdom of Great Britain and Northern Ireland | 75–79 years | 0.46 (0.37, 0.55) |
| United Kingdom of Great Britain and Northern Ireland | 80–84 years | 0.73 (0.63, 0.83) |
| United Kingdom of Great Britain and Northern Ireland | 85–89 years | 0.96 (0.82, 1.09) |
| United Kingdom of Great Britain and Northern Ireland | 90–94 years | 1.15 (0.90, 1.39) |
| United Kingdom of Great Britain and Northern Ireland | 95+ years | 1.23 (0.65, 1.82) |
| United Mexican States | 30–34 years | 0.46 (0.17, 0.75) |
| United Mexican States | 35–39 years | 0.64 (0.46, 0.81) |
| United Mexican States | 40–44 years | 0.76 (0.64, 0.88) |
| United Mexican States | 45–49 years | 0.84 (0.74, 0.94) |
| United Mexican States | 50–54 years | 0.85 (0.77, 0.93) |
| United Mexican States | 55–59 years | 0.81 (0.73, 0.88) |
| United Mexican States | 60–64 years | 0.75 (0.69, 0.82) |
| United Mexican States | 65–69 years | 0.72 (0.65, 0.78) |
| United Mexican States | 70–74 years | 0.72 (0.66, 0.78) |
| United Mexican States | 75–79 years | 0.81 (0.74, 0.87) |
| United Mexican States | 80–84 years | 0.95 (0.87, 1.03) |
| United Mexican States | 85–89 years | 1.09 (0.98, 1.21) |
| United Mexican States | 90–94 years | 1.17 (0.96, 1.39) |
| United Mexican States | 95+ years | 1.11 (0.56, 1.66) |
| United Republic of Tanzania | 30–34 years | 0.15 (-0.46, 0.76) |
| United Republic of Tanzania | 35–39 years | 0.25 (-0.16, 0.66) |
| United Republic of Tanzania | 40–44 years | 0.34 (0.01, 0.66) |
| United Republic of Tanzania | 45–49 years | 0.42 (0.14, 0.70) |
| United Republic of Tanzania | 50–54 years | 0.50 (0.25, 0.75) |
| United Republic of Tanzania | 55–59 years | 0.57 (0.35, 0.79) |
| United Republic of Tanzania | 60–64 years | 0.62 (0.42, 0.81) |
| United Republic of Tanzania | 65–69 years | 0.65 (0.47, 0.83) |
| United Republic of Tanzania | 70–74 years | 0.67 (0.50, 0.84) |
| United Republic of Tanzania | 75–79 years | 0.70 (0.52, 0.88) |
| United Republic of Tanzania | 80–84 years | 0.76 (0.53, 0.99) |
| United Republic of Tanzania | 85–89 years | 0.85 (0.46, 1.24) |
| United Republic of Tanzania | 90–94 years | 0.92 (0.13, 1.72) |
| United Republic of Tanzania | 95+ years | 0.94 (-1.07, 2.99) |
| United States of America | 30–34 years | -0.57 (-1.72, 0.60) |
| United States of America | 35–39 years | -0.51 (-1.19, 0.17) |
| United States of America | 40–44 years | -0.34 (-0.79, 0.11) |
| United States of America | 45–49 years | -0.11 (-0.41, 0.19) |
| United States of America | 50–54 years | 0.10 (-0.11, 0.30) |
| United States of America | 55–59 years | 0.27 (0.12, 0.42) |
| United States of America | 60–64 years | 0.41 (0.30, 0.53) |
| United States of America | 65–69 years | 0.55 (0.46, 0.64) |
| United States of America | 70–74 years | 0.69 (0.61, 0.76) |
| United States of America | 75–79 years | 0.81 (0.74, 0.89) |
| United States of America | 80–84 years | 0.94 (0.85, 1.02) |
| United States of America | 85–89 years | 0.99 (0.87, 1.10) |
| United States of America | 90–94 years | 0.88 (0.68, 1.08) |
| United States of America | 95+ years | 0.49 (0.05, 0.94) |
| United States Virgin Islands | 30–34 years | 0.01 (-11.20, 12.64) |
| United States Virgin Islands | 35–39 years | 0.17 (-6.01, 6.76) |
| United States Virgin Islands | 40–44 years | 0.33 (-3.66, 4.48) |
| United States Virgin Islands | 45–49 years | 0.45 (-2.25, 3.22) |
| United States Virgin Islands | 50–54 years | 0.51 (-1.49, 2.55) |
| United States Virgin Islands | 55–59 years | 0.53 (-1.14, 2.23) |
| United States Virgin Islands | 60–64 years | 0.56 (-0.93, 2.08) |
| United States Virgin Islands | 65–69 years | 0.60 (-0.78, 2.00) |
| United States Virgin Islands | 70–74 years | 0.65 (-0.69, 2.01) |
| United States Virgin Islands | 75–79 years | 0.66 (-0.78, 2.12) |
| United States Virgin Islands | 80–84 years | 0.58 (-1.20, 2.39) |
| United States Virgin Islands | 85–89 years | 0.37 (-2.25, 3.06) |
| United States Virgin Islands | 90–94 years | 0.19 (-5.06, 5.73) |
| United States Virgin Islands | 95+ years | 0.06 (-15.55, 18.57) |

**Supplementary Table S4 Age effects on Parkinson’s disease prevalence across regions**

| **Location** | **Age** | **Prevalence rate (per 100,000 population)** |
| --- | --- | --- |
| Global | 30–34 years | 2.88 (2.73, 3.04) |
| Global | 35–39 years | 6.92 (6.67, 7.17) |
| Global | 40–44 years | 15.38 (14.97, 15.79) |
| Global | 45–49 years | 37.26 (36.53, 38.01) |
| Global | 50–54 years | 78.39 (77.19, 79.61) |
| Global | 55–59 years | 146.36 (144.51, 148.24) |
| Global | 60–64 years | 262.66 (259.85, 265.50) |
| Global | 65–69 years | 492.67 (488.11, 497.27) |
| Global | 70–74 years | 876.70 (869.24, 884.23) |
| Global | 75–79 years | 1449.48 (1437.38, 1461.69) |
| Global | 80–84 years | 2083.08 (2061.37, 2105.02) |
| Global | 85–89 years | 2456.26 (2425.74, 2487.16) |
| Global | 90–94 years | 2517.21 (2472.48, 2562.75) |
| Global | 95+ years | 2450.78 (2373.54, 2530.54) |
| High SDI | 30–34 years | 3.25 (3.09, 3.42) |
| High SDI | 35–39 years | 6.93 (6.70, 7.17) |
| High SDI | 40–44 years | 14.03 (13.69, 14.38) |
| High SDI | 45–49 years | 31.36 (30.80, 31.92) |
| High SDI | 50–54 years | 64.00 (63.15, 64.86) |
| High SDI | 55–59 years | 125.38 (124.08, 126.68) |
| High SDI | 60–64 years | 239.92 (237.94, 241.92) |
| High SDI | 65–69 years | 484.50 (481.23, 487.80) |
| High SDI | 70–74 years | 889.01 (883.66, 894.40) |
| High SDI | 75–79 years | 1471.69 (1463.18, 1480.26) |
| High SDI | 80–84 years | 2080.73 (2066.22, 2095.34) |
| High SDI | 85–89 years | 2345.20 (2326.71, 2363.85) |
| High SDI | 90–94 years | 2250.36 (2226.90, 2274.06) |
| High SDI | 95+ years | 2030.18 (1995.12, 2065.87) |
| High - middle SDI | 30–34 years | 2.33 (2.08, 2.61) |
| High - middle SDI | 35–39 years | 5.66 (5.25, 6.09) |
| High - middle SDI | 40–44 years | 14.07 (13.37, 14.81) |
| High - middle SDI | 45–49 years | 40.98 (39.56, 42.46) |
| High - middle SDI | 50–54 years | 93.85 (91.35, 96.41) |
| High - middle SDI | 55–59 years | 180.97 (177.05, 184.98) |
| High - middle SDI | 60–64 years | 328.18 (322.16, 334.31) |
| High - middle SDI | 65–69 years | 610.72 (601.00, 620.61) |
| High - middle SDI | 70–74 years | 1073.76 (1057.94, 1089.82) |
| High - middle SDI | 75–79 years | 1738.79 (1713.65, 1764.30) |
| High - middle SDI | 80–84 years | 2479.44 (2435.21, 2524.48) |
| High - middle SDI | 85–89 years | 2996.80 (2933.38, 3061.58) |
| High - middle SDI | 90–94 years | 3222.52 (3124.73, 3323.38) |
| High - middle SDI | 95+ years | 3232.12 (3047.99, 3427.36) |
| Middle SDI | 30–34 years | 2.95 (2.77, 3.14) |
| Middle SDI | 35–39 years | 7.34 (7.03, 7.65) |
| Middle SDI | 40–44 years | 16.99 (16.48, 17.52) |
| Middle SDI | 45–49 years | 43.31 (42.33, 44.31) |
| Middle SDI | 50–54 years | 93.30 (91.63, 95.00) |
| Middle SDI | 55–59 years | 172.48 (169.90, 175.10) |
| Middle SDI | 60–64 years | 301.74 (297.84, 305.70) |
| Middle SDI | 65–69 years | 539.68 (533.48, 545.96) |
| Middle SDI | 70–74 years | 932.70 (922.68, 942.84) |
| Middle SDI | 75–79 years | 1547.50 (1531.00, 1564.17) |
| Middle SDI | 80–84 years | 2271.59 (2240.03, 2303.60) |
| Middle SDI | 85–89 years | 2826.78 (2777.49, 2876.94) |
| Middle SDI | 90–94 years | 3184.70 (3099.95, 3271.76) |
| Middle SDI | 95+ years | 3620.47 (3446.93, 3802.74) |
| Low - middle SDI | 30–34 years | 3.59 (3.53, 3.65) |
| Low - middle SDI | 35–39 years | 8.78 (8.67, 8.88) |
| Low - middle SDI | 40–44 years | 18.10 (17.93, 18.27) |
| Low - middle SDI | 45–49 years | 35.18 (34.91, 35.45) |
| Low - middle SDI | 50–54 years | 62.88 (62.48, 63.28) |
| Low - middle SDI | 55–59 years | 107.94 (107.36, 108.52) |
| Low - middle SDI | 60–64 years | 186.69 (185.82, 187.55) |
| Low - middle SDI | 65–69 years | 349.76 (348.34, 351.18) |
| Low - middle SDI | 70–74 years | 613.13 (610.81, 615.45) |
| Low - middle SDI | 75–79 years | 981.66 (977.94, 985.40) |
| Low - middle SDI | 80–84 years | 1323.28 (1316.72, 1329.87) |
| Low - middle SDI | 85–89 years | 1379.14 (1370.12, 1388.23) |
| Low - middle SDI | 90–94 years | 1290.50 (1276.71, 1304.44) |
| Low - middle SDI | 95+ years | 1482.71 (1455.54, 1510.37) |
| Low SDI | 30–34 years | 3.72 (3.63, 3.81) |
| Low SDI | 35–39 years | 8.51 (8.36, 8.67) |
| Low SDI | 40–44 years | 16.47 (16.23, 16.72) |
| Low SDI | 45–49 years | 29.65 (29.29, 30.02) |
| Low SDI | 50–54 years | 49.88 (49.36, 50.40) |
| Low SDI | 55–59 years | 83.73 (82.99, 84.49) |
| Low SDI | 60–64 years | 145.20 (144.08, 146.32) |
| Low SDI | 65–69 years | 276.13 (274.27, 278.01) |
| Low SDI | 70–74 years | 488.54 (485.45, 491.65) |
| Low SDI | 75–79 years | 791.59 (786.53, 796.69) |
| Low SDI | 80–84 years | 1066.51 (1057.59, 1075.51) |
| Low SDI | 85–89 years | 1103.56 (1090.99, 1116.27) |
| Low SDI | 90–94 years | 1014.50 (994.19, 1035.22) |
| Low SDI | 95+ years | 1013.56 (971.53, 1057.42) |
| Central Europe, Eastern Europe, and Central Asia | 30–34 years | 2.93 (2.81, 3.04) |
| Central Europe, Eastern Europe, and Central Asia | 35–39 years | 6.57 (6.40, 6.75) |
| Central Europe, Eastern Europe, and Central Asia | 40–44 years | 13.34 (13.09, 13.59) |
| Central Europe, Eastern Europe, and Central Asia | 45–49 years | 28.10 (27.72, 28.49) |
| Central Europe, Eastern Europe, and Central Asia | 50–54 years | 55.80 (55.23, 56.37) |
| Central Europe, Eastern Europe, and Central Asia | 55–59 years | 111.93 (111.06, 112.80) |
| Central Europe, Eastern Europe, and Central Asia | 60–64 years | 216.99 (215.63, 218.35) |
| Central Europe, Eastern Europe, and Central Asia | 65–69 years | 434.88 (432.58, 437.20) |
| Central Europe, Eastern Europe, and Central Asia | 70–74 years | 750.94 (747.25, 754.66) |
| Central Europe, Eastern Europe, and Central Asia | 75–79 years | 1078.89 (1073.57, 1084.22) |
| Central Europe, Eastern Europe, and Central Asia | 80–84 years | 1288.91 (1281.22, 1296.65) |
| Central Europe, Eastern Europe, and Central Asia | 85–89 years | 1212.46 (1203.35, 1221.64) |
| Central Europe, Eastern Europe, and Central Asia | 90–94 years | 941.69 (929.82, 953.71) |
| Central Europe, Eastern Europe, and Central Asia | 95+ years | 660.75 (641.06, 681.04) |
| High - income | 30–34 years | 3.96 (3.71, 4.23) |
| High - income | 35–39 years | 8.07 (7.71, 8.44) |
| High - income | 40–44 years | 15.35 (14.85, 15.87) |
| High - income | 45–49 years | 31.62 (30.86, 32.39) |
| High - income | 50–54 years | 61.87 (60.76, 63.00) |
| High - income | 55–59 years | 120.05 (118.38, 121.73) |
| High - income | 60–64 years | 231.32 (228.80, 233.88) |
| High - income | 65–69 years | 477.89 (473.69, 482.12) |
| High - income | 70–74 years | 883.59 (876.72, 890.51) |
| High - income | 75–79 years | 1457.09 (1446.26, 1468.01) |
| High - income | 80–84 years | 2033.42 (2015.41, 2051.59) |
| High - income | 85–89 years | 2245.41 (2223.13, 2267.92) |
| High - income | 90–94 years | 2111.69 (2084.33, 2139.40) |
| High - income | 95+ years | 1848.90 (1809.11, 1889.57) |
| Latin America and Caribbean | 30–34 years | 4.52 (4.34, 4.70) |
| Latin America and Caribbean | 35–39 years | 11.80 (11.48, 12.13) |
| Latin America and Caribbean | 40–44 years | 25.38 (24.85, 25.92) |
| Latin America and Caribbean | 45–49 years | 50.60 (49.74, 51.48) |
| Latin America and Caribbean | 50–54 years | 89.30 (88.04, 90.58) |
| Latin America and Caribbean | 55–59 years | 140.05 (138.31, 141.81) |
| Latin America and Caribbean | 60–64 years | 214.02 (211.64, 216.42) |
| Latin America and Caribbean | 65–69 years | 356.95 (353.39, 360.55) |
| Latin America and Caribbean | 70–74 years | 590.57 (585.06, 596.13) |
| Latin America and Caribbean | 75–79 years | 940.10 (931.40, 948.88) |
| Latin America and Caribbean | 80–84 years | 1292.52 (1277.52, 1307.70) |
| Latin America and Caribbean | 85–89 years | 1441.16 (1421.19, 1461.42) |
| Latin America and Caribbean | 90–94 years | 1509.11 (1480.58, 1538.18) |
| Latin America and Caribbean | 95+ years | 1880.51 (1830.97, 1931.40) |
| North Africa and Middle East | 30–34 years | 2.50 (2.42, 2.58) |
| North Africa and Middle East | 35–39 years | 6.54 (6.40, 6.69) |
| North Africa and Middle East | 40–44 years | 14.43 (14.19, 14.67) |
| North Africa and Middle East | 45–49 years | 30.23 (29.83, 30.64) |
| North Africa and Middle East | 50–54 years | 56.66 (56.04, 57.28) |
| North Africa and Middle East | 55–59 years | 100.62 (99.69, 101.56) |
| North Africa and Middle East | 60–64 years | 178.21 (176.78, 179.65) |
| North Africa and Middle East | 65–69 years | 340.28 (337.89, 342.69) |
| North Africa and Middle East | 70–74 years | 622.56 (618.53, 626.63) |
| North Africa and Middle East | 75–79 years | 1114.13 (1107.11, 1121.20) |
| North Africa and Middle East | 80–84 years | 1688.26 (1674.84, 1701.78) |
| North Africa and Middle East | 85–89 years | 1999.24 (1979.75, 2018.92) |
| North Africa and Middle East | 90–94 years | 2066.20 (2035.55, 2097.31) |
| North Africa and Middle East | 95+ years | 2149.80 (2097.09, 2203.83) |
| South Asia | 30–34 years | 3.76 (3.67, 3.85) |
| South Asia | 35–39 years | 9.27 (9.11, 9.42) |
| South Asia | 40–44 years | 19.05 (18.80, 19.31) |
| South Asia | 45–49 years | 36.10 (35.70, 36.50) |
| South Asia | 50–54 years | 63.16 (62.57, 63.74) |
| South Asia | 55–59 years | 107.34 (106.50, 108.19) |
| South Asia | 60–64 years | 185.21 (183.97, 186.46) |
| South Asia | 65–69 years | 349.75 (347.70, 351.80) |
| South Asia | 70–74 years | 616.55 (613.19, 619.92) |
| South Asia | 75–79 years | 982.24 (976.86, 987.64) |
| South Asia | 80–84 years | 1310.76 (1301.24, 1320.35) |
| South Asia | 85–89 years | 1331.95 (1318.86, 1345.17) |
| South Asia | 90–94 years | 1183.25 (1163.08, 1203.77) |
| South Asia | 95+ years | 1141.14 (1100.65, 1183.12) |
| Southeast Asia, East Asia, and Oceania | 30–34 years | 2.36 (2.15, 2.59) |
| Southeast Asia, East Asia, and Oceania | 35–39 years | 5.83 (5.48, 6.20) |
| Southeast Asia, East Asia, and Oceania | 40–44 years | 15.27 (14.65, 15.93) |
| Southeast Asia, East Asia, and Oceania | 45–49 years | 47.72 (46.37, 49.12) |
| Southeast Asia, East Asia, and Oceania | 50–54 years | 113.26 (110.78, 115.79) |
| Southeast Asia, East Asia, and Oceania | 55–59 years | 220.13 (216.15, 224.19) |
| Southeast Asia, East Asia, and Oceania | 60–64 years | 394.31 (388.17, 400.54) |
| Southeast Asia, East Asia, and Oceania | 65–69 years | 702.36 (692.61, 712.24) |
| Southeast Asia, East Asia, and Oceania | 70–74 years | 1214.12 (1198.32, 1230.13) |
| Southeast Asia, East Asia, and Oceania | 75–79 years | 2038.53 (2012.19, 2065.22) |
| Southeast Asia, East Asia, and Oceania | 80–84 years | 3150.13 (3097.09, 3204.08) |
| Southeast Asia, East Asia, and Oceania | 85–89 years | 4395.24 (4303.36, 4489.07) |
| Southeast Asia, East Asia, and Oceania | 90–94 years | 5692.86 (5514.09, 5877.43) |
| Southeast Asia, East Asia, and Oceania | 95+ years | 7117.33 (6695.49, 7565.75) |
| Sub - Saharan Africa | 30–34 years | 3.85 (3.75, 3.95) |
| Sub - Saharan Africa | 35–39 years | 8.30 (8.14, 8.46) |
| Sub - Saharan Africa | 40–44 years | 15.27 (15.03, 15.52) |
| Sub - Saharan Africa | 45–49 years | 26.33 (25.98, 26.70) |
| Sub - Saharan Africa | 50–54 years | 43.15 (42.66, 43.65) |
| Sub - Saharan Africa | 55–59 years | 71.56 (70.85, 72.28) |
| Sub - Saharan Africa | 60–64 years | 123.54 (122.47, 124.62) |
| Sub - Saharan Africa | 65–69 years | 238.41 (236.61, 240.23) |
| Sub - Saharan Africa | 70–74 years | 435.30 (432.25, 438.38) |
| Sub - Saharan Africa | 75–79 years | 757.02 (751.77, 762.31) |
| Sub - Saharan Africa | 80–84 years | 1085.73 (1076.19, 1095.35) |
| Sub - Saharan Africa | 85–89 years | 1194.63 (1181.19, 1208.22) |
| Sub - Saharan Africa | 90–94 years | 1148.91 (1127.72, 1170.49) |
| Sub - Saharan Africa | 95+ years | 1116.47 (1074.86, 1159.69) |

**Supplementary Table S5 Period effects on Parkinson’s disease prevalence across regions**

| **Location** | **Period** | **Prevalence rate ratio** |
| --- | --- | --- |
| Global | 1992–1996 | 0.87 (0.86, 0.88) |
| Global | 1997–2001 | 0.93 (0.92, 0.94) |
| Global | 2002–2006 | 1.00 (1.00, 1.00) |
| Global | 2007–2011 | 1.08 (1.07, 1.09) |
| Global | 2012–2016 | 1.16 (1.15, 1.17) |
| Global | 2017–2021 | 1.23 (1.22, 1.24) |
| High SDI | 1992–1996 | 0.90 (0.89, 0.91) |
| High SDI | 1997–2001 | 0.94 (0.94, 0.95) |
| High SDI | 2002–2006 | 1.00 (1.00, 1.00) |
| High SDI | 2007–2011 | 1.04 (1.04, 1.05) |
| High SDI | 2012–2016 | 1.08 (1.08, 1.09) |
| High SDI | 2017–2021 | 1.11 (1.10, 1.12) |
| High - middle SDI | 1992–1996 | 0.84 (0.82, 0.86) |
| High - middle SDI | 1997–2001 | 0.91 (0.89, 0.92) |
| High - middle SDI | 2002–2006 | 1.00 (1.00, 1.00) |
| High - middle SDI | 2007–2011 | 1.08 (1.06, 1.09) |
| High - middle SDI | 2012–2016 | 1.17 (1.15, 1.18) |
| High - middle SDI | 2017–2021 | 1.25 (1.23, 1.27) |
| Middle SDI | 1992–1996 | 0.84 (0.83, 0.85) |
| Middle SDI | 1997–2001 | 0.92 (0.91, 0.93) |
| Middle SDI | 2002–2006 | 1.00 (1.00, 1.00) |
| Middle SDI | 2007–2011 | 1.12 (1.11, 1.14) |
| Middle SDI | 2012–2016 | 1.24 (1.23, 1.26) |
| Middle SDI | 2017–2021 | 1.36 (1.34, 1.37) |
| Low - middle SDI | 1992–1996 | 0.93 (0.93, 0.94) |
| Low - middle SDI | 1997–2001 | 0.95 (0.95, 0.96) |
| Low - middle SDI | 2002–2006 | 1.00 (1.00, 1.00) |
| Low - middle SDI | 2007–2011 | 1.07 (1.06, 1.07) |
| Low - middle SDI | 2012–2016 | 1.14 (1.13, 1.14) |
| Low - middle SDI | 2017–2021 | 1.21 (1.21, 1.22) |
| Low SDI | 1992–1996 | 0.98 (0.97, 0.98) |
| Low SDI | 1997–2001 | 0.98 (0.97, 0.98) |
| Low SDI | 2002–2006 | 1.00 (1.00, 1.00) |
| Low SDI | 2007–2011 | 1.04 (1.04, 1.05) |
| Low SDI | 2012–2016 | 1.10 (1.10, 1.11) |
| Low SDI | 2017–2021 | 1.17 (1.16, 1.18) |
| Central Europe, Eastern Europe, and Central Asia | 1992–1996 | 1.03 (1.02, 1.03) |
| Central Europe, Eastern Europe, and Central Asia | 1997–2001 | 0.99 (0.99, 1.00) |
| Central Europe, Eastern Europe, and Central Asia | 2002–2006 | 1.00 (1.00, 1.00) |
| Central Europe, Eastern Europe, and Central Asia | 2007–2011 | 0.99 (0.99, 1.00) |
| Central Europe, Eastern Europe, and Central Asia | 2012–2016 | 1.02 (1.01, 1.02) |
| Central Europe, Eastern Europe, and Central Asia | 2017–2021 | 1.03 (1.02, 1.03) |
| High - income | 1992–1996 | 0.92 (0.91, 0.93) |
| High - income | 1997–2001 | 0.96 (0.95, 0.96) |
| High - income | 2002–2006 | 1.00 (1.00, 1.00) |
| High - income | 2007–2011 | 1.00 (0.99, 1.01) |
| High - income | 2012–2016 | 1.01 (1.01, 1.02) |
| High - income | 2017–2021 | 1.03 (1.02, 1.04) |
| Latin America and Caribbean | 1992–1996 | 0.89 (0.88, 0.90) |
| Latin America and Caribbean | 1997–2001 | 0.94 (0.93, 0.95) |
| Latin America and Caribbean | 2002–2006 | 1.00 (1.00, 1.00) |
| Latin America and Caribbean | 2007–2011 | 1.05 (1.04, 1.06) |
| Latin America and Caribbean | 2012–2016 | 1.09 (1.08, 1.10) |
| Latin America and Caribbean | 2017–2021 | 1.12 (1.11, 1.13) |
| North Africa and Middle East | 1992–1996 | 0.92 (0.91, 0.92) |
| North Africa and Middle East | 1997–2001 | 0.95 (0.94, 0.95) |
| North Africa and Middle East | 2002–2006 | 1.00 (1.00, 1.00) |
| North Africa and Middle East | 2007–2011 | 1.07 (1.07, 1.08) |
| North Africa and Middle East | 2012–2016 | 1.16 (1.15, 1.17) |
| North Africa and Middle East | 2017–2021 | 1.25 (1.24, 1.26) |
| South Asia | 1992–1996 | 0.94 (0.93, 0.94) |
| South Asia | 1997–2001 | 0.95 (0.94, 0.96) |
| South Asia | 2002–2006 | 1.00 (1.00, 1.00) |
| South Asia | 2007–2011 | 1.08 (1.07, 1.08) |
| South Asia | 2012–2016 | 1.17 (1.16, 1.17) |
| South Asia | 2017–2021 | 1.25 (1.24, 1.26) |
| Southeast Asia, East Asia, and Oceania | 1992–1996 | 0.78 (0.77, 0.80) |
| Southeast Asia, East Asia, and Oceania | 1997–2001 | 0.89 (0.88, 0.90) |
| Southeast Asia, East Asia, and Oceania | 2002–2006 | 1.00 (1.00, 1.00) |
| Southeast Asia, East Asia, and Oceania | 2007–2011 | 1.16 (1.14, 1.18) |
| Southeast Asia, East Asia, and Oceania | 2012–2016 | 1.31 (1.29, 1.33) |
| Southeast Asia, East Asia, and Oceania | 2017–2021 | 1.44 (1.42, 1.47) |
| Sub - Saharan Africa | 1992–1996 | 0.99 (0.99, 1.00) |
| Sub - Saharan Africa | 1997–2001 | 0.98 (0.98, 0.99) |
| Sub - Saharan Africa | 2002–2006 | 1.00 (1.00, 1.00) |
| Sub - Saharan Africa | 2007–2011 | 1.02 (1.01, 1.03) |
| Sub - Saharan Africa | 2012–2016 | 1.07 (1.06, 1.07) |
| Sub - Saharan Africa | 2017–2021 | 1.11 (1.10, 1.12) |

**Supplementary Table S6 Cohort effects on Parkinson’s disease prevalence across regions**

| **Location** | **Cohort** | **Prevalence rate ratio** |
| --- | --- | --- |
| Global | 1897–1901 | 0.52 (0.46, 0.59) |
| Global | 1902–1906 | 0.55 (0.52, 0.58) |
| Global | 1907–1911 | 0.60 (0.58, 0.61) |
| Global | 1912–1916 | 0.65 (0.64, 0.66) |
| Global | 1917–1921 | 0.70 (0.69, 0.71) |
| Global | 1922–1926 | 0.76 (0.75, 0.77) |
| Global | 1927–1931 | 0.81 (0.80, 0.82) |
| Global | 1932–1936 | 0.87 (0.86, 0.88) |
| Global | 1937–1941 | 0.93 (0.92, 0.94) |
| Global | 1942–1946 | 1.00 (1.00, 1.00) |
| Global | 1947–1951 | 1.10 (1.08, 1.11) |
| Global | 1952–1956 | 1.21 (1.20, 1.23) |
| Global | 1957–1961 | 1.28 (1.26, 1.30) |
| Global | 1962–1966 | 1.37 (1.35, 1.40) |
| Global | 1967–1971 | 1.49 (1.46, 1.52) |
| Global | 1972–1976 | 1.51 (1.47, 1.56) |
| Global | 1977–1981 | 1.53 (1.47, 1.60) |
| Global | 1982–1986 | 1.58 (1.48, 1.68) |
| Global | 1987–1991 | 1.60 (1.44, 1.78) |
| High SDI | 1897–1901 | 0.65 (0.61, 0.69) |
| High SDI | 1902–1906 | 0.67 (0.65, 0.68) |
| High SDI | 1907–1911 | 0.69 (0.68, 0.70) |
| High SDI | 1912–1916 | 0.73 (0.72, 0.73) |
| High SDI | 1917–1921 | 0.76 (0.76, 0.77) |
| High SDI | 1922–1926 | 0.82 (0.81, 0.83) |
| High SDI | 1927–1931 | 0.87 (0.86, 0.88) |
| High SDI | 1932–1936 | 0.92 (0.92, 0.93) |
| High SDI | 1937–1941 | 0.97 (0.97, 0.98) |
| High SDI | 1942–1946 | 1.00 (1.00, 1.00) |
| High SDI | 1947–1951 | 1.04 (1.03, 1.05) |
| High SDI | 1952–1956 | 1.10 (1.09, 1.11) |
| High SDI | 1957–1961 | 1.15 (1.14, 1.17) |
| High SDI | 1962–1966 | 1.19 (1.18, 1.21) |
| High SDI | 1967–1971 | 1.23 (1.21, 1.26) |
| High SDI | 1972–1976 | 1.24 (1.20, 1.28) |
| High SDI | 1977–1981 | 1.25 (1.19, 1.30) |
| High SDI | 1982–1986 | 1.24 (1.16, 1.32) |
| High SDI | 1987–1991 | 1.23 (1.10, 1.38) |
| High - middle SDI | 1897–1901 | 0.49 (0.39, 0.63) |
| High - middle SDI | 1902–1906 | 0.54 (0.49, 0.59) |
| High - middle SDI | 1907–1911 | 0.60 (0.58, 0.63) |
| High - middle SDI | 1912–1916 | 0.67 (0.65, 0.69) |
| High - middle SDI | 1917–1921 | 0.72 (0.70, 0.74) |
| High - middle SDI | 1922–1926 | 0.77 (0.75, 0.78) |
| High - middle SDI | 1927–1931 | 0.79 (0.78, 0.81) |
| High - middle SDI | 1932–1936 | 0.85 (0.84, 0.87) |
| High - middle SDI | 1937–1941 | 0.91 (0.89, 0.92) |
| High - middle SDI | 1942–1946 | 1.00 (1.00, 1.00) |
| High - middle SDI | 1947–1951 | 1.12 (1.10, 1.14) |
| High - middle SDI | 1952–1956 | 1.26 (1.24, 1.29) |
| High - middle SDI | 1957–1961 | 1.37 (1.34, 1.41) |
| High - middle SDI | 1962–1966 | 1.52 (1.47, 1.56) |
| High - middle SDI | 1967–1971 | 1.69 (1.63, 1.76) |
| High - middle SDI | 1972–1976 | 1.73 (1.63, 1.82) |
| High - middle SDI | 1977–1981 | 1.73 (1.58, 1.89) |
| High - middle SDI | 1982–1986 | 1.77 (1.54, 2.03) |
| High - middle SDI | 1987–1991 | 1.81 (1.43, 2.30) |
| Middle SDI | 1897–1901 | 0.32 (0.25, 0.41) |
| Middle SDI | 1902–1906 | 0.35 (0.32, 0.39) |
| Middle SDI | 1907–1911 | 0.41 (0.39, 0.43) |
| Middle SDI | 1912–1916 | 0.48 (0.46, 0.49) |
| Middle SDI | 1917–1921 | 0.55 (0.54, 0.56) |
| Middle SDI | 1922–1926 | 0.63 (0.62, 0.64) |
| Middle SDI | 1927–1931 | 0.71 (0.70, 0.72) |
| Middle SDI | 1932–1936 | 0.80 (0.79, 0.81) |
| Middle SDI | 1937–1941 | 0.90 (0.89, 0.91) |
| Middle SDI | 1942–1946 | 1.00 (1.00, 1.00) |
| Middle SDI | 1947–1951 | 1.12 (1.11, 1.14) |
| Middle SDI | 1952–1956 | 1.24 (1.23, 1.26) |
| Middle SDI | 1957–1961 | 1.28 (1.26, 1.30) |
| Middle SDI | 1962–1966 | 1.38 (1.35, 1.41) |
| Middle SDI | 1967–1971 | 1.51 (1.47, 1.54) |
| Middle SDI | 1972–1976 | 1.54 (1.49, 1.59) |
| Middle SDI | 1977–1981 | 1.57 (1.50, 1.65) |
| Middle SDI | 1982–1986 | 1.63 (1.51, 1.75) |
| Middle SDI | 1987–1991 | 1.66 (1.46, 1.88) |
| Low - middle SDI | 1897–1901 | 0.53 (0.49, 0.57) |
| Low - middle SDI | 1902–1906 | 0.57 (0.55, 0.59) |
| Low - middle SDI | 1907–1911 | 0.61 (0.60, 0.62) |
| Low - middle SDI | 1912–1916 | 0.67 (0.66, 0.67) |
| Low - middle SDI | 1917–1921 | 0.73 (0.72, 0.73) |
| Low - middle SDI | 1922–1926 | 0.78 (0.78, 0.78) |
| Low - middle SDI | 1927–1931 | 0.83 (0.83, 0.84) |
| Low - middle SDI | 1932–1936 | 0.89 (0.88, 0.89) |
| Low - middle SDI | 1937–1941 | 0.95 (0.94, 0.95) |
| Low - middle SDI | 1942–1946 | 1.00 (1.00, 1.00) |
| Low - middle SDI | 1947–1951 | 1.05 (1.05, 1.06) |
| Low - middle SDI | 1952–1956 | 1.11 (1.10, 1.11) |
| Low - middle SDI | 1957–1961 | 1.17 (1.16, 1.17) |
| Low - middle SDI | 1962–1966 | 1.22 (1.21, 1.23) |
| Low - middle SDI | 1967–1971 | 1.26 (1.25, 1.28) |
| Low - middle SDI | 1972–1976 | 1.30 (1.28, 1.31) |
| Low - middle SDI | 1977–1981 | 1.32 (1.30, 1.34) |
| Low - middle SDI | 1982–1986 | 1.34 (1.32, 1.37) |
| Low - middle SDI | 1987–1991 | 1.36 (1.31, 1.40) |
| Low SDI | 1897–1901 | 0.62 (0.53, 0.72) |
| Low SDI | 1902–1906 | 0.66 (0.62, 0.70) |
| Low SDI | 1907–1911 | 0.69 (0.67, 0.71) |
| Low SDI | 1912–1916 | 0.74 (0.73, 0.75) |
| Low SDI | 1917–1921 | 0.79 (0.78, 0.79) |
| Low SDI | 1922–1926 | 0.83 (0.82, 0.84) |
| Low SDI | 1927–1931 | 0.88 (0.87, 0.88) |
| Low SDI | 1932–1936 | 0.92 (0.91, 0.93) |
| Low SDI | 1937–1941 | 0.96 (0.95, 0.97) |
| Low SDI | 1942–1946 | 1.00 (1.00, 1.00) |
| Low SDI | 1947–1951 | 1.05 (1.04, 1.05) |
| Low SDI | 1952–1956 | 1.08 (1.07, 1.09) |
| Low SDI | 1957–1961 | 1.10 (1.09, 1.11) |
| Low SDI | 1962–1966 | 1.13 (1.11, 1.14) |
| Low SDI | 1967–1971 | 1.15 (1.13, 1.17) |
| Low SDI | 1972–1976 | 1.17 (1.15, 1.19) |
| Low SDI | 1977–1981 | 1.17 (1.15, 1.20) |
| Low SDI | 1982–1986 | 1.18 (1.15, 1.21) |
| Low SDI | 1987–1991 | 1.19 (1.14, 1.24) |
| Central Europe, Eastern Europe, and Central Asia | 1897–1901 | 1.01 (0.92, 1.11) |
| Central Europe, Eastern Europe, and Central Asia | 1902–1906 | 0.96 (0.93, 0.99) |
| Central Europe, Eastern Europe, and Central Asia | 1907–1911 | 0.96 (0.95, 0.98) |
| Central Europe, Eastern Europe, and Central Asia | 1912–1916 | 0.96 (0.96, 0.97) |
| Central Europe, Eastern Europe, and Central Asia | 1917–1921 | 0.96 (0.96, 0.97) |
| Central Europe, Eastern Europe, and Central Asia | 1922–1926 | 0.98 (0.97, 0.98) |
| Central Europe, Eastern Europe, and Central Asia | 1927–1931 | 0.98 (0.97, 0.98) |
| Central Europe, Eastern Europe, and Central Asia | 1932–1936 | 0.99 (0.98, 0.99) |
| Central Europe, Eastern Europe, and Central Asia | 1937–1941 | 0.99 (0.98, 0.99) |
| Central Europe, Eastern Europe, and Central Asia | 1942–1946 | 1.00 (1.00, 1.00) |
| Central Europe, Eastern Europe, and Central Asia | 1947–1951 | 1.01 (1.01, 1.02) |
| Central Europe, Eastern Europe, and Central Asia | 1952–1956 | 1.01 (1.01, 1.02) |
| Central Europe, Eastern Europe, and Central Asia | 1957–1961 | 1.01 (1.00, 1.02) |
| Central Europe, Eastern Europe, and Central Asia | 1962–1966 | 1.01 (1.00, 1.02) |
| Central Europe, Eastern Europe, and Central Asia | 1967–1971 | 0.99 (0.98, 1.01) |
| Central Europe, Eastern Europe, and Central Asia | 1972–1976 | 0.99 (0.96, 1.01) |
| Central Europe, Eastern Europe, and Central Asia | 1977–1981 | 0.98 (0.94, 1.01) |
| Central Europe, Eastern Europe, and Central Asia | 1982–1986 | 0.98 (0.93, 1.03) |
| Central Europe, Eastern Europe, and Central Asia | 1987–1991 | 0.98 (0.90, 1.07) |
| High - income | 1897–1901 | 0.79 (0.74, 0.86) |
| High - income | 1902–1906 | 0.80 (0.78, 0.83) |
| High - income | 1907–1911 | 0.83 (0.81, 0.84) |
| High - income | 1912–1916 | 0.85 (0.84, 0.86) |
| High - income | 1917–1921 | 0.87 (0.86, 0.88) |
| High - income | 1922–1926 | 0.92 (0.91, 0.93) |
| High - income | 1927–1931 | 0.95 (0.94, 0.96) |
| High - income | 1932–1936 | 0.97 (0.96, 0.98) |
| High - income | 1937–1941 | 1.00 (0.99, 1.00) |
| High - income | 1942–1946 | 1.00 (1.00, 1.00) |
| High - income | 1947–1951 | 1.01 (1.00, 1.02) |
| High - income | 1952–1956 | 1.04 (1.02, 1.05) |
| High - income | 1957–1961 | 1.06 (1.04, 1.08) |
| High - income | 1962–1966 | 1.08 (1.06, 1.11) |
| High - income | 1967–1971 | 1.11 (1.08, 1.14) |
| High - income | 1972–1976 | 1.12 (1.08, 1.17) |
| High - income | 1977–1981 | 1.14 (1.07, 1.20) |
| High - income | 1982–1986 | 1.13 (1.03, 1.23) |
| High - income | 1987–1991 | 1.12 (0.97, 1.30) |
| Latin America and Caribbean | 1897–1901 | 0.65 (0.58, 0.73) |
| Latin America and Caribbean | 1902–1906 | 0.67 (0.64, 0.71) |
| Latin America and Caribbean | 1907–1911 | 0.70 (0.68, 0.72) |
| Latin America and Caribbean | 1912–1916 | 0.73 (0.72, 0.75) |
| Latin America and Caribbean | 1917–1921 | 0.78 (0.77, 0.79) |
| Latin America and Caribbean | 1922–1926 | 0.82 (0.81, 0.84) |
| Latin America and Caribbean | 1927–1931 | 0.87 (0.86, 0.88) |
| Latin America and Caribbean | 1932–1936 | 0.91 (0.90, 0.92) |
| Latin America and Caribbean | 1937–1941 | 0.95 (0.94, 0.96) |
| Latin America and Caribbean | 1942–1946 | 1.00 (1.00, 1.00) |
| Latin America and Caribbean | 1947–1951 | 1.05 (1.04, 1.06) |
| Latin America and Caribbean | 1952–1956 | 1.11 (1.09, 1.12) |
| Latin America and Caribbean | 1957–1961 | 1.16 (1.14, 1.18) |
| Latin America and Caribbean | 1962–1966 | 1.22 (1.20, 1.24) |
| Latin America and Caribbean | 1967–1971 | 1.27 (1.25, 1.29) |
| Latin America and Caribbean | 1972–1976 | 1.31 (1.28, 1.34) |
| Latin America and Caribbean | 1977–1981 | 1.34 (1.30, 1.38) |
| Latin America and Caribbean | 1982–1986 | 1.36 (1.30, 1.43) |
| Latin America and Caribbean | 1987–1991 | 1.38 (1.27, 1.49) |
| North Africa and Middle East | 1897–1901 | 0.47 (0.43, 0.52) |
| North Africa and Middle East | 1902–1906 | 0.51 (0.49, 0.53) |
| North Africa and Middle East | 1907–1911 | 0.56 (0.55, 0.58) |
| North Africa and Middle East | 1912–1916 | 0.64 (0.63, 0.65) |
| North Africa and Middle East | 1917–1921 | 0.70 (0.70, 0.71) |
| North Africa and Middle East | 1922–1926 | 0.76 (0.75, 0.77) |
| North Africa and Middle East | 1927–1931 | 0.82 (0.81, 0.83) |
| North Africa and Middle East | 1932–1936 | 0.88 (0.87, 0.88) |
| North Africa and Middle East | 1937–1941 | 0.93 (0.93, 0.94) |
| North Africa and Middle East | 1942–1946 | 1.00 (1.00, 1.00) |
| North Africa and Middle East | 1947–1951 | 1.07 (1.06, 1.08) |
| North Africa and Middle East | 1952–1956 | 1.14 (1.13, 1.15) |
| North Africa and Middle East | 1957–1961 | 1.20 (1.19, 1.22) |
| North Africa and Middle East | 1962–1966 | 1.27 (1.25, 1.28) |
| North Africa and Middle East | 1967–1971 | 1.33 (1.31, 1.35) |
| North Africa and Middle East | 1972–1976 | 1.38 (1.35, 1.40) |
| North Africa and Middle East | 1977–1981 | 1.41 (1.38, 1.45) |
| North Africa and Middle East | 1982–1986 | 1.44 (1.39, 1.49) |
| North Africa and Middle East | 1987–1991 | 1.45 (1.37, 1.54) |
| South Asia | 1897–1901 | 0.48 (0.41, 0.55) |
| South Asia | 1902–1906 | 0.51 (0.48, 0.54) |
| South Asia | 1907–1911 | 0.56 (0.54, 0.57) |
| South Asia | 1912–1916 | 0.63 (0.62, 0.64) |
| South Asia | 1917–1921 | 0.71 (0.70, 0.71) |
| South Asia | 1922–1926 | 0.77 (0.77, 0.78) |
| South Asia | 1927–1931 | 0.84 (0.83, 0.84) |
| South Asia | 1932–1936 | 0.89 (0.89, 0.90) |
| South Asia | 1937–1941 | 0.95 (0.94, 0.95) |
| South Asia | 1942–1946 | 1.00 (1.00, 1.00) |
| South Asia | 1947–1951 | 1.06 (1.05, 1.06) |
| South Asia | 1952–1956 | 1.12 (1.11, 1.13) |
| South Asia | 1957–1961 | 1.19 (1.18, 1.20) |
| South Asia | 1962–1966 | 1.24 (1.23, 1.26) |
| South Asia | 1967–1971 | 1.29 (1.28, 1.31) |
| South Asia | 1972–1976 | 1.34 (1.32, 1.36) |
| South Asia | 1977–1981 | 1.37 (1.34, 1.39) |
| South Asia | 1982–1986 | 1.39 (1.36, 1.43) |
| South Asia | 1987–1991 | 1.41 (1.35, 1.47) |
| Southeast Asia, East Asia, and Oceania | 1897–1901 | 0.22 (0.15, 0.31) |
| Southeast Asia, East Asia, and Oceania | 1902–1906 | 0.26 (0.23, 0.30) |
| Southeast Asia, East Asia, and Oceania | 1907–1911 | 0.32 (0.30, 0.34) |
| Southeast Asia, East Asia, and Oceania | 1912–1916 | 0.39 (0.38, 0.41) |
| Southeast Asia, East Asia, and Oceania | 1917–1921 | 0.47 (0.46, 0.48) |
| Southeast Asia, East Asia, and Oceania | 1922–1926 | 0.56 (0.54, 0.57) |
| Southeast Asia, East Asia, and Oceania | 1927–1931 | 0.65 (0.64, 0.66) |
| Southeast Asia, East Asia, and Oceania | 1932–1936 | 0.76 (0.74, 0.77) |
| Southeast Asia, East Asia, and Oceania | 1937–1941 | 0.87 (0.86, 0.89) |
| Southeast Asia, East Asia, and Oceania | 1942–1946 | 1.00 (1.00, 1.00) |
| Southeast Asia, East Asia, and Oceania | 1947–1951 | 1.15 (1.13, 1.17) |
| Southeast Asia, East Asia, and Oceania | 1952–1956 | 1.30 (1.28, 1.32) |
| Southeast Asia, East Asia, and Oceania | 1957–1961 | 1.40 (1.37, 1.43) |
| Southeast Asia, East Asia, and Oceania | 1962–1966 | 1.53 (1.49, 1.56) |
| Southeast Asia, East Asia, and Oceania | 1967–1971 | 1.67 (1.62, 1.72) |
| Southeast Asia, East Asia, and Oceania | 1972–1976 | 1.73 (1.66, 1.81) |
| Southeast Asia, East Asia, and Oceania | 1977–1981 | 1.76 (1.64, 1.89) |
| Southeast Asia, East Asia, and Oceania | 1982–1986 | 1.82 (1.62, 2.04) |
| Southeast Asia, East Asia, and Oceania | 1987–1991 | 1.88 (1.55, 2.28) |
| Sub - Saharan Africa | 1897–1901 | 0.71 (0.63, 0.80) |
| Sub - Saharan Africa | 1902–1906 | 0.75 (0.71, 0.78) |
| Sub - Saharan Africa | 1907–1911 | 0.78 (0.76, 0.80) |
| Sub - Saharan Africa | 1912–1916 | 0.81 (0.80, 0.82) |
| Sub - Saharan Africa | 1917–1921 | 0.84 (0.83, 0.85) |
| Sub - Saharan Africa | 1922–1926 | 0.88 (0.87, 0.89) |
| Sub - Saharan Africa | 1927–1931 | 0.91 (0.91, 0.92) |
| Sub - Saharan Africa | 1932–1936 | 0.95 (0.94, 0.95) |
| Sub - Saharan Africa | 1937–1941 | 0.97 (0.97, 0.98) |
| Sub - Saharan Africa | 1942–1946 | 1.00 (1.00, 1.00) |
| Sub - Saharan Africa | 1947–1951 | 1.02 (1.01, 1.03) |
| Sub - Saharan Africa | 1952–1956 | 1.04 (1.03, 1.05) |
| Sub - Saharan Africa | 1957–1961 | 1.05 (1.04, 1.06) |
| Sub - Saharan Africa | 1962–1966 | 1.06 (1.04, 1.07) |
| Sub - Saharan Africa | 1967–1971 | 1.07 (1.05, 1.08) |
| Sub - Saharan Africa | 1972–1976 | 1.08 (1.06, 1.10) |
| Sub - Saharan Africa | 1977–1981 | 1.08 (1.06, 1.11) |
| Sub - Saharan Africa | 1982–1986 | 1.09 (1.06, 1.12) |
| Sub - Saharan Africa | 1987–1991 | 1.09 (1.05, 1.14) |

**Supplementary Table S7 Age effects on Parkinson’s disease prevalence across 204 countries**

| **Location** | **Age** | **Prevalence rate (per 100,000 population)** |
| --- | --- | --- |
| American Samoa | 30–34 years | 2.95 (0.18, 48.97) |
| American Samoa | 35–39 years | 6.13 (0.79, 47.32) |
| American Samoa | 40–44 years | 11.83 (2.43, 57.64) |
| American Samoa | 45–49 years | 24.69 (7.14, 85.37) |
| American Samoa | 50–54 years | 48.42 (17.99, 130.32) |
| American Samoa | 55–59 years | 92.78 (40.70, 211.52) |
| American Samoa | 60–64 years | 168.40 (81.85, 346.48) |
| American Samoa | 65–69 years | 303.49 (158.00, 582.94) |
| American Samoa | 70–74 years | 505.20 (271.66, 939.50) |
| American Samoa | 75–79 years | 783.34 (419.52, 1462.69) |
| American Samoa | 80–84 years | 1081.48 (498.27, 2347.36) |
| American Samoa | 85–89 years | 1334.50 (513.81, 3466.01) |
| American Samoa | 90–94 years | 1582.63 (369.42, 6780.11) |
| American Samoa | 95+ years | 1911.23 (106.06, 34439.78) |
| Antigua and Barbuda | 30–34 years | 5.12 (0.97, 27.00) |
| Antigua and Barbuda | 35–39 years | 12.34 (3.92, 38.85) |
| Antigua and Barbuda | 40–44 years | 25.14 (10.31, 61.29) |
| Antigua and Barbuda | 45–49 years | 48.23 (23.24, 100.13) |
| Antigua and Barbuda | 50–54 years | 83.50 (45.11, 154.58) |
| Antigua and Barbuda | 55–59 years | 131.35 (76.06, 226.84) |
| Antigua and Barbuda | 60–64 years | 199.13 (122.39, 323.99) |
| Antigua and Barbuda | 65–69 years | 312.83 (200.70, 487.61) |
| Antigua and Barbuda | 70–74 years | 498.94 (326.89, 761.55) |
| Antigua and Barbuda | 75–79 years | 822.89 (545.91, 1240.39) |
| Antigua and Barbuda | 80–84 years | 1146.69 (691.14, 1902.50) |
| Antigua and Barbuda | 85–89 years | 1272.46 (695.05, 2329.55) |
| Antigua and Barbuda | 90–94 years | 1307.11 (551.54, 3097.77) |
| Antigua and Barbuda | 95+ years | 1514.98 (345.57, 6641.60) |
| Arab Republic of Egypt | 30–34 years | 2.31 (2.14, 2.49) |
| Arab Republic of Egypt | 35–39 years | 6.13 (5.82, 6.46) |
| Arab Republic of Egypt | 40–44 years | 14.37 (13.84, 14.93) |
| Arab Republic of Egypt | 45–49 years | 33.80 (32.84, 34.79) |
| Arab Republic of Egypt | 50–54 years | 68.31 (66.75, 69.90) |
| Arab Republic of Egypt | 55–59 years | 126.37 (123.94, 128.86) |
| Arab Republic of Egypt | 60–64 years | 231.59 (227.70, 235.54) |
| Arab Republic of Egypt | 65–69 years | 459.11 (452.22, 466.11) |
| Arab Republic of Egypt | 70–74 years | 846.66 (834.23, 859.28) |
| Arab Republic of Egypt | 75–79 years | 1467.72 (1445.57, 1490.21) |
| Arab Republic of Egypt | 80–84 years | 2195.95 (2152.63, 2240.15) |
| Arab Republic of Egypt | 85–89 years | 2612.65 (2544.11, 2683.04) |
| Arab Republic of Egypt | 90–94 years | 2742.47 (2623.78, 2866.53) |
| Arab Republic of Egypt | 95+ years | 3234.09 (3020.10, 3463.25) |
| Argentine Republic | 30–34 years | 2.38 (2.08, 2.71) |
| Argentine Republic | 35–39 years | 5.18 (4.72, 5.69) |
| Argentine Republic | 40–44 years | 10.35 (9.66, 11.09) |
| Argentine Republic | 45–49 years | 21.65 (20.57, 22.78) |
| Argentine Republic | 50–54 years | 45.72 (44.05, 47.45) |
| Argentine Republic | 55–59 years | 105.06 (102.28, 107.93) |
| Argentine Republic | 60–64 years | 218.84 (214.36, 223.42) |
| Argentine Republic | 65–69 years | 437.32 (429.86, 444.91) |
| Argentine Republic | 70–74 years | 784.51 (772.47, 796.75) |
| Argentine Republic | 75–79 years | 1314.46 (1295.07, 1334.13) |
| Argentine Republic | 80–84 years | 1816.66 (1784.50, 1849.40) |
| Argentine Republic | 85–89 years | 1852.92 (1814.89, 1891.75) |
| Argentine Republic | 90–94 years | 1611.03 (1564.60, 1658.84) |
| Argentine Republic | 95+ years | 1606.35 (1532.28, 1684.01) |
| Australia | 30–34 years | 1.99 (1.67, 2.38) |
| Australia | 35–39 years | 4.19 (3.71, 4.75) |
| Australia | 40–44 years | 7.90 (7.20, 8.65) |
| Australia | 45–49 years | 15.24 (14.23, 16.33) |
| Australia | 50–54 years | 31.23 (29.68, 32.87) |
| Australia | 55–59 years | 73.37 (70.73, 76.11) |
| Australia | 60–64 years | 164.52 (160.08, 169.08) |
| Australia | 65–69 years | 375.63 (367.70, 383.73) |
| Australia | 70–74 years | 715.73 (702.57, 729.13) |
| Australia | 75–79 years | 1139.11 (1119.04, 1159.53) |
| Australia | 80–84 years | 1506.77 (1474.14, 1540.12) |
| Australia | 85–89 years | 1582.06 (1542.83, 1622.28) |
| Australia | 90–94 years | 1430.49 (1382.18, 1480.49) |
| Australia | 95+ years | 1218.53 (1143.29, 1298.73) |
| Barbados | 30–34 years | 5.00 (1.99, 12.55) |
| Barbados | 35–39 years | 12.27 (6.70, 22.47) |
| Barbados | 40–44 years | 25.05 (15.87, 39.55) |
| Barbados | 45–49 years | 47.29 (32.91, 67.96) |
| Barbados | 50–54 years | 80.39 (59.62, 108.40) |
| Barbados | 55–59 years | 124.94 (96.41, 161.93) |
| Barbados | 60–64 years | 187.84 (149.57, 235.89) |
| Barbados | 65–69 years | 296.47 (242.12, 363.03) |
| Barbados | 70–74 years | 467.82 (387.89, 564.23) |
| Barbados | 75–79 years | 733.31 (610.16, 881.31) |
| Barbados | 80–84 years | 1006.69 (807.38, 1255.20) |
| Barbados | 85–89 years | 1169.86 (908.79, 1505.94) |
| Barbados | 90–94 years | 1270.17 (902.98, 1786.67) |
| Barbados | 95+ years | 1474.73 (837.93, 2595.47) |
| Belize | 30–34 years | 4.68 (1.69, 12.94) |
| Belize | 35–39 years | 11.57 (5.55, 24.09) |
| Belize | 40–44 years | 24.01 (13.33, 43.27) |
| Belize | 45–49 years | 46.82 (28.58, 76.69) |
| Belize | 50–54 years | 81.37 (53.37, 124.04) |
| Belize | 55–59 years | 126.64 (87.05, 184.24) |
| Belize | 60–64 years | 188.26 (134.04, 264.42) |
| Belize | 65–69 years | 290.12 (212.26, 396.54) |
| Belize | 70–74 years | 446.36 (330.89, 602.12) |
| Belize | 75–79 years | 683.15 (507.50, 919.58) |
| Belize | 80–84 years | 904.81 (625.24, 1309.39) |
| Belize | 85–89 years | 983.47 (630.23, 1534.68) |
| Belize | 90–94 years | 1017.12 (553.47, 1869.16) |
| Belize | 95+ years | 1194.23 (490.67, 2906.64) |
| Bermuda | 30–34 years | 5.42 (0.90, 32.62) |
| Bermuda | 35–39 years | 13.59 (4.33, 42.68) |
| Bermuda | 40–44 years | 28.30 (12.25, 65.41) |
| Bermuda | 45–49 years | 55.04 (28.70, 105.56) |
| Bermuda | 50–54 years | 94.80 (56.22, 159.88) |
| Bermuda | 55–59 years | 146.17 (93.43, 228.68) |
| Bermuda | 60–64 years | 215.96 (145.19, 321.22) |
| Bermuda | 65–69 years | 332.41 (231.64, 477.02) |
| Bermuda | 70–74 years | 528.48 (376.94, 740.94) |
| Bermuda | 75–79 years | 894.05 (649.71, 1230.29) |
| Bermuda | 80–84 years | 1300.57 (882.30, 1917.11) |
| Bermuda | 85–89 years | 1509.44 (967.99, 2353.76) |
| Bermuda | 90–94 years | 1575.54 (881.31, 2816.65) |
| Bermuda | 95+ years | 1706.29 (717.67, 4056.79) |
| Bolivarian Republic of Venezuela | 30–34 years | 4.49 (4.07, 4.95) |
| Bolivarian Republic of Venezuela | 35–39 years | 11.57 (10.81, 12.39) |
| Bolivarian Republic of Venezuela | 40–44 years | 24.65 (23.38, 26.00) |
| Bolivarian Republic of Venezuela | 45–49 years | 48.56 (46.48, 50.74) |
| Bolivarian Republic of Venezuela | 50–54 years | 84.99 (81.91, 88.18) |
| Bolivarian Republic of Venezuela | 55–59 years | 133.40 (129.12, 137.82) |
| Bolivarian Republic of Venezuela | 60–64 years | 204.44 (198.53, 210.52) |
| Bolivarian Republic of Venezuela | 65–69 years | 338.09 (329.27, 347.14) |
| Bolivarian Republic of Venezuela | 70–74 years | 555.55 (541.96, 569.48) |
| Bolivarian Republic of Venezuela | 75–79 years | 890.05 (868.31, 912.34) |
| Bolivarian Republic of Venezuela | 80–84 years | 1231.85 (1194.43, 1270.45) |
| Bolivarian Republic of Venezuela | 85–89 years | 1382.69 (1333.35, 1433.86) |
| Bolivarian Republic of Venezuela | 90–94 years | 1459.97 (1393.06, 1530.09) |
| Bolivarian Republic of Venezuela | 95+ years | 1874.63 (1774.90, 1979.95) |
| Bosnia and Herzegovina | 30–34 years | 2.76 (1.91, 4.00) |
| Bosnia and Herzegovina | 35–39 years | 5.99 (4.70, 7.62) |
| Bosnia and Herzegovina | 40–44 years | 12.13 (10.25, 14.34) |
| Bosnia and Herzegovina | 45–49 years | 26.49 (23.52, 29.85) |
| Bosnia and Herzegovina | 50–54 years | 55.43 (50.91, 60.35) |
| Bosnia and Herzegovina | 55–59 years | 119.49 (112.41, 127.01) |
| Bosnia and Herzegovina | 60–64 years | 243.25 (232.00, 255.05) |
| Bosnia and Herzegovina | 65–69 years | 504.05 (484.47, 524.42) |
| Bosnia and Herzegovina | 70–74 years | 873.34 (841.65, 906.21) |
| Bosnia and Herzegovina | 75–79 years | 1240.08 (1194.82, 1287.06) |
| Bosnia and Herzegovina | 80–84 years | 1467.05 (1401.12, 1536.09) |
| Bosnia and Herzegovina | 85–89 years | 1329.09 (1251.11, 1411.93) |
| Bosnia and Herzegovina | 90–94 years | 978.49 (881.12, 1086.63) |
| Bosnia and Herzegovina | 95+ years | 668.08 (548.08, 814.35) |
| Brunei Darussalam | 30–34 years | 2.88 (1.10, 7.53) |
| Brunei Darussalam | 35–39 years | 6.70 (3.36, 13.34) |
| Brunei Darussalam | 40–44 years | 13.62 (7.87, 23.55) |
| Brunei Darussalam | 45–49 years | 27.01 (17.11, 42.63) |
| Brunei Darussalam | 50–54 years | 52.31 (35.59, 76.87) |
| Brunei Darussalam | 55–59 years | 108.54 (78.37, 150.35) |
| Brunei Darussalam | 60–64 years | 216.89 (164.16, 286.56) |
| Brunei Darussalam | 65–69 years | 436.17 (341.80, 556.60) |
| Brunei Darussalam | 70–74 years | 751.05 (596.99, 944.88) |
| Brunei Darussalam | 75–79 years | 1104.99 (877.96, 1390.73) |
| Brunei Darussalam | 80–84 years | 1373.05 (1013.71, 1859.77) |
| Brunei Darussalam | 85–89 years | 1378.06 (913.07, 2079.84) |
| Brunei Darussalam | 90–94 years | 1023.32 (384.95, 2720.30) |
| Brunei Darussalam | 95+ years | 734.87 (8.95, 60307.02) |
| Burkina Faso | 30–34 years | 3.29 (2.70, 4.01) |
| Burkina Faso | 35–39 years | 7.21 (6.20, 8.38) |
| Burkina Faso | 40–44 years | 13.35 (11.80, 15.12) |
| Burkina Faso | 45–49 years | 22.87 (20.60, 25.39) |
| Burkina Faso | 50–54 years | 37.98 (34.81, 41.44) |
| Burkina Faso | 55–59 years | 66.59 (61.91, 71.63) |
| Burkina Faso | 60–64 years | 121.75 (114.53, 129.43) |
| Burkina Faso | 65–69 years | 243.05 (230.79, 255.96) |
| Burkina Faso | 70–74 years | 458.61 (437.56, 480.67) |
| Burkina Faso | 75–79 years | 838.40 (800.88, 877.68) |
| Burkina Faso | 80–84 years | 1212.55 (1144.32, 1284.86) |
| Burkina Faso | 85–89 years | 1274.18 (1182.63, 1372.82) |
| Burkina Faso | 90–94 years | 1124.28 (991.96, 1274.25) |
| Burkina Faso | 95+ years | 952.55 (726.53, 1248.86) |
| Canada | 30–34 years | 3.19 (2.90, 3.51) |
| Canada | 35–39 years | 8.14 (7.67, 8.64) |
| Canada | 40–44 years | 18.63 (17.90, 19.40) |
| Canada | 45–49 years | 43.69 (42.47, 44.94) |
| Canada | 50–54 years | 93.06 (91.12, 95.03) |
| Canada | 55–59 years | 194.80 (191.66, 198.00) |
| Canada | 60–64 years | 386.08 (381.09, 391.14) |
| Canada | 65–69 years | 767.68 (759.48, 775.98) |
| Canada | 70–74 years | 1401.93 (1388.55, 1415.44) |
| Canada | 75–79 years | 2371.04 (2349.28, 2393.01) |
| Canada | 80–84 years | 3448.05 (3409.56, 3486.98) |
| Canada | 85–89 years | 3918.90 (3869.40, 3969.03) |
| Canada | 90–94 years | 3721.92 (3659.61, 3785.28) |
| Canada | 95+ years | 3234.77 (3140.46, 3331.90) |
| Central African Republic | 30–34 years | 3.81 (2.71, 5.35) |
| Central African Republic | 35–39 years | 8.64 (6.69, 11.16) |
| Central African Republic | 40–44 years | 16.73 (13.57, 20.62) |
| Central African Republic | 45–49 years | 30.97 (25.98, 36.91) |
| Central African Republic | 50–54 years | 52.56 (45.35, 60.92) |
| Central African Republic | 55–59 years | 86.30 (75.81, 98.25) |
| Central African Republic | 60–64 years | 142.87 (127.08, 160.63) |
| Central African Republic | 65–69 years | 258.84 (232.22, 288.50) |
| Central African Republic | 70–74 years | 419.25 (376.19, 467.23) |
| Central African Republic | 75–79 years | 579.65 (513.53, 654.28) |
| Central African Republic | 80–84 years | 681.84 (575.65, 807.63) |
| Central African Republic | 85–89 years | 680.48 (519.43, 891.47) |
| Central African Republic | 90–94 years | 648.23 (370.02, 1135.62) |
| Central African Republic | 95+ years | 657.40 (185.50, 2329.78) |
| Commonwealth of Dominica | 30–34 years | 5.20 (0.79, 34.19) |
| Commonwealth of Dominica | 35–39 years | 12.64 (3.48, 45.91) |
| Commonwealth of Dominica | 40–44 years | 25.70 (9.57, 69.03) |
| Commonwealth of Dominica | 45–49 years | 48.48 (21.74, 108.14) |
| Commonwealth of Dominica | 50–54 years | 81.45 (41.89, 158.37) |
| Commonwealth of Dominica | 55–59 years | 121.34 (67.45, 218.30) |
| Commonwealth of Dominica | 60–64 years | 176.48 (104.01, 299.43) |
| Commonwealth of Dominica | 65–69 years | 279.06 (172.56, 451.29) |
| Commonwealth of Dominica | 70–74 years | 438.08 (276.86, 693.20) |
| Commonwealth of Dominica | 75–79 years | 684.17 (432.70, 1081.78) |
| Commonwealth of Dominica | 80–84 years | 941.15 (545.01, 1625.20) |
| Commonwealth of Dominica | 85–89 years | 1086.31 (568.93, 2074.20) |
| Commonwealth of Dominica | 90–94 years | 1128.68 (449.84, 2831.91) |
| Commonwealth of Dominica | 95+ years | 1142.56 (228.66, 5709.21) |
| Commonwealth of the Bahamas | 30–34 years | 5.44 (2.44, 12.13) |
| Commonwealth of the Bahamas | 35–39 years | 13.50 (7.77, 23.45) |
| Commonwealth of the Bahamas | 40–44 years | 27.90 (18.10, 43.00) |
| Commonwealth of the Bahamas | 45–49 years | 53.93 (37.67, 77.22) |
| Commonwealth of the Bahamas | 50–54 years | 91.81 (67.87, 124.19) |
| Commonwealth of the Bahamas | 55–59 years | 137.42 (104.99, 179.87) |
| Commonwealth of the Bahamas | 60–64 years | 197.29 (153.98, 252.78) |
| Commonwealth of the Bahamas | 65–69 years | 297.41 (235.37, 375.79) |
| Commonwealth of the Bahamas | 70–74 years | 451.74 (359.46, 567.72) |
| Commonwealth of the Bahamas | 75–79 years | 705.48 (560.59, 887.82) |
| Commonwealth of the Bahamas | 80–84 years | 964.85 (724.72, 1284.53) |
| Commonwealth of the Bahamas | 85–89 years | 1071.63 (757.10, 1516.82) |
| Commonwealth of the Bahamas | 90–94 years | 1075.98 (666.43, 1737.22) |
| Commonwealth of the Bahamas | 95+ years | 1178.81 (578.57, 2401.77) |
| Cook Islands | 30–34 years | 3.39 (0.04, 320.59) |
| Cook Islands | 35–39 years | 7.23 (0.29, 180.96) |
| Cook Islands | 40–44 years | 14.56 (1.40, 151.86) |
| Cook Islands | 45–49 years | 32.04 (5.78, 177.47) |
| Cook Islands | 50–54 years | 64.74 (18.53, 226.17) |
| Cook Islands | 55–59 years | 126.19 (47.36, 336.21) |
| Cook Islands | 60–64 years | 229.93 (102.60, 515.24) |
| Cook Islands | 65–69 years | 414.67 (204.72, 839.93) |
| Cook Islands | 70–74 years | 695.25 (363.15, 1331.07) |
| Cook Islands | 75–79 years | 1112.09 (592.45, 2087.48) |
| Cook Islands | 80–84 years | 1574.71 (710.41, 3490.54) |
| Cook Islands | 85–89 years | 1924.19 (744.97, 4970.05) |
| Cook Islands | 90–94 years | 2164.41 (541.24, 8655.46) |
| Cook Islands | 95+ years | 2405.43 (186.66, 30997.57) |
| Czech Republic | 30–34 years | 2.28 (1.81, 2.87) |
| Czech Republic | 35–39 years | 4.94 (4.23, 5.77) |
| Czech Republic | 40–44 years | 10.06 (9.03, 11.21) |
| Czech Republic | 45–49 years | 22.16 (20.57, 23.87) |
| Czech Republic | 50–54 years | 46.18 (43.80, 48.68) |
| Czech Republic | 55–59 years | 97.78 (94.06, 101.65) |
| Czech Republic | 60–64 years | 203.30 (197.38, 209.39) |
| Czech Republic | 65–69 years | 449.04 (438.63, 459.70) |
| Czech Republic | 70–74 years | 822.80 (805.77, 840.18) |
| Czech Republic | 75–79 years | 1202.42 (1177.84, 1227.51) |
| Czech Republic | 80–84 years | 1441.30 (1404.36, 1479.21) |
| Czech Republic | 85–89 years | 1341.09 (1298.73, 1384.82) |
| Czech Republic | 90–94 years | 1032.62 (980.19, 1087.85) |
| Czech Republic | 95+ years | 744.19 (669.22, 827.56) |
| Democratic People's Republic of Korea | 30–34 years | 2.54 (2.28, 2.83) |
| Democratic People's Republic of Korea | 35–39 years | 5.99 (5.57, 6.44) |
| Democratic People's Republic of Korea | 40–44 years | 13.93 (13.22, 14.67) |
| Democratic People's Republic of Korea | 45–49 years | 36.93 (35.56, 38.34) |
| Democratic People's Republic of Korea | 50–54 years | 83.61 (81.30, 85.97) |
| Democratic People's Republic of Korea | 55–59 years | 173.19 (169.39, 177.07) |
| Democratic People's Republic of Korea | 60–64 years | 330.95 (324.97, 337.04) |
| Democratic People's Republic of Korea | 65–69 years | 609.48 (599.77, 619.34) |
| Democratic People's Republic of Korea | 70–74 years | 1031.79 (1016.23, 1047.58) |
| Democratic People's Republic of Korea | 75–79 years | 1554.05 (1529.66, 1578.84) |
| Democratic People's Republic of Korea | 80–84 years | 2132.96 (2087.55, 2179.35) |
| Democratic People's Republic of Korea | 85–89 years | 2816.84 (2736.56, 2899.48) |
| Democratic People's Republic of Korea | 90–94 years | 3652.47 (3481.09, 3832.29) |
| Democratic People's Republic of Korea | 95+ years | 4779.03 (4303.98, 5306.53) |
| Democratic Republic of Sao Tome and Principe | 30–34 years | 3.58 (0.69, 18.58) |
| Democratic Republic of Sao Tome and Principe | 35–39 years | 8.27 (2.36, 29.03) |
| Democratic Republic of Sao Tome and Principe | 40–44 years | 15.70 (5.58, 44.16) |
| Democratic Republic of Sao Tome and Principe | 45–49 years | 26.34 (10.84, 64.01) |
| Democratic Republic of Sao Tome and Principe | 50–54 years | 42.66 (19.96, 91.16) |
| Democratic Republic of Sao Tome and Principe | 55–59 years | 73.88 (39.11, 139.58) |
| Democratic Republic of Sao Tome and Principe | 60–64 years | 137.35 (80.77, 233.56) |
| Democratic Republic of Sao Tome and Principe | 65–69 years | 286.76 (183.15, 448.99) |
| Democratic Republic of Sao Tome and Principe | 70–74 years | 553.32 (368.47, 830.90) |
| Democratic Republic of Sao Tome and Principe | 75–79 years | 1013.39 (683.95, 1501.51) |
| Democratic Republic of Sao Tome and Principe | 80–84 years | 1517.99 (942.37, 2445.20) |
| Democratic Republic of Sao Tome and Principe | 85–89 years | 1710.06 (954.99, 3062.14) |
| Democratic Republic of Sao Tome and Principe | 90–94 years | 1630.71 (659.05, 4034.95) |
| Democratic Republic of Sao Tome and Principe | 95+ years | 1454.67 (244.30, 8661.78) |
| Democratic Republic of the Congo | 30–34 years | 3.40 (3.10, 3.74) |
| Democratic Republic of the Congo | 35–39 years | 7.49 (6.97, 8.04) |
| Democratic Republic of the Congo | 40–44 years | 14.16 (13.36, 15.01) |
| Democratic Republic of the Congo | 45–49 years | 25.72 (24.49, 27.02) |
| Democratic Republic of the Congo | 50–54 years | 44.12 (42.33, 45.98) |
| Democratic Republic of the Congo | 55–59 years | 75.25 (72.59, 78.00) |
| Democratic Republic of the Congo | 60–64 years | 130.21 (126.13, 134.41) |
| Democratic Republic of the Congo | 65–69 years | 246.03 (239.10, 253.15) |
| Democratic Republic of the Congo | 70–74 years | 416.74 (405.48, 428.32) |
| Democratic Republic of the Congo | 75–79 years | 602.59 (585.50, 620.18) |
| Democratic Republic of the Congo | 80–84 years | 746.11 (718.44, 774.84) |
| Democratic Republic of the Congo | 85–89 years | 798.24 (757.70, 840.95) |
| Democratic Republic of the Congo | 90–94 years | 799.90 (729.17, 877.49) |
| Democratic Republic of the Congo | 95+ years | 820.38 (670.06, 1004.43) |
| Democratic Republic of Timor - Leste | 30–34 years | 3.29 (1.67, 6.49) |
| Democratic Republic of Timor - Leste | 35–39 years | 7.03 (4.25, 11.61) |
| Democratic Republic of Timor - Leste | 40–44 years | 13.32 (8.97, 19.78) |
| Democratic Republic of Timor - Leste | 45–49 years | 25.65 (18.60, 35.36) |
| Democratic Republic of Timor - Leste | 50–54 years | 47.42 (36.37, 61.84) |
| Democratic Republic of Timor - Leste | 55–59 years | 88.73 (71.38, 110.30) |
| Democratic Republic of Timor - Leste | 60–64 years | 162.33 (136.15, 193.55) |
| Democratic Republic of Timor - Leste | 65–69 years | 306.58 (263.71, 356.41) |
| Democratic Republic of Timor - Leste | 70–74 years | 530.07 (459.97, 610.85) |
| Democratic Republic of Timor - Leste | 75–79 years | 828.96 (714.22, 962.12) |
| Democratic Republic of Timor - Leste | 80–84 years | 1081.80 (880.83, 1328.62) |
| Democratic Republic of Timor - Leste | 85–89 years | 1075.10 (803.78, 1438.00) |
| Democratic Republic of Timor - Leste | 90–94 years | 1034.81 (611.72, 1750.53) |
| Democratic Republic of Timor - Leste | 95+ years | 1440.66 (556.12, 3732.09) |
| Democratic Socialist Republic of Sri Lanka | 30–34 years | 3.73 (3.30, 4.23) |
| Democratic Socialist Republic of Sri Lanka | 35–39 years | 7.90 (7.22, 8.65) |
| Democratic Socialist Republic of Sri Lanka | 40–44 years | 14.95 (13.95, 16.02) |
| Democratic Socialist Republic of Sri Lanka | 45–49 years | 29.01 (27.48, 30.62) |
| Democratic Socialist Republic of Sri Lanka | 50–54 years | 53.62 (51.37, 55.97) |
| Democratic Socialist Republic of Sri Lanka | 55–59 years | 98.67 (95.25, 102.22) |
| Democratic Socialist Republic of Sri Lanka | 60–64 years | 184.53 (179.14, 190.07) |
| Democratic Socialist Republic of Sri Lanka | 65–69 years | 376.61 (367.24, 386.23) |
| Democratic Socialist Republic of Sri Lanka | 70–74 years | 682.12 (666.51, 698.10) |
| Democratic Socialist Republic of Sri Lanka | 75–79 years | 1057.46 (1033.16, 1082.33) |
| Democratic Socialist Republic of Sri Lanka | 80–84 years | 1392.85 (1351.40, 1435.58) |
| Democratic Socialist Republic of Sri Lanka | 85–89 years | 1481.75 (1426.23, 1539.44) |
| Democratic Socialist Republic of Sri Lanka | 90–94 years | 1494.27 (1409.70, 1583.92) |
| Democratic Socialist Republic of Sri Lanka | 95+ years | 1959.14 (1767.47, 2171.61) |
| Dominican Republic | 30–34 years | 4.31 (3.63, 5.13) |
| Dominican Republic | 35–39 years | 10.74 (9.50, 12.15) |
| Dominican Republic | 40–44 years | 22.13 (20.07, 24.39) |
| Dominican Republic | 45–49 years | 41.90 (38.63, 45.43) |
| Dominican Republic | 50–54 years | 71.38 (66.67, 76.41) |
| Dominican Republic | 55–59 years | 110.66 (104.22, 117.50) |
| Dominican Republic | 60–64 years | 167.85 (159.07, 177.11) |
| Dominican Republic | 65–69 years | 274.57 (261.55, 288.24) |
| Dominican Republic | 70–74 years | 444.72 (424.66, 465.72) |
| Dominican Republic | 75–79 years | 689.28 (658.31, 721.70) |
| Dominican Republic | 80–84 years | 909.22 (858.63, 962.79) |
| Dominican Republic | 85–89 years | 947.42 (884.45, 1014.88) |
| Dominican Republic | 90–94 years | 959.94 (871.83, 1056.95) |
| Dominican Republic | 95+ years | 1211.74 (1040.71, 1410.87) |
| Eastern Republic of Uruguay | 30–34 years | 2.07 (1.40, 3.07) |
| Eastern Republic of Uruguay | 35–39 years | 4.62 (3.53, 6.05) |
| Eastern Republic of Uruguay | 40–44 years | 9.47 (7.78, 11.53) |
| Eastern Republic of Uruguay | 45–49 years | 20.45 (17.73, 23.59) |
| Eastern Republic of Uruguay | 50–54 years | 44.62 (40.26, 49.45) |
| Eastern Republic of Uruguay | 55–59 years | 106.09 (98.55, 114.21) |
| Eastern Republic of Uruguay | 60–64 years | 224.65 (212.33, 237.67) |
| Eastern Republic of Uruguay | 65–69 years | 443.71 (423.54, 464.84) |
| Eastern Republic of Uruguay | 70–74 years | 801.43 (768.69, 835.56) |
| Eastern Republic of Uruguay | 75–79 years | 1391.19 (1337.13, 1447.43) |
| Eastern Republic of Uruguay | 80–84 years | 2018.24 (1925.16, 2115.82) |
| Eastern Republic of Uruguay | 85–89 years | 2174.50 (2060.37, 2294.96) |
| Eastern Republic of Uruguay | 90–94 years | 2004.09 (1862.52, 2156.42) |
| Eastern Republic of Uruguay | 95+ years | 2073.73 (1852.08, 2321.91) |
| Federal Democratic Republic of Ethiopia | 30–34 years | 4.97 (4.59, 5.38) |
| Federal Democratic Republic of Ethiopia | 35–39 years | 10.45 (9.84, 11.09) |
| Federal Democratic Republic of Ethiopia | 40–44 years | 18.79 (17.88, 19.74) |
| Federal Democratic Republic of Ethiopia | 45–49 years | 31.73 (30.41, 33.12) |
| Federal Democratic Republic of Ethiopia | 50–54 years | 50.08 (48.30, 51.94) |
| Federal Democratic Republic of Ethiopia | 55–59 years | 78.37 (75.90, 80.91) |
| Federal Democratic Republic of Ethiopia | 60–64 years | 127.76 (124.20, 131.43) |
| Federal Democratic Republic of Ethiopia | 65–69 years | 236.66 (230.84, 242.64) |
| Federal Democratic Republic of Ethiopia | 70–74 years | 408.88 (399.48, 418.49) |
| Federal Democratic Republic of Ethiopia | 75–79 years | 636.02 (621.41, 650.97) |
| Federal Democratic Republic of Ethiopia | 80–84 years | 835.82 (810.53, 861.90) |
| Federal Democratic Republic of Ethiopia | 85–89 years | 902.86 (865.70, 941.62) |
| Federal Democratic Republic of Ethiopia | 90–94 years | 901.92 (836.35, 972.64) |
| Federal Democratic Republic of Ethiopia | 95+ years | 945.78 (797.40, 1121.78) |
| Federal Democratic Republic of Nepal | 30–34 years | 2.78 (2.44, 3.16) |
| Federal Democratic Republic of Nepal | 35–39 years | 6.81 (6.21, 7.47) |
| Federal Democratic Republic of Nepal | 40–44 years | 14.07 (13.09, 15.12) |
| Federal Democratic Republic of Nepal | 45–49 years | 27.13 (25.59, 28.77) |
| Federal Democratic Republic of Nepal | 50–54 years | 48.52 (46.25, 50.90) |
| Federal Democratic Republic of Nepal | 55–59 years | 84.82 (81.47, 88.31) |
| Federal Democratic Republic of Nepal | 60–64 years | 152.48 (147.34, 157.80) |
| Federal Democratic Republic of Nepal | 65–69 years | 307.01 (298.14, 316.14) |
| Federal Democratic Republic of Nepal | 70–74 years | 567.69 (552.60, 583.19) |
| Federal Democratic Republic of Nepal | 75–79 years | 919.89 (895.16, 945.30) |
| Federal Democratic Republic of Nepal | 80–84 years | 1241.37 (1196.87, 1287.53) |
| Federal Democratic Republic of Nepal | 85–89 years | 1285.18 (1223.50, 1349.97) |
| Federal Democratic Republic of Nepal | 90–94 years | 1153.39 (1059.18, 1255.99) |
| Federal Democratic Republic of Nepal | 95+ years | 1127.09 (945.34, 1343.79) |
| Federal Republic of Germany | 30–34 years | 5.43 (4.80, 6.15) |
| Federal Republic of Germany | 35–39 years | 10.55 (9.67, 11.52) |
| Federal Republic of Germany | 40–44 years | 19.54 (18.32, 20.84) |
| Federal Republic of Germany | 45–49 years | 41.16 (39.27, 43.13) |
| Federal Republic of Germany | 50–54 years | 81.04 (78.28, 83.90) |
| Federal Republic of Germany | 55–59 years | 151.09 (147.15, 155.13) |
| Federal Republic of Germany | 60–64 years | 287.90 (281.98, 293.94) |
| Federal Republic of Germany | 65–69 years | 618.35 (608.27, 628.60) |
| Federal Republic of Germany | 70–74 years | 1221.85 (1204.68, 1239.27) |
| Federal Republic of Germany | 75–79 years | 2234.86 (2205.59, 2264.51) |
| Federal Republic of Germany | 80–84 years | 3543.79 (3487.53, 3600.96) |
| Federal Republic of Germany | 85–89 years | 4660.52 (4577.45, 4745.09) |
| Federal Republic of Germany | 90–94 years | 5454.98 (5330.96, 5581.89) |
| Federal Republic of Germany | 95+ years | 6279.50 (6056.49, 6510.71) |
| Federal Republic of Nigeria | 30–34 years | 3.80 (3.59, 4.02) |
| Federal Republic of Nigeria | 35–39 years | 8.07 (7.72, 8.43) |
| Federal Republic of Nigeria | 40–44 years | 14.71 (14.19, 15.26) |
| Federal Republic of Nigeria | 45–49 years | 24.93 (24.17, 25.71) |
| Federal Republic of Nigeria | 50–54 years | 40.68 (39.63, 41.76) |
| Federal Republic of Nigeria | 55–59 years | 69.49 (67.96, 71.07) |
| Federal Republic of Nigeria | 60–64 years | 126.84 (124.41, 129.31) |
| Federal Republic of Nigeria | 65–69 years | 262.54 (258.23, 266.91) |
| Federal Republic of Nigeria | 70–74 years | 512.31 (504.73, 520.01) |
| Federal Republic of Nigeria | 75–79 years | 961.52 (947.82, 975.40) |
| Federal Republic of Nigeria | 80–84 years | 1445.46 (1419.72, 1471.67) |
| Federal Republic of Nigeria | 85–89 years | 1608.16 (1572.77, 1644.34) |
| Federal Republic of Nigeria | 90–94 years | 1541.32 (1489.06, 1595.41) |
| Federal Republic of Nigeria | 95+ years | 1467.25 (1374.74, 1565.99) |
| Federal Republic of Somalia | 30–34 years | 4.17 (3.38, 5.16) |
| Federal Republic of Somalia | 35–39 years | 9.00 (7.65, 10.60) |
| Federal Republic of Somalia | 40–44 years | 16.52 (14.45, 18.89) |
| Federal Republic of Somalia | 45–49 years | 28.27 (25.21, 31.71) |
| Federal Republic of Somalia | 50–54 years | 45.23 (41.02, 49.87) |
| Federal Republic of Somalia | 55–59 years | 71.48 (65.49, 78.01) |
| Federal Republic of Somalia | 60–64 years | 115.00 (106.12, 124.62) |
| Federal Republic of Somalia | 65–69 years | 202.19 (187.50, 218.04) |
| Federal Republic of Somalia | 70–74 years | 330.32 (306.04, 356.53) |
| Federal Republic of Somalia | 75–79 years | 494.41 (454.33, 538.03) |
| Federal Republic of Somalia | 80–84 years | 626.36 (555.28, 706.55) |
| Federal Republic of Somalia | 85–89 years | 646.40 (533.70, 782.90) |
| Federal Republic of Somalia | 90–94 years | 623.09 (421.78, 920.49) |
| Federal Republic of Somalia | 95+ years | 650.30 (275.89, 1532.80) |
| Federated States of Micronesia | 30–34 years | 3.02 (0.38, 23.84) |
| Federated States of Micronesia | 35–39 years | 6.32 (1.39, 28.64) |
| Federated States of Micronesia | 40–44 years | 12.94 (4.13, 40.54) |
| Federated States of Micronesia | 45–49 years | 30.27 (12.59, 72.76) |
| Federated States of Micronesia | 50–54 years | 62.16 (30.41, 127.07) |
| Federated States of Micronesia | 55–59 years | 117.20 (63.98, 214.69) |
| Federated States of Micronesia | 60–64 years | 210.99 (123.60, 360.16) |
| Federated States of Micronesia | 65–69 years | 386.86 (232.05, 644.93) |
| Federated States of Micronesia | 70–74 years | 644.75 (390.21, 1065.32) |
| Federated States of Micronesia | 75–79 years | 992.38 (591.98, 1663.60) |
| Federated States of Micronesia | 80–84 years | 1322.89 (712.97, 2454.56) |
| Federated States of Micronesia | 85–89 years | 1472.79 (674.31, 3216.77) |
| Federated States of Micronesia | 90–94 years | 1536.77 (421.23, 5606.52) |
| Federated States of Micronesia | 95+ years | 1656.04 (101.55, 27006.65) |
| Federative Republic of Brazil | 30–34 years | 4.24 (3.96, 4.53) |
| Federative Republic of Brazil | 35–39 years | 10.91 (10.42, 11.43) |
| Federative Republic of Brazil | 40–44 years | 23.27 (22.46, 24.12) |
| Federative Republic of Brazil | 45–49 years | 45.96 (44.65, 47.31) |
| Federative Republic of Brazil | 50–54 years | 81.11 (79.20, 83.08) |
| Federative Republic of Brazil | 55–59 years | 129.39 (126.72, 132.11) |
| Federative Republic of Brazil | 60–64 years | 203.03 (199.32, 206.80) |
| Federative Republic of Brazil | 65–69 years | 347.93 (342.29, 353.66) |
| Federative Republic of Brazil | 70–74 years | 578.72 (569.90, 587.67) |
| Federative Republic of Brazil | 75–79 years | 898.03 (884.37, 911.90) |
| Federative Republic of Brazil | 80–84 years | 1179.05 (1156.45, 1202.08) |
| Federative Republic of Brazil | 85–89 years | 1204.47 (1176.20, 1233.42) |
| Federative Republic of Brazil | 90–94 years | 1167.24 (1128.50, 1207.31) |
| Federative Republic of Brazil | 95+ years | 1486.04 (1416.59, 1558.89) |
| French Republic | 30–34 years | 5.10 (4.58, 5.67) |
| French Republic | 35–39 years | 9.87 (9.15, 10.64) |
| French Republic | 40–44 years | 17.79 (16.81, 18.81) |
| French Republic | 45–49 years | 35.27 (33.82, 36.77) |
| French Republic | 50–54 years | 66.76 (64.65, 68.94) |
| French Republic | 55–59 years | 122.49 (119.42, 125.64) |
| French Republic | 60–64 years | 237.18 (232.48, 241.97) |
| French Republic | 65–69 years | 533.62 (525.31, 542.07) |
| French Republic | 70–74 years | 1065.75 (1051.35, 1080.36) |
| French Republic | 75–79 years | 1867.96 (1844.01, 1892.21) |
| French Republic | 80–84 years | 2759.81 (2718.18, 2802.08) |
| French Republic | 85–89 years | 3286.52 (3232.41, 3341.55) |
| French Republic | 90–94 years | 3393.44 (3324.53, 3463.78) |
| French Republic | 95+ years | 3358.92 (3250.97, 3470.46) |
| Gabonese Republic | 30–34 years | 3.88 (2.25, 6.69) |
| Gabonese Republic | 35–39 years | 8.88 (5.96, 13.23) |
| Gabonese Republic | 40–44 years | 17.51 (12.79, 23.98) |
| Gabonese Republic | 45–49 years | 33.31 (25.78, 43.04) |
| Gabonese Republic | 50–54 years | 58.37 (47.30, 72.03) |
| Gabonese Republic | 55–59 years | 99.38 (83.14, 118.78) |
| Gabonese Republic | 60–64 years | 172.06 (147.34, 200.92) |
| Gabonese Republic | 65–69 years | 328.16 (286.07, 376.43) |
| Gabonese Republic | 70–74 years | 562.87 (494.16, 641.13) |
| Gabonese Republic | 75–79 years | 840.29 (735.78, 959.63) |
| Gabonese Republic | 80–84 years | 1076.01 (908.68, 1274.15) |
| Gabonese Republic | 85–89 years | 1181.16 (945.68, 1475.26) |
| Gabonese Republic | 90–94 years | 1176.06 (804.17, 1719.91) |
| Gabonese Republic | 95+ years | 1106.42 (489.61, 2500.25) |
| Georgia | 30–34 years | 3.27 (2.40, 4.46) |
| Georgia | 35–39 years | 7.32 (5.95, 9.00) |
| Georgia | 40–44 years | 14.51 (12.47, 16.89) |
| Georgia | 45–49 years | 28.80 (25.67, 32.31) |
| Georgia | 50–54 years | 53.44 (49.05, 58.24) |
| Georgia | 55–59 years | 99.74 (93.37, 106.55) |
| Georgia | 60–64 years | 187.36 (177.80, 197.45) |
| Georgia | 65–69 years | 375.93 (359.47, 393.15) |
| Georgia | 70–74 years | 632.96 (606.54, 660.52) |
| Georgia | 75–79 years | 859.64 (823.62, 897.23) |
| Georgia | 80–84 years | 958.46 (910.90, 1008.50) |
| Georgia | 85–89 years | 836.44 (785.68, 890.48) |
| Georgia | 90–94 years | 613.18 (559.27, 672.29) |
| Georgia | 95+ years | 432.43 (371.57, 503.25) |
| Grand Duchy of Luxembourg | 30–34 years | 5.64 (2.97, 10.70) |
| Grand Duchy of Luxembourg | 35–39 years | 10.79 (6.82, 17.07) |
| Grand Duchy of Luxembourg | 40–44 years | 19.23 (13.53, 27.33) |
| Grand Duchy of Luxembourg | 45–49 years | 37.73 (28.86, 49.32) |
| Grand Duchy of Luxembourg | 50–54 years | 70.92 (57.61, 87.29) |
| Grand Duchy of Luxembourg | 55–59 years | 129.67 (109.87, 153.05) |
| Grand Duchy of Luxembourg | 60–64 years | 250.47 (219.53, 285.78) |
| Grand Duchy of Luxembourg | 65–69 years | 562.53 (506.67, 624.56) |
| Grand Duchy of Luxembourg | 70–74 years | 1112.43 (1016.06, 1217.95) |
| Grand Duchy of Luxembourg | 75–79 years | 1919.52 (1762.21, 2090.88) |
| Grand Duchy of Luxembourg | 80–84 years | 2777.42 (2504.78, 3079.72) |
| Grand Duchy of Luxembourg | 85–89 years | 3196.86 (2845.91, 3591.08) |
| Grand Duchy of Luxembourg | 90–94 years | 3171.06 (2715.68, 3702.81) |
| Grand Duchy of Luxembourg | 95+ years | 2945.26 (2270.11, 3821.20) |
| Greenland | 30–34 years | 2.07 (0.11, 38.43) |
| Greenland | 35–39 years | 5.02 (0.76, 33.18) |
| Greenland | 40–44 years | 11.47 (3.07, 42.89) |
| Greenland | 45–49 years | 27.76 (10.90, 70.72) |
| Greenland | 50–54 years | 58.11 (28.64, 117.89) |
| Greenland | 55–59 years | 115.81 (65.25, 205.53) |
| Greenland | 60–64 years | 225.76 (138.91, 366.89) |
| Greenland | 65–69 years | 468.33 (306.68, 715.16) |
| Greenland | 70–74 years | 860.94 (578.21, 1281.93) |
| Greenland | 75–79 years | 1405.76 (934.39, 2114.94) |
| Greenland | 80–84 years | 1895.01 (1089.97, 3294.65) |
| Greenland | 85–89 years | 1896.63 (875.21, 4110.12) |
| Greenland | 90–94 years | 1536.14 (380.01, 6209.63) |
| Greenland | 95+ years | 1030.16 (29.85, 35550.05) |
| Grenada | 30–34 years | 4.82 (0.93, 24.92) |
| Grenada | 35–39 years | 11.82 (3.76, 37.19) |
| Grenada | 40–44 years | 23.99 (9.96, 57.81) |
| Grenada | 45–49 years | 44.74 (22.16, 90.31) |
| Grenada | 50–54 years | 75.14 (42.70, 132.23) |
| Grenada | 55–59 years | 115.10 (70.84, 187.01) |
| Grenada | 60–64 years | 174.92 (112.83, 271.17) |
| Grenada | 65–69 years | 290.17 (193.06, 436.14) |
| Grenada | 70–74 years | 467.23 (317.06, 688.52) |
| Grenada | 75–79 years | 705.63 (474.59, 1049.15) |
| Grenada | 80–84 years | 907.35 (538.45, 1529.01) |
| Grenada | 85–89 years | 982.73 (517.64, 1865.68) |
| Grenada | 90–94 years | 1037.26 (444.28, 2421.72) |
| Grenada | 95+ years | 1263.83 (393.75, 4056.53) |
| Guam | 30–34 years | 3.26 (0.75, 14.27) |
| Guam | 35–39 years | 7.04 (2.52, 19.65) |
| Guam | 40–44 years | 14.35 (6.61, 31.15) |
| Guam | 45–49 years | 32.42 (18.20, 57.78) |
| Guam | 50–54 years | 66.26 (42.07, 104.34) |
| Guam | 55–59 years | 129.08 (89.19, 186.82) |
| Guam | 60–64 years | 232.51 (169.92, 318.14) |
| Guam | 65–69 years | 402.10 (303.34, 533.01) |
| Guam | 70–74 years | 646.24 (492.14, 848.58) |
| Guam | 75–79 years | 1003.06 (765.00, 1315.21) |
| Guam | 80–84 years | 1407.75 (1015.94, 1950.67) |
| Guam | 85–89 years | 1722.31 (1197.94, 2476.21) |
| Guam | 90–94 years | 1947.32 (1261.38, 3006.26) |
| Guam | 95+ years | 2174.98 (1286.36, 3677.46) |
| Hashemite Kingdom of Jordan | 30–34 years | 2.06 (1.51, 2.81) |
| Hashemite Kingdom of Jordan | 35–39 years | 5.32 (4.26, 6.64) |
| Hashemite Kingdom of Jordan | 40–44 years | 11.52 (9.68, 13.70) |
| Hashemite Kingdom of Jordan | 45–49 years | 23.62 (20.49, 27.22) |
| Hashemite Kingdom of Jordan | 50–54 years | 43.62 (38.84, 48.99) |
| Hashemite Kingdom of Jordan | 55–59 years | 77.59 (70.39, 85.53) |
| Hashemite Kingdom of Jordan | 60–64 years | 134.56 (123.66, 146.43) |
| Hashemite Kingdom of Jordan | 65–69 years | 238.08 (221.18, 256.26) |
| Hashemite Kingdom of Jordan | 70–74 years | 432.91 (405.43, 462.25) |
| Hashemite Kingdom of Jordan | 75–79 years | 853.26 (802.34, 907.41) |
| Hashemite Kingdom of Jordan | 80–84 years | 1362.24 (1251.35, 1482.96) |
| Hashemite Kingdom of Jordan | 85–89 years | 1631.42 (1462.99, 1819.24) |
| Hashemite Kingdom of Jordan | 90–94 years | 1736.93 (1474.04, 2046.70) |
| Hashemite Kingdom of Jordan | 95+ years | 2042.57 (1579.93, 2640.69) |
| Hellenic Republic | 30–34 years | 5.26 (4.56, 6.07) |
| Hellenic Republic | 35–39 years | 10.18 (9.19, 11.28) |
| Hellenic Republic | 40–44 years | 18.36 (16.98, 19.85) |
| Hellenic Republic | 45–49 years | 36.14 (34.10, 38.31) |
| Hellenic Republic | 50–54 years | 68.08 (65.14, 71.15) |
| Hellenic Republic | 55–59 years | 125.16 (120.99, 129.47) |
| Hellenic Republic | 60–64 years | 235.04 (228.88, 241.37) |
| Hellenic Republic | 65–69 years | 491.52 (481.15, 502.11) |
| Hellenic Republic | 70–74 years | 926.48 (909.33, 943.95) |
| Hellenic Republic | 75–79 years | 1576.93 (1549.20, 1605.17) |
| Hellenic Republic | 80–84 years | 2275.61 (2228.82, 2323.38) |
| Hellenic Republic | 85–89 years | 2606.40 (2546.45, 2667.77) |
| Hellenic Republic | 90–94 years | 2619.74 (2538.73, 2703.33) |
| Hellenic Republic | 95+ years | 2667.77 (2516.45, 2828.19) |
| Hungary | 30–34 years | 2.21 (1.74, 2.81) |
| Hungary | 35–39 years | 4.81 (4.10, 5.64) |
| Hungary | 40–44 years | 9.69 (8.67, 10.82) |
| Hungary | 45–49 years | 20.87 (19.30, 22.56) |
| Hungary | 50–54 years | 42.85 (40.52, 45.31) |
| Hungary | 55–59 years | 90.33 (86.71, 94.09) |
| Hungary | 60–64 years | 185.13 (179.43, 191.01) |
| Hungary | 65–69 years | 397.65 (387.72, 407.84) |
| Hungary | 70–74 years | 722.41 (706.16, 739.03) |
| Hungary | 75–79 years | 1069.18 (1045.50, 1093.39) |
| Hungary | 80–84 years | 1301.57 (1266.01, 1338.12) |
| Hungary | 85–89 years | 1238.37 (1196.83, 1281.34) |
| Hungary | 90–94 years | 964.99 (912.98, 1019.96) |
| Hungary | 95+ years | 675.31 (594.04, 767.70) |
| Independent State of Papua New Guinea | 30–34 years | 2.90 (2.21, 3.80) |
| Independent State of Papua New Guinea | 35–39 years | 6.09 (4.97, 7.47) |
| Independent State of Papua New Guinea | 40–44 years | 12.07 (10.26, 14.20) |
| Independent State of Papua New Guinea | 45–49 years | 26.46 (23.25, 30.12) |
| Independent State of Papua New Guinea | 50–54 years | 52.85 (47.58, 58.71) |
| Independent State of Papua New Guinea | 55–59 years | 99.90 (91.42, 109.18) |
| Independent State of Papua New Guinea | 60–64 years | 179.71 (166.27, 194.24) |
| Independent State of Papua New Guinea | 65–69 years | 326.68 (304.34, 350.65) |
| Independent State of Papua New Guinea | 70–74 years | 539.49 (503.97, 577.51) |
| Independent State of Papua New Guinea | 75–79 years | 819.14 (763.27, 879.09) |
| Independent State of Papua New Guinea | 80–84 years | 1090.96 (995.57, 1195.49) |
| Independent State of Papua New Guinea | 85–89 years | 1235.89 (1095.54, 1394.22) |
| Independent State of Papua New Guinea | 90–94 years | 1328.32 (1088.94, 1620.31) |
| Independent State of Papua New Guinea | 95+ years | 1567.76 (1051.78, 2336.87) |
| Independent State of Samoa | 30–34 years | 3.14 (0.67, 14.76) |
| Independent State of Samoa | 35–39 years | 6.66 (2.14, 20.70) |
| Independent State of Samoa | 40–44 years | 13.35 (5.55, 32.08) |
| Independent State of Samoa | 45–49 years | 29.35 (14.94, 57.69) |
| Independent State of Samoa | 50–54 years | 59.62 (35.23, 100.91) |
| Independent State of Samoa | 55–59 years | 117.81 (77.09, 180.03) |
| Independent State of Samoa | 60–64 years | 216.34 (151.08, 309.78) |
| Independent State of Samoa | 65–69 years | 385.94 (280.24, 531.51) |
| Independent State of Samoa | 70–74 years | 645.58 (477.15, 873.48) |
| Independent State of Samoa | 75–79 years | 1058.43 (784.02, 1428.88) |
| Independent State of Samoa | 80–84 years | 1486.49 (1030.56, 2144.14) |
| Independent State of Samoa | 85–89 years | 1622.69 (1025.62, 2567.36) |
| Independent State of Samoa | 90–94 years | 1604.57 (762.67, 3375.82) |
| Independent State of Samoa | 95+ years | 1815.33 (394.40, 8355.58) |
| Ireland | 30–34 years | 5.18 (4.14, 6.47) |
| Ireland | 35–39 years | 10.10 (8.59, 11.88) |
| Ireland | 40–44 years | 18.48 (16.31, 20.95) |
| Ireland | 45–49 years | 37.57 (34.17, 41.30) |
| Ireland | 50–54 years | 72.29 (67.17, 77.80) |
| Ireland | 55–59 years | 132.96 (125.37, 141.02) |
| Ireland | 60–64 years | 249.70 (238.11, 261.86) |
| Ireland | 65–69 years | 526.36 (506.60, 546.89) |
| Ireland | 70–74 years | 1027.58 (993.84, 1062.47) |
| Ireland | 75–79 years | 1857.81 (1800.33, 1917.11) |
| Ireland | 80–84 years | 2826.45 (2720.01, 2937.06) |
| Ireland | 85–89 years | 3339.83 (3197.48, 3488.52) |
| Ireland | 90–94 years | 3376.88 (3185.11, 3580.20) |
| Ireland | 95+ years | 3303.87 (2988.87, 3652.07) |
| Islamic Republic of Afghanistan | 30–34 years | 3.00 (2.53, 3.55) |
| Islamic Republic of Afghanistan | 35–39 years | 8.10 (7.16, 9.17) |
| Islamic Republic of Afghanistan | 40–44 years | 17.60 (15.96, 19.41) |
| Islamic Republic of Afghanistan | 45–49 years | 34.69 (32.02, 37.59) |
| Islamic Republic of Afghanistan | 50–54 years | 60.02 (56.23, 64.06) |
| Islamic Republic of Afghanistan | 55–59 years | 98.37 (93.10, 103.94) |
| Islamic Republic of Afghanistan | 60–64 years | 163.06 (155.47, 171.01) |
| Islamic Republic of Afghanistan | 65–69 years | 291.02 (279.01, 303.54) |
| Islamic Republic of Afghanistan | 70–74 years | 498.04 (478.53, 518.34) |
| Islamic Republic of Afghanistan | 75–79 years | 815.81 (783.12, 849.86) |
| Islamic Republic of Afghanistan | 80–84 years | 1132.47 (1074.40, 1193.68) |
| Islamic Republic of Afghanistan | 85–89 years | 1220.27 (1133.93, 1313.19) |
| Islamic Republic of Afghanistan | 90–94 years | 1169.20 (1019.49, 1340.91) |
| Islamic Republic of Afghanistan | 95+ years | 1156.26 (849.86, 1573.12) |
| Islamic Republic of Iran | 30–34 years | 2.90 (2.70, 3.11) |
| Islamic Republic of Iran | 35–39 years | 7.27 (6.91, 7.64) |
| Islamic Republic of Iran | 40–44 years | 15.48 (14.89, 16.10) |
| Islamic Republic of Iran | 45–49 years | 31.44 (30.45, 32.47) |
| Islamic Republic of Iran | 50–54 years | 57.79 (56.28, 59.35) |
| Islamic Republic of Iran | 55–59 years | 101.31 (99.05, 103.62) |
| Islamic Republic of Iran | 60–64 years | 175.26 (171.89, 178.70) |
| Islamic Republic of Iran | 65–69 years | 322.81 (317.36, 328.35) |
| Islamic Republic of Iran | 70–74 years | 585.87 (576.83, 595.06) |
| Islamic Republic of Iran | 75–79 years | 1064.09 (1048.44, 1079.97) |
| Islamic Republic of Iran | 80–84 years | 1633.75 (1604.29, 1663.76) |
| Islamic Republic of Iran | 85–89 years | 1980.16 (1937.79, 2023.45) |
| Islamic Republic of Iran | 90–94 years | 2152.29 (2084.99, 2221.76) |
| Islamic Republic of Iran | 95+ years | 2446.30 (2306.70, 2594.35) |
| Islamic Republic of Mauritania | 30–34 years | 3.55 (2.32, 5.42) |
| Islamic Republic of Mauritania | 35–39 years | 7.56 (5.50, 10.39) |
| Islamic Republic of Mauritania | 40–44 years | 13.79 (10.66, 17.83) |
| Islamic Republic of Mauritania | 45–49 years | 23.59 (18.97, 29.32) |
| Islamic Republic of Mauritania | 50–54 years | 39.14 (32.56, 47.06) |
| Islamic Republic of Mauritania | 55–59 years | 68.18 (58.38, 79.63) |
| Islamic Republic of Mauritania | 60–64 years | 124.56 (109.46, 141.74) |
| Islamic Republic of Mauritania | 65–69 years | 251.77 (225.90, 280.61) |
| Islamic Republic of Mauritania | 70–74 years | 478.61 (434.20, 527.56) |
| Islamic Republic of Mauritania | 75–79 years | 879.95 (801.86, 965.65) |
| Islamic Republic of Mauritania | 80–84 years | 1301.09 (1159.97, 1459.37) |
| Islamic Republic of Mauritania | 85–89 years | 1408.17 (1223.62, 1620.57) |
| Islamic Republic of Mauritania | 90–94 years | 1303.83 (1045.77, 1625.57) |
| Islamic Republic of Mauritania | 95+ years | 1222.87 (799.54, 1870.32) |
| Islamic Republic of Pakistan | 30–34 years | 3.94 (3.76, 4.14) |
| Islamic Republic of Pakistan | 35–39 years | 9.45 (9.12, 9.79) |
| Islamic Republic of Pakistan | 40–44 years | 18.79 (18.27, 19.33) |
| Islamic Republic of Pakistan | 45–49 years | 34.14 (33.35, 34.94) |
| Islamic Republic of Pakistan | 50–54 years | 57.66 (56.55, 58.79) |
| Islamic Republic of Pakistan | 55–59 years | 96.03 (94.42, 97.67) |
| Islamic Republic of Pakistan | 60–64 years | 168.96 (166.48, 171.48) |
| Islamic Republic of Pakistan | 65–69 years | 344.71 (340.36, 349.12) |
| Islamic Republic of Pakistan | 70–74 years | 632.31 (624.94, 639.76) |
| Islamic Republic of Pakistan | 75–79 years | 1006.07 (994.24, 1018.04) |
| Islamic Republic of Pakistan | 80–84 years | 1312.02 (1292.18, 1332.17) |
| Islamic Republic of Pakistan | 85–89 years | 1261.69 (1236.40, 1287.50) |
| Islamic Republic of Pakistan | 90–94 years | 1050.09 (1014.34, 1087.10) |
| Islamic Republic of Pakistan | 95+ years | 968.50 (901.86, 1040.06) |
| Jamaica | 30–34 years | 4.46 (3.25, 6.13) |
| Jamaica | 35–39 years | 11.29 (9.06, 14.06) |
| Jamaica | 40–44 years | 23.70 (19.96, 28.13) |
| Jamaica | 45–49 years | 45.89 (39.89, 52.78) |
| Jamaica | 50–54 years | 79.21 (70.51, 89.00) |
| Jamaica | 55–59 years | 124.29 (112.27, 137.59) |
| Jamaica | 60–64 years | 185.61 (169.59, 203.13) |
| Jamaica | 65–69 years | 281.51 (259.01, 305.97) |
| Jamaica | 70–74 years | 427.60 (394.67, 463.27) |
| Jamaica | 75–79 years | 662.38 (611.76, 717.18) |
| Jamaica | 80–84 years | 897.41 (817.02, 985.72) |
| Jamaica | 85–89 years | 991.42 (891.45, 1102.60) |
| Jamaica | 90–94 years | 1048.89 (920.84, 1194.74) |
| Jamaica | 95+ years | 1331.40 (1148.50, 1543.42) |
| Japan | 30–34 years | 3.35 (2.92, 3.84) |
| Japan | 35–39 years | 7.69 (7.02, 8.43) |
| Japan | 40–44 years | 15.30 (14.33, 16.33) |
| Japan | 45–49 years | 29.43 (28.07, 30.86) |
| Japan | 50–54 years | 53.28 (51.43, 55.20) |
| Japan | 55–59 years | 96.72 (94.15, 99.37) |
| Japan | 60–64 years | 168.93 (165.36, 172.59) |
| Japan | 65–69 years | 298.15 (292.85, 303.54) |
| Japan | 70–74 years | 479.65 (471.83, 487.61) |
| Japan | 75–79 years | 686.35 (675.29, 697.59) |
| Japan | 80–84 years | 843.15 (826.83, 859.80) |
| Japan | 85–89 years | 841.54 (822.76, 860.75) |
| Japan | 90–94 years | 737.60 (715.51, 760.38) |
| Japan | 95+ years | 636.55 (606.71, 667.87) |
| Kingdom of Bahrain | 30–34 years | 2.72 (1.52, 4.88) |
| Kingdom of Bahrain | 35–39 years | 6.84 (4.46, 10.47) |
| Kingdom of Bahrain | 40–44 years | 14.64 (10.34, 20.74) |
| Kingdom of Bahrain | 45–49 years | 30.06 (22.30, 40.52) |
| Kingdom of Bahrain | 50–54 years | 56.53 (43.59, 73.32) |
| Kingdom of Bahrain | 55–59 years | 103.66 (82.26, 130.65) |
| Kingdom of Bahrain | 60–64 years | 189.39 (153.71, 233.36) |
| Kingdom of Bahrain | 65–69 years | 377.37 (312.65, 455.49) |
| Kingdom of Bahrain | 70–74 years | 717.39 (598.77, 859.50) |
| Kingdom of Bahrain | 75–79 years | 1323.15 (1098.95, 1593.08) |
| Kingdom of Bahrain | 80–84 years | 2031.48 (1606.76, 2568.46) |
| Kingdom of Bahrain | 85–89 years | 2412.95 (1732.00, 3361.64) |
| Kingdom of Bahrain | 90–94 years | 2408.39 (1112.79, 5212.44) |
| Kingdom of Bahrain | 95+ years | 2257.04 (171.54, 29697.79) |
| Kingdom of Belgium | 30–34 years | 5.32 (4.61, 6.15) |
| Kingdom of Belgium | 35–39 years | 10.36 (9.36, 11.46) |
| Kingdom of Belgium | 40–44 years | 18.62 (17.25, 20.10) |
| Kingdom of Belgium | 45–49 years | 36.38 (34.36, 38.53) |
| Kingdom of Belgium | 50–54 years | 68.43 (65.49, 71.50) |
| Kingdom of Belgium | 55–59 years | 125.88 (121.61, 130.30) |
| Kingdom of Belgium | 60–64 years | 238.34 (231.84, 245.02) |
| Kingdom of Belgium | 65–69 years | 507.54 (496.43, 518.89) |
| Kingdom of Belgium | 70–74 years | 980.88 (962.03, 1000.10) |
| Kingdom of Belgium | 75–79 years | 1705.84 (1674.68, 1737.59) |
| Kingdom of Belgium | 80–84 years | 2496.25 (2442.86, 2550.80) |
| Kingdom of Belgium | 85–89 years | 2893.57 (2825.05, 2963.76) |
| Kingdom of Belgium | 90–94 years | 2908.32 (2819.88, 2999.52) |
| Kingdom of Belgium | 95+ years | 2868.38 (2727.84, 3016.15) |
| Kingdom of Bhutan | 30–34 years | 3.70 (1.84, 7.46) |
| Kingdom of Bhutan | 35–39 years | 8.68 (5.19, 14.54) |
| Kingdom of Bhutan | 40–44 years | 17.26 (11.43, 26.08) |
| Kingdom of Bhutan | 45–49 years | 32.10 (22.86, 45.07) |
| Kingdom of Bhutan | 50–54 years | 55.88 (42.16, 74.07) |
| Kingdom of Bhutan | 55–59 years | 95.92 (75.57, 121.75) |
| Kingdom of Bhutan | 60–64 years | 172.20 (140.60, 210.89) |
| Kingdom of Bhutan | 65–69 years | 353.19 (297.30, 419.58) |
| Kingdom of Bhutan | 70–74 years | 659.18 (563.47, 771.14) |
| Kingdom of Bhutan | 75–79 years | 1072.40 (918.81, 1251.66) |
| Kingdom of Bhutan | 80–84 years | 1447.76 (1186.22, 1766.96) |
| Kingdom of Bhutan | 85–89 years | 1495.50 (1154.29, 1937.57) |
| Kingdom of Bhutan | 90–94 years | 1330.66 (851.86, 2078.58) |
| Kingdom of Bhutan | 95+ years | 1236.17 (489.79, 3119.96) |
| Kingdom of Cambodia | 30–34 years | 3.67 (3.09, 4.36) |
| Kingdom of Cambodia | 35–39 years | 7.84 (6.90, 8.91) |
| Kingdom of Cambodia | 40–44 years | 14.58 (13.15, 16.17) |
| Kingdom of Cambodia | 45–49 years | 27.34 (25.12, 29.75) |
| Kingdom of Cambodia | 50–54 years | 49.28 (45.99, 52.81) |
| Kingdom of Cambodia | 55–59 years | 89.91 (84.82, 95.30) |
| Kingdom of Cambodia | 60–64 years | 161.71 (153.81, 170.02) |
| Kingdom of Cambodia | 65–69 years | 301.92 (288.90, 315.52) |
| Kingdom of Cambodia | 70–74 years | 522.11 (501.18, 543.93) |
| Kingdom of Cambodia | 75–79 years | 818.75 (785.40, 853.51) |
| Kingdom of Cambodia | 80–84 years | 1055.79 (998.56, 1116.31) |
| Kingdom of Cambodia | 85–89 years | 989.94 (913.21, 1073.11) |
| Kingdom of Cambodia | 90–94 years | 905.84 (777.81, 1054.95) |
| Kingdom of Cambodia | 95+ years | 1320.14 (998.82, 1744.84) |
| Kingdom of Denmark | 30–34 years | 4.83 (3.93, 5.92) |
| Kingdom of Denmark | 35–39 years | 9.44 (8.16, 10.91) |
| Kingdom of Denmark | 40–44 years | 17.06 (15.29, 19.03) |
| Kingdom of Denmark | 45–49 years | 33.71 (31.11, 36.52) |
| Kingdom of Denmark | 50–54 years | 63.71 (59.95, 67.70) |
| Kingdom of Denmark | 55–59 years | 116.46 (110.98, 122.21) |
| Kingdom of Denmark | 60–64 years | 218.73 (210.41, 227.37) |
| Kingdom of Denmark | 65–69 years | 464.62 (450.35, 479.34) |
| Kingdom of Denmark | 70–74 years | 916.13 (891.83, 941.10) |
| Kingdom of Denmark | 75–79 years | 1641.64 (1600.39, 1683.97) |
| Kingdom of Denmark | 80–84 years | 2507.17 (2429.53, 2587.29) |
| Kingdom of Denmark | 85–89 years | 3054.40 (2948.12, 3164.51) |
| Kingdom of Denmark | 90–94 years | 3236.71 (3092.64, 3387.48) |
| Kingdom of Denmark | 95+ years | 3397.79 (3172.85, 3638.68) |
| Kingdom of Eswatini | 30–34 years | 4.09 (2.15, 7.76) |
| Kingdom of Eswatini | 35–39 years | 8.96 (5.48, 14.65) |
| Kingdom of Eswatini | 40–44 years | 16.99 (11.35, 25.43) |
| Kingdom of Eswatini | 45–49 years | 31.04 (22.11, 43.57) |
| Kingdom of Eswatini | 50–54 years | 51.75 (38.94, 68.78) |
| Kingdom of Eswatini | 55–59 years | 80.33 (62.46, 103.33) |
| Kingdom of Eswatini | 60–64 years | 128.02 (102.33, 160.15) |
| Kingdom of Eswatini | 65–69 years | 236.82 (194.11, 288.93) |
| Kingdom of Eswatini | 70–74 years | 422.25 (349.16, 510.63) |
| Kingdom of Eswatini | 75–79 years | 709.20 (582.21, 863.90) |
| Kingdom of Eswatini | 80–84 years | 993.70 (761.34, 1296.98) |
| Kingdom of Eswatini | 85–89 years | 1129.30 (768.27, 1660.00) |
| Kingdom of Eswatini | 90–94 years | 1129.38 (538.51, 2368.60) |
| Kingdom of Eswatini | 95+ years | 1017.54 (178.88, 5788.10) |
| Kingdom of Lesotho | 30–34 years | 3.62 (2.17, 6.04) |
| Kingdom of Lesotho | 35–39 years | 7.77 (5.29, 11.41) |
| Kingdom of Lesotho | 40–44 years | 14.39 (10.59, 19.55) |
| Kingdom of Lesotho | 45–49 years | 25.78 (20.09, 33.08) |
| Kingdom of Lesotho | 50–54 years | 43.09 (35.14, 52.83) |
| Kingdom of Lesotho | 55–59 years | 69.32 (58.06, 82.75) |
| Kingdom of Lesotho | 60–64 years | 113.31 (96.83, 132.61) |
| Kingdom of Lesotho | 65–69 years | 205.90 (178.73, 237.21) |
| Kingdom of Lesotho | 70–74 years | 365.13 (318.85, 418.12) |
| Kingdom of Lesotho | 75–79 years | 633.68 (551.18, 728.54) |
| Kingdom of Lesotho | 80–84 years | 896.77 (753.31, 1067.55) |
| Kingdom of Lesotho | 85–89 years | 966.16 (763.95, 1221.88) |
| Kingdom of Lesotho | 90–94 years | 917.05 (607.43, 1384.50) |
| Kingdom of Lesotho | 95+ years | 895.01 (398.10, 2012.20) |
| Kingdom of Morocco | 30–34 years | 2.20 (1.96, 2.46) |
| Kingdom of Morocco | 35–39 years | 5.94 (5.50, 6.42) |
| Kingdom of Morocco | 40–44 years | 13.37 (12.61, 14.17) |
| Kingdom of Morocco | 45–49 years | 28.29 (27.02, 29.63) |
| Kingdom of Morocco | 50–54 years | 53.52 (51.53, 55.59) |
| Kingdom of Morocco | 55–59 years | 96.32 (93.26, 99.47) |
| Kingdom of Morocco | 60–64 years | 172.79 (168.04, 177.66) |
| Kingdom of Morocco | 65–69 years | 335.30 (327.31, 343.49) |
| Kingdom of Morocco | 70–74 years | 613.66 (600.45, 627.16) |
| Kingdom of Morocco | 75–79 years | 1056.52 (1034.34, 1079.19) |
| Kingdom of Morocco | 80–84 years | 1545.67 (1504.37, 1588.10) |
| Kingdom of Morocco | 85–89 years | 1787.30 (1727.96, 1848.69) |
| Kingdom of Morocco | 90–94 years | 1822.85 (1729.10, 1921.69) |
| Kingdom of Morocco | 95+ years | 1904.33 (1725.21, 2102.05) |
| Kingdom of Norway | 30–34 years | 2.12 (1.67, 2.70) |
| Kingdom of Norway | 35–39 years | 4.18 (3.50, 5.00) |
| Kingdom of Norway | 40–44 years | 7.47 (6.49, 8.60) |
| Kingdom of Norway | 45–49 years | 13.89 (12.41, 15.55) |
| Kingdom of Norway | 50–54 years | 26.20 (23.90, 28.72) |
| Kingdom of Norway | 55–59 years | 51.55 (47.79, 55.59) |
| Kingdom of Norway | 60–64 years | 106.62 (100.23, 113.42) |
| Kingdom of Norway | 65–69 years | 245.97 (234.15, 258.39) |
| Kingdom of Norway | 70–74 years | 538.75 (516.45, 562.02) |
| Kingdom of Norway | 75–79 years | 1109.34 (1066.91, 1153.45) |
| Kingdom of Norway | 80–84 years | 1961.29 (1865.33, 2062.20) |
| Kingdom of Norway | 85–89 years | 2833.76 (2679.12, 2997.33) |
| Kingdom of Norway | 90–94 years | 3588.13 (3344.41, 3849.62) |
| Kingdom of Norway | 95+ years | 4356.50 (3925.86, 4834.37) |
| Kingdom of Saudi Arabia | 30–34 years | 2.24 (1.99, 2.52) |
| Kingdom of Saudi Arabia | 35–39 years | 5.97 (5.48, 6.50) |
| Kingdom of Saudi Arabia | 40–44 years | 13.40 (12.52, 14.35) |
| Kingdom of Saudi Arabia | 45–49 years | 28.71 (27.10, 30.40) |
| Kingdom of Saudi Arabia | 50–54 years | 54.97 (52.31, 57.77) |
| Kingdom of Saudi Arabia | 55–59 years | 100.74 (96.42, 105.24) |
| Kingdom of Saudi Arabia | 60–64 years | 182.48 (175.37, 189.88) |
| Kingdom of Saudi Arabia | 65–69 years | 352.49 (340.06, 365.36) |
| Kingdom of Saudi Arabia | 70–74 years | 709.94 (686.43, 734.27) |
| Kingdom of Saudi Arabia | 75–79 years | 1549.14 (1498.41, 1601.58) |
| Kingdom of Saudi Arabia | 80–84 years | 2577.24 (2472.39, 2686.55) |
| Kingdom of Saudi Arabia | 85–89 years | 2982.23 (2834.48, 3137.68) |
| Kingdom of Saudi Arabia | 90–94 years | 2830.19 (2612.65, 3065.85) |
| Kingdom of Saudi Arabia | 95+ years | 2557.84 (2139.79, 3057.58) |
| Kingdom of Spain | 30–34 years | 4.37 (3.60, 5.31) |
| Kingdom of Spain | 35–39 years | 8.77 (7.62, 10.11) |
| Kingdom of Spain | 40–44 years | 16.52 (14.82, 18.40) |
| Kingdom of Spain | 45–49 years | 34.40 (31.72, 37.30) |
| Kingdom of Spain | 50–54 years | 68.39 (64.29, 72.75) |
| Kingdom of Spain | 55–59 years | 132.60 (126.45, 139.04) |
| Kingdom of Spain | 60–64 years | 276.09 (266.41, 286.12) |
| Kingdom of Spain | 65–69 years | 669.03 (651.45, 687.08) |
| Kingdom of Spain | 70–74 years | 1358.54 (1327.45, 1390.35) |
| Kingdom of Spain | 75–79 years | 2341.36 (2290.24, 2393.62) |
| Kingdom of Spain | 80–84 years | 3353.21 (3268.65, 3439.97) |
| Kingdom of Spain | 85–89 years | 3810.26 (3705.38, 3918.11) |
| Kingdom of Spain | 90–94 years | 3739.63 (3610.82, 3873.04) |
| Kingdom of Spain | 95+ years | 3518.57 (3324.75, 3723.68) |
| Kingdom of Sweden | 30–34 years | 5.95 (5.08, 6.97) |
| Kingdom of Sweden | 35–39 years | 11.41 (10.19, 12.78) |
| Kingdom of Sweden | 40–44 years | 19.81 (18.19, 21.58) |
| Kingdom of Sweden | 45–49 years | 36.08 (33.85, 38.46) |
| Kingdom of Sweden | 50–54 years | 64.18 (61.15, 67.36) |
| Kingdom of Sweden | 55–59 years | 114.71 (110.42, 119.17) |
| Kingdom of Sweden | 60–64 years | 211.93 (205.59, 218.45) |
| Kingdom of Sweden | 65–69 years | 437.46 (427.01, 448.16) |
| Kingdom of Sweden | 70–74 years | 827.12 (810.03, 844.57) |
| Kingdom of Sweden | 75–79 years | 1389.76 (1362.67, 1417.38) |
| Kingdom of Sweden | 80–84 years | 2003.22 (1955.31, 2052.30) |
| Kingdom of Sweden | 85–89 years | 2356.84 (2293.91, 2421.50) |
| Kingdom of Sweden | 90–94 years | 2470.42 (2386.78, 2556.98) |
| Kingdom of Sweden | 95+ years | 2602.53 (2469.52, 2742.69) |
| Kingdom of Thailand | 30–34 years | 4.10 (3.85, 4.37) |
| Kingdom of Thailand | 35–39 years | 8.71 (8.33, 9.11) |
| Kingdom of Thailand | 40–44 years | 16.34 (15.78, 16.92) |
| Kingdom of Thailand | 45–49 years | 30.92 (30.07, 31.78) |
| Kingdom of Thailand | 50–54 years | 56.72 (55.47, 58.00) |
| Kingdom of Thailand | 55–59 years | 107.50 (105.56, 109.48) |
| Kingdom of Thailand | 60–64 years | 198.47 (195.46, 201.53) |
| Kingdom of Thailand | 65–69 years | 370.74 (365.83, 375.72) |
| Kingdom of Thailand | 70–74 years | 634.91 (627.17, 642.74) |
| Kingdom of Thailand | 75–79 years | 994.37 (982.44, 1006.45) |
| Kingdom of Thailand | 80–84 years | 1332.90 (1312.54, 1353.57) |
| Kingdom of Thailand | 85–89 years | 1456.89 (1429.98, 1484.30) |
| Kingdom of Thailand | 90–94 years | 1506.57 (1468.60, 1545.53) |
| Kingdom of Thailand | 95+ years | 1879.74 (1813.00, 1948.94) |
| Kingdom of the Netherlands | 30–34 years | 5.71 (4.78, 6.82) |
| Kingdom of the Netherlands | 35–39 years | 11.08 (9.77, 12.56) |
| Kingdom of the Netherlands | 40–44 years | 20.31 (18.49, 22.30) |
| Kingdom of the Netherlands | 45–49 years | 41.33 (38.60, 44.26) |
| Kingdom of the Netherlands | 50–54 years | 79.56 (75.55, 83.79) |
| Kingdom of the Netherlands | 55–59 years | 148.72 (142.82, 154.87) |
| Kingdom of the Netherlands | 60–64 years | 285.98 (277.00, 295.25) |
| Kingdom of the Netherlands | 65–69 years | 615.10 (599.58, 631.01) |
| Kingdom of the Netherlands | 70–74 years | 1168.60 (1142.61, 1195.18) |
| Kingdom of the Netherlands | 75–79 years | 1978.26 (1936.75, 2020.66) |
| Kingdom of the Netherlands | 80–84 years | 2792.03 (2722.98, 2862.84) |
| Kingdom of the Netherlands | 85–89 years | 3063.03 (2979.09, 3149.33) |
| Kingdom of the Netherlands | 90–94 years | 2887.08 (2785.53, 2992.33) |
| Kingdom of the Netherlands | 95+ years | 2642.58 (2496.43, 2797.29) |
| Kingdom of Tonga | 30–34 years | 2.99 (0.34, 26.58) |
| Kingdom of Tonga | 35–39 years | 6.32 (1.27, 31.43) |
| Kingdom of Tonga | 40–44 years | 12.56 (3.67, 43.05) |
| Kingdom of Tonga | 45–49 years | 27.36 (10.75, 69.63) |
| Kingdom of Tonga | 50–54 years | 54.91 (27.04, 111.54) |
| Kingdom of Tonga | 55–59 years | 106.19 (60.24, 187.19) |
| Kingdom of Tonga | 60–64 years | 194.03 (120.53, 312.36) |
| Kingdom of Tonga | 65–69 years | 352.72 (231.61, 537.16) |
| Kingdom of Tonga | 70–74 years | 593.61 (399.07, 882.98) |
| Kingdom of Tonga | 75–79 years | 953.82 (642.87, 1415.17) |
| Kingdom of Tonga | 80–84 years | 1328.79 (827.40, 2134.01) |
| Kingdom of Tonga | 85–89 years | 1510.12 (860.20, 2651.10) |
| Kingdom of Tonga | 90–94 years | 1556.39 (698.43, 3468.30) |
| Kingdom of Tonga | 95+ years | 1699.30 (442.86, 6520.45) |
| Kyrgyz Republic | 30–34 years | 3.20 (2.35, 4.36) |
| Kyrgyz Republic | 35–39 years | 6.90 (5.54, 8.60) |
| Kyrgyz Republic | 40–44 years | 13.33 (11.23, 15.81) |
| Kyrgyz Republic | 45–49 years | 26.05 (22.68, 29.93) |
| Kyrgyz Republic | 50–54 years | 47.53 (42.47, 53.18) |
| Kyrgyz Republic | 55–59 years | 86.59 (78.89, 95.05) |
| Kyrgyz Republic | 60–64 years | 161.60 (149.42, 174.78) |
| Kyrgyz Republic | 65–69 years | 331.13 (309.27, 354.54) |
| Kyrgyz Republic | 70–74 years | 578.87 (542.96, 617.16) |
| Kyrgyz Republic | 75–79 years | 827.29 (775.85, 882.15) |
| Kyrgyz Republic | 80–84 years | 970.92 (898.26, 1049.47) |
| Kyrgyz Republic | 85–89 years | 888.95 (805.27, 981.33) |
| Kyrgyz Republic | 90–94 years | 682.19 (577.23, 806.24) |
| Kyrgyz Republic | 95+ years | 490.84 (325.99, 739.06) |
| Lao People's Democratic Republic | 30–34 years | 4.25 (3.28, 5.52) |
| Lao People's Democratic Republic | 35–39 years | 9.06 (7.47, 11.00) |
| Lao People's Democratic Republic | 40–44 years | 16.74 (14.31, 19.59) |
| Lao People's Democratic Republic | 45–49 years | 30.49 (26.79, 34.71) |
| Lao People's Democratic Republic | 50–54 years | 53.15 (47.81, 59.09) |
| Lao People's Democratic Republic | 55–59 years | 94.51 (86.40, 103.39) |
| Lao People's Democratic Republic | 60–64 years | 167.26 (154.67, 180.86) |
| Lao People's Democratic Republic | 65–69 years | 308.74 (287.97, 331.01) |
| Lao People's Democratic Republic | 70–74 years | 525.34 (491.88, 561.09) |
| Lao People's Democratic Republic | 75–79 years | 802.53 (750.65, 857.99) |
| Lao People's Democratic Republic | 80–84 years | 1008.91 (925.00, 1100.43) |
| Lao People's Democratic Republic | 85–89 years | 938.78 (831.40, 1060.03) |
| Lao People's Democratic Republic | 90–94 years | 855.25 (687.34, 1064.17) |
| Lao People's Democratic Republic | 95+ years | 1177.42 (782.27, 1772.17) |
| Lebanese Republic | 30–34 years | 2.26 (1.60, 3.18) |
| Lebanese Republic | 35–39 years | 5.89 (4.68, 7.43) |
| Lebanese Republic | 40–44 years | 12.94 (10.91, 15.34) |
| Lebanese Republic | 45–49 years | 27.05 (23.76, 30.78) |
| Lebanese Republic | 50–54 years | 50.69 (45.75, 56.17) |
| Lebanese Republic | 55–59 years | 90.10 (82.90, 97.94) |
| Lebanese Republic | 60–64 years | 159.29 (148.78, 170.54) |
| Lebanese Republic | 65–69 years | 303.95 (287.18, 321.71) |
| Lebanese Republic | 70–74 years | 570.70 (542.99, 599.84) |
| Lebanese Republic | 75–79 years | 1079.27 (1030.34, 1130.52) |
| Lebanese Republic | 80–84 years | 1656.39 (1562.72, 1755.67) |
| Lebanese Republic | 85–89 years | 1790.73 (1670.10, 1920.08) |
| Lebanese Republic | 90–94 years | 1665.35 (1504.62, 1843.25) |
| Lebanese Republic | 95+ years | 1910.63 (1618.88, 2254.95) |
| Malaysia | 30–34 years | 3.52 (3.14, 3.95) |
| Malaysia | 35–39 years | 7.61 (7.00, 8.27) |
| Malaysia | 40–44 years | 14.53 (13.60, 15.52) |
| Malaysia | 45–49 years | 28.07 (26.61, 29.61) |
| Malaysia | 50–54 years | 52.01 (49.80, 54.32) |
| Malaysia | 55–59 years | 97.80 (94.32, 101.41) |
| Malaysia | 60–64 years | 184.64 (179.11, 190.35) |
| Malaysia | 65–69 years | 373.90 (364.26, 383.79) |
| Malaysia | 70–74 years | 672.04 (655.89, 688.59) |
| Malaysia | 75–79 years | 1023.73 (998.81, 1049.28) |
| Malaysia | 80–84 years | 1330.49 (1287.60, 1374.81) |
| Malaysia | 85–89 years | 1479.73 (1418.99, 1543.06) |
| Malaysia | 90–94 years | 1620.74 (1524.32, 1723.25) |
| Malaysia | 95+ years | 2313.37 (2113.89, 2531.68) |
| Mongolia | 30–34 years | 3.01 (1.95, 4.63) |
| Mongolia | 35–39 years | 6.59 (4.81, 9.03) |
| Mongolia | 40–44 years | 12.75 (9.90, 16.41) |
| Mongolia | 45–49 years | 24.61 (20.02, 30.26) |
| Mongolia | 50–54 years | 45.97 (38.90, 54.32) |
| Mongolia | 55–59 years | 89.62 (78.16, 102.76) |
| Mongolia | 60–64 years | 170.18 (151.44, 191.25) |
| Mongolia | 65–69 years | 331.71 (299.26, 367.68) |
| Mongolia | 70–74 years | 561.44 (509.15, 619.11) |
| Mongolia | 75–79 years | 791.82 (716.35, 875.24) |
| Mongolia | 80–84 years | 935.34 (821.83, 1064.52) |
| Mongolia | 85–89 years | 902.97 (755.27, 1079.55) |
| Mongolia | 90–94 years | 724.63 (502.84, 1044.24) |
| Mongolia | 95+ years | 472.89 (166.06, 1346.63) |
| Montenegro | 30–34 years | 2.38 (0.94, 6.00) |
| Montenegro | 35–39 years | 5.21 (2.81, 9.68) |
| Montenegro | 40–44 years | 10.63 (6.87, 16.44) |
| Montenegro | 45–49 years | 23.06 (16.89, 31.48) |
| Montenegro | 50–54 years | 47.20 (37.63, 59.21) |
| Montenegro | 55–59 years | 98.37 (83.16, 116.38) |
| Montenegro | 60–64 years | 202.49 (177.71, 230.71) |
| Montenegro | 65–69 years | 443.89 (399.29, 493.47) |
| Montenegro | 70–74 years | 822.73 (747.50, 905.53) |
| Montenegro | 75–79 years | 1284.20 (1168.04, 1411.91) |
| Montenegro | 80–84 years | 1633.43 (1451.96, 1837.59) |
| Montenegro | 85–89 years | 1516.80 (1301.22, 1768.10) |
| Montenegro | 90–94 years | 1107.04 (853.30, 1436.22) |
| Montenegro | 95+ years | 746.06 (447.84, 1242.86) |
| New Zealand | 30–34 years | 1.81 (1.22, 2.67) |
| New Zealand | 35–39 years | 3.76 (2.86, 4.94) |
| New Zealand | 40–44 years | 6.93 (5.65, 8.50) |
| New Zealand | 45–49 years | 12.89 (11.02, 15.07) |
| New Zealand | 50–54 years | 24.49 (21.72, 27.61) |
| New Zealand | 55–59 years | 51.98 (47.48, 56.90) |
| New Zealand | 60–64 years | 113.77 (106.26, 121.82) |
| New Zealand | 65–69 years | 268.49 (254.75, 282.96) |
| New Zealand | 70–74 years | 524.37 (500.99, 548.84) |
| New Zealand | 75–79 years | 857.27 (820.65, 895.53) |
| New Zealand | 80–84 years | 1145.27 (1085.82, 1207.97) |
| New Zealand | 85–89 years | 1190.11 (1119.60, 1265.06) |
| New Zealand | 90–94 years | 1077.50 (990.71, 1171.89) |
| New Zealand | 95+ years | 969.05 (832.62, 1127.84) |
| North Macedonia | 30–34 years | 2.25 (1.36, 3.73) |
| North Macedonia | 35–39 years | 5.04 (3.60, 7.06) |
| North Macedonia | 40–44 years | 10.56 (8.33, 13.39) |
| North Macedonia | 45–49 years | 23.57 (19.92, 27.89) |
| North Macedonia | 50–54 years | 48.41 (42.77, 54.80) |
| North Macedonia | 55–59 years | 98.82 (89.98, 108.52) |
| North Macedonia | 60–64 years | 201.30 (187.23, 216.44) |
| North Macedonia | 65–69 years | 442.63 (417.63, 469.13) |
| North Macedonia | 70–74 years | 832.74 (790.11, 877.67) |
| North Macedonia | 75–79 years | 1344.33 (1275.86, 1416.48) |
| North Macedonia | 80–84 years | 1770.29 (1655.38, 1893.17) |
| North Macedonia | 85–89 years | 1714.72 (1550.77, 1896.01) |
| North Macedonia | 90–94 years | 1297.48 (949.13, 1773.66) |
| North Macedonia | 95+ years | 812.39 (321.21, 2054.67) |
| Northern Mariana Islands | 30–34 years | 3.50 (0.38, 32.34) |
| Northern Mariana Islands | 35–39 years | 7.48 (1.52, 36.93) |
| Northern Mariana Islands | 40–44 years | 15.35 (4.45, 53.00) |
| Northern Mariana Islands | 45–49 years | 34.13 (12.58, 92.56) |
| Northern Mariana Islands | 50–54 years | 68.75 (30.02, 157.47) |
| Northern Mariana Islands | 55–59 years | 128.86 (61.72, 269.01) |
| Northern Mariana Islands | 60–64 years | 233.73 (119.88, 455.72) |
| Northern Mariana Islands | 65–69 years | 436.29 (234.11, 813.09) |
| Northern Mariana Islands | 70–74 years | 739.25 (408.53, 1337.69) |
| Northern Mariana Islands | 75–79 years | 1157.36 (635.72, 2107.02) |
| Northern Mariana Islands | 80–84 years | 1581.05 (735.46, 3398.85) |
| Northern Mariana Islands | 85–89 years | 1841.72 (711.62, 4766.51) |
| Northern Mariana Islands | 90–94 years | 1999.77 (461.22, 8670.60) |
| Northern Mariana Islands | 95+ years | 2136.07 (111.55, 40904.19) |
| Palestine | 30–34 years | 2.34 (1.56, 3.51) |
| Palestine | 35–39 years | 5.97 (4.47, 7.96) |
| Palestine | 40–44 years | 12.97 (10.34, 16.26) |
| Palestine | 45–49 years | 27.06 (22.53, 32.49) |
| Palestine | 50–54 years | 51.09 (43.94, 59.40) |
| Palestine | 55–59 years | 92.67 (81.54, 105.31) |
| Palestine | 60–64 years | 165.15 (147.94, 184.35) |
| Palestine | 65–69 years | 312.28 (283.75, 343.68) |
| Palestine | 70–74 years | 594.75 (545.18, 648.82) |
| Palestine | 75–79 years | 1187.74 (1093.14, 1290.53) |
| Palestine | 80–84 years | 1844.24 (1660.18, 2048.70) |
| Palestine | 85–89 years | 1906.38 (1663.42, 2184.84) |
| Palestine | 90–94 years | 1610.05 (1256.41, 2063.24) |
| Palestine | 95+ years | 1527.53 (856.52, 2724.25) |
| People's Democratic Republic of Algeria | 30–34 years | 2.63 (2.35, 2.95) |
| People's Democratic Republic of Algeria | 35–39 years | 6.92 (6.39, 7.50) |
| People's Democratic Republic of Algeria | 40–44 years | 15.00 (14.10, 15.96) |
| People's Democratic Republic of Algeria | 45–49 years | 30.10 (28.62, 31.66) |
| People's Democratic Republic of Algeria | 50–54 years | 54.37 (52.14, 56.69) |
| People's Democratic Republic of Algeria | 55–59 years | 93.96 (90.69, 97.36) |
| People's Democratic Republic of Algeria | 60–64 years | 161.59 (156.74, 166.60) |
| People's Democratic Republic of Algeria | 65–69 years | 297.99 (290.21, 305.97) |
| People's Democratic Republic of Algeria | 70–74 years | 548.65 (535.67, 561.94) |
| People's Democratic Republic of Algeria | 75–79 years | 1028.20 (1005.10, 1051.83) |
| People's Democratic Republic of Algeria | 80–84 years | 1594.35 (1550.30, 1639.65) |
| People's Democratic Republic of Algeria | 85–89 years | 1917.86 (1851.05, 1987.08) |
| People's Democratic Republic of Algeria | 90–94 years | 2006.62 (1853.66, 2172.21) |
| People's Democratic Republic of Algeria | 95+ years | 2169.72 (1794.00, 2624.13) |
| People's Republic of Bangladesh | 30–34 years | 3.73 (3.54, 3.93) |
| People's Republic of Bangladesh | 35–39 years | 8.90 (8.57, 9.24) |
| People's Republic of Bangladesh | 40–44 years | 17.85 (17.33, 18.39) |
| People's Republic of Bangladesh | 45–49 years | 33.49 (32.68, 34.32) |
| People's Republic of Bangladesh | 50–54 years | 58.77 (57.58, 59.98) |
| People's Republic of Bangladesh | 55–59 years | 102.30 (100.56, 104.08) |
| People's Republic of Bangladesh | 60–64 years | 177.60 (175.01, 180.22) |
| People's Republic of Bangladesh | 65–69 years | 328.16 (324.00, 332.37) |
| People's Republic of Bangladesh | 70–74 years | 570.02 (563.50, 576.61) |
| People's Republic of Bangladesh | 75–79 years | 916.26 (906.10, 926.53) |
| People's Republic of Bangladesh | 80–84 years | 1251.42 (1232.72, 1270.40) |
| People's Republic of Bangladesh | 85–89 years | 1300.68 (1274.46, 1327.44) |
| People's Republic of Bangladesh | 90–94 years | 1155.41 (1114.43, 1197.90) |
| People's Republic of Bangladesh | 95+ years | 1028.57 (945.98, 1118.38) |
| People's Republic of China | 30–34 years | 2.13 (1.92, 2.37) |
| People's Republic of China | 35–39 years | 5.54 (5.17, 5.93) |
| People's Republic of China | 40–44 years | 16.06 (15.36, 16.80) |
| People's Republic of China | 45–49 years | 54.96 (53.35, 56.62) |
| People's Republic of China | 50–54 years | 134.47 (131.47, 137.55) |
| People's Republic of China | 55–59 years | 262.13 (257.28, 267.06) |
| People's Republic of China | 60–64 years | 465.92 (458.49, 473.46) |
| People's Republic of China | 65–69 years | 816.44 (804.80, 828.24) |
| People's Republic of China | 70–74 years | 1392.97 (1374.32, 1411.88) |
| People's Republic of China | 75–79 years | 2337.25 (2306.22, 2368.70) |
| People's Republic of China | 80–84 years | 3675.89 (3612.08, 3740.83) |
| People's Republic of China | 85–89 years | 5343.10 (5228.08, 5460.66) |
| People's Republic of China | 90–94 years | 7263.04 (7027.76, 7506.21) |
| People's Republic of China | 95+ years | 9661.08 (9046.32, 10317.62) |
| Plurinational State of Bolivia | 30–34 years | 7.06 (6.11, 8.17) |
| Plurinational State of Bolivia | 35–39 years | 21.98 (20.07, 24.07) |
| Plurinational State of Bolivia | 40–44 years | 51.06 (47.73, 54.62) |
| Plurinational State of Bolivia | 45–49 years | 107.81 (102.13, 113.82) |
| Plurinational State of Bolivia | 50–54 years | 188.73 (180.37, 197.47) |
| Plurinational State of Bolivia | 55–59 years | 262.93 (252.37, 273.93) |
| Plurinational State of Bolivia | 60–64 years | 347.44 (334.38, 361.01) |
| Plurinational State of Bolivia | 65–69 years | 537.90 (519.18, 557.31) |
| Plurinational State of Bolivia | 70–74 years | 903.36 (873.96, 933.75) |
| Plurinational State of Bolivia | 75–79 years | 1560.18 (1509.35, 1612.73) |
| Plurinational State of Bolivia | 80–84 years | 2279.48 (2182.67, 2380.59) |
| Plurinational State of Bolivia | 85–89 years | 2496.51 (2357.30, 2643.93) |
| Plurinational State of Bolivia | 90–94 years | 2420.27 (2196.79, 2666.48) |
| Plurinational State of Bolivia | 95+ years | 2728.41 (2264.59, 3287.22) |
| Portuguese Republic | 30–34 years | 5.12 (4.43, 5.90) |
| Portuguese Republic | 35–39 years | 9.63 (8.67, 10.69) |
| Portuguese Republic | 40–44 years | 16.77 (15.47, 18.18) |
| Portuguese Republic | 45–49 years | 31.73 (29.83, 33.76) |
| Portuguese Republic | 50–54 years | 57.88 (55.18, 60.73) |
| Portuguese Republic | 55–59 years | 102.17 (98.37, 106.13) |
| Portuguese Republic | 60–64 years | 186.96 (181.40, 192.69) |
| Portuguese Republic | 65–69 years | 389.95 (380.68, 399.45) |
| Portuguese Republic | 70–74 years | 746.70 (731.20, 762.52) |
| Portuguese Republic | 75–79 years | 1286.96 (1261.76, 1312.66) |
| Portuguese Republic | 80–84 years | 1882.28 (1837.90, 1927.72) |
| Portuguese Republic | 85–89 years | 2235.32 (2176.02, 2296.23) |
| Portuguese Republic | 90–94 years | 2311.50 (2229.41, 2396.61) |
| Portuguese Republic | 95+ years | 2261.09 (2115.88, 2416.25) |
| Principality of Andorra | 30–34 years | 5.47 (1.21, 24.74) |
| Principality of Andorra | 35–39 years | 10.65 (3.57, 31.80) |
| Principality of Andorra | 40–44 years | 19.27 (8.22, 45.16) |
| Principality of Andorra | 45–49 years | 38.17 (19.70, 73.93) |
| Principality of Andorra | 50–54 years | 72.09 (42.64, 121.89) |
| Principality of Andorra | 55–59 years | 131.92 (85.77, 202.90) |
| Principality of Andorra | 60–64 years | 248.67 (174.63, 354.09) |
| Principality of Andorra | 65–69 years | 529.79 (396.79, 707.38) |
| Principality of Andorra | 70–74 years | 1009.41 (779.08, 1307.84) |
| Principality of Andorra | 75–79 years | 1684.69 (1316.21, 2156.33) |
| Principality of Andorra | 80–84 years | 2412.73 (1794.82, 3243.36) |
| Principality of Andorra | 85–89 years | 2867.91 (2074.27, 3965.19) |
| Principality of Andorra | 90–94 years | 2997.44 (2008.12, 4474.17) |
| Principality of Andorra | 95+ years | 2893.38 (1564.39, 5351.40) |
| Principality of Monaco | 30–34 years | 5.44 (0.38, 78.54) |
| Principality of Monaco | 35–39 years | 10.56 (1.75, 63.61) |
| Principality of Monaco | 40–44 years | 19.19 (5.22, 70.52) |
| Principality of Monaco | 45–49 years | 38.28 (15.08, 97.21) |
| Principality of Monaco | 50–54 years | 73.00 (36.84, 144.67) |
| Principality of Monaco | 55–59 years | 136.49 (81.17, 229.52) |
| Principality of Monaco | 60–64 years | 264.03 (177.26, 393.26) |
| Principality of Monaco | 65–69 years | 579.44 (425.44, 789.18) |
| Principality of Monaco | 70–74 years | 1125.83 (863.53, 1467.82) |
| Principality of Monaco | 75–79 years | 1894.80 (1471.48, 2439.90) |
| Principality of Monaco | 80–84 years | 2711.38 (2009.23, 3658.90) |
| Principality of Monaco | 85–89 years | 3159.93 (2274.79, 4389.48) |
| Principality of Monaco | 90–94 years | 3173.92 (2108.14, 4778.52) |
| Principality of Monaco | 95+ years | 2970.01 (1608.62, 5483.55) |
| Puerto Rico | 30–34 years | 4.72 (3.64, 6.11) |
| Puerto Rico | 35–39 years | 11.90 (10.05, 14.10) |
| Puerto Rico | 40–44 years | 24.92 (22.02, 28.20) |
| Puerto Rico | 45–49 years | 48.41 (44.03, 53.22) |
| Puerto Rico | 50–54 years | 83.49 (77.46, 90.00) |
| Puerto Rico | 55–59 years | 128.55 (120.60, 137.03) |
| Puerto Rico | 60–64 years | 191.93 (181.54, 202.91) |
| Puerto Rico | 65–69 years | 306.33 (291.55, 321.86) |
| Puerto Rico | 70–74 years | 490.85 (468.72, 514.02) |
| Puerto Rico | 75–79 years | 782.95 (748.36, 819.15) |
| Puerto Rico | 80–84 years | 1087.04 (1028.92, 1148.44) |
| Puerto Rico | 85–89 years | 1213.23 (1139.30, 1291.95) |
| Puerto Rico | 90–94 years | 1273.91 (1176.95, 1378.86) |
| Puerto Rico | 95+ years | 1601.75 (1457.11, 1760.75) |
| Republic of Albania | 30–34 years | 2.92 (1.97, 4.33) |
| Republic of Albania | 35–39 years | 6.28 (4.80, 8.23) |
| Republic of Albania | 40–44 years | 12.46 (10.23, 15.17) |
| Republic of Albania | 45–49 years | 25.93 (22.42, 29.99) |
| Republic of Albania | 50–54 years | 51.83 (46.54, 57.73) |
| Republic of Albania | 55–59 years | 106.78 (98.44, 115.83) |
| Republic of Albania | 60–64 years | 214.54 (201.18, 228.79) |
| Republic of Albania | 65–69 years | 449.85 (426.67, 474.30) |
| Republic of Albania | 70–74 years | 817.21 (779.24, 857.03) |
| Republic of Albania | 75–79 years | 1265.13 (1207.36, 1325.67) |
| Republic of Albania | 80–84 years | 1581.69 (1489.69, 1679.37) |
| Republic of Albania | 85–89 years | 1405.51 (1297.44, 1522.58) |
| Republic of Albania | 90–94 years | 964.60 (832.07, 1118.24) |
| Republic of Albania | 95+ years | 630.48 (452.68, 878.10) |
| Republic of Angola | 30–34 years | 3.55 (3.00, 4.21) |
| Republic of Angola | 35–39 years | 7.83 (6.89, 8.88) |
| Republic of Angola | 40–44 years | 14.91 (13.44, 16.53) |
| Republic of Angola | 45–49 years | 27.31 (25.03, 29.80) |
| Republic of Angola | 50–54 years | 46.82 (43.52, 50.37) |
| Republic of Angola | 55–59 years | 79.24 (74.35, 84.46) |
| Republic of Angola | 60–64 years | 136.37 (128.85, 144.32) |
| Republic of Angola | 65–69 years | 258.26 (245.48, 271.72) |
| Republic of Angola | 70–74 years | 447.44 (426.40, 469.51) |
| Republic of Angola | 75–79 years | 682.89 (649.28, 718.23) |
| Republic of Angola | 80–84 years | 877.47 (818.05, 941.21) |
| Republic of Angola | 85–89 years | 924.98 (835.68, 1023.82) |
| Republic of Angola | 90–94 years | 899.96 (745.96, 1085.75) |
| Republic of Angola | 95+ years | 920.72 (607.20, 1396.12) |
| Republic of Armenia | 30–34 years | 2.66 (1.81, 3.92) |
| Republic of Armenia | 35–39 years | 5.88 (4.53, 7.63) |
| Republic of Armenia | 40–44 years | 11.67 (9.62, 14.15) |
| Republic of Armenia | 45–49 years | 23.67 (20.43, 27.44) |
| Republic of Armenia | 50–54 years | 45.40 (40.51, 50.88) |
| Republic of Armenia | 55–59 years | 87.72 (80.17, 95.98) |
| Republic of Armenia | 60–64 years | 172.02 (160.09, 184.84) |
| Republic of Armenia | 65–69 years | 365.69 (344.10, 388.62) |
| Republic of Armenia | 70–74 years | 654.01 (618.14, 691.95) |
| Republic of Armenia | 75–79 years | 952.41 (900.47, 1007.35) |
| Republic of Armenia | 80–84 years | 1136.78 (1061.99, 1216.83) |
| Republic of Armenia | 85–89 years | 1063.34 (973.52, 1161.44) |
| Republic of Armenia | 90–94 years | 847.11 (723.57, 991.75) |
| Republic of Armenia | 95+ years | 685.65 (460.52, 1020.84) |
| Republic of Austria | 30–34 years | 5.31 (4.52, 6.23) |
| Republic of Austria | 35–39 years | 10.24 (9.13, 11.49) |
| Republic of Austria | 40–44 years | 18.36 (16.83, 20.03) |
| Republic of Austria | 45–49 years | 36.00 (33.73, 38.43) |
| Republic of Austria | 50–54 years | 67.41 (64.17, 70.82) |
| Republic of Austria | 55–59 years | 122.58 (117.92, 127.43) |
| Republic of Austria | 60–64 years | 232.42 (225.39, 239.67) |
| Republic of Austria | 65–69 years | 504.12 (492.18, 516.35) |
| Republic of Austria | 70–74 years | 986.58 (966.44, 1007.13) |
| Republic of Austria | 75–79 years | 1732.72 (1699.43, 1766.66) |
| Republic of Austria | 80–84 years | 2587.41 (2527.50, 2648.75) |
| Republic of Austria | 85–89 years | 3139.46 (3058.91, 3222.13) |
| Republic of Austria | 90–94 years | 3276.14 (3168.95, 3386.96) |
| Republic of Austria | 95+ years | 2959.23 (2797.37, 3130.46) |
| Republic of Azerbaijan | 30–34 years | 2.87 (2.29, 3.59) |
| Republic of Azerbaijan | 35–39 years | 6.23 (5.32, 7.30) |
| Republic of Azerbaijan | 40–44 years | 12.07 (10.68, 13.65) |
| Republic of Azerbaijan | 45–49 years | 23.60 (21.37, 26.06) |
| Republic of Azerbaijan | 50–54 years | 43.88 (40.52, 47.53) |
| Republic of Azerbaijan | 55–59 years | 82.93 (77.79, 88.41) |
| Republic of Azerbaijan | 60–64 years | 163.24 (154.82, 172.11) |
| Republic of Azerbaijan | 65–69 years | 358.48 (342.62, 375.07) |
| Republic of Azerbaijan | 70–74 years | 680.32 (652.51, 709.32) |
| Republic of Azerbaijan | 75–79 years | 1108.79 (1064.13, 1155.33) |
| Republic of Azerbaijan | 80–84 years | 1483.01 (1409.94, 1559.86) |
| Republic of Azerbaijan | 85–89 years | 1490.44 (1398.49, 1588.44) |
| Republic of Azerbaijan | 90–94 years | 1199.64 (1087.95, 1322.79) |
| Republic of Azerbaijan | 95+ years | 728.00 (597.03, 887.70) |
| Republic of Belarus | 30–34 years | 2.73 (2.21, 3.37) |
| Republic of Belarus | 35–39 years | 6.25 (5.45, 7.16) |
| Republic of Belarus | 40–44 years | 13.00 (11.81, 14.31) |
| Republic of Belarus | 45–49 years | 28.36 (26.47, 30.40) |
| Republic of Belarus | 50–54 years | 58.65 (55.75, 61.71) |
| Republic of Belarus | 55–59 years | 124.70 (120.07, 129.51) |
| Republic of Belarus | 60–64 years | 251.74 (244.30, 259.41) |
| Republic of Belarus | 65–69 years | 515.88 (502.88, 529.22) |
| Republic of Belarus | 70–74 years | 887.18 (866.40, 908.45) |
| Republic of Belarus | 75–79 years | 1220.31 (1191.33, 1249.99) |
| Republic of Belarus | 80–84 years | 1390.45 (1350.85, 1431.21) |
| Republic of Belarus | 85–89 years | 1253.93 (1209.27, 1300.24) |
| Republic of Belarus | 90–94 years | 955.56 (901.46, 1012.91) |
| Republic of Belarus | 95+ years | 683.40 (604.46, 772.65) |
| Republic of Benin | 30–34 years | 3.49 (2.70, 4.51) |
| Republic of Benin | 35–39 years | 7.59 (6.22, 9.25) |
| Republic of Benin | 40–44 years | 13.96 (11.83, 16.48) |
| Republic of Benin | 45–49 years | 23.76 (20.60, 27.41) |
| Republic of Benin | 50–54 years | 39.17 (34.66, 44.28) |
| Republic of Benin | 55–59 years | 68.12 (61.36, 75.62) |
| Republic of Benin | 60–64 years | 123.26 (112.90, 134.57) |
| Republic of Benin | 65–69 years | 243.89 (226.48, 262.64) |
| Republic of Benin | 70–74 years | 462.31 (432.64, 494.02) |
| Republic of Benin | 75–79 years | 862.89 (810.12, 919.10) |
| Republic of Benin | 80–84 years | 1280.06 (1182.65, 1385.49) |
| Republic of Benin | 85–89 years | 1374.40 (1245.37, 1516.80) |
| Republic of Benin | 90–94 years | 1249.16 (1069.45, 1459.06) |
| Republic of Benin | 95+ years | 1118.64 (825.59, 1515.73) |
| Republic of Botswana | 30–34 years | 3.73 (2.35, 5.91) |
| Republic of Botswana | 35–39 years | 8.14 (5.71, 11.61) |
| Republic of Botswana | 40–44 years | 15.25 (11.38, 20.45) |
| Republic of Botswana | 45–49 years | 27.44 (21.39, 35.20) |
| Republic of Botswana | 50–54 years | 46.13 (37.33, 57.01) |
| Republic of Botswana | 55–59 years | 74.97 (62.36, 90.13) |
| Republic of Botswana | 60–64 years | 125.41 (106.69, 147.42) |
| Republic of Botswana | 65–69 years | 235.93 (204.84, 271.74) |
| Republic of Botswana | 70–74 years | 431.77 (378.78, 492.18) |
| Republic of Botswana | 75–79 years | 767.82 (674.50, 874.03) |
| Republic of Botswana | 80–84 years | 1115.26 (945.51, 1315.49) |
| Republic of Botswana | 85–89 years | 1246.19 (1005.64, 1544.28) |
| Republic of Botswana | 90–94 years | 1210.45 (841.86, 1740.42) |
| Republic of Botswana | 95+ years | 1145.31 (535.08, 2451.46) |
| Republic of Bulgaria | 30–34 years | 1.61 (1.17, 2.21) |
| Republic of Bulgaria | 35–39 years | 3.81 (3.08, 4.70) |
| Republic of Bulgaria | 40–44 years | 8.59 (7.43, 9.92) |
| Republic of Bulgaria | 45–49 years | 20.75 (18.85, 22.84) |
| Republic of Bulgaria | 50–54 years | 44.94 (42.04, 48.04) |
| Republic of Bulgaria | 55–59 years | 95.22 (90.77, 99.88) |
| Republic of Bulgaria | 60–64 years | 203.84 (196.81, 211.11) |
| Republic of Bulgaria | 65–69 years | 473.11 (460.56, 486.01) |
| Republic of Bulgaria | 70–74 years | 878.82 (857.41, 900.77) |
| Republic of Bulgaria | 75–79 years | 1332.18 (1299.93, 1365.23) |
| Republic of Bulgaria | 80–84 years | 1611.91 (1566.37, 1658.76) |
| Republic of Bulgaria | 85–89 years | 1442.32 (1392.41, 1494.02) |
| Republic of Bulgaria | 90–94 years | 1022.50 (952.39, 1097.78) |
| Republic of Bulgaria | 95+ years | 632.28 (487.65, 819.80) |
| Republic of Burundi | 30–34 years | 4.15 (3.23, 5.34) |
| Republic of Burundi | 35–39 years | 9.11 (7.54, 11.01) |
| Republic of Burundi | 40–44 years | 16.87 (14.42, 19.73) |
| Republic of Burundi | 45–49 years | 28.93 (25.26, 33.13) |
| Republic of Burundi | 50–54 years | 46.66 (41.54, 52.41) |
| Republic of Burundi | 55–59 years | 74.92 (67.57, 83.07) |
| Republic of Burundi | 60–64 years | 123.59 (112.65, 135.59) |
| Republic of Burundi | 65–69 years | 225.24 (207.18, 244.87) |
| Republic of Burundi | 70–74 years | 383.20 (353.94, 414.88) |
| Republic of Burundi | 75–79 years | 593.56 (547.51, 643.49) |
| Republic of Burundi | 80–84 years | 767.82 (692.26, 851.61) |
| Republic of Burundi | 85–89 years | 810.16 (705.21, 930.74) |
| Republic of Burundi | 90–94 years | 787.64 (619.75, 1001.02) |
| Republic of Burundi | 95+ years | 800.33 (484.47, 1322.14) |
| Republic of Cabo Verde | 30–34 years | 3.35 (1.36, 8.27) |
| Republic of Cabo Verde | 35–39 years | 7.70 (3.85, 15.37) |
| Republic of Cabo Verde | 40–44 years | 14.40 (8.08, 25.66) |
| Republic of Cabo Verde | 45–49 years | 23.99 (14.53, 39.60) |
| Republic of Cabo Verde | 50–54 years | 38.81 (24.99, 60.29) |
| Republic of Cabo Verde | 55–59 years | 67.66 (46.75, 97.92) |
| Republic of Cabo Verde | 60–64 years | 125.49 (93.30, 168.79) |
| Republic of Cabo Verde | 65–69 years | 260.28 (203.62, 332.72) |
| Republic of Cabo Verde | 70–74 years | 511.38 (411.32, 635.78) |
| Republic of Cabo Verde | 75–79 years | 985.12 (802.69, 1209.00) |
| Republic of Cabo Verde | 80–84 years | 1569.68 (1236.97, 1991.87) |
| Republic of Cabo Verde | 85–89 years | 1866.59 (1431.84, 2433.33) |
| Republic of Cabo Verde | 90–94 years | 1889.25 (1353.03, 2637.98) |
| Republic of Cabo Verde | 95+ years | 1898.36 (1195.72, 3013.90) |
| Republic of Cameroon | 30–34 years | 3.49 (2.95, 4.11) |
| Republic of Cameroon | 35–39 years | 7.80 (6.88, 8.85) |
| Republic of Cameroon | 40–44 years | 14.57 (13.13, 16.17) |
| Republic of Cameroon | 45–49 years | 24.79 (22.69, 27.09) |
| Republic of Cameroon | 50–54 years | 40.87 (37.93, 44.04) |
| Republic of Cameroon | 55–59 years | 71.45 (67.08, 76.10) |
| Republic of Cameroon | 60–64 years | 131.36 (124.54, 138.55) |
| Republic of Cameroon | 65–69 years | 265.04 (253.26, 277.37) |
| Republic of Cameroon | 70–74 years | 503.87 (483.30, 525.31) |
| Republic of Cameroon | 75–79 years | 936.00 (898.92, 974.62) |
| Republic of Cameroon | 80–84 years | 1378.10 (1308.01, 1451.95) |
| Republic of Cameroon | 85–89 years | 1463.17 (1365.71, 1567.58) |
| Republic of Cameroon | 90–94 years | 1299.14 (1151.66, 1465.52) |
| Republic of Cameroon | 95+ years | 1115.09 (852.91, 1457.86) |
| Republic of Chad | 30–34 years | 3.14 (2.45, 4.03) |
| Republic of Chad | 35–39 years | 6.83 (5.65, 8.26) |
| Republic of Chad | 40–44 years | 12.66 (10.84, 14.80) |
| Republic of Chad | 45–49 years | 21.73 (19.03, 24.81) |
| Republic of Chad | 50–54 years | 35.96 (32.13, 40.26) |
| Republic of Chad | 55–59 years | 62.55 (56.81, 68.88) |
| Republic of Chad | 60–64 years | 115.16 (106.18, 124.91) |
| Republic of Chad | 65–69 years | 235.93 (220.30, 252.67) |
| Republic of Chad | 70–74 years | 444.49 (417.77, 472.92) |
| Republic of Chad | 75–79 years | 777.28 (730.83, 826.68) |
| Republic of Chad | 80–84 years | 1091.99 (1010.41, 1180.17) |
| Republic of Chad | 85–89 years | 1137.54 (1028.28, 1258.42) |
| Republic of Chad | 90–94 years | 1015.57 (855.31, 1205.85) |
| Republic of Chad | 95+ years | 902.78 (632.12, 1289.32) |
| Republic of Chile | 30–34 years | 1.89 (1.58, 2.25) |
| Republic of Chile | 35–39 years | 4.19 (3.71, 4.74) |
| Republic of Chile | 40–44 years | 8.53 (7.79, 9.33) |
| Republic of Chile | 45–49 years | 18.20 (17.00, 19.49) |
| Republic of Chile | 50–54 years | 39.58 (37.60, 41.66) |
| Republic of Chile | 55–59 years | 94.37 (90.82, 98.06) |
| Republic of Chile | 60–64 years | 202.96 (196.95, 209.16) |
| Republic of Chile | 65–69 years | 412.96 (402.87, 423.31) |
| Republic of Chile | 70–74 years | 780.12 (763.35, 797.27) |
| Republic of Chile | 75–79 years | 1456.38 (1426.69, 1486.69) |
| Republic of Chile | 80–84 years | 2271.85 (2214.23, 2330.97) |
| Republic of Chile | 85–89 years | 2700.77 (2622.71, 2781.16) |
| Republic of Chile | 90–94 years | 2732.05 (2625.88, 2842.51) |
| Republic of Chile | 95+ years | 2846.77 (2667.16, 3038.49) |
| Republic of Colombia | 30–34 years | 4.39 (4.07, 4.75) |
| Republic of Colombia | 35–39 years | 11.11 (10.54, 11.71) |
| Republic of Colombia | 40–44 years | 23.42 (22.49, 24.40) |
| Republic of Colombia | 45–49 years | 46.21 (44.71, 47.77) |
| Republic of Colombia | 50–54 years | 81.57 (79.37, 83.84) |
| Republic of Colombia | 55–59 years | 130.14 (127.06, 133.29) |
| Republic of Colombia | 60–64 years | 203.18 (198.89, 207.56) |
| Republic of Colombia | 65–69 years | 343.90 (337.44, 350.49) |
| Republic of Colombia | 70–74 years | 572.91 (562.93, 583.06) |
| Republic of Colombia | 75–79 years | 908.64 (892.99, 924.57) |
| Republic of Colombia | 80–84 years | 1246.73 (1219.74, 1274.32) |
| Republic of Colombia | 85–89 years | 1410.86 (1374.98, 1447.67) |
| Republic of Colombia | 90–94 years | 1528.80 (1478.89, 1580.40) |
| Republic of Colombia | 95+ years | 2013.53 (1933.77, 2096.57) |
| Republic of Costa Rica | 30–34 years | 4.57 (3.60, 5.79) |
| Republic of Costa Rica | 35–39 years | 11.87 (10.10, 13.95) |
| Republic of Costa Rica | 40–44 years | 25.81 (22.80, 29.21) |
| Republic of Costa Rica | 45–49 years | 53.06 (48.01, 58.64) |
| Republic of Costa Rica | 50–54 years | 95.86 (88.25, 104.13) |
| Republic of Costa Rica | 55–59 years | 152.94 (142.25, 164.44) |
| Republic of Costa Rica | 60–64 years | 236.00 (221.24, 251.76) |
| Republic of Costa Rica | 65–69 years | 391.63 (369.71, 414.85) |
| Republic of Costa Rica | 70–74 years | 642.63 (609.03, 678.09) |
| Republic of Costa Rica | 75–79 years | 1024.79 (971.96, 1080.49) |
| Republic of Costa Rica | 80–84 years | 1432.25 (1341.97, 1528.60) |
| Republic of Costa Rica | 85–89 years | 1657.65 (1538.15, 1786.44) |
| Republic of Costa Rica | 90–94 years | 1804.17 (1640.65, 1983.99) |
| Republic of Costa Rica | 95+ years | 2275.10 (2017.04, 2566.16) |
| Republic of Côte d'Ivoire | 30–34 years | 3.57 (3.06, 4.17) |
| Republic of Côte d'Ivoire | 35–39 years | 7.92 (7.03, 8.93) |
| Republic of Côte d'Ivoire | 40–44 years | 14.72 (13.31, 16.29) |
| Republic of Côte d'Ivoire | 45–49 years | 24.80 (22.72, 27.07) |
| Republic of Côte d'Ivoire | 50–54 years | 40.61 (37.66, 43.78) |
| Republic of Côte d'Ivoire | 55–59 years | 70.96 (66.50, 75.72) |
| Republic of Côte d'Ivoire | 60–64 years | 130.27 (123.19, 137.75) |
| Republic of Côte d'Ivoire | 65–69 years | 262.47 (249.94, 275.64) |
| Republic of Côte d'Ivoire | 70–74 years | 501.92 (479.68, 525.19) |
| Republic of Côte d'Ivoire | 75–79 years | 934.99 (894.40, 977.43) |
| Republic of Côte d'Ivoire | 80–84 years | 1372.41 (1296.44, 1452.83) |
| Republic of Côte d'Ivoire | 85–89 years | 1446.52 (1341.83, 1559.38) |
| Republic of Côte d'Ivoire | 90–94 years | 1276.96 (1120.36, 1455.46) |
| Republic of Côte d'Ivoire | 95+ years | 1107.53 (830.97, 1476.14) |
| Republic of Croatia | 30–34 years | 2.28 (1.59, 3.27) |
| Republic of Croatia | 35–39 years | 4.96 (3.92, 6.27) |
| Republic of Croatia | 40–44 years | 10.23 (8.70, 12.02) |
| Republic of Croatia | 45–49 years | 22.97 (20.53, 25.70) |
| Republic of Croatia | 50–54 years | 48.07 (44.41, 52.04) |
| Republic of Croatia | 55–59 years | 100.87 (95.22, 106.86) |
| Republic of Croatia | 60–64 years | 205.86 (197.05, 215.07) |
| Republic of Croatia | 65–69 years | 442.82 (427.45, 458.73) |
| Republic of Croatia | 70–74 years | 807.15 (781.90, 833.22) |
| Republic of Croatia | 75–79 years | 1227.67 (1190.33, 1266.17) |
| Republic of Croatia | 80–84 years | 1537.77 (1480.98, 1596.73) |
| Republic of Croatia | 85–89 years | 1419.94 (1354.12, 1488.97) |
| Republic of Croatia | 90–94 years | 1036.02 (952.47, 1126.90) |
| Republic of Croatia | 95+ years | 718.85 (579.35, 891.94) |
| Republic of Cuba | 30–34 years | 3.78 (3.27, 4.37) |
| Republic of Cuba | 35–39 years | 9.52 (8.64, 10.49) |
| Republic of Cuba | 40–44 years | 19.97 (18.56, 21.49) |
| Republic of Cuba | 45–49 years | 39.00 (36.80, 41.33) |
| Republic of Cuba | 50–54 years | 68.27 (65.13, 71.56) |
| Republic of Cuba | 55–59 years | 108.04 (103.72, 112.54) |
| Republic of Cuba | 60–64 years | 164.65 (158.77, 170.75) |
| Republic of Cuba | 65–69 years | 259.79 (251.35, 268.51) |
| Republic of Cuba | 70–74 years | 414.35 (401.75, 427.33) |
| Republic of Cuba | 75–79 years | 679.33 (659.03, 700.26) |
| Republic of Cuba | 80–84 years | 965.41 (930.19, 1001.97) |
| Republic of Cuba | 85–89 years | 1105.56 (1059.06, 1154.11) |
| Republic of Cuba | 90–94 years | 1193.83 (1129.53, 1261.78) |
| Republic of Cuba | 95+ years | 1507.03 (1397.47, 1625.19) |
| Republic of Cyprus | 30–34 years | 6.02 (3.81, 9.52) |
| Republic of Cyprus | 35–39 years | 11.40 (8.18, 15.91) |
| Republic of Cyprus | 40–44 years | 20.38 (15.76, 26.34) |
| Republic of Cyprus | 45–49 years | 40.76 (33.63, 49.40) |
| Republic of Cyprus | 50–54 years | 77.94 (67.29, 90.26) |
| Republic of Cyprus | 55–59 years | 146.20 (130.28, 164.06) |
| Republic of Cyprus | 60–64 years | 272.91 (249.16, 298.93) |
| Republic of Cyprus | 65–69 years | 548.02 (509.22, 589.79) |
| Republic of Cyprus | 70–74 years | 1027.49 (963.82, 1095.37) |
| Republic of Cyprus | 75–79 years | 1837.55 (1730.03, 1951.76) |
| Republic of Cyprus | 80–84 years | 2739.97 (2544.78, 2950.14) |
| Republic of Cyprus | 85–89 years | 3080.47 (2819.13, 3366.03) |
| Republic of Cyprus | 90–94 years | 2867.53 (2408.88, 3413.51) |
| Republic of Cyprus | 95+ years | 2606.40 (1213.29, 5599.11) |
| Republic of Djibouti | 30–34 years | 3.60 (1.69, 7.65) |
| Republic of Djibouti | 35–39 years | 7.86 (4.42, 13.99) |
| Republic of Djibouti | 40–44 years | 14.78 (9.15, 23.86) |
| Republic of Djibouti | 45–49 years | 25.81 (17.15, 38.86) |
| Republic of Djibouti | 50–54 years | 42.59 (29.94, 60.58) |
| Republic of Djibouti | 55–59 years | 70.28 (51.53, 95.86) |
| Republic of Djibouti | 60–64 years | 119.81 (90.83, 158.05) |
| Republic of Djibouti | 65–69 years | 226.13 (176.36, 289.93) |
| Republic of Djibouti | 70–74 years | 399.38 (315.21, 506.03) |
| Republic of Djibouti | 75–79 years | 656.01 (513.81, 837.58) |
| Republic of Djibouti | 80–84 years | 888.68 (633.77, 1246.11) |
| Republic of Djibouti | 85–89 years | 935.57 (578.21, 1513.77) |
| Republic of Djibouti | 90–94 years | 891.24 (371.33, 2139.07) |
| Republic of Djibouti | 95+ years | 923.83 (146.54, 5823.99) |
| Republic of Ecuador | 30–34 years | 4.75 (4.21, 5.37) |
| Republic of Ecuador | 35–39 years | 14.54 (13.44, 15.73) |
| Republic of Ecuador | 40–44 years | 34.79 (32.80, 36.89) |
| Republic of Ecuador | 45–49 years | 76.68 (73.16, 80.36) |
| Republic of Ecuador | 50–54 years | 142.68 (137.22, 148.35) |
| Republic of Ecuador | 55–59 years | 217.17 (209.81, 224.80) |
| Republic of Ecuador | 60–64 years | 309.75 (300.10, 319.70) |
| Republic of Ecuador | 65–69 years | 483.01 (469.21, 497.23) |
| Republic of Ecuador | 70–74 years | 789.05 (767.91, 810.77) |
| Republic of Ecuador | 75–79 years | 1322.66 (1288.34, 1357.90) |
| Republic of Ecuador | 80–84 years | 1997.63 (1931.73, 2065.79) |
| Republic of Ecuador | 85–89 years | 2551.96 (2453.28, 2654.61) |
| Republic of Ecuador | 90–94 years | 2955.91 (2800.47, 3119.98) |
| Republic of Ecuador | 95+ years | 3377.48 (3029.45, 3765.50) |
| Republic of El Salvador | 30–34 years | 4.92 (3.99, 6.07) |
| Republic of El Salvador | 35–39 years | 12.30 (10.62, 14.26) |
| Republic of El Salvador | 40–44 years | 25.62 (22.85, 28.74) |
| Republic of El Salvador | 45–49 years | 49.77 (45.35, 54.63) |
| Republic of El Salvador | 50–54 years | 86.59 (80.23, 93.46) |
| Republic of El Salvador | 55–59 years | 136.33 (127.64, 145.62) |
| Republic of El Salvador | 60–64 years | 212.59 (200.63, 225.27) |
| Republic of El Salvador | 65–69 years | 366.32 (348.15, 385.43) |
| Republic of El Salvador | 70–74 years | 617.42 (588.94, 647.27) |
| Republic of El Salvador | 75–79 years | 977.71 (933.19, 1024.35) |
| Republic of El Salvador | 80–84 years | 1339.29 (1265.21, 1417.71) |
| Republic of El Salvador | 85–89 years | 1502.58 (1407.53, 1604.06) |
| Republic of El Salvador | 90–94 years | 1583.75 (1457.09, 1721.43) |
| Republic of El Salvador | 95+ years | 2018.48 (1834.23, 2221.25) |
| Republic of Equatorial Guinea | 30–34 years | 3.21 (1.43, 7.20) |
| Republic of Equatorial Guinea | 35–39 years | 7.24 (3.92, 13.39) |
| Republic of Equatorial Guinea | 40–44 years | 14.13 (8.58, 23.29) |
| Republic of Equatorial Guinea | 45–49 years | 26.88 (17.76, 40.68) |
| Republic of Equatorial Guinea | 50–54 years | 47.84 (33.98, 67.35) |
| Republic of Equatorial Guinea | 55–59 years | 83.43 (62.35, 111.64) |
| Republic of Equatorial Guinea | 60–64 years | 148.70 (115.60, 191.28) |
| Republic of Equatorial Guinea | 65–69 years | 295.84 (237.77, 368.08) |
| Republic of Equatorial Guinea | 70–74 years | 537.07 (438.36, 658.01) |
| Republic of Equatorial Guinea | 75–79 years | 865.10 (704.18, 1062.80) |
| Republic of Equatorial Guinea | 80–84 years | 1192.69 (904.30, 1573.05) |
| Republic of Equatorial Guinea | 85–89 years | 1376.11 (945.12, 2003.65) |
| Republic of Equatorial Guinea | 90–94 years | 1448.74 (755.00, 2779.93) |
| Republic of Equatorial Guinea | 95+ years | 1509.68 (376.99, 6045.67) |
| Republic of Estonia | 30–34 years | 3.26 (1.87, 5.68) |
| Republic of Estonia | 35–39 years | 7.89 (5.55, 11.23) |
| Republic of Estonia | 40–44 years | 17.11 (13.45, 21.75) |
| Republic of Estonia | 45–49 years | 38.10 (32.24, 45.03) |
| Republic of Estonia | 50–54 years | 78.94 (70.17, 88.81) |
| Republic of Estonia | 55–59 years | 166.22 (152.57, 181.10) |
| Republic of Estonia | 60–64 years | 316.86 (296.31, 338.83) |
| Republic of Estonia | 65–69 years | 577.10 (545.35, 610.71) |
| Republic of Estonia | 70–74 years | 916.44 (868.86, 966.61) |
| Republic of Estonia | 75–79 years | 1212.71 (1148.91, 1280.06) |
| Republic of Estonia | 80–84 years | 1382.11 (1294.81, 1475.30) |
| Republic of Estonia | 85–89 years | 1298.80 (1198.91, 1407.03) |
| Republic of Estonia | 90–94 years | 1053.99 (928.07, 1196.99) |
| Republic of Estonia | 95+ years | 796.50 (593.59, 1068.77) |
| Republic of Fiji | 30–34 years | 3.24 (1.66, 6.36) |
| Republic of Fiji | 35–39 years | 6.78 (4.16, 11.04) |
| Republic of Fiji | 40–44 years | 13.45 (9.27, 19.51) |
| Republic of Fiji | 45–49 years | 29.68 (22.38, 39.36) |
| Republic of Fiji | 50–54 years | 59.90 (48.05, 74.67) |
| Republic of Fiji | 55–59 years | 116.03 (96.73, 139.19) |
| Republic of Fiji | 60–64 years | 214.09 (183.02, 250.43) |
| Republic of Fiji | 65–69 years | 398.40 (345.89, 458.89) |
| Republic of Fiji | 70–74 years | 662.14 (577.15, 759.64) |
| Republic of Fiji | 75–79 years | 982.22 (849.75, 1135.34) |
| Republic of Fiji | 80–84 years | 1269.27 (1047.24, 1538.38) |
| Republic of Fiji | 85–89 years | 1384.83 (1054.11, 1819.30) |
| Republic of Fiji | 90–94 years | 1207.63 (679.98, 2144.74) |
| Republic of Fiji | 95+ years | 1199.77 (289.86, 4966.02) |
| Republic of Finland | 30–34 years | 5.45 (4.43, 6.70) |
| Republic of Finland | 35–39 years | 10.46 (9.04, 12.10) |
| Republic of Finland | 40–44 years | 18.64 (16.74, 20.76) |
| Republic of Finland | 45–49 years | 36.77 (34.02, 39.73) |
| Republic of Finland | 50–54 years | 69.95 (65.98, 74.16) |
| Republic of Finland | 55–59 years | 129.89 (124.08, 135.98) |
| Republic of Finland | 60–64 years | 249.52 (240.61, 258.76) |
| Republic of Finland | 65–69 years | 548.83 (533.15, 564.98) |
| Republic of Finland | 70–74 years | 1072.23 (1045.71, 1099.42) |
| Republic of Finland | 75–79 years | 1817.21 (1773.81, 1861.68) |
| Republic of Finland | 80–84 years | 2617.22 (2541.17, 2695.53) |
| Republic of Finland | 85–89 years | 3084.73 (2983.79, 3189.09) |
| Republic of Finland | 90–94 years | 3223.03 (3086.94, 3365.13) |
| Republic of Finland | 95+ years | 3331.82 (3119.13, 3559.02) |
| Republic of Ghana | 30–34 years | 3.45 (2.98, 4.00) |
| Republic of Ghana | 35–39 years | 7.21 (6.45, 8.07) |
| Republic of Ghana | 40–44 years | 12.96 (11.81, 14.21) |
| Republic of Ghana | 45–49 years | 21.88 (20.22, 23.68) |
| Republic of Ghana | 50–54 years | 36.22 (33.86, 38.75) |
| Republic of Ghana | 55–59 years | 64.06 (60.51, 67.82) |
| Republic of Ghana | 60–64 years | 116.02 (110.58, 121.73) |
| Republic of Ghana | 65–69 years | 223.09 (213.99, 232.57) |
| Republic of Ghana | 70–74 years | 418.83 (403.06, 435.22) |
| Republic of Ghana | 75–79 years | 805.17 (775.81, 835.65) |
| Republic of Ghana | 80–84 years | 1236.89 (1179.58, 1296.98) |
| Republic of Ghana | 85–89 years | 1348.07 (1267.53, 1433.73) |
| Republic of Ghana | 90–94 years | 1242.38 (1118.97, 1379.39) |
| Republic of Ghana | 95+ years | 1217.08 (980.86, 1510.18) |
| Republic of Guatemala | 30–34 years | 4.72 (4.00, 5.58) |
| Republic of Guatemala | 35–39 years | 11.83 (10.51, 13.32) |
| Republic of Guatemala | 40–44 years | 24.45 (22.27, 26.84) |
| Republic of Guatemala | 45–49 years | 46.45 (43.01, 50.16) |
| Republic of Guatemala | 50–54 years | 79.11 (74.18, 84.37) |
| Republic of Guatemala | 55–59 years | 121.82 (115.14, 128.90) |
| Republic of Guatemala | 60–64 years | 184.55 (175.50, 194.07) |
| Republic of Guatemala | 65–69 years | 304.96 (291.52, 319.03) |
| Republic of Guatemala | 70–74 years | 500.58 (479.86, 522.20) |
| Republic of Guatemala | 75–79 years | 792.67 (760.20, 826.52) |
| Republic of Guatemala | 80–84 years | 1112.77 (1056.05, 1172.54) |
| Republic of Guatemala | 85–89 years | 1373.76 (1290.47, 1462.42) |
| Republic of Guatemala | 90–94 years | 1534.40 (1385.42, 1699.40) |
| Republic of Guatemala | 95+ years | 1701.74 (1193.99, 2425.42) |
| Republic of Guinea - Bissau | 30–34 years | 4.06 (2.26, 7.29) |
| Republic of Guinea - Bissau | 35–39 years | 8.98 (5.72, 14.09) |
| Republic of Guinea - Bissau | 40–44 years | 16.54 (11.37, 24.06) |
| Republic of Guinea - Bissau | 45–49 years | 27.50 (19.90, 38.01) |
| Republic of Guinea - Bissau | 50–54 years | 44.03 (33.42, 58.02) |
| Republic of Guinea - Bissau | 55–59 years | 74.43 (58.70, 94.39) |
| Republic of Guinea - Bissau | 60–64 years | 133.09 (108.46, 163.32) |
| Republic of Guinea - Bissau | 65–69 years | 263.95 (220.69, 315.70) |
| Republic of Guinea - Bissau | 70–74 years | 479.07 (404.16, 567.86) |
| Republic of Guinea - Bissau | 75–79 years | 827.26 (695.29, 984.28) |
| Republic of Guinea - Bissau | 80–84 years | 1163.01 (928.62, 1456.57) |
| Republic of Guinea - Bissau | 85–89 years | 1207.16 (879.71, 1656.50) |
| Republic of Guinea - Bissau | 90–94 years | 1067.92 (583.78, 1953.57) |
| Republic of Guinea - Bissau | 95+ years | 915.44 (222.31, 3769.64) |
| Republic of Guinea | 30–34 years | 3.20 (2.50, 4.11) |
| Republic of Guinea | 35–39 years | 7.03 (5.83, 8.47) |
| Republic of Guinea | 40–44 years | 13.07 (11.23, 15.22) |
| Republic of Guinea | 45–49 years | 22.70 (19.99, 25.77) |
| Republic of Guinea | 50–54 years | 37.94 (34.14, 42.17) |
| Republic of Guinea | 55–59 years | 66.42 (60.80, 72.55) |
| Republic of Guinea | 60–64 years | 122.13 (113.49, 131.42) |
| Republic of Guinea | 65–69 years | 248.41 (233.60, 264.15) |
| Republic of Guinea | 70–74 years | 472.80 (446.95, 500.15) |
| Republic of Guinea | 75–79 years | 868.69 (822.11, 917.90) |
| Republic of Guinea | 80–84 years | 1258.12 (1177.84, 1343.88) |
| Republic of Guinea | 85–89 years | 1301.53 (1199.87, 1411.79) |
| Republic of Guinea | 90–94 years | 1135.00 (994.71, 1295.07) |
| Republic of Guinea | 95+ years | 1003.93 (758.76, 1328.30) |
| Republic of Guyana | 30–34 years | 4.99 (2.79, 8.92) |
| Republic of Guyana | 35–39 years | 12.24 (8.10, 18.50) |
| Republic of Guyana | 40–44 years | 25.03 (18.04, 34.72) |
| Republic of Guyana | 45–49 years | 47.54 (36.08, 62.65) |
| Republic of Guyana | 50–54 years | 80.23 (63.51, 101.35) |
| Republic of Guyana | 55–59 years | 120.52 (97.36, 149.19) |
| Republic of Guyana | 60–64 years | 174.29 (142.86, 212.63) |
| Republic of Guyana | 65–69 years | 265.00 (219.43, 320.02) |
| Republic of Guyana | 70–74 years | 397.74 (329.47, 480.17) |
| Republic of Guyana | 75–79 years | 575.73 (474.06, 699.19) |
| Republic of Guyana | 80–84 years | 728.84 (571.44, 929.58) |
| Republic of Guyana | 85–89 years | 806.73 (596.53, 1091.00) |
| Republic of Guyana | 90–94 years | 837.80 (534.90, 1312.23) |
| Republic of Guyana | 95+ years | 862.54 (325.69, 2284.28) |
| Republic of Haiti | 30–34 years | 5.33 (4.45, 6.39) |
| Republic of Haiti | 35–39 years | 12.86 (11.28, 14.67) |
| Republic of Haiti | 40–44 years | 25.71 (23.14, 28.57) |
| Republic of Haiti | 45–49 years | 47.23 (43.20, 51.63) |
| Republic of Haiti | 50–54 years | 77.72 (72.06, 83.82) |
| Republic of Haiti | 55–59 years | 116.09 (108.49, 124.23) |
| Republic of Haiti | 60–64 years | 168.18 (158.05, 178.95) |
| Republic of Haiti | 65–69 years | 257.71 (243.11, 273.18) |
| Republic of Haiti | 70–74 years | 388.74 (366.70, 412.10) |
| Republic of Haiti | 75–79 years | 566.37 (532.57, 602.32) |
| Republic of Haiti | 80–84 years | 698.61 (644.53, 757.24) |
| Republic of Haiti | 85–89 years | 710.14 (633.89, 795.57) |
| Republic of Haiti | 90–94 years | 691.95 (564.25, 848.54) |
| Republic of Haiti | 95+ years | 740.47 (482.71, 1135.87) |
| Republic of Honduras | 30–34 years | 4.49 (3.64, 5.53) |
| Republic of Honduras | 35–39 years | 11.34 (9.78, 13.14) |
| Republic of Honduras | 40–44 years | 24.17 (21.54, 27.11) |
| Republic of Honduras | 45–49 years | 49.16 (44.78, 53.98) |
| Republic of Honduras | 50–54 years | 88.40 (81.80, 95.54) |
| Republic of Honduras | 55–59 years | 141.21 (132.00, 151.05) |
| Republic of Honduras | 60–64 years | 224.15 (211.20, 237.89) |
| Republic of Honduras | 65–69 years | 401.21 (380.98, 422.51) |
| Republic of Honduras | 70–74 years | 708.16 (675.66, 742.22) |
| Republic of Honduras | 75–79 years | 1190.85 (1136.70, 1247.59) |
| Republic of Honduras | 80–84 years | 1696.01 (1596.56, 1801.65) |
| Republic of Honduras | 85–89 years | 2016.68 (1867.31, 2178.00) |
| Republic of Honduras | 90–94 years | 2162.47 (1902.24, 2458.30) |
| Republic of Honduras | 95+ years | 2250.86 (1699.18, 2981.67) |
| Republic of Iceland | 30–34 years | 5.58 (2.44, 12.75) |
| Republic of Iceland | 35–39 years | 10.85 (5.97, 19.71) |
| Republic of Iceland | 40–44 years | 19.64 (12.45, 30.99) |
| Republic of Iceland | 45–49 years | 39.21 (27.78, 55.34) |
| Republic of Iceland | 50–54 years | 75.20 (57.55, 98.27) |
| Republic of Iceland | 55–59 years | 141.37 (114.19, 175.02) |
| Republic of Iceland | 60–64 years | 277.63 (234.31, 328.96) |
| Republic of Iceland | 65–69 years | 623.76 (545.20, 713.64) |
| Republic of Iceland | 70–74 years | 1227.90 (1092.11, 1380.58) |
| Republic of Iceland | 75–79 years | 2107.34 (1884.93, 2356.00) |
| Republic of Iceland | 80–84 years | 3053.32 (2672.53, 3488.37) |
| Republic of Iceland | 85–89 years | 3523.88 (3039.54, 4085.40) |
| Republic of Iceland | 90–94 years | 3500.59 (2896.91, 4230.07) |
| Republic of Iceland | 95+ years | 3325.00 (2519.06, 4388.80) |
| Republic of India | 30–34 years | 3.77 (3.66, 3.88) |
| Republic of India | 35–39 years | 9.33 (9.14, 9.52) |
| Republic of India | 40–44 years | 19.29 (18.98, 19.60) |
| Republic of India | 45–49 years | 36.74 (36.25, 37.24) |
| Republic of India | 50–54 years | 64.52 (63.80, 65.25) |
| Republic of India | 55–59 years | 109.54 (108.50, 110.60) |
| Republic of India | 60–64 years | 188.15 (186.63, 189.69) |
| Republic of India | 65–69 years | 353.28 (350.78, 355.79) |
| Republic of India | 70–74 years | 621.23 (617.12, 625.37) |
| Republic of India | 75–79 years | 989.08 (982.47, 995.73) |
| Republic of India | 80–84 years | 1319.62 (1307.91, 1331.44) |
| Republic of India | 85–89 years | 1346.22 (1330.02, 1362.62) |
| Republic of India | 90–94 years | 1207.25 (1182.08, 1232.97) |
| Republic of India | 95+ years | 1183.11 (1132.18, 1236.34) |
| Republic of Indonesia | 30–34 years | 3.65 (3.52, 3.80) |
| Republic of Indonesia | 35–39 years | 7.94 (7.73, 8.17) |
| Republic of Indonesia | 40–44 years | 14.96 (14.63, 15.29) |
| Republic of Indonesia | 45–49 years | 27.67 (27.17, 28.18) |
| Republic of Indonesia | 50–54 years | 49.18 (48.45, 49.92) |
| Republic of Indonesia | 55–59 years | 89.12 (88.00, 90.26) |
| Republic of Indonesia | 60–64 years | 162.30 (160.54, 164.07) |
| Republic of Indonesia | 65–69 years | 314.66 (311.67, 317.68) |
| Republic of Indonesia | 70–74 years | 557.79 (552.78, 562.85) |
| Republic of Indonesia | 75–79 years | 877.58 (869.48, 885.75) |
| Republic of Indonesia | 80–84 years | 1160.65 (1146.64, 1174.83) |
| Republic of Indonesia | 85–89 years | 1212.07 (1192.27, 1232.20) |
| Republic of Indonesia | 90–94 years | 1195.25 (1162.20, 1229.24) |
| Republic of Indonesia | 95+ years | 1480.58 (1405.86, 1559.28) |
| Republic of Iraq | 30–34 years | 2.36 (2.08, 2.69) |
| Republic of Iraq | 35–39 years | 6.10 (5.57, 6.69) |
| Republic of Iraq | 40–44 years | 13.18 (12.26, 14.17) |
| Republic of Iraq | 45–49 years | 26.79 (25.24, 28.43) |
| Republic of Iraq | 50–54 years | 49.12 (46.74, 51.63) |
| Republic of Iraq | 55–59 years | 86.28 (82.66, 90.07) |
| Republic of Iraq | 60–64 years | 153.10 (147.46, 158.95) |
| Republic of Iraq | 65–69 years | 296.91 (287.36, 306.77) |
| Republic of Iraq | 70–74 years | 575.93 (559.15, 593.21) |
| Republic of Iraq | 75–79 years | 1144.92 (1112.96, 1177.81) |
| Republic of Iraq | 80–84 years | 1833.00 (1767.37, 1901.07) |
| Republic of Iraq | 85–89 years | 2065.81 (1975.61, 2160.14) |
| Republic of Iraq | 90–94 years | 1978.27 (1852.06, 2113.08) |
| Republic of Iraq | 95+ years | 2155.57 (1935.13, 2401.12) |
| Republic of Italy | 30–34 years | 7.49 (5.31, 10.56) |
| Republic of Italy | 35–39 years | 14.70 (11.51, 18.78) |
| Republic of Italy | 40–44 years | 25.67 (21.27, 30.99) |
| Republic of Italy | 45–49 years | 45.90 (39.73, 53.03) |
| Republic of Italy | 50–54 years | 77.13 (69.02, 86.18) |
| Republic of Italy | 55–59 years | 124.79 (114.25, 136.29) |
| Republic of Italy | 60–64 years | 221.07 (206.53, 236.63) |
| Republic of Italy | 65–69 years | 476.25 (451.93, 501.88) |
| Republic of Italy | 70–74 years | 854.13 (815.09, 895.03) |
| Republic of Italy | 75–79 years | 1271.99 (1216.24, 1330.29) |
| Republic of Italy | 80–84 years | 1576.23 (1497.74, 1658.84) |
| Republic of Italy | 85–89 years | 1563.87 (1478.75, 1653.89) |
| Republic of Italy | 90–94 years | 1377.72 (1283.83, 1478.47) |
| Republic of Italy | 95+ years | 1220.23 (1084.96, 1372.37) |
| Republic of Kazakhstan | 30–34 years | 3.07 (2.63, 3.59) |
| Republic of Kazakhstan | 35–39 years | 6.81 (6.11, 7.58) |
| Republic of Kazakhstan | 40–44 years | 13.35 (12.30, 14.48) |
| Republic of Kazakhstan | 45–49 years | 26.01 (24.38, 27.75) |
| Republic of Kazakhstan | 50–54 years | 48.31 (45.90, 50.85) |
| Republic of Kazakhstan | 55–59 years | 91.76 (88.06, 95.62) |
| Republic of Kazakhstan | 60–64 years | 177.92 (171.92, 184.13) |
| Republic of Kazakhstan | 65–69 years | 376.84 (365.93, 388.08) |
| Republic of Kazakhstan | 70–74 years | 701.48 (682.64, 720.83) |
| Republic of Kazakhstan | 75–79 years | 1120.98 (1091.36, 1151.41) |
| Republic of Kazakhstan | 80–84 years | 1455.48 (1406.68, 1505.96) |
| Republic of Kazakhstan | 85–89 years | 1386.28 (1323.91, 1451.59) |
| Republic of Kazakhstan | 90–94 years | 1065.82 (980.70, 1158.34) |
| Republic of Kazakhstan | 95+ years | 793.79 (637.86, 987.83) |
| Republic of Kenya | 30–34 years | 3.79 (3.38, 4.25) |
| Republic of Kenya | 35–39 years | 8.42 (7.70, 9.21) |
| Republic of Kenya | 40–44 years | 15.85 (14.72, 17.08) |
| Republic of Kenya | 45–49 years | 27.80 (26.09, 29.63) |
| Republic of Kenya | 50–54 years | 45.65 (43.24, 48.20) |
| Republic of Kenya | 55–59 years | 73.68 (70.25, 77.28) |
| Republic of Kenya | 60–64 years | 123.77 (118.72, 129.03) |
| Republic of Kenya | 65–69 years | 236.67 (228.23, 245.43) |
| Republic of Kenya | 70–74 years | 417.98 (403.96, 432.49) |
| Republic of Kenya | 75–79 years | 669.28 (646.50, 692.86) |
| Republic of Kenya | 80–84 years | 891.26 (852.20, 932.12) |
| Republic of Kenya | 85–89 years | 939.69 (886.30, 996.29) |
| Republic of Kenya | 90–94 years | 893.49 (813.03, 981.91) |
| Republic of Kenya | 95+ years | 907.25 (762.93, 1078.88) |
| Republic of Kiribati | 30–34 years | 3.61 (0.45, 28.81) |
| Republic of Kiribati | 35–39 years | 7.28 (1.55, 34.10) |
| Republic of Kiribati | 40–44 years | 14.33 (4.27, 48.06) |
| Republic of Kiribati | 45–49 years | 32.40 (12.82, 81.84) |
| Republic of Kiribati | 50–54 years | 64.73 (31.01, 135.12) |
| Republic of Kiribati | 55–59 years | 120.54 (64.94, 223.73) |
| Republic of Kiribati | 60–64 years | 212.05 (123.01, 365.53) |
| Republic of Kiribati | 65–69 years | 370.09 (224.31, 610.63) |
| Republic of Kiribati | 70–74 years | 599.65 (368.12, 976.79) |
| Republic of Kiribati | 75–79 years | 938.37 (567.47, 1551.69) |
| Republic of Kiribati | 80–84 years | 1226.22 (634.74, 2368.87) |
| Republic of Kiribati | 85–89 years | 1212.88 (465.31, 3161.55) |
| Republic of Kiribati | 90–94 years | 1092.18 (160.19, 7446.75) |
| Republic of Kiribati | 95+ years | 1145.80 (12.94, 101478.74) |
| Republic of Korea | 30–34 years | 2.21 (2.03, 2.42) |
| Republic of Korea | 35–39 years | 5.08 (4.79, 5.39) |
| Republic of Korea | 40–44 years | 10.33 (9.89, 10.80) |
| Republic of Korea | 45–49 years | 20.99 (20.29, 21.72) |
| Republic of Korea | 50–54 years | 42.21 (41.10, 43.35) |
| Republic of Korea | 55–59 years | 91.35 (89.47, 93.26) |
| Republic of Korea | 60–64 years | 183.61 (180.54, 186.73) |
| Republic of Korea | 65–69 years | 350.94 (345.95, 356.01) |
| Republic of Korea | 70–74 years | 613.65 (605.75, 621.64) |
| Republic of Korea | 75–79 years | 995.81 (983.33, 1008.44) |
| Republic of Korea | 80–84 years | 1377.17 (1354.46, 1400.26) |
| Republic of Korea | 85–89 years | 1508.31 (1476.93, 1540.36) |
| Republic of Korea | 90–94 years | 1404.43 (1357.63, 1452.85) |
| Republic of Korea | 95+ years | 1197.67 (1112.19, 1289.73) |
| Republic of Latvia | 30–34 years | 3.19 (2.04, 4.98) |
| Republic of Latvia | 35–39 years | 7.37 (5.54, 9.81) |
| Republic of Latvia | 40–44 years | 15.29 (12.53, 18.66) |
| Republic of Latvia | 45–49 years | 32.66 (28.38, 37.59) |
| Republic of Latvia | 50–54 years | 65.00 (58.83, 71.82) |
| Republic of Latvia | 55–59 years | 130.43 (121.20, 140.37) |
| Republic of Latvia | 60–64 years | 245.91 (232.07, 260.58) |
| Republic of Latvia | 65–69 years | 464.02 (441.94, 487.21) |
| Republic of Latvia | 70–74 years | 766.83 (732.94, 802.29) |
| Republic of Latvia | 75–79 years | 1066.96 (1020.29, 1115.77) |
| Republic of Latvia | 80–84 years | 1245.85 (1179.44, 1316.00) |
| Republic of Latvia | 85–89 years | 1153.52 (1078.04, 1234.28) |
| Republic of Latvia | 90–94 years | 901.79 (810.90, 1002.85) |
| Republic of Latvia | 95+ years | 656.34 (527.80, 816.19) |
| Republic of Liberia | 30–34 years | 3.39 (2.28, 5.04) |
| Republic of Liberia | 35–39 years | 7.42 (5.54, 9.95) |
| Republic of Liberia | 40–44 years | 13.82 (10.86, 17.59) |
| Republic of Liberia | 45–49 years | 23.61 (19.09, 29.19) |
| Republic of Liberia | 50–54 years | 38.79 (32.29, 46.60) |
| Republic of Liberia | 55–59 years | 67.50 (57.69, 78.96) |
| Republic of Liberia | 60–64 years | 122.36 (107.14, 139.76) |
| Republic of Liberia | 65–69 years | 242.43 (216.48, 271.50) |
| Republic of Liberia | 70–74 years | 453.52 (408.54, 503.45) |
| Republic of Liberia | 75–79 years | 816.37 (737.02, 904.26) |
| Republic of Liberia | 80–84 years | 1183.97 (1046.00, 1340.14) |
| Republic of Liberia | 85–89 years | 1270.22 (1090.02, 1480.21) |
| Republic of Liberia | 90–94 years | 1172.20 (919.32, 1494.64) |
| Republic of Liberia | 95+ years | 1113.25 (681.17, 1819.40) |
| Republic of Lithuania | 30–34 years | 2.95 (2.06, 4.24) |
| Republic of Lithuania | 35–39 years | 6.88 (5.44, 8.69) |
| Republic of Lithuania | 40–44 years | 14.36 (12.17, 16.93) |
| Republic of Lithuania | 45–49 years | 30.84 (27.41, 34.70) |
| Republic of Lithuania | 50–54 years | 62.14 (57.05, 67.69) |
| Republic of Lithuania | 55–59 years | 127.03 (119.18, 135.40) |
| Republic of Lithuania | 60–64 years | 243.24 (231.29, 255.81) |
| Republic of Lithuania | 65–69 years | 464.18 (444.99, 484.19) |
| Republic of Lithuania | 70–74 years | 758.60 (729.51, 788.85) |
| Republic of Lithuania | 75–79 years | 1005.80 (966.99, 1046.17) |
| Republic of Lithuania | 80–84 years | 1134.46 (1081.51, 1190.02) |
| Republic of Lithuania | 85–89 years | 1063.46 (1002.55, 1128.06) |
| Republic of Lithuania | 90–94 years | 877.78 (799.41, 963.83) |
| Republic of Lithuania | 95+ years | 709.09 (584.37, 860.44) |
| Republic of Madagascar | 30–34 years | 3.60 (3.05, 4.26) |
| Republic of Madagascar | 35–39 years | 7.85 (6.91, 8.92) |
| Republic of Madagascar | 40–44 years | 14.50 (13.02, 16.14) |
| Republic of Madagascar | 45–49 years | 24.99 (22.78, 27.42) |
| Republic of Madagascar | 50–54 years | 40.63 (37.52, 43.99) |
| Republic of Madagascar | 55–59 years | 65.90 (61.42, 70.71) |
| Republic of Madagascar | 60–64 years | 110.23 (103.47, 117.44) |
| Republic of Madagascar | 65–69 years | 204.17 (192.76, 216.25) |
| Republic of Madagascar | 70–74 years | 352.08 (333.05, 372.19) |
| Republic of Madagascar | 75–79 years | 551.97 (520.82, 584.99) |
| Republic of Madagascar | 80–84 years | 733.83 (680.78, 791.01) |
| Republic of Madagascar | 85–89 years | 812.58 (735.23, 898.06) |
| Republic of Madagascar | 90–94 years | 829.52 (702.27, 979.81) |
| Republic of Madagascar | 95+ years | 873.41 (629.21, 1212.39) |
| Republic of Malawi | 30–34 years | 3.93 (3.24, 4.77) |
| Republic of Malawi | 35–39 years | 8.49 (7.30, 9.89) |
| Republic of Malawi | 40–44 years | 15.69 (13.81, 17.82) |
| Republic of Malawi | 45–49 years | 27.03 (24.23, 30.16) |
| Republic of Malawi | 50–54 years | 43.94 (40.04, 48.21) |
| Republic of Malawi | 55–59 years | 71.04 (65.49, 77.06) |
| Republic of Malawi | 60–64 years | 119.14 (111.01, 127.86) |
| Republic of Malawi | 65–69 years | 224.04 (210.65, 238.28) |
| Republic of Malawi | 70–74 years | 388.53 (366.40, 411.99) |
| Republic of Malawi | 75–79 years | 610.93 (575.13, 648.95) |
| Republic of Malawi | 80–84 years | 797.20 (736.78, 862.57) |
| Republic of Malawi | 85–89 years | 835.35 (750.54, 929.74) |
| Republic of Malawi | 90–94 years | 808.56 (672.38, 972.33) |
| Republic of Malawi | 95+ years | 841.73 (577.39, 1227.07) |
| Republic of Maldives | 30–34 years | 4.59 (1.61, 13.05) |
| Republic of Maldives | 35–39 years | 9.70 (4.39, 21.41) |
| Republic of Maldives | 40–44 years | 18.10 (9.49, 34.52) |
| Republic of Maldives | 45–49 years | 33.89 (19.95, 57.58) |
| Republic of Maldives | 50–54 years | 60.63 (39.32, 93.49) |
| Republic of Maldives | 55–59 years | 110.16 (77.28, 157.04) |
| Republic of Maldives | 60–64 years | 196.82 (146.32, 264.75) |
| Republic of Maldives | 65–69 years | 364.45 (281.39, 472.04) |
| Republic of Maldives | 70–74 years | 641.56 (505.08, 814.92) |
| Republic of Maldives | 75–79 years | 1085.37 (861.37, 1367.63) |
| Republic of Maldives | 80–84 years | 1544.78 (1153.64, 2068.53) |
| Republic of Maldives | 85–89 years | 1658.74 (1145.93, 2401.03) |
| Republic of Maldives | 90–94 years | 1646.21 (910.94, 2974.94) |
| Republic of Maldives | 95+ years | 2210.56 (777.96, 6281.30) |
| Republic of Mali | 30–34 years | 3.54 (2.88, 4.36) |
| Republic of Mali | 35–39 years | 7.50 (6.41, 8.79) |
| Republic of Mali | 40–44 years | 13.57 (11.93, 15.44) |
| Republic of Mali | 45–49 years | 22.86 (20.51, 25.49) |
| Republic of Mali | 50–54 years | 37.33 (34.09, 40.88) |
| Republic of Mali | 55–59 years | 63.99 (59.29, 69.08) |
| Republic of Mali | 60–64 years | 116.26 (109.02, 123.98) |
| Republic of Mali | 65–69 years | 236.27 (223.68, 249.58) |
| Republic of Mali | 70–74 years | 441.82 (420.02, 464.76) |
| Republic of Mali | 75–79 years | 768.13 (730.16, 808.08) |
| Republic of Mali | 80–84 years | 1067.41 (998.57, 1141.00) |
| Republic of Mali | 85–89 years | 1040.51 (947.75, 1142.35) |
| Republic of Mali | 90–94 years | 884.32 (742.24, 1053.59) |
| Republic of Mali | 95+ years | 955.32 (675.29, 1351.49) |
| Republic of Malta | 30–34 years | 5.41 (2.61, 11.24) |
| Republic of Malta | 35–39 years | 10.35 (6.15, 17.44) |
| Republic of Malta | 40–44 years | 18.36 (12.41, 27.17) |
| Republic of Malta | 45–49 years | 35.74 (26.83, 47.62) |
| Republic of Malta | 50–54 years | 67.14 (53.96, 83.53) |
| Republic of Malta | 55–59 years | 123.72 (104.29, 146.79) |
| Republic of Malta | 60–64 years | 243.37 (212.78, 278.35) |
| Republic of Malta | 65–69 years | 561.48 (505.38, 623.80) |
| Republic of Malta | 70–74 years | 1119.42 (1021.66, 1226.52) |
| Republic of Malta | 75–79 years | 1908.99 (1748.56, 2084.14) |
| Republic of Malta | 80–84 years | 2719.97 (2439.04, 3033.25) |
| Republic of Malta | 85–89 years | 3062.05 (2698.55, 3474.51) |
| Republic of Malta | 90–94 years | 2976.60 (2505.81, 3535.86) |
| Republic of Malta | 95+ years | 2895.99 (2253.08, 3722.35) |
| Republic of Mauritius | 30–34 years | 4.53 (2.87, 7.17) |
| Republic of Mauritius | 35–39 years | 9.42 (6.79, 13.06) |
| Republic of Mauritius | 40–44 years | 17.63 (13.71, 22.66) |
| Republic of Mauritius | 45–49 years | 34.01 (27.89, 41.48) |
| Republic of Mauritius | 50–54 years | 62.25 (53.00, 73.10) |
| Republic of Mauritius | 55–59 years | 113.51 (99.22, 129.87) |
| Republic of Mauritius | 60–64 years | 201.04 (179.04, 225.73) |
| Republic of Mauritius | 65–69 years | 362.21 (326.72, 401.57) |
| Republic of Mauritius | 70–74 years | 602.39 (546.81, 663.62) |
| Republic of Mauritius | 75–79 years | 903.31 (819.98, 995.12) |
| Republic of Mauritius | 80–84 years | 1162.52 (1028.64, 1313.82) |
| Republic of Mauritius | 85–89 years | 1221.46 (1053.52, 1416.17) |
| Republic of Mauritius | 90–94 years | 1253.10 (1019.41, 1540.38) |
| Republic of Mauritius | 95+ years | 1696.06 (1281.56, 2244.61) |
| Republic of Moldova | 30–34 years | 3.37 (2.46, 4.60) |
| Republic of Moldova | 35–39 years | 7.61 (6.19, 9.35) |
| Republic of Moldova | 40–44 years | 15.33 (13.23, 17.75) |
| Republic of Moldova | 45–49 years | 31.38 (28.09, 35.06) |
| Republic of Moldova | 50–54 years | 60.35 (55.52, 65.61) |
| Republic of Moldova | 55–59 years | 117.63 (110.28, 125.48) |
| Republic of Moldova | 60–64 years | 219.55 (208.22, 231.51) |
| Republic of Moldova | 65–69 years | 414.69 (396.13, 434.12) |
| Republic of Moldova | 70–74 years | 672.06 (643.46, 701.93) |
| Republic of Moldova | 75–79 years | 874.52 (836.41, 914.36) |
| Republic of Moldova | 80–84 years | 944.07 (893.59, 997.40) |
| Republic of Moldova | 85–89 years | 818.91 (762.70, 879.26) |
| Republic of Moldova | 90–94 years | 625.42 (557.29, 701.88) |
| Republic of Moldova | 95+ years | 494.03 (396.12, 616.14) |
| Republic of Mozambique | 30–34 years | 3.57 (3.04, 4.19) |
| Republic of Mozambique | 35–39 years | 7.87 (6.96, 8.90) |
| Republic of Mozambique | 40–44 years | 14.75 (13.32, 16.32) |
| Republic of Mozambique | 45–49 years | 25.50 (23.38, 27.81) |
| Republic of Mozambique | 50–54 years | 41.39 (38.46, 44.54) |
| Republic of Mozambique | 55–59 years | 67.60 (63.37, 72.10) |
| Republic of Mozambique | 60–64 years | 113.54 (107.22, 120.23) |
| Republic of Mozambique | 65–69 years | 207.28 (196.92, 218.17) |
| Republic of Mozambique | 70–74 years | 359.31 (341.95, 377.55) |
| Republic of Mozambique | 75–79 years | 596.26 (566.74, 627.31) |
| Republic of Mozambique | 80–84 years | 802.64 (752.35, 856.31) |
| Republic of Mozambique | 85–89 years | 833.56 (764.17, 909.25) |
| Republic of Mozambique | 90–94 years | 784.84 (678.04, 908.46) |
| Republic of Mozambique | 95+ years | 789.91 (593.09, 1052.06) |
| Republic of Namibia | 30–34 years | 3.66 (2.30, 5.84) |
| Republic of Namibia | 35–39 years | 7.96 (5.56, 11.39) |
| Republic of Namibia | 40–44 years | 15.01 (11.18, 20.14) |
| Republic of Namibia | 45–49 years | 27.36 (21.36, 35.04) |
| Republic of Namibia | 50–54 years | 46.22 (37.57, 56.86) |
| Republic of Namibia | 55–59 years | 74.76 (62.48, 89.44) |
| Republic of Namibia | 60–64 years | 124.80 (106.82, 145.81) |
| Republic of Namibia | 65–69 years | 237.41 (207.60, 271.49) |
| Republic of Namibia | 70–74 years | 430.15 (379.90, 487.05) |
| Republic of Namibia | 75–79 years | 732.37 (646.57, 829.55) |
| Republic of Namibia | 80–84 years | 1037.75 (883.94, 1218.33) |
| Republic of Namibia | 85–89 years | 1172.89 (946.36, 1453.64) |
| Republic of Namibia | 90–94 years | 1164.01 (790.85, 1713.24) |
| Republic of Namibia | 95+ years | 1128.78 (463.80, 2747.15) |
| Republic of Nauru | 30–34 years | 3.40 (0.01, 1282.74) |
| Republic of Nauru | 35–39 years | 7.04 (0.09, 571.76) |
| Republic of Nauru | 40–44 years | 14.18 (0.45, 446.61) |
| Republic of Nauru | 45–49 years | 32.76 (2.26, 475.65) |
| Republic of Nauru | 50–54 years | 66.79 (7.74, 576.66) |
| Republic of Nauru | 55–59 years | 125.59 (19.18, 822.25) |
| Republic of Nauru | 60–64 years | 225.81 (43.04, 1184.65) |
| Republic of Nauru | 65–69 years | 413.65 (91.14, 1877.50) |
| Republic of Nauru | 70–74 years | 686.01 (156.22, 3012.47) |
| Republic of Nauru | 75–79 years | 1043.04 (218.43, 4980.67) |
| Republic of Nauru | 80–84 years | 1370.26 (179.92, 10435.74) |
| Republic of Nauru | 85–89 years | 1537.93 (87.39, 27064.79) |
| Republic of Nauru | 90–94 years | 1661.01 (7.88, 350094.13) |
| Republic of Nauru | 95+ years | 1801.43 (0.01, 430932034.99) |
| Republic of Nicaragua | 30–34 years | 4.66 (3.70, 5.88) |
| Republic of Nicaragua | 35–39 years | 11.72 (9.93, 13.84) |
| Republic of Nicaragua | 40–44 years | 24.79 (21.76, 28.24) |
| Republic of Nicaragua | 45–49 years | 49.59 (44.57, 55.18) |
| Republic of Nicaragua | 50–54 years | 87.89 (80.39, 96.10) |
| Republic of Nicaragua | 55–59 years | 139.11 (128.67, 150.40) |
| Republic of Nicaragua | 60–64 years | 220.88 (206.12, 236.70) |
| Republic of Nicaragua | 65–69 years | 401.40 (377.77, 426.50) |
| Republic of Nicaragua | 70–74 years | 712.75 (674.34, 753.35) |
| Republic of Nicaragua | 75–79 years | 1189.93 (1127.57, 1255.73) |
| Republic of Nicaragua | 80–84 years | 1637.04 (1530.55, 1750.94) |
| Republic of Nicaragua | 85–89 years | 1688.60 (1557.12, 1831.19) |
| Republic of Nicaragua | 90–94 years | 1597.42 (1418.95, 1798.33) |
| Republic of Nicaragua | 95+ years | 1987.68 (1625.52, 2430.53) |
| Republic of Niue | 30–34 years | 3.66 (0.00, 6436702.85) |
| Republic of Niue | 35–39 years | 7.57 (0.00, 176457.81) |
| Republic of Niue | 40–44 years | 14.99 (0.01, 20673.35) |
| Republic of Niue | 45–49 years | 33.06 (0.19, 5677.06) |
| Republic of Niue | 50–54 years | 66.68 (1.54, 2886.76) |
| Republic of Niue | 55–59 years | 128.18 (6.55, 2506.90) |
| Republic of Niue | 60–64 years | 235.68 (20.05, 2770.61) |
| Republic of Niue | 65–69 years | 440.40 (48.81, 3973.63) |
| Republic of Niue | 70–74 years | 741.83 (93.80, 5866.75) |
| Republic of Niue | 75–79 years | 1134.26 (142.92, 9002.02) |
| Republic of Niue | 80–84 years | 1499.01 (129.16, 17397.74) |
| Republic of Niue | 85–89 years | 1671.50 (87.75, 31839.22) |
| Republic of Niue | 90–94 years | 1716.23 (21.92, 134385.80) |
| Republic of Niue | 95+ years | 1805.06 (0.78, 4154912.67) |
| Republic of Palau | 30–34 years | 4.18 (0.11, 159.34) |
| Republic of Palau | 35–39 years | 8.89 (0.67, 117.98) |
| Republic of Palau | 40–44 years | 17.84 (2.50, 127.13) |
| Republic of Palau | 45–49 years | 39.51 (8.72, 179.13) |
| Republic of Palau | 50–54 years | 78.29 (23.04, 265.98) |
| Republic of Palau | 55–59 years | 146.09 (51.34, 415.70) |
| Republic of Palau | 60–64 years | 261.29 (106.64, 640.21) |
| Republic of Palau | 65–69 years | 475.93 (205.32, 1103.16) |
| Republic of Palau | 70–74 years | 787.47 (349.92, 1772.13) |
| Republic of Palau | 75–79 years | 1211.17 (529.34, 2771.28) |
| Republic of Palau | 80–84 years | 1662.12 (608.21, 4542.26) |
| Republic of Palau | 85–89 years | 1981.46 (579.62, 6773.68) |
| Republic of Palau | 90–94 years | 2208.81 (297.67, 16390.05) |
| Republic of Palau | 95+ years | 2489.88 (17.04, 363894.96) |
| Republic of Panama | 30–34 years | 4.73 (3.62, 6.17) |
| Republic of Panama | 35–39 years | 12.22 (10.19, 14.66) |
| Republic of Panama | 40–44 years | 26.24 (22.81, 30.18) |
| Republic of Panama | 45–49 years | 52.76 (47.14, 59.05) |
| Republic of Panama | 50–54 years | 94.11 (85.77, 103.27) |
| Republic of Panama | 55–59 years | 150.59 (138.92, 163.24) |
| Republic of Panama | 60–64 years | 233.56 (217.47, 250.84) |
| Republic of Panama | 65–69 years | 384.59 (360.74, 410.00) |
| Republic of Panama | 70–74 years | 626.07 (590.12, 664.21) |
| Republic of Panama | 75–79 years | 995.70 (939.54, 1055.21) |
| Republic of Panama | 80–84 years | 1389.44 (1292.75, 1493.37) |
| Republic of Panama | 85–89 years | 1589.81 (1462.45, 1728.27) |
| Republic of Panama | 90–94 years | 1717.18 (1542.05, 1912.20) |
| Republic of Panama | 95+ years | 2244.37 (1967.52, 2560.18) |
| Republic of Paraguay | 30–34 years | 3.92 (3.11, 4.94) |
| Republic of Paraguay | 35–39 years | 10.44 (8.91, 12.25) |
| Republic of Paraguay | 40–44 years | 22.73 (20.11, 25.69) |
| Republic of Paraguay | 45–49 years | 45.46 (41.15, 50.22) |
| Republic of Paraguay | 50–54 years | 80.62 (74.22, 87.57) |
| Republic of Paraguay | 55–59 years | 128.06 (119.10, 137.70) |
| Republic of Paraguay | 60–64 years | 199.42 (186.88, 212.81) |
| Republic of Paraguay | 65–69 years | 340.32 (321.11, 360.69) |
| Republic of Paraguay | 70–74 years | 564.05 (534.05, 595.74) |
| Republic of Paraguay | 75–79 years | 869.62 (823.14, 918.73) |
| Republic of Paraguay | 80–84 years | 1140.73 (1065.41, 1221.38) |
| Republic of Paraguay | 85–89 years | 1168.76 (1076.20, 1269.28) |
| Republic of Paraguay | 90–94 years | 1142.94 (1021.75, 1278.51) |
| Republic of Paraguay | 95+ years | 1490.98 (1300.52, 1709.33) |
| Republic of Peru | 30–34 years | 5.26 (4.85, 5.70) |
| Republic of Peru | 35–39 years | 16.00 (15.18, 16.86) |
| Republic of Peru | 40–44 years | 38.09 (36.63, 39.62) |
| Republic of Peru | 45–49 years | 83.80 (81.21, 86.47) |
| Republic of Peru | 50–54 years | 155.09 (151.11, 159.17) |
| Republic of Peru | 55–59 years | 234.73 (229.36, 240.22) |
| Republic of Peru | 60–64 years | 334.49 (327.43, 341.69) |
| Republic of Peru | 65–69 years | 525.66 (515.47, 536.06) |
| Republic of Peru | 70–74 years | 858.67 (843.16, 874.47) |
| Republic of Peru | 75–79 years | 1413.79 (1388.77, 1439.26) |
| Republic of Peru | 80–84 years | 2066.46 (2020.76, 2113.19) |
| Republic of Peru | 85–89 years | 2479.84 (2415.82, 2545.55) |
| Republic of Peru | 90–94 years | 2734.72 (2641.45, 2831.29) |
| Republic of Peru | 95+ years | 3403.41 (3245.48, 3569.02) |
| Republic of Poland | 30–34 years | 3.88 (3.02, 4.97) |
| Republic of Poland | 35–39 years | 7.37 (6.23, 8.71) |
| Republic of Poland | 40–44 years | 13.19 (11.70, 14.87) |
| Republic of Poland | 45–49 years | 26.21 (24.03, 28.59) |
| Republic of Poland | 50–54 years | 51.02 (47.82, 54.44) |
| Republic of Poland | 55–59 years | 103.59 (98.62, 108.81) |
| Republic of Poland | 60–64 years | 210.93 (202.94, 219.23) |
| Republic of Poland | 65–69 years | 461.99 (447.64, 476.81) |
| Republic of Poland | 70–74 years | 860.31 (836.14, 885.17) |
| Republic of Poland | 75–79 years | 1315.42 (1279.17, 1352.70) |
| Republic of Poland | 80–84 years | 1641.71 (1587.31, 1697.97) |
| Republic of Poland | 85–89 years | 1544.67 (1483.17, 1608.71) |
| Republic of Poland | 90–94 years | 1121.35 (1049.64, 1197.96) |
| Republic of Poland | 95+ years | 659.50 (560.86, 775.48) |
| Republic of Rwanda | 30–34 years | 4.35 (3.46, 5.46) |
| Republic of Rwanda | 35–39 years | 9.41 (7.91, 11.20) |
| Republic of Rwanda | 40–44 years | 17.34 (15.02, 20.03) |
| Republic of Rwanda | 45–49 years | 30.03 (26.54, 33.97) |
| Republic of Rwanda | 50–54 years | 48.05 (43.25, 53.39) |
| Republic of Rwanda | 55–59 years | 74.30 (67.68, 81.57) |
| Republic of Rwanda | 60–64 years | 118.05 (108.54, 128.40) |
| Republic of Rwanda | 65–69 years | 210.51 (195.22, 226.99) |
| Republic of Rwanda | 70–74 years | 365.78 (340.64, 392.77) |
| Republic of Rwanda | 75–79 years | 616.46 (574.25, 661.77) |
| Republic of Rwanda | 80–84 years | 850.32 (775.03, 932.92) |
| Republic of Rwanda | 85–89 years | 878.39 (773.55, 997.45) |
| Republic of Rwanda | 90–94 years | 821.38 (653.64, 1032.16) |
| Republic of Rwanda | 95+ years | 906.70 (564.73, 1455.77) |
| Republic of San Marino | 30–34 years | 5.93 (0.40, 88.57) |
| Republic of San Marino | 35–39 years | 11.29 (1.63, 78.21) |
| Republic of San Marino | 40–44 years | 19.94 (4.57, 87.00) |
| Republic of San Marino | 45–49 years | 38.43 (12.66, 116.64) |
| Republic of San Marino | 50–54 years | 71.14 (30.41, 166.42) |
| Republic of San Marino | 55–59 years | 128.55 (66.11, 249.94) |
| Republic of San Marino | 60–64 years | 238.29 (140.99, 402.75) |
| Republic of San Marino | 65–69 years | 496.24 (327.26, 752.46) |
| Republic of San Marino | 70–74 years | 929.21 (648.64, 1331.13) |
| Republic of San Marino | 75–79 years | 1552.15 (1106.06, 2178.14) |
| Republic of San Marino | 80–84 years | 2239.88 (1504.20, 3335.37) |
| Republic of San Marino | 85–89 years | 2677.35 (1747.80, 4101.25) |
| Republic of San Marino | 90–94 years | 2860.57 (1747.64, 4682.22) |
| Republic of San Marino | 95+ years | 3002.32 (1631.18, 5526.03) |
| Republic of Senegal | 30–34 years | 3.27 (2.64, 4.05) |
| Republic of Senegal | 35–39 years | 7.11 (6.04, 8.38) |
| Republic of Senegal | 40–44 years | 13.14 (11.48, 15.03) |
| Republic of Senegal | 45–49 years | 22.68 (20.25, 25.41) |
| Republic of Senegal | 50–54 years | 38.15 (34.71, 41.94) |
| Republic of Senegal | 55–59 years | 67.65 (62.49, 73.23) |
| Republic of Senegal | 60–64 years | 125.50 (117.41, 134.16) |
| Republic of Senegal | 65–69 years | 255.47 (241.51, 270.23) |
| Republic of Senegal | 70–74 years | 494.70 (470.36, 520.30) |
| Republic of Senegal | 75–79 years | 941.86 (897.39, 988.53) |
| Republic of Senegal | 80–84 years | 1410.82 (1328.20, 1498.58) |
| Republic of Senegal | 85–89 years | 1517.14 (1406.97, 1635.92) |
| Republic of Senegal | 90–94 years | 1367.80 (1211.36, 1544.43) |
| Republic of Senegal | 95+ years | 1212.72 (943.19, 1559.27) |
| Republic of Serbia | 30–34 years | 2.30 (1.79, 2.95) |
| Republic of Serbia | 35–39 years | 4.97 (4.21, 5.86) |
| Republic of Serbia | 40–44 years | 10.01 (8.90, 11.25) |
| Republic of Serbia | 45–49 years | 21.57 (19.85, 23.44) |
| Republic of Serbia | 50–54 years | 44.69 (42.14, 47.40) |
| Republic of Serbia | 55–59 years | 96.18 (92.25, 100.28) |
| Republic of Serbia | 60–64 years | 193.31 (187.24, 199.57) |
| Republic of Serbia | 65–69 years | 387.04 (376.92, 397.43) |
| Republic of Serbia | 70–74 years | 704.39 (687.76, 721.41) |
| Republic of Serbia | 75–79 years | 1206.47 (1179.05, 1234.53) |
| Republic of Serbia | 80–84 years | 1700.13 (1652.35, 1749.29) |
| Republic of Serbia | 85–89 years | 1691.39 (1629.71, 1755.41) |
| Republic of Serbia | 90–94 years | 1256.37 (1178.23, 1339.69) |
| Republic of Serbia | 95+ years | 732.82 (631.04, 851.02) |
| Republic of Seychelles | 30–34 years | 4.39 (0.79, 24.46) |
| Republic of Seychelles | 35–39 years | 9.86 (2.93, 33.22) |
| Republic of Seychelles | 40–44 years | 19.12 (7.38, 49.53) |
| Republic of Seychelles | 45–49 years | 36.24 (16.75, 78.40) |
| Republic of Seychelles | 50–54 years | 66.80 (35.60, 125.31) |
| Republic of Seychelles | 55–59 years | 129.24 (77.35, 215.94) |
| Republic of Seychelles | 60–64 years | 236.39 (153.50, 364.04) |
| Republic of Seychelles | 65–69 years | 422.54 (289.51, 616.70) |
| Republic of Seychelles | 70–74 years | 714.51 (504.29, 1012.36) |
| Republic of Seychelles | 75–79 years | 1169.24 (833.32, 1640.59) |
| Republic of Seychelles | 80–84 years | 1592.96 (1053.31, 2409.10) |
| Republic of Seychelles | 85–89 years | 1677.50 (1020.92, 2756.36) |
| Republic of Seychelles | 90–94 years | 1638.42 (798.73, 3360.85) |
| Republic of Seychelles | 95+ years | 1896.46 (574.97, 6255.19) |
| Republic of Sierra Leone | 30–34 years | 2.99 (2.19, 4.07) |
| Republic of Sierra Leone | 35–39 years | 6.56 (5.18, 8.31) |
| Republic of Sierra Leone | 40–44 years | 12.25 (10.07, 14.89) |
| Republic of Sierra Leone | 45–49 years | 21.16 (17.87, 25.04) |
| Republic of Sierra Leone | 50–54 years | 35.14 (30.43, 40.57) |
| Republic of Sierra Leone | 55–59 years | 61.30 (54.24, 69.28) |
| Republic of Sierra Leone | 60–64 years | 111.19 (100.28, 123.30) |
| Republic of Sierra Leone | 65–69 years | 219.64 (201.22, 239.74) |
| Republic of Sierra Leone | 70–74 years | 414.10 (382.98, 447.74) |
| Republic of Sierra Leone | 75–79 years | 764.35 (709.57, 823.35) |
| Republic of Sierra Leone | 80–84 years | 1116.56 (1017.50, 1225.27) |
| Republic of Sierra Leone | 85–89 years | 1176.17 (1045.77, 1322.82) |
| Republic of Sierra Leone | 90–94 years | 1054.67 (870.60, 1277.67) |
| Republic of Sierra Leone | 95+ years | 954.64 (644.90, 1413.14) |
| Republic of Singapore | 30–34 years | 2.80 (2.15, 3.65) |
| Republic of Singapore | 35–39 years | 6.37 (5.28, 7.70) |
| Republic of Singapore | 40–44 years | 12.71 (11.00, 14.69) |
| Republic of Singapore | 45–49 years | 25.29 (22.57, 28.34) |
| Republic of Singapore | 50–54 years | 48.97 (44.71, 53.63) |
| Republic of Singapore | 55–59 years | 99.44 (92.36, 107.06) |
| Republic of Singapore | 60–64 years | 185.66 (174.51, 197.53) |
| Republic of Singapore | 65–69 years | 325.97 (308.77, 344.14) |
| Republic of Singapore | 70–74 years | 547.97 (521.10, 576.23) |
| Republic of Singapore | 75–79 years | 922.05 (877.97, 968.34) |
| Republic of Singapore | 80–84 years | 1326.33 (1246.66, 1411.09) |
| Republic of Singapore | 85–89 years | 1462.17 (1358.47, 1573.80) |
| Republic of Singapore | 90–94 years | 1356.05 (1224.40, 1501.85) |
| Republic of Singapore | 95+ years | 1176.36 (992.53, 1394.23) |
| Republic of Slovenia | 30–34 years | 2.59 (1.58, 4.26) |
| Republic of Slovenia | 35–39 years | 5.54 (3.98, 7.71) |
| Republic of Slovenia | 40–44 years | 11.20 (8.90, 14.10) |
| Republic of Slovenia | 45–49 years | 24.77 (21.08, 29.09) |
| Republic of Slovenia | 50–54 years | 51.40 (45.78, 57.71) |
| Republic of Slovenia | 55–59 years | 107.35 (98.57, 116.92) |
| Republic of Slovenia | 60–64 years | 217.72 (203.87, 232.50) |
| Republic of Slovenia | 65–69 years | 463.37 (439.42, 488.62) |
| Republic of Slovenia | 70–74 years | 838.75 (800.15, 879.21) |
| Republic of Slovenia | 75–79 years | 1258.76 (1202.30, 1317.88) |
| Republic of Slovenia | 80–84 years | 1555.70 (1470.62, 1645.70) |
| Republic of Slovenia | 85–89 years | 1458.04 (1363.42, 1559.22) |
| Republic of Slovenia | 90–94 years | 1106.97 (1001.45, 1223.61) |
| Republic of Slovenia | 95+ years | 805.95 (662.16, 980.96) |
| Republic of South Africa | 30–34 years | 4.29 (3.95, 4.67) |
| Republic of South Africa | 35–39 years | 8.97 (8.43, 9.56) |
| Republic of South Africa | 40–44 years | 16.27 (15.45, 17.13) |
| Republic of South Africa | 45–49 years | 28.35 (27.15, 29.61) |
| Republic of South Africa | 50–54 years | 46.53 (44.87, 48.26) |
| Republic of South Africa | 55–59 years | 73.86 (71.54, 76.25) |
| Republic of South Africa | 60–64 years | 119.62 (116.33, 123.00) |
| Republic of South Africa | 65–69 years | 217.89 (212.57, 223.35) |
| Republic of South Africa | 70–74 years | 398.25 (389.37, 407.33) |
| Republic of South Africa | 75–79 years | 735.15 (719.22, 751.43) |
| Republic of South Africa | 80–84 years | 1125.95 (1096.42, 1156.27) |
| Republic of South Africa | 85–89 years | 1312.61 (1271.60, 1354.94) |
| Republic of South Africa | 90–94 years | 1309.59 (1246.55, 1375.81) |
| Republic of South Africa | 95+ years | 1276.09 (1140.79, 1427.43) |
| Republic of South Sudan | 30–34 years | 3.97 (3.10, 5.09) |
| Republic of South Sudan | 35–39 years | 8.38 (6.91, 10.18) |
| Republic of South Sudan | 40–44 years | 15.20 (12.90, 17.92) |
| Republic of South Sudan | 45–49 years | 25.94 (22.48, 29.93) |
| Republic of South Sudan | 50–54 years | 41.97 (37.12, 47.47) |
| Republic of South Sudan | 55–59 years | 68.39 (61.32, 76.27) |
| Republic of South Sudan | 60–64 years | 115.60 (104.88, 127.41) |
| Republic of South Sudan | 65–69 years | 217.49 (199.45, 237.17) |
| Republic of South Sudan | 70–74 years | 377.18 (347.37, 409.55) |
| Republic of South Sudan | 75–79 years | 587.63 (539.92, 639.57) |
| Republic of South Sudan | 80–84 years | 766.32 (690.80, 850.08) |
| Republic of South Sudan | 85–89 years | 821.87 (721.89, 935.70) |
| Republic of South Sudan | 90–94 years | 808.90 (661.86, 988.61) |
| Republic of South Sudan | 95+ years | 830.54 (567.59, 1215.32) |
| Republic of Sudan | 30–34 years | 2.70 (2.38, 3.07) |
| Republic of Sudan | 35–39 years | 6.96 (6.35, 7.62) |
| Republic of Sudan | 40–44 years | 14.95 (13.90, 16.06) |
| Republic of Sudan | 45–49 years | 30.14 (28.41, 31.98) |
| Republic of Sudan | 50–54 years | 54.60 (51.96, 57.37) |
| Republic of Sudan | 55–59 years | 95.41 (91.42, 99.58) |
| Republic of Sudan | 60–64 years | 167.69 (161.54, 174.08) |
| Republic of Sudan | 65–69 years | 317.36 (307.10, 327.96) |
| Republic of Sudan | 70–74 years | 565.26 (548.47, 582.58) |
| Republic of Sudan | 75–79 years | 940.05 (912.40, 968.53) |
| Republic of Sudan | 80–84 years | 1323.32 (1274.63, 1373.88) |
| Republic of Sudan | 85–89 years | 1478.33 (1410.89, 1548.99) |
| Republic of Sudan | 90–94 years | 1468.54 (1366.77, 1577.87) |
| Republic of Sudan | 95+ years | 1560.76 (1369.43, 1778.83) |
| Republic of Suriname | 30–34 years | 4.14 (1.95, 8.80) |
| Republic of Suriname | 35–39 years | 10.20 (6.01, 17.30) |
| Republic of Suriname | 40–44 years | 20.86 (13.80, 31.52) |
| Republic of Suriname | 45–49 years | 39.43 (28.14, 55.27) |
| Republic of Suriname | 50–54 years | 66.93 (50.52, 88.68) |
| Republic of Suriname | 55–59 years | 103.47 (80.69, 132.68) |
| Republic of Suriname | 60–64 years | 154.61 (123.51, 193.55) |
| Republic of Suriname | 65–69 years | 240.33 (195.14, 295.99) |
| Republic of Suriname | 70–74 years | 373.40 (306.15, 455.41) |
| Republic of Suriname | 75–79 years | 585.86 (481.69, 712.57) |
| Republic of Suriname | 80–84 years | 780.83 (608.49, 1001.97) |
| Republic of Suriname | 85–89 years | 807.00 (587.58, 1108.36) |
| Republic of Suriname | 90–94 years | 792.90 (498.71, 1260.62) |
| Republic of Suriname | 95+ years | 973.26 (551.21, 1718.45) |
| Republic of Tajikistan | 30–34 years | 3.05 (2.31, 4.02) |
| Republic of Tajikistan | 35–39 years | 6.55 (5.34, 8.02) |
| Republic of Tajikistan | 40–44 years | 12.59 (10.70, 14.81) |
| Republic of Tajikistan | 45–49 years | 24.55 (21.50, 28.04) |
| Republic of Tajikistan | 50–54 years | 45.60 (40.94, 50.79) |
| Republic of Tajikistan | 55–59 years | 86.00 (78.75, 93.90) |
| Republic of Tajikistan | 60–64 years | 173.18 (161.10, 186.16) |
| Republic of Tajikistan | 65–69 years | 396.10 (372.72, 420.94) |
| Republic of Tajikistan | 70–74 years | 790.53 (748.08, 835.38) |
| Republic of Tajikistan | 75–79 years | 1401.27 (1329.07, 1477.39) |
| Republic of Tajikistan | 80–84 years | 1911.11 (1790.93, 2039.36) |
| Republic of Tajikistan | 85–89 years | 1742.33 (1603.31, 1893.41) |
| Republic of Tajikistan | 90–94 years | 1251.65 (1086.04, 1442.51) |
| Republic of Tajikistan | 95+ years | 868.71 (627.49, 1202.67) |
| Republic of the Congo | 30–34 years | 3.99 (2.86, 5.58) |
| Republic of the Congo | 35–39 years | 8.96 (6.97, 11.51) |
| Republic of the Congo | 40–44 years | 17.27 (14.08, 21.19) |
| Republic of the Congo | 45–49 years | 32.12 (27.06, 38.14) |
| Republic of the Congo | 50–54 years | 55.23 (47.81, 63.81) |
| Republic of the Congo | 55–59 years | 92.13 (81.31, 104.38) |
| Republic of the Congo | 60–64 years | 155.92 (139.71, 174.01) |
| Republic of the Congo | 65–69 years | 291.87 (264.81, 321.69) |
| Republic of the Congo | 70–74 years | 496.05 (452.53, 543.75) |
| Republic of the Congo | 75–79 years | 732.80 (666.26, 805.99) |
| Republic of the Congo | 80–84 years | 929.72 (818.30, 1056.30) |
| Republic of the Congo | 85–89 years | 1011.64 (842.50, 1214.74) |
| Republic of the Congo | 90–94 years | 1017.80 (711.17, 1456.65) |
| Republic of the Congo | 95+ years | 991.87 (402.32, 2445.33) |
| Republic of the Gambia | 30–34 years | 3.04 (1.70, 5.40) |
| Republic of the Gambia | 35–39 years | 6.72 (4.30, 10.52) |
| Republic of the Gambia | 40–44 years | 12.64 (8.70, 18.36) |
| Republic of the Gambia | 45–49 years | 22.05 (16.03, 30.33) |
| Republic of the Gambia | 50–54 years | 37.46 (28.50, 49.23) |
| Republic of the Gambia | 55–59 years | 67.47 (53.56, 84.99) |
| Republic of the Gambia | 60–64 years | 125.57 (104.02, 151.58) |
| Republic of the Gambia | 65–69 years | 252.19 (215.92, 294.55) |
| Republic of the Gambia | 70–74 years | 479.91 (417.94, 551.07) |
| Republic of the Gambia | 75–79 years | 915.29 (800.95, 1045.94) |
| Republic of the Gambia | 80–84 years | 1395.49 (1177.44, 1653.92) |
| Republic of the Gambia | 85–89 years | 1525.36 (1224.02, 1900.88) |
| Republic of the Gambia | 90–94 years | 1392.97 (954.93, 2031.96) |
| Republic of the Gambia | 95+ years | 1242.15 (542.09, 2846.28) |
| Republic of the Marshall Islands | 30–34 years | 3.59 (0.23, 56.05) |
| Republic of the Marshall Islands | 35–39 years | 7.36 (0.93, 58.33) |
| Republic of the Marshall Islands | 40–44 years | 14.68 (2.87, 75.00) |
| Republic of the Marshall Islands | 45–49 years | 33.50 (9.27, 121.03) |
| Republic of the Marshall Islands | 50–54 years | 67.83 (23.77, 193.57) |
| Republic of the Marshall Islands | 55–59 years | 127.45 (51.63, 314.62) |
| Republic of the Marshall Islands | 60–64 years | 227.40 (101.50, 509.46) |
| Republic of the Marshall Islands | 65–69 years | 408.37 (192.57, 866.00) |
| Republic of the Marshall Islands | 70–74 years | 670.40 (318.21, 1412.37) |
| Republic of the Marshall Islands | 75–79 years | 1035.84 (469.45, 2285.57) |
| Republic of the Marshall Islands | 80–84 years | 1394.82 (494.05, 3937.89) |
| Republic of the Marshall Islands | 85–89 years | 1575.92 (376.86, 6590.07) |
| Republic of the Marshall Islands | 90–94 years | 1678.22 (129.34, 21775.09) |
| Republic of the Marshall Islands | 95+ years | 1827.53 (6.10, 547584.97) |
| Republic of the Niger | 30–34 years | 3.60 (2.92, 4.43) |
| Republic of the Niger | 35–39 years | 7.63 (6.51, 8.94) |
| Republic of the Niger | 40–44 years | 13.59 (11.91, 15.52) |
| Republic of the Niger | 45–49 years | 22.50 (20.04, 25.25) |
| Republic of the Niger | 50–54 years | 36.69 (33.29, 40.43) |
| Republic of the Niger | 55–59 years | 63.32 (58.34, 68.73) |
| Republic of the Niger | 60–64 years | 114.12 (106.33, 122.47) |
| Republic of the Niger | 65–69 years | 224.78 (211.51, 238.90) |
| Republic of the Niger | 70–74 years | 418.73 (395.96, 442.80) |
| Republic of the Niger | 75–79 years | 752.66 (712.02, 795.61) |
| Republic of the Niger | 80–84 years | 1082.94 (1005.97, 1165.80) |
| Republic of the Niger | 85–89 years | 1141.16 (1033.97, 1259.47) |
| Republic of the Niger | 90–94 years | 1036.92 (874.68, 1229.25) |
| Republic of the Niger | 95+ years | 983.05 (681.36, 1418.33) |
| Republic of the Philippines | 30–34 years | 3.84 (3.60, 4.09) |
| Republic of the Philippines | 35–39 years | 8.60 (8.21, 9.01) |
| Republic of the Philippines | 40–44 years | 16.41 (15.81, 17.04) |
| Republic of the Philippines | 45–49 years | 29.84 (28.93, 30.78) |
| Republic of the Philippines | 50–54 years | 51.83 (50.51, 53.19) |
| Republic of the Philippines | 55–59 years | 91.83 (89.82, 93.89) |
| Republic of the Philippines | 60–64 years | 162.88 (159.78, 166.03) |
| Republic of the Philippines | 65–69 years | 303.82 (298.67, 309.07) |
| Republic of the Philippines | 70–74 years | 515.58 (507.31, 523.99) |
| Republic of the Philippines | 75–79 years | 772.46 (759.78, 785.35) |
| Republic of the Philippines | 80–84 years | 994.74 (973.98, 1015.94) |
| Republic of the Philippines | 85–89 years | 1092.83 (1064.38, 1122.03) |
| Republic of the Philippines | 90–94 years | 1155.08 (1110.66, 1201.29) |
| Republic of the Philippines | 95+ years | 1527.32 (1433.29, 1627.53) |
| Republic of the Union of Myanmar | 30–34 years | 3.61 (3.31, 3.92) |
| Republic of the Union of Myanmar | 35–39 years | 7.76 (7.29, 8.25) |
| Republic of the Union of Myanmar | 40–44 years | 14.63 (13.92, 15.37) |
| Republic of the Union of Myanmar | 45–49 years | 27.65 (26.55, 28.79) |
| Republic of the Union of Myanmar | 50–54 years | 49.79 (48.17, 51.47) |
| Republic of the Union of Myanmar | 55–59 years | 90.35 (87.87, 92.90) |
| Republic of the Union of Myanmar | 60–64 years | 162.57 (158.72, 166.52) |
| Republic of the Union of Myanmar | 65–69 years | 303.61 (297.21, 310.14) |
| Republic of the Union of Myanmar | 70–74 years | 524.71 (514.33, 535.30) |
| Republic of the Union of Myanmar | 75–79 years | 828.07 (811.66, 844.82) |
| Republic of the Union of Myanmar | 80–84 years | 1083.67 (1056.44, 1111.61) |
| Republic of the Union of Myanmar | 85–89 years | 1049.50 (1014.64, 1085.56) |
| Republic of the Union of Myanmar | 90–94 years | 969.34 (914.04, 1028.00) |
| Republic of the Union of Myanmar | 95+ years | 1354.67 (1213.40, 1512.38) |
| Republic of Trinidad and Tobago | 30–34 years | 4.68 (3.03, 7.22) |
| Republic of Trinidad and Tobago | 35–39 years | 11.58 (8.62, 15.56) |
| Republic of Trinidad and Tobago | 40–44 years | 23.78 (18.98, 29.80) |
| Republic of Trinidad and Tobago | 45–49 years | 45.34 (37.80, 54.38) |
| Republic of Trinidad and Tobago | 50–54 years | 76.53 (65.85, 88.95) |
| Republic of Trinidad and Tobago | 55–59 years | 114.73 (100.41, 131.10) |
| Republic of Trinidad and Tobago | 60–64 years | 166.06 (147.13, 187.44) |
| Republic of Trinidad and Tobago | 65–69 years | 254.94 (228.09, 284.94) |
| Republic of Trinidad and Tobago | 70–74 years | 394.31 (354.26, 438.89) |
| Republic of Trinidad and Tobago | 75–79 years | 610.77 (548.54, 680.06) |
| Republic of Trinidad and Tobago | 80–84 years | 811.99 (710.09, 928.52) |
| Republic of Trinidad and Tobago | 85–89 years | 858.24 (729.16, 1010.17) |
| Republic of Trinidad and Tobago | 90–94 years | 890.67 (711.47, 1114.99) |
| Republic of Trinidad and Tobago | 95+ years | 1217.72 (900.78, 1646.18) |
| Republic of Tunisia | 30–34 years | 2.16 (1.76, 2.64) |
| Republic of Tunisia | 35–39 years | 5.67 (4.95, 6.50) |
| Republic of Tunisia | 40–44 years | 12.51 (11.29, 13.86) |
| Republic of Tunisia | 45–49 years | 26.13 (24.09, 28.35) |
| Republic of Tunisia | 50–54 years | 49.37 (46.22, 52.74) |
| Republic of Tunisia | 55–59 years | 89.53 (84.71, 94.63) |
| Republic of Tunisia | 60–64 years | 160.32 (152.98, 168.02) |
| Republic of Tunisia | 65–69 years | 302.60 (290.77, 314.92) |
| Republic of Tunisia | 70–74 years | 570.95 (550.93, 591.69) |
| Republic of Tunisia | 75–79 years | 1118.41 (1081.06, 1157.06) |
| Republic of Tunisia | 80–84 years | 1757.06 (1684.62, 1832.62) |
| Republic of Tunisia | 85–89 years | 1908.76 (1813.43, 2009.09) |
| Republic of Tunisia | 90–94 years | 1758.08 (1625.33, 1901.67) |
| Republic of Tunisia | 95+ years | 1902.37 (1655.40, 2186.19) |
| Republic of Turkey | 30–34 years | 2.58 (2.40, 2.78) |
| Republic of Turkey | 35–39 years | 6.85 (6.52, 7.21) |
| Republic of Turkey | 40–44 years | 14.87 (14.32, 15.44) |
| Republic of Turkey | 45–49 years | 29.78 (28.91, 30.67) |
| Republic of Turkey | 50–54 years | 53.76 (52.48, 55.08) |
| Republic of Turkey | 55–59 years | 93.13 (91.27, 95.03) |
| Republic of Turkey | 60–64 years | 165.31 (162.49, 168.18) |
| Republic of Turkey | 65–69 years | 328.73 (323.96, 333.57) |
| Republic of Turkey | 70–74 years | 620.72 (612.61, 628.95) |
| Republic of Turkey | 75–79 years | 1104.34 (1090.45, 1118.41) |
| Republic of Turkey | 80–84 years | 1678.99 (1652.12, 1706.29) |
| Republic of Turkey | 85–89 years | 2099.51 (2059.80, 2139.98) |
| Republic of Turkey | 90–94 years | 2265.73 (2206.91, 2326.13) |
| Republic of Turkey | 95+ years | 2163.19 (2082.13, 2247.40) |
| Republic of Uganda | 30–34 years | 3.72 (3.24, 4.28) |
| Republic of Uganda | 35–39 years | 8.15 (7.30, 9.10) |
| Republic of Uganda | 40–44 years | 15.15 (13.82, 16.62) |
| Republic of Uganda | 45–49 years | 26.43 (24.41, 28.61) |
| Republic of Uganda | 50–54 years | 43.49 (40.65, 46.53) |
| Republic of Uganda | 55–59 years | 71.50 (67.44, 75.82) |
| Republic of Uganda | 60–64 years | 122.16 (116.10, 128.53) |
| Republic of Uganda | 65–69 years | 233.08 (222.98, 243.64) |
| Republic of Uganda | 70–74 years | 409.09 (392.36, 426.53) |
| Republic of Uganda | 75–79 years | 652.05 (625.09, 680.17) |
| Republic of Uganda | 80–84 years | 864.18 (817.74, 913.27) |
| Republic of Uganda | 85–89 years | 929.76 (863.88, 1000.68) |
| Republic of Uganda | 90–94 years | 913.07 (809.17, 1030.31) |
| Republic of Uganda | 95+ years | 932.84 (739.38, 1176.93) |
| Republic of Uzbekistan | 30–34 years | 3.03 (2.66, 3.46) |
| Republic of Uzbekistan | 35–39 years | 6.57 (5.97, 7.23) |
| Republic of Uzbekistan | 40–44 years | 12.66 (11.75, 13.65) |
| Republic of Uzbekistan | 45–49 years | 24.54 (23.09, 26.08) |
| Republic of Uzbekistan | 50–54 years | 45.75 (43.56, 48.05) |
| Republic of Uzbekistan | 55–59 years | 88.24 (84.80, 91.82) |
| Republic of Uzbekistan | 60–64 years | 175.83 (170.17, 181.67) |
| Republic of Uzbekistan | 65–69 years | 385.76 (375.28, 396.53) |
| Republic of Uzbekistan | 70–74 years | 717.93 (699.90, 736.43) |
| Republic of Uzbekistan | 75–79 years | 1111.25 (1083.66, 1139.55) |
| Republic of Uzbekistan | 80–84 years | 1384.19 (1340.60, 1429.20) |
| Republic of Uzbekistan | 85–89 years | 1256.09 (1204.62, 1309.75) |
| Republic of Uzbekistan | 90–94 years | 896.84 (835.01, 963.24) |
| Republic of Uzbekistan | 95+ years | 606.14 (515.76, 712.35) |
| Republic of Vanuatu | 30–34 years | 3.44 (0.87, 13.68) |
| Republic of Vanuatu | 35–39 years | 7.14 (2.54, 20.06) |
| Republic of Vanuatu | 40–44 years | 14.14 (6.25, 31.99) |
| Republic of Vanuatu | 45–49 years | 31.47 (16.66, 59.48) |
| Republic of Vanuatu | 50–54 years | 63.29 (38.14, 105.02) |
| Republic of Vanuatu | 55–59 years | 119.45 (78.29, 182.26) |
| Republic of Vanuatu | 60–64 years | 214.85 (149.99, 307.78) |
| Republic of Vanuatu | 65–69 years | 391.36 (283.98, 539.34) |
| Republic of Vanuatu | 70–74 years | 642.61 (471.68, 875.48) |
| Republic of Vanuatu | 75–79 years | 963.32 (698.42, 1328.69) |
| Republic of Vanuatu | 80–84 years | 1262.72 (821.67, 1940.51) |
| Republic of Vanuatu | 85–89 years | 1414.93 (778.77, 2570.78) |
| Republic of Vanuatu | 90–94 years | 1484.78 (494.61, 4457.20) |
| Republic of Vanuatu | 95+ years | 1603.54 (113.60, 22634.52) |
| Republic of Yemen | 30–34 years | 2.29 (1.94, 2.70) |
| Republic of Yemen | 35–39 years | 5.96 (5.28, 6.72) |
| Republic of Yemen | 40–44 years | 12.88 (11.69, 14.18) |
| Republic of Yemen | 45–49 years | 25.93 (23.94, 28.08) |
| Republic of Yemen | 50–54 years | 46.98 (43.97, 50.20) |
| Republic of Yemen | 55–59 years | 82.27 (77.74, 87.06) |
| Republic of Yemen | 60–64 years | 144.03 (137.16, 151.23) |
| Republic of Yemen | 65–69 years | 267.93 (256.74, 279.60) |
| Republic of Yemen | 70–74 years | 481.20 (462.51, 500.65) |
| Republic of Yemen | 75–79 years | 844.32 (811.55, 878.42) |
| Republic of Yemen | 80–84 years | 1214.75 (1153.42, 1279.34) |
| Republic of Yemen | 85–89 years | 1256.99 (1172.52, 1347.53) |
| Republic of Yemen | 90–94 years | 1147.43 (1016.62, 1295.09) |
| Republic of Yemen | 95+ years | 1304.28 (1031.48, 1649.22) |
| Republic of Zambia | 30–34 years | 3.85 (3.14, 4.72) |
| Republic of Zambia | 35–39 years | 8.54 (7.29, 10.01) |
| Republic of Zambia | 40–44 years | 16.08 (14.08, 18.37) |
| Republic of Zambia | 45–49 years | 28.23 (25.19, 31.64) |
| Republic of Zambia | 50–54 years | 46.44 (42.15, 51.17) |
| Republic of Zambia | 55–59 years | 75.47 (69.26, 82.23) |
| Republic of Zambia | 60–64 years | 125.78 (116.59, 135.70) |
| Republic of Zambia | 65–69 years | 233.25 (218.14, 249.41) |
| Republic of Zambia | 70–74 years | 406.08 (381.64, 432.10) |
| Republic of Zambia | 75–79 years | 645.36 (606.40, 686.83) |
| Republic of Zambia | 80–84 years | 853.02 (785.49, 926.36) |
| Republic of Zambia | 85–89 years | 915.84 (816.81, 1026.87) |
| Republic of Zambia | 90–94 years | 893.22 (725.56, 1099.63) |
| Republic of Zambia | 95+ years | 890.42 (565.23, 1402.70) |
| Republic of Zimbabwe | 30–34 years | 4.60 (3.82, 5.53) |
| Republic of Zimbabwe | 35–39 years | 9.82 (8.50, 11.36) |
[truncated: 319,826 more chars]
